# Exploring the Potential of a School Impact on Pupil Weight Status: Exploratory Factor Analysis and Repeat Cross-Sectional Study of the National Child Measurement Programme

**DOI:** 10.1371/journal.pone.0145128

**Published:** 2015-12-23

**Authors:** Andrew James Williams, Katrina M. Wyatt, Craig A. Williams, Stuart Logan, William E. Henley

**Affiliations:** 1 Farr Institute @ Scotland and Scottish Collaboration for Public Health Research and Policy, Usher Institute of Population Health Sciences and Informatics, University of Edinburgh, 20 West Richmond Street, Edinburgh EH8 9DX, United Kingdom; 2 Institute of Health Services Research, University of Exeter Medical School (formerly Peninsula College of Medicine and Dentistry), South Cloisters, St. Luke’s Campus, Exeter EX1 2LU, United Kingdom; 3 Children’s Health and Exercise Research Centre, Sport and Health Sciences, St. Luke’s Campus, University of Exeter, Exeter, EX1 2LU, United Kingdom; 4 Institute of Health Services Research, University of Exeter Medical School (formerly Peninsula College of Medicine and Dentistry), College House, St. Luke’s Campus, Exeter EX1 2LU, United Kingdom; Institute for Health & the Environment, UNITED STATES

## Abstract

Schools are common sites for obesity prevention interventions. Although many theories suggest that the school context influences weight status, there has been little empirical research. The objective of this study was to explore whether features of the school context were consistently and meaningfully associated with pupil weight status (overweight or obese). Exploratory factor analysis of routinely collected data on 319 primary schools in Devon, England, was used to identify possible school-based contextual factors. Repeated cross-sectional multilevel analysis of five years (2006/07-2010/11) of data from the National Child Measurement Programme was then used to test for consistent and meaningful associations. Four school-based contextual factors were derived which ranked schools according to deprivation, location, resource and prioritisation of physical activity. None of which were meaningfully and consistently associated with pupil weight status, across the five years. The lack of consistent associations between the factors and pupil weight status suggests that the school context is not inherently obesogenic. In contrast, incorporating findings from education research indicates that schools may be equalising weight status, and obesity prevention research, policy and practice might need to address what is happening outside schools and particularly during the school holidays.

## Introduction

The rising prevalence of overweight and obesity and the associated non-communicable diseases are among the leading public health concerns of the 21^st^ century [1–3]. Childhood is seen as a key stage of life in which to intervene to prevent the development of overweight, obesity and the associated behaviours [1,4,5]. Schools, as an environment to which the majority of children are exposed and in which they eat, drink, exercise and learn, have been perceived as important sites for obesity prevention interventions [5–7]. Consequently, there have been a large number of school-based obesity interventions [4,8–12], however, a recent systematic review concluded that they had only been moderately effective [10]. Data from the National Child Measurement Programme (NCMP) in England have identified that the prevalence of obesity doubles during the period of primary education (ages 4–11 years), which may indicate that primary schools may be crucial to the development and prevention of obesity [13–17].

The World Health Organization health promoting schools initiative recognises the importance of the school context, however, few school-based obesity prevention interventions are developed to be adapted to each schools context [18]. Bonell et al. [5] recently reported a systematic review of both quantitative and qualitative evidence on the effect of school and school environment on health. The review, concluded that the empirical evidence supported a number of the theories as to how schools affect health such as the theories of human functioning and school organisation, social capital and social development [5]. Bonell et al. [5] called for future school-based health research to be informed by theory and examine a broader range of health and education outcomes than those examined in the studies reviewed (e.g. substance misuse, violence, physical activity and healthy eating). The review highlighted the strong theoretical basis for a school effect on pupil weight status but emphasised the lack of empirical research [5]. Procter et al. [19] undertook a cross-sectional ‘value-added’ analysis of 2,367 children attending 35 primary schools in Leeds, England, seeking to address the question of a school effect on pupil weight status. Their findings led them to conclude that schools had an independent impact upon children’s weight status [19]. However, this study lacked the power to evaluate which elements of the school context might be associated with the school effect on weight status.

We previously undertook an analysis replicating the methods proposed by Procter et al. [19] across five years [20]. Although we were able to replicate their findings within any single year, each schools impact (value-added ranking) varied considerably across the five years [20]. We therefore concluded that the effect observed by Procter et al. [19] did not reflect a systematic differential school impact on pupil weight status [20]. However, the possibility remains that there are features of the school context that influence weight status but that these are not evident when studying consistency of school rankings over time because the rankings are sensitive to other factors including the characteristics of the pupils in each yearly cohort [21]. Furthermore, studying associations between school context and pupil weight status related to a systematic *common* school impact, is complicated by the difficulty of collecting data on a suitably large matched control sample of children who do not attend school. In this study we explored the impact of measured characteristics of the school context on pupil weight status by exploiting the differential exposure to the school context of pupils in the different year groups, measured within the NCMP. Recognising that the school context is not one dimensional and heeding the danger of over adjustment raised by Bonell et al. [5], we first used exploratory factor analysis of variables related to school context to derive a robust set of interpretable school-based contextual factors that summarise the key sources of variation between schools [22–24]. Subsequently, we tested for consistent associations between pupil weight status and the school-based contextual factors, to explore the possibility that common features shared by groups of schools may have a systematic impact on pupil weight status.

## Materials and Methods

The NCMP was introduced in England in 2005 in order to monitor progress towards a 2004 Public Service Agreement to reduce the prevalence of obesity among children less than 11 years old to the level reported in 2000 [25,26]. Within the programme, each year the children in the first (Reception, 4–5 year olds) and last (Year 6, 10–11 year olds) years of primary education at state-maintained primary schools are weighed and measured by health professionals. The data are collected in order that body mass index standard deviation scores (BMI-SDSs) relative to the UK 1990 growth reference can be calculated [27,28]. Subsequently each individual is categorised by weight status (underweight, healthy weight, overweight or obese) according to the epidemiological definition for monitoring purposes [28].

The data in this study relate to the Local Authority of Devon, a large county in the South West of England, with a mix of rural and urban, isolated and dense dwellings, including the city of Exeter. Although the urban areas of Plymouth and Torbay are within the county of Devon they are governed by separate unitary authorities and hence not included in this study. Devon has two coastlines, low levels of deprivation and low ethnic diversity; summary characteristics of the study sample, Devon and England as a whole are presented in Table 1 [29]. Due to low numbers some of the ethnicity categories were combined; Chinese with Asian or Asian British and Black or Black British with Any other ethnic group [30]. Reports on the NCMP note that the South West has a statistically lower than average prevalence of obese children, however, there is a statistically higher than average prevalence of overweight children in Reception, but not Year 6 across the region (Table 1) [14–17].

**Table 1:** Devon schools compared with national schools data 2008/09 to 2010/11^a^.

	2008/09			2009/10			2010/11		
	Study	Devon	England	Study	Devon	England	Study	Devon	England
**Pupils**									
Gender mix (percentage female)	48.6%	48.6%	48.9%	48.2%	48.6%	49.0%	48.2%	48.5%	48.7%
Ethnicity									
White—British	94.7%	94.5%	75.3%	94.4%	94.3%	74.3%	94.2%	94.3%	73.3%
Any other white background	2.5%	2.6%	4.5%	2.5%	2.5%	4.7%	2.7%	2.3%	4.9%
Chinese, Asian or Asian British	0.5%	0.7%	9.7%	0.5%	0.7%	10.0%	0.7%	0.8%	10.4%
Mixed/dual ethnicity	1.6%	1.5%	4.2%	1.8%	1.6%	4.4%	1.6%	1.8%	4.6%
Any other ethnic group^b^	0.7%	0.7%	6.3%	0.8%	0.9%	6.6%	0.8%	0.9%	6.8%
Percentage for whom English is an additional language^c^	0.7% (0.0%, 2.1%)	2.1%	15.3%	0.9% (0.0%, 2.1%)	2.4%	16.0%	0.9% (0.0%, 2.4%)	2.6%	16.8%
Percentage eligible for free school meals^c^	7.3% (4.1%, 13.2%)	10.5%	17.1%	8.1% (5.1%, 13.5%)	11.6%	18.5%	9.3% (5.2%, 15.2%)	12.3%	19.2%
Percentage using active transport^c^	45.5% (24.7%, 64.5%)	-	-	47.5% (26.8%, 65.3%)	-	-	49.6% (28.6%, 66.9%)	56.1%	60.5%
Percentage Special Educational Needs^c^	14.9% (11.2%, 20.3%)	-	19.7%	15.3% (11.2%, 21.0%)	-	20.0%	15.6% (11.2%, 21.0%)	-	19.3%
Percentage overweight^d^									
Reception	15.4%	14.5% to 16.3%	13.1% to 13.3%	14.7%	13.8% to 15.6%	13.2% to 13.4%	14.7%	13.8% to 15.6%	13.1% to 13.3%
Year 6	14.3%	13.5% to 15.1%	14.2% to 14.4%	14.8%	14.0% to 15.6%	14.5% to 14.7%	14.1%	13.3% to 14.9%	14.3% to 14.5%
Percentage obese^d^									
Reception	9.8%	9.1% to 10.5%	9.5% to 9.7%	8.8%	8.1% to 9.5%	9.7% to 9.9%	8.8%	8.1% to 9.5%	9.3% to 9.5%
Year 6	16.1%	15.2% to 17.0%	18.2% to 18.4%	15.8%	14.9% to 16.7%	18.6% to 18.8%	16.1%	15.2% to 17.0%	18.9% to 19.1%
**School**									
Mean number of pupils per school	-	164.3	231.3	-	164.6	233.6	-	164.9	237.1
Governance									
Community	61.7%	61.4%	61.1%	62.3%	61.6%	60.8%	62.6%	61.2%	60.1%
Voluntary controlled	20.1%	20.3%	14.8%	19.9%	20.3%	14.8%	19.9%	20.4%	14.9%
Voluntary aided	18.2%	18.4%	21.8%	17.9%	18.1%	21.8%	17.6%	18.2%	21.9%
Other	0.0%	0.0%	2.4%	0.0%	0.0%	2.5%	0.0%	0.3%	3.1%
Religious denomination									
None	61.4%	61.4%	63.5%	61.9%	61.6%	63.4%	62.3%	61.5%	63.3%
Church of England	35.6%	35.4%	25.9%	35.1%	35.2%	26.0%	34.8%	35.4%	26.1%
Roman Catholic	3.0%	2.9%	9.9%	3.0%	2.9%	9.9%	3.0%	2.9%	9.9%
Other	0.0%	0.3%	0.7%	0.0%	0.3%	0.7%	0.0%	0.3%	0.8%

Devon and National data taken from the Department for Education January statistics for each year [29] and the NHS Information Centre annual National Child Measurement Programme report [15–17].

^a^2006/07 or 2007/08 Devon and National data was not available for all variables through the Department for Education [29].

^b^This ethnicity category includes Black and Black British [30].

^c^The study values for these data are based upon the median and interquartile range of schools, whereas the Devon and UK data are the proportions of pupil

^d^Devon and National values are 95% confidence intervals

- No equivalent data available.

### Individual weight status data from the NCMP

Following approval from the institution’s ethics committee and the Caldicott guardian, five years (2006/07 to 2010/11) of anonymised cross-sectional data from the NCMP were received from the primary care trust (NHS Devon) to form the individual data for this study. Across the five years there were 62,554 participants with valid BMI-SDS which had been checked internally, as well as according to the national validity checks [30]. Two binary outcome variables were generated indicating those categorised as overweight (1.04≤BMI-SDS<1.64) or obese (BMI-SDS≥1.64). Within Devon, children and parents are required actively to opt out of the NCMP and no data are collected on non-participants. However, NHS Devon were able to provide data on the proportions of each school year group which did not participate. Across the five years analysed participation in Devon was lowest in 2006/07 (82%) and rose to 91% in 2010/11 [13–17].

The anonymised NCMP dataset contained the following demographic data on each participant: age in months at time of measurement, gender and ethnicity. The location of participant’s home was indicated by their Lower Super Output Area (LSOA) [31]. The LSOA data were used to link individuals and schools to the following indices of area deprivation: Index of Multiple Deprivation (IMD), Income Deprivation Affecting Children Index (IDACI) and Child Wellbeing Index (CWI) [32,33]. Multiple indices of deprivation were examined due to the previously identified association between weight status and socioeconomic status [26]. In order that the indices were comparable they were rescaled nationally prior to linking with the dataset, so that 1 was the most deprived and 0 the least deprived LSOA in England.

### School contextual data

A reference number for each participant’s school and the school’s LSOA were provided as part of the NCMP data extract. A wide variety of school data were sought relating to demographics, socioeconomic status, built environment, physical activity, diet and ethos from multiple sources. The selection of school data was informed by previous research [24,34–39]. The acquired data were combined into a school dataset as outlined in S1 File, however, due to the widely recognised difficulties in collecting dietary data, no routinely collected sources of such data were identified. Only a few variables (e.g. school building age, catchment area and SATs pass rate) contained large quantities of missing data. Therefore, it was felt prudent not to include these three variables in the exploratory factor analysis, but otherwise to undertake a complete case analysis.

The school variables collected reflected the compositional (e.g. ethnic mix), collective (e.g. Healthy School award), as well as contextual (e.g. location) aspects of the school as discussed by Macintyre et al. [40]. As the school composition varies from year to year, and Year 6 pupils would have been exposed to the school environment for a number of years, each of the compositional variables was averaged across the six years of primary education. This assumed that children remained at the same school for their entire primary education and the effect of each year was equal, which although unlikely to be absolutely true was a useful preliminary assumption. Reception pupils' values for the compositional variables were those of the year of measurement. Summary statistics and histograms of each school variable were examined and highly skewed variables were categorised as shown in S1 File. The school and individual datasets were merged using the school reference number.

### Analysis

In line with the study purpose there were two phases to the analysis; firstly deriving the school-based contextual factors and, secondly testing the impact of the school-based contextual factors on pupil weight status. All data preparation and analysis was undertaken in Stata IC 11 [41].

#### Phase 1: derivation of school-based contextual factors

Any impact of school context on pupil weight status would be expected to be more evident among those with more exposure to the school context (Year 6 compared with Reception). Hence, only the school variables related to Year 6 pupils were used to derive the school-based contextual factors. Furthermore, as the intention was to test the consistency of the associations between pupil weight status and school context, the school-based contextual factors were developed using the 2010/11 data as, within the dataset this was the year with the highest NCMP participation, resulting in one observation per school (n = 289). With the majority of school variables being categorical the factor analysis was based on the polychoric correlation matrix [42]. This required that all the categorical variables were either ordinal or binary and therefore school governance and denomination were transformed into binary variables (community or other and not-religious or other respectively). As well as excluding school building age, catchment area and SATs pass rate from the factor analysis three further variables were excluded. Governance and denomination were almost identical variables and therefore only governance was included. By 2010/11 all schools were required to have a travel plan and therefore this variable was excluded. Non-participation was excluded from the factor analysis as it related to the NCMP and not the school context, but to assess how non-participation may have biased the results, we adjusted for non-participation in all models in phase 2. Promax rotation was used to calculate the school-based contextual factors as it does not rely on the variables being uncorrelated (non-orthogonal). Examination of the variables with highest loadings in each school-based contextual factor and data from the excluded variables was then used to define a label for each school-based contextual factor.

#### Phase 2: testing the impact of school context on pupil weight status

A series of logistic regression models were developed to explore the associations between the derived school-based contextual factors and pupil weight status (overweight or obese). As the data were hierarchical, the primary analysis involved fitting multilevel logistic regression models with a random school effect (intercept). Non-hierarchical (single level) models were also estimated in order to provide some insight into the impact of the various multilevel structures evaluated. Having hypothesised that the school context would have greater impact on Year 6 than Reception pupils, the two year groups were examined separately in two-level models (pupils within schools). Three-level models (pupils within year groups within schools) were also developed in order to be able to explore the differences within schools (between year groups, akin to ‘value-added’) as well as between schools and the interactions between year group and the school-based contextual factors. Neighbourhood has been considered an important context in the child’s life. However, our previous research, and that of Procter et al. [19], found cross-classification by neighbourhood to have little impact upon the results, and hence cross-classification was not undertaken in the present study [20].

In line with our aim to examine not only significance but also consistency, each year (2006/07 to 2010/11) was analysed separately. Initially empty null models were examined to identify the intraclass correlation coefficients (ICCs). Due to the underlying logistic distribution the individual variation in the null models was assumed to be *π* ^2^/3 [37,43]. Subsequently, the derived school-based contextual factors were examined individually in models which were adjusted for individual gender, ethnicity and socioeconomic status (based on previous analyses IMD was chosen as the sole individual measure of socioeconomic status [39]) and NCMP non-participation. All analyses were two-tailed with *α* = 0.05. Restricted maximum likelihood estimation and numerical integration with seven quadrature points were used for the multilevel analyses. The normality and homoscedasticity of the residuals from the school and year group levels of each model were assessed for any violation of assumptions using graphical methods.

Caterpillar plots were constructed from the fitted models to quantify each school’s residual effect upon pupil weight status after adjustment for the fitted variables. The width of the confidence intervals was set at ±1.4 times the standard error in order that 5% significance was maintained, given that the ratio between the majority of pairs of standard errors did not exceed 1:2 [43,44]. Plots of the school-level residuals from the two-level and three-level models, illustrated the differences *between* schools. However, caterpillar plots of the difference in year group (Year 6-Reception) residuals from the three-level models indicated the differences between year groups *within* schools (‘value-added’).

## Results

Descriptive characteristics of the individual data can be found in Table 2 and school data in S1 File. The mean age of the children in each year group decreased slightly over the five years, while the gender balance remained fairly constant with slightly more males than females. Around 95% of the sample were White—British and the levels of deprivation were low. The BMI-SDS and weight categories reflect the national pattern of steady increase in weight status; however, in 2008/09 there was a larger increase in BMI-SDS, overweight and obesity. This coincides with a change in the NCMP legislation to enable letters be sent to parents informing them of their child's weight status [45]. Whilst it might have been expected that parents of overweight or obese children would not have wanted to know their child's weight status and therefore prevented the child participating, this would have resulted in a drop in prevalence of obesity; furthermore, the proportion of pupils participating in the NCMP continued to rise across this time period [13–17]. Therefore the jump in prevalence requires further exploration beyond the current study. Additionally, contained in Table 2 is information on the distribution of pupil weight status within schools. These values demonstrate that there is marked variation in pupil weight status between schools, with the prevalence of overweight and obesity varying by more than 0.1 (10%).

**Table 2:** Individual characteristics.

Variable	Summary statistic^a^						Missing data
	2006/07	2007/08	2008/09	2009/10	2010/11	Total	
	n = 11,492	n = 12,349	n = 12,609	n = 13,028	n = 13,076	n = 62,554	
Gender							
Males	51.28%	52.53%	51.45%	51.77%	51.80%	51.77%	0
Females	48.72%	47.47%	48.55%	48.23%	48.20%	48.23%	
Age (years)							
Reception	5.23±0.28	5.18±0.28	5.04±0.27	5.05±0.27	5.02±0.26	5.10±0.28	0
Year 6	11.33±0.30	11.32±0.30	11.02±0.30	10.92±0.34	10.84±0.34	11.08±0.37	
Ethnicity^b^							
White—British	95.63%	94.59%	94.68%	94.40%	94.18%	94.68%	3,802
Any other white background	2.12%	2.59%	2.53%	2.48%	2.74%	2.50%	
Chinese, Asian or Asian British	0.50%	0.85%	0.52%	0.52%	0.73%	0.62%	
Mixed/dual ethnicity	1.19%	1.43%	1.59%	1.79%	1.57%	1.52%	
Any other ethnic group	0.56%	0.53%	0.68%	0.82%	0.78%	0.67%	
Index Multiple Deprivation 2010^c^	0.18 (0.13,0.24)	0.18 (0.13,0.24)	0.18 (0.12,0.24)	0.18 (0.13,0.24)	0.18 (0.12,0.24)	0.18 (0.13,0.24)	1,137
Income Deprivation Affecting Children Index 2010^c^	0.12 (0.08,0.18)	0.12 (0.08,0.18)	0.12 (0.08,0.18)	0.12 (0.08,0.18)	0.12 (0.08,0.18)	0.12 (0.08,0.18)	1,458
Child Wellbeing Index 2009^c^	0.20 (0.14,0.27)	0.20 (0.14,0.27)	0.20 (0.14,0.27)	0.20 (0.14,0.27)	0.20 (0.14,0.27)	0.20 (0.14,0.27)	1,137
BMI-SDS	0.35±1.08	0.32±1.08	0.46±1.05	0.43±1.06	0.45±1.03	0.40±1.06	0
Underweight (UK90)	1.23%	1.17%	0.76%	0.79%	0.69%	0.92%	0
Healthy Weight (UK90)	73.52%	74.22%	71.32%	72.02%	72.48%	72.69%	0
Overweight (UK90)	13.44%	13.35%	14.84%	14.75%	14.42%	14.18%	0
Obese (UK90)	11.82%	11.27%	13.08%	12.44%	12.40%	12.22%	0
School year group^d^							
BMI-SDS							
Reception	0.32±0.37	0.29±0.39	0.45±0.32	0.45±0.42	0.47±0.33	-	0
Year 6	0.40±0.38	0.34±0.36	0.47±0.38	0.49±0.38	0.48±0.36	-	0
Overweight (UK90)							
Reception	0.13 (0.05, 0.20)	0.12 (0.05, 0.18)	0.14 (0.07, 0.22)	0.14 (0.08, 0.22)	0.14 (0.09, 0.20)	-	0
Year 6	0.12 (0.07, 0.19)	0.13 (0.06, 0.19)	0.14 (0.07, 0.22)	0.14 (0.08, 0.21)	0.13 (0.08, 0.20)	-	0
Obese (UK90)							
Reception	0.07 (0.00, 0.13)	0.07 (0.00, 0.11)	0.07 (0.00, 0.13)	0.07 (0.00, 0.13)	0.08 (0.00, 0.13)	-	0
Year 6	0.13 (0.07, 0.20)	0.13 (0.06, 0.19)	0.14 (0.09, 0.22)	0.15 (0.09, 0.23)	0.15 (0.08, 0.22)	-	0

BMI-SDS: body mass index standard deviation score, UK90: United Kingdom 1990 reference population and categorisation.

^a^Summary statistics are percentages (%) for categorical variables, means±standard deviations for data which is approximately normally distributed, and otherwise median (interquartile range: 25^th^ and 75^th^ percentiles).

^b^These ethnicity categories are derived from the Department for Children, Schools and Families categories and sub categories, due to small numbers the Chinese and Asian or Asian British categories were combined as was the Black or Black British category and the Any other ethnic group category [30].

^c^Nationally rescaled

^d^The school year group rows are for BMI-SDS the mean±standard deviation of each school year group mean BMI-SDS, whereas for overweight and obesity the values are median (interquartile range) proportion overweight or obese of each school year group.

### Phase 1: derivation of school-based contextual factors

We initially examined the extent to which the factors identified changed depending on which combination of school variables were included in the factor analysis, (to assess the robustness of the factors). Furthermore, in recognition that deprivation is a recognised determinant of weight status, we wanted to see whether deprivation as a factor could be isolated from other aspects of the school context. The experimentation primarily consisted of varying the number of measures of school socioeconomic status included in the factor analysis, while also comparing the results of the promax rotation with those from varimax rotation. We found little variation in the nature of the factors identified with the inclusion and exclusion of variables and between types of rotation and therefore settled on the pragmatic approach of including all the deprivation variables (results not included but available from the corresponding author on request). Subsequently, four school-based contextual factors were identified with eigenvalues greater than 1 and the Kaiser-Meyer-Olkin measure of sampling adequacy was 0.55 indicating that factor analysis was appropriate (Bartlett’s test p<0.0001). Table 3 lists the composition and definition of the four factors, where loading values of less than 0.3 have been suppressed. In order to test the stability of each factor, the scores for a school with a high, medium and low score in 2010/11 were compared across the five years and between year groups. These results can be found in S2 File, alongside the polychoric correlation matrix from which the factors were derived.

**Table 3:** School-based contextual factor composition and definition.

Variable	Factor 1	Factor 2	Factor 3	Factor 4	Uniqueness
School pupil capacity		0.8065			0.2118
Proportion female					0.9623
Proportion not White—British		0.3851			0.8506
School location IMD 2010 (rescaled)	0.8996				0.2483
School location IDACI 2010 (rescaled)	0.7828				0.2540
School location CWI 2009 (rescaled)	0.9123				0.2080
Proportion eligible for FSM	0.6222	0.3266			0.3687
School mean IMD 2010 (rescaled)	0.9262				0.1960
School mean IDACI 2010 (rescaled)	0.7885				0.1698
School mean CWI 2009 (rescaled)	0.8750				0.2583
School location (from rural to urban)		0.9592			0.0748
Coastal catchment area				0.3545	0.8445
School has multiple sites			0.5348		0.6216
Grass play area (m^2^/pupil)			0.7816		0.3993
Hard surface play area (m^2^/pupil)					0.7976
Total site area (m^2^/pupil)			0.8066		0.1793
Proportion of pupils walking or cycling		0.6235			0.4852
School PEDPASS award				0.6367	0.5750
School Active Lifestyle award				0.5888	0.6588
School governance					0.8610
School over- or under-subscribed				0.5193	0.7222
Proportion of pupils for whom EAL		0.8073			0.3720
Proportion with SEN	0.5606				0.4987
Devon Healthy School Award (timing)					0.9593
Eigen value	6.0736	3.3388	1.4703	1.3401	
**Definition**	Deprivation	Location	Resource	Prioritisation of physical activity	
Factor mean±standard deviation	0.69±0.26	4.80±2.10	2.05±0.48	2.63±1.05	

KMO = 0.5464, promax rotation. Factor loadings less than 0.3 have been left blank.

CWI; Child wellbeing index, EAL; English as an additional language, IDACI; Income deprivation affecting children index, IMD; Index of multiple deprivation, PEDPASS; Physical Education, Daily Physical Activity and School Sports, SEN; Special educational needs.

#### Factor 1

The variables which loaded most strongly in factor 1 were the measures of school socioeconomic status and proportion of pupils with special educational needs. Profiling schools with high and low factor scores identified that the schools with lowest scores were large schools with low deprivation, prevalence of special education needs and use of active travel (walking or cycling) to school, which tended to be oversubscribed. Whereas, schools with high scores for factor 1 were high deprivation, smaller schools with more special educational needs and higher tendency to active travel. Henceforth factor 1 will be referred to as *Deprivation*.

#### Factor 2

The variables which loaded most strongly in factor 2 were: school capacity, proportion not White—British, proportion eligible for free school meals, school location, proportion walking or cycling to school and proportion for whom English was an additional language. Schools with low scores for factor 2 were small rural schools with older buildings, large catchment areas, classes containing pupils from different year groups and little to no ethnic diversity. The high score schools were large urban schools with modern buildings, and more ethnic diversity. Factor 2 will be referred to as *Location*.

#### Factor 3

The variables which loaded most strongly in factor 3 were: an indicator of whether the school was split over more than one site (e.g. separate playing field), grass play area and site area per pupil. Profiling schools identified that factor 3 ranked schools from (low scores) large rural community governed schools that have received recent investment, to (high scores) older small urban (town centre) voluntary (church) governed schools with little access to grass play area. Notably a number of the schools with low scores have subsequently become academies. Subsequently, factor 3 has been labelled *Resource*.

#### Factor 4

The variables which loaded most strongly in factor 4 were: an indicator of whether the school catchment area included a coastline, school achievement of an Active Lifestyle or PEDPASS (Physical Education, Daily Physical Activity and School Sports) award and subscription (whether the number of pupils was markedly higher (oversubscribed) or lower (undersubscribed) than the school capacity). Factor 4 ranked schools similarly to factor 3 from large catchment area rural schools to small urban schools with increased active travel. There also appeared to be some association with school inspections (Office for Standards in Education (Ofsted) reports) with lower scoring schools receiving consistent Good grades, whereas those with higher scores were improving or in need of improvement. Schools with lower scores, compared to those with higher scores, had more quickly achieved Healthy School status particularly focussing on pupil emotional health and wellbeing but had not received the more physical activity specific awards (Active Lifestyle and PEDPASS) [46,47]. Consequently, factor 4 was labelled *Prioritisation of physical activity*.

Notably, none of the measures of school socioeconomic status loaded heavily into factors 3 or 4.

### Phase 2: testing the impact of school context on pupil weight status

The final (complete) analysis included 55,826 pupils (Table 4). The ICCs of the null models are listed in Table 4 with less than 3% of the variation in pupil overweight status and less than 9% of pupil obesity status attributed to differences between schools or year groups. Contrary to expectations, in the two level-models the school ICCs tended to be larger for Reception than Year 6 pupils. In the three-level models the school ICC was generally <0.1%, indicating that any clustering was due to differences between year groups rather than between schools. Overall, the school and year group ICCs varied from year to year.

**Table 4:** Null model intraclass correlation coefficients.

Year	Logistic regression model	IPV		Year group ICC		School ICC		n	
		Overwgt	Obese	Overwgt	Obese	Overwgt	Obese	Overwgt	Obese
2006/07	Single level	1.000	1.000	-	-	-	-	8,747	9,910
	Reception 2-level	0.975	1.000	-	-	0.025	<0.001	4,002	4,352
	Year 6 2-level	0.994	0.992	-	-	0.006	0.008	4,745	5,558
	Three-level	0.985	0.964	0.015	0.036	<0.001	<0.001	8,747	9,910
2007/08	Single level	1.000	1.000	-	-	-	-	10,061	11,353
	Reception 2-level	0.996	0.973	-	-	0.004	0.027	4,769	5,190
	Year 6 2-level	0.995	0.974	-	-	0.005	0.026	5,292	6,163
	Three-level	0.993	0.945	0.005	0.055	0.002	<0.001	10,061	11,353
2008/09	Single level	1.000	1.000	-	-	-	-	10,149	11,696
	Reception 2-level	0.974	0.958	-	-	0.026	0.042	4,889	5,421
	Year 6 2-level	0.987	0.973	-	-	0.013	0.027	5,260	6,275
	Three-level	0.980	0.948	0.020	0.052	<0.001	<0.001	10,149	11,696
2009/10	Single level	1.000	1.000	-	-	-	-	10,356	11,851
	Reception 2-level	0.992	0.915	-	-	0.008	0.085	4,908	5,373
	Year 6 2-level	0.992	0.978	-	-	0.008	0.022	5,448	6,478
	Three-level	0.991	0.926	0.008	0.074	0.001	<0.001	10,356	11,851
2010/11	Single level	1.000	1.000	-	-	-	-	9,604	11,016
	Reception 2-level	0.995	0.969	-	-	0.005	0.031	4,520	4,970
	Year 6 2-level	1.000	0.990	-	-	<0.001	0.010	5,084	6,046
	Three-level	0.999	0.956	<0.001	0.044	<0.001	<0.001	9,604	11,016

ICC; intraclass correlation coefficient, IPV; individual proportion of variation, Overwgt; overweight (body mass index standard deviation score ≥85^th^ percentile and >95^th^ percentile of the United Kingdom 1990 reference population [27]), Obese; obesity (body mass index standard deviation score ≥95% percentile of the United Kingdom 1990 reference population [27]).

The results of the models fitted to test the significance and consistency of the associations between school based contextual factors and pupil weight status are presented in Table 5. Given the large number of statistical tests involved, the results have been presented as effect sizes (Cohen’s d values converted from odds ratios [48]), placing the emphasis on meaningful rather than solely statistical significance. Due to a sparse matrix in some models it was necessary to estimate the models in R [49] using lme4 [50] and then transfer the residuals back into Stata.

**Table 5:** Effect sizes (UK90).

	2006/07		2007/08		2008/09		2009/10		2010/11	
	ES	95% CI	ES	95% CI	ES	95% CI	ES	95% CI	ES	95% CI
**Factor 1—Deprivation**										
*Overweight*										
Single level	-0.001	-0.131 to 0.130	0.120	-0.002 to 0.242	-0.035	-0.150 to 0.081	-0.050	-0.163 to 0.063	0.026	-0.099 to 0.151
Reception two-level	0.055	-0.162 to 0.272	0.150	-0.035 to 0.335	0.045	-0.143 to 0.233	-0.036	-0.211 to 0.139	0.079	-0.111 to 0.269
Year 6 two-level	-0.063	-0.242 to 0.116	0.098	-0.067 to 0.263	-0.129	-0.300 to 0.043	-0.032	-0.193 to 0.129	-0.019	-0.187 to 0.148
Three-level	0.005	-0.134 to 0.144	0.121	-0.004 to 0.245	-0.047	-0.175 to 0.080	-0.047	-0.167 to 0.072	0.025	-0.102 to 0.151
Interaction (main effect)	0.052	-0.149 to 0.253	*0*.*181*	*0*.*001 to 0*.*361*	0.054	-0.125 to 0.233	-0.066	-0.236 to 0.104	0.073	-0.109 to 0.255
Interaction (interaction effect)	-0.082	-0.350 to 0.185	-0.097	-0.330 to 0.135	-0.199	-0.442 to 0.045	0.051	-0.174 to 0.275	-0.088	-0.325 to 0.149
*Obese*										
Single level	0.055	-0.082 to 0.193	*0*.*138*	*0*.*008 to 0*.*268*	***0*.*236***	***0*.*114 to 0*.*358***	0.076	-0.045 to 0.197	*0*.*178*	*0*.*049 to 0*.*308*
Reception two-level	**0.201**	**-0.044 to 0.446**	0.170	-0.065 to 0.404	***0*.*346***	***0*.*111 to 0*.*581***	**0.240**	**-0.019 to 0.499**	0.179	-0.065 to 0.422
Year 6 two-level	0.010	-0.166 to 0.187	0.156	-0.024 to 0.337	***0*.*221***	***0*.*051 to 0*.*391***	0.071	-0.096 to 0.238	*0*.*166*	*0*.*008 to 0*.*325*
Three-level	0.079	-0.080 to 0.237	0.140	-0.016 to 0.296	***0*.*248***	***0*.*101 to 0*.*396***	0.081	-0.076 to 0.237	*0*.*168*	*0*.*020 to 0*.*316*
Interaction (main effect)	**0.201**	**-0.041 to 0.443**	***0*.*235***	***0*.*003 to 0*.*466***	***0*.*311***	***0*.*092 to 0*.*530***	***0*.*237***	***0*.*008 to 0*.*467***	0.175	-0.046 to 0.395
Interaction (interaction effect)	-0.183	-0.467 to 0.102	-0.111	-0.380 to 0.158	-0.072	-0.335 to 0.190	-0.171	-0.443 to 0.101	-0.003	-0.260 to 0.254
**Factor 2—Location**										
*Overweight*										
Single level	-0.004	-0.021 to 0.013	0.013	-0.003 to 0.029	-0.005	-0.020 to 0.010	*-0*.*015*	*-0*.*030 to -0*.*001*	0.005	-0.011 to 0.020
Reception two-level	0.002	-0.026 to 0.029	0.003	-0.021 to 0.026	0.009	-0.015 to 0.033	-0.021	-0.043 to 0.002	0.010	-0.013 to 0.034
Year 6 two-level	-0.011	-0.033 to 0.012	0.021	<-0.001 to 0.043	-0.020	-0.042 to 0.002	-0.006	-0.027 to 0.014	<0.001	-0.021 to 0.022
Three-level	-0.004	-0.021 to 0.014	0.013	-0.003 to 0.029	-.0.007	-0.023 to 0.009	-0.015	-0.030 to <0.001	0.005	-0.011 to 0.021
Interaction (main effect)	0.003	-0.023 to 0.029	0.006	-0.018 to 0.029	0.009	-0.014 to 0.032	*-0*.*023*	*-0*.*045 to -0*.*001*	0.009	-0.014 to 0.032
Interaction (interaction effect)	-0.013	-0.047 to 0.022	0.014	-0.017 to 0.046	-0.030	-0.062 to 0.002	0.016	-0.014 to 0.046	-0.008	-0.039 to 0.023
*Obese*										
Single level	-0.005	-0.022 to 0.013	0.017	<-0.001 to 0.034	*0*.*027*	*0*.*011 to 0*.*043*	-0.002	-0.017 to 0.014	0.006	-0.011 to 0.022
Reception two-level	-0.001	-0.031 to 0.030	0.013	-0.018 to 0.043	*0*.*051*	*0*.*020 to 0*.*081*	0.017	-0.016 to 0.051	-0.004	-0.035 to 0.027
Year 6 two-level	-0.006	-0.029 to 0.016	0.019	-0.004 to 0.042	0.019	-0.003 to 0.041	-0.002	-0.024 to 0.019	0.011	-0.010 to 0.031
Three-level	-0.003	-0.023 to 0.018	0.014	-0.006 to 0.034	*0*.*028*	*0*.*009 to 0*.*047*	-0.002	-0.022 to 0.018	0.005	-0.014 to 0.024
Interaction (main effect)	<0.001	-0.031 to 0.032	0.017	-0.014 to 0.048	*0*.*047*	*0*.*018 to 0*.*077*	0.018	-0.013 to 0.049	-0.004	-0.033 to 0.024
Interaction (interaction effect)	-0.006	-0.044 to 0.031	0.001	-0.036 to 0.037	-0.027	-0.062 to 0.009	-0.022	-0.058 to 0.015	0.015	-0.019 to 0.049
**Factor 3—Resource**										
*Overweight*										
Single level	0.013	-0.067 to 0.092	0.038	-0.033 to 0.108	-0.016	-0.083 to 0.051	0.005	-0.062 to 0.071	0.013	-0.058 to 0.085
Reception two-level	0.154	0.019 to 0.289	0.080	-0.033 to 0.193	<-0.001	-0.111 to 0.110	-0.032	-0.140 to 0.076	-0.008	-0.120 to 0.105
Year 6 two-level	-0.086	-0.192 to 0.021	-0.001	-0.094 to 0.092	-0.049	-0.149 to 0.050	0.040	-0.052 to 0.132	0.023	-0.072 to 0.117
Three-level	0.019	-0.067 to 0.104	0.038	-0.035 to 0.111	-0.025	-0.099 to 0.049	0.008	-0.063 to 0.078	0.013	-0.060 to 0.085
Interaction (main effect)	*0*.*144*	*0*.*016 to 0*.*272*	0.090	-0.022 to 0.202	0.009	-0.099 to 0.116	-0.042	-0.149 to 0.065	-0.002	-0.112 to 0.108
Interaction (interaction effect)	***-0*.*223***	***-0*.*392 to -0*.*054***	-0.089	-0.234 to 0.055	-0.063	-0.211 to 0.085	0.089	-0.051 to 0.229	0.024	-0.120 to 0.168
*Obese*										
Single level	-0.045	-0.129 to 0.040	0.035	-0.040 to 0.110	0.014	-0.055 to 0.084	-0.026	-0.097 to 0.045	-0.004	-0.079 to 0.071
Reception two-level	0.009	-0.145 to 0.163	0.055	-0.089 to 0.199	0.027	-0.110 to 0.165	0.064	-0.091 to 0.219	0.013	-0.132 to 0.159
Year 6 two-level	-0.073	-0.178 to 0.032	0.030	-0.073 to 0.133	0.006	-0.092 to 0.104	-0.043	-0.139 to 0.052	-0.035	-0.127 to 0.057
Three-level	-0.033	-0.130 to 0.064	0.050	-0.042 to 0.141	0.015	-0.071 to 0.101	-0.021	-0.113 to 0.070	-0.011	-0.098 to 0.076
Interaction (main effect)	0.010	-0.145 to 0.166	0.076	-0.070 to 0.221	0.025	-0.108 to 0.157	0.058	-0.084 to 0.201	0.018	-0.119 to 0.155
Interaction (interaction effect)	-0.079	-0.264 to 0.106	-0.050	-0.220 to 0.120	-0.011	-0.172 to 0.150	-0.100	-0.270 to 0.070	-0.048	-0.210 to 0.115
**Factor 4—Prioritisation of physical activity**										
*Overweight*										
Single level	-0.008	-0.041 to 0.024	0.019	-0.012 to 0.049	-0.012	-0.041 to 0.016	-0.015	-0.043 to 0.014	0.015	-0.016 to 0.046
Reception two-level	0.008	-0.046 to 0.062	0.003	-0.045 to 0.050	0.009	-0.037 to 0.056	-0.035	-0.080 to 0.011	0.012	-0.034 to 0.058
Year 6 two-level	-0.024	-0.066 to 0.019	0.028	-0.014 to 0.069	-0.037	-0.079 to 0.006	0.005	-0.035 to 0.045	0.018	-0.024 to 0.060
Three-level	-0.008	-0.042 to 0.026	0.019	-0.013 to 0.050	-0.015	-0.047 to 0.016	-0.015	-0.045 to 0.015	0.015	-0.016 to 0.046
Interaction (main effect)	0.010	-0.041 to 0.061	0.012	-0.035 to 0.059	0.010	-0.035 to 0.056	-0.039	-0.084 to 0.005	0.010	-0.034 to 0.055
Interaction (interaction effect)	-0.032	-0.099 to 0.036	0.013	-0.049 to 0.074	-0.050	-0.112 to 0.013	0.045	-0.014 to 0.105	0.008	-0.052 to 0.069
*Obese*										
Single level	-0.017	-0.050 to 0.017	0.023	-0.009 to 0.056	0.028	-0.002 to 0.058	0.005	-0.026 to 0.035	<0.001	-0.032 to 0.032
Reception two-level	<-0.001	-0.061 to 0.060	0.007	-0.054 to 0.068	0.049	-0.011 to 0.108	0.025	-0.042 to 0.091	-0.013	-0.073 to 0.047
Year 6 two-level	-0.021	-0.063 to 0.021	0.030	-0.015 to 0.075	0.026	-0.017 to 0.069	-0.002	-0.043 to 0.039	0.011	-0.029 to 0.051
Three-level	-0.013	-0.052 to 0.026	0.018	-0.021 to 0.058	0.034	-0.004 to 0.071	0.001	-0.038 to 0.041	0.002	-0.036 to 0.039
Interaction (main effect)	0.002	-0.060 to 0.063	0.015	-0.046 to 0.076	0.044	-0.013 to 0.100	0.026	-0.035 to 0.088	-0.015	-0.070 to 0.041
Interaction (interaction effect)	-0.022	-0.094 to 0.051	0.011	-0.060 to 0.082	-0.014	-0.083 to 0.054	-0.030	-0.103 to 0.042	0.027	-0.040 to 0.093

**Bold** indicates effect sizes (Cohen’s d) larger than ±0.2, *Italics* indicates statistical significance (p>0.05)

All models have been adjusted for gender, ethnicity (White—British; Any other White background; Chinese, Asian or Asian British; Mixed/Dual background; Any other ethnic group), the IMD 2010 score (rescaled least to most deprived 0–1) of the child’s home lower super output area and whether more than 20% of eligible pupils didn’t participate in the NCMP.

Outcome: Overwgt; overweight (body mass index standard deviation score ≥85^th^ percentile and >95^th^ percentile of the United Kingdom 1990 reference population[27]), Obese; obesity (body mass index standard deviation score ≥95% percentile of the United Kingdom 1990 reference population[27]).

None of the school-based contextual factors demonstrated consistent associations with pupil overweight or obesity status, with effect sizes varying from model to model and year to year, all the effect sizes were less than |0.35|. Of the school-based contextual factors, *Deprivation* was most often meaningfully (d>|0.2|) associated with pupil weight status (increased odds of obesity). *Location* and *Prioritisation of physical activity* were never meaningfully associated with pupil overweight or obesity status. *Resource* was only once meaningfully associated with reduced odds of overweight. The expectation that school context would be more consistently and meaningfully associated with Year 6 pupil weight status than Reception pupil was not evident in the results. When the interactions between year group and factor were tested in the three-level models, the direction of the association of the main (Reception) and interaction (Year 6) effects were almost always in opposition (Table 5). For example in the 2007/08 *Deprivation* three-level interaction model of obese status the effect size in Reception (the main effect) is 0.235, while in Year 6 it is 0.124 (= 0.235–0.111), with the interaction effect flattening the main effect (Table 5). This indicates that any associations with contextual factors were stronger during Reception than during Year 6. The full details of the models can be found in S3 File alongside the sensitivity analysis undertaken using overweight and obesity categorised according to the International Obesity Task Force classification [51] and continuous BMI-SDS as outcomes. The sensitivity analysis supports the lack of consistent and meaningfully significant associations.

S4 File contains all the calculated caterpillar plots, which were smoother than those calculated in our previous study [20], demonstrating the effect of the fitted variables. The caterpillar plots differed very little between factors, which is coherent with the minimal impact of the factors demonstrated in Table 5. For brevity in Table 6 the plots have been categorised by the amount of uncertainty in each school or year group difference residual; little to none (−), small (~), medium (≈), large (≋). There were two slight variants of these four plot types labelled positive skew (+) and ‘outlier’ (°), although no true outliers were identified and none were consistent. Generally there was more variation in residuals from models of obesity than overweight, and the positive skew variant only occurred from obesity models, which likely reflects the increasing prevalence of obesity during primary school. The caterpillar plots confirm the finding from the ICCs (Table 4) that in the three-level models there is no variation between schools. Introducing the year group interaction revealed a small quantity of between school variation in some models, but the variation between year groups within schools is more marked. The caterpillar plots support our previous finding that there is no systematic differential school impact on pupil weight status, as well as indicating that there are unlikely to be unmeasured features of groups of schools that influence weight status [20]. Any remaining systematic common school impact is confounded with age-period-cohort effects which we cannot assess.

**Table 6:** Caterpillar plot types and explained variation.

	2006/07		2007/08		2008/09		2009/10		2010/11	
	Caterpillar plot type	Variation explained	Caterpillar plot type	Variation explained	Caterpillar plot type	Variation explained	Caterpillar plot type	Variation explained	Caterpillar plot type	Variation explained
**Factor 1—Deprivation**										
*Overweight*										
Variation between schools										
Reception two-level	≈	7.19%	−	100.00%	≈°	2.45%	~	8.22%	~	29.61%
Year 6 two-level	~	96.52%	~	44.94%	≈	5.29%	~	25.34%	−	[0%]
Three-level	−	76.02%	−	99.98%	−	99.58%	~	12.17%	~	[0%]
Interaction	−	100.00%	~	[0%]	~	[0%]	~	[0%]	~	[0%]
Variation between year groups										
Three-level	≈	12.43%	~	12.69%	≋	[0%]	≈	1.16%	−	99.69%
Interaction	≈	32.05%	−	100.00%	≋	6.99%	~	34.40%	−	99.96%
*Obese*										
Variation between schools										
Reception two-level	−	[0%]	~	54.30%	~°	14.88%	≈°	26.08%	~	10.32%
Year 6 two-level	~	45.04%	~	8.60%	~	24.33%	~	11.48%	−	97.06%
Three-level	−	[0%]	−	[0%]	−	99.52%	−	99.90%	−	75.77%
Interaction	~	[0%]	~°	[0%]	~	[0%]	~°	[0%]	~	[0%]
Variation between year groups										
Three-level	≈	5.64%	≈+	6.72%	≈+	12.95%	≋+	10.79%	≈+	16.65%
Interaction	−	100.00%	~	89.06%	~	67.85%	~	72.34%	−	100.00%
**Factor 2 —Location**										
*Overweight*										
Variation between schools										
Reception two-level	≈	4.82%	−	100.00%	≈°	3.21%	~	22.06%	~	29.93%
Year 6 two-level	~	95.25%	~	64.77%	≈	4.30%	~	28.63%	−	100.00%
Three-level	−	100.00%	~	91.41%	−	99.98%	−	99.53%	~	[0%]
Interaction	−	99.39%	~	[0%]	~	[0%]	~	[0%]	~	[0%]
Variation between year groups										
Three-level	≈	13.28%	~	13.78%	≋	[0%]	≈	2.15%	−	99.81%
Interaction	≈	33.55%	−	100.00%	≋	5.15%	~	36.22%	−	99.83%
*Obese*										
Variation between schools										
Reception two-level	−	[0%]	~	49.77%	~°	23.58%	≈°	24.05%	~	1.98%
Year 6 two-level	~	54.12%	~	12.38%	~	19.93%	~	11.23%	−	87.83%
Three-level	−	[0%]	−	[0%]	−	99.83%	−	96.15%	−	[0%]
Interaction	~	[0%]	~°	[0%]	~	[0%]	~°	[0%]	~	[0%]
Variation between year groups										
Three-level	≈	7.21%	≈+	6.92%	≈+	12.08%	≋+	10.80%	≈+	13.10%
Interaction	−	100.00%	~	89.62%	~	70.32%	~	72.21%	−	100.00%
**Factor 3—Resource**										
*Overweight*										
Variation between schools										
Reception two-level	≈	15.26%	−	100.00%	≈°	1.48%	~	6.57%	~	4.93%
Year 6 two-level	−	100.00%	~	45.15%	≈	[0%]	~	10.87%	−	[0%]
Three-level	−	100.00%	−	99.99%	−	99.99%	~	[0%]	~	[0%]
Interaction	−	99.86%	~	[0%]	~	28.96%	~	[0%]	~	[0%]
Variation between year groups										
Three-level	≈	11.12%	~	4.13%	≋	[0%]	~	[0%]	−	[0%]
Interaction	~	43.54%	−	100.00%	≋	[0%]	~	25.53%	−	99.98%
*Obese*										
Variation between schools										
Reception two-level	−	[0%]	~	47.78%	~°	5.96%	≈°	23.30%	~	2.14%
Year 6 two-level	~	79.89%	~	2.33%	~	18.42%	~	12.77%	~	86.24%
Three-level	−	[0%]	−	[0%]	−	99.99%	−	99.27%	−	100.00%
Interaction	~	[0%]	~°	[0%]	~	[0%]	~°	[0%]	~	[0%]
Variation between year groups										
Three-level	≈	8.03%	≈+	4.85%	≈+	8.44%	≋+	10.94%	≈+	12.98%
Interaction	−	100.00%	~	89.30%	~	69.08%	~	70.24%	−	100.00%
**Factor 4—Prioritisation of physical activity**										
*Overweight*										
Variation between schools										
Reception two-level	≈	5.94%	−	100.00%	≈°	1.93%	~	3.74%	~	19.32%
Year 6 two-level	~	98.93%	~	50.83%	≈	4.44%	~	19.73%	−	[0%]
Three-level	−	96.64%	~	83.96%	−	100.00%	~	19.26%	~	4.54%
Interaction	−	100.00%	~	[0%]	−	81.04%	~	[0%]	~	11.12%
Variation between year groups										
Three-level	≈	13.57%	~	11.69%	≋	[0%]	~	0.42%	−	91.07%
Interaction	~	35.05%	~	96.21%	≈	0.63%	~	24.78%	−	82.87%
*Obese*										
Variation between schools										
Reception two-level	−	[0%]	~	47.08%	~°	7.91%	≈°	24.06%	~	2.74%
Year 6 two-level	~	62.77%	~	8.94%	~	17.98%	~	18.31%	~	84.03%
Three-level	−	[0%]	−	[0%]	−	[0%]	−	98.10%	−	[0%]
Interaction	~	[0%]	~°	[0%]	~	[0%]	~°	[0%]	~	[0%]
Variation between year groups										
Three-level	≈	8.18%	≈+	6.24%	≈+	8.75%	≋+	10.72%	≈+	13.02%
Interaction	−	100.00%	~	89.24%	~	68.67%	~	71.27%	−	100.00%

[0%] indicates that the variance increased slightly from the null to fitted model meaning that the variation explained is effectively null.

Table 6 also lists the amount of variation explained comparing the fitted models with the null models (Table 4), which alongside the ICCs (Table 4) demonstrate that the variation components are unstable, varying between year and model structure. Consistent with the findings of Miyazaki and Stack [52], the percentages of variation explained was sometimes negative as a result of chance fluctuations. This suggests an underlying complex variation structure, invalidating the use of simple methods to explore school impact on pupil weight status.

## Discussion

The overarching objective of this study has been to explore the potential for measurable characteristics of the school context to have an impact on pupil weight status. Based on previous research, data were collected on a variety of school variables reflecting the contextual, compositional and collective attributes of the schools [24,34–40]. Exploratory factor analysis identified four school-based contextual factors from the school variables representing; *Deprivation*, *Location*, *Resource* and *Prioritisation of physical activity*. Given that the school context is complex, drawing conclusions from studying individual school variables in isolation is inappropriate, whereas these school-based contextual factors reflect the multiple inter-related components of the school context. However, no meaningfully significant and consistent associations between any of the derived school-based contextual factors and pupil overweight or obesity status were identified. The *Location* and *Prioritisation of physical activity* factors were consistently not meaningfully associated with pupil weight status, while *Deprivation* was most consistently associated with obesity. Three additional findings from the second analytical phase provide further insights into the impact of school context on pupil weight status:

- The relative and absolute proportion of variation in pupil weight status attributable to differences between schools decreased from Reception to Year 6.
- When differences between year groups within schools had been accounted for, there was little to no variation in pupil weight status attributable to differences between schools.
- When exploring the interactions between year group and school-based contextual factors, the associations weakened between Reception and Year 6.

Consequently, through novel, theory driven, analysis it has not been possible to identify common and measurable features of the school context that have an obesogenic impact.

This study has made extensive use of routinely collected data. The use of cross-sectional data meant that the consideration of causal links and age-period-cohort effects relevant to this study was more difficult; at present an equally large longitudinal dataset is not available. Recent changes to the NCMP mean that from around 2020 pupil Reception and Year 6 data will be linkable enabling more refined analyses [53]. Using routinely collected school data, has meant that only the measureable objective aspects of the school context have been captured, a particular weakness being the inability to include dietary variables. We note that from September 2015, as part of the School Food Plan, school inspections will include inspections of the school food and food environment [54]. The subjective school context may not be represented, although some of the derived school-based contextual factors reflected less tangible aspects of the school context (e.g. prioritisation of physical activity). But, using routinely collected data and thorough analyses have permitted the undertaking of a cost-efficient large-scale study to test and subsequently contest a common and important assumption that schools are contributing to the obesity epidemic. In calculating the compositional variables for Year 6 pupils, we made the assumption that each child remained at the same school for the entirety of their primary education and that the influence of each year was equal. These simplifying assumptions were necessary because of the lack of information on which school each Year 6 pupil had attended in previous years and are likely to attenuate the effects of the contextual variables. However, in the absence of any significant findings no analysis of the sensitivity of this assumption has been undertaken. In order to overcome some of the issues with multiple testing the results have been presented as effect sizes (Cohen’s d values) which also placed the emphasis on meaningful, rather than just statistically significant results. We note that the Kaiser-Meyer-Olkin measure of sampling adequacy was low but considered adequate to proceed within this study.

Being conducted in Devon, England, precluded this study from exploring possible ethnic differences, which have been the focus of previous studies in this area [55,56] and limited the scope for examining associations with socioeconomic status (Table 1). However, our finding that deprivation was more markedly associated with pupil obese than overweight status is consistent with Conrad and Capewell [57]. Previous studies have reported school-based contextual variables which they had found to be associated with pupil weight status, diet or physical activity [24,34–38]. In this study we strove to examine multiple aspects of the school context simultaneously, as advocated by Bonell et al. [5], in the form of school-based contextual factors, which may explain the absence of previous associations among the findings. Although the school ICCs observed within this study were small, they are consistent with previously reported values, as was the finding that school ICCs were larger among Reception rather than Year 6 pupils [26,56,58,59]. Pallan et al. [56] acknowledge that this finding is counterintuitive, but posit two explanations; that the early primary school years are more conducive to physical activity whereas the later years focus more on academic attainment, and/ or that across the school years the influence of non-school environments swamp the school environment. Our results are consistent with their second explanation, but when considering their first explanation we argue that nine months or less exposure (for Reception pupils) is unlikely to impact significantly on weight status in comparison with five years of exposure to the home environment. Moreover, unlike previous studies the additional year group level in the three-level models made it possible to examine both *between* and *within* school effects. The finding that with the additional level the school ICCs in the three-level models effectively becomes zero indicates that there are no observed differences *between* schools, only differences between year groups *within* schools. Subsequently, the observed doubling in the prevalence of obesity during primary education does not appear to differ significantly between schools [13–17].

It has been a commonly held assumption that the school environment has been contributing to the obesity epidemic through prolonged sedentary behaviour, less healthy foods and insufficient physical activity [5–7]. However, within this study no evidence of such an effect has been detected. Schools have been targeted as they offer a structured environment within which the majority of children can be reached, but as Downey et al. [60] emphasise, even during the half of the year that children attend school the average child spends more than 60% of their waking time outside school. Studies with repeated measures of pupil weight status have found that pupil weight status increases more markedly during the school holidays than during term time [61–64]. Within the education literature this phenomenon is observed in regards to educational attainment and known as the summer learning gap [60,65–67]. Specifically, the evidence demonstrates that children tend to learn at the same rate during term time, but during the summer holiday the drop-off in learning increases with deprivation to such an extent that the summer learning gap is considered to be responsible for the socioeconomic inequalities in educational attainment, with schools considered the ‘great equaliser’ [59,65–67]. Gershenson [68] studied the differences in summer time-use by socioeconomic status and found that television viewing was the time-use most related to socioeconomic status. Given that sedentary behaviour is associated with weight status, it seems highly plausible that this impacts on weight status as well as educational attainment [9,24]. Our finding that the impact of school context seemed to lessen from Reception to Year 6 would be consistent with increasing disparities due to either increasing biological variation, or unequal opportunities outside the school, or a combination thereof. Furthermore if, as in the findings from those studies with repeated measures, growth in weight status slows or even decreases during term time, the school context may be stabilising pupil weight status and functioning as the great equaliser, holding-back weight gain [61–64]. The flattening of the association between school-based contextual factor and pupil weight status identified when exploring the interaction with year group may reflect this phenomenon. We are not aware of any longitudinal study with sufficiently frequent (at the beginning and end of each school year or term) anthropometric measures to test this hypothesis. However, Anderson et al. [69] using the endogeneity in school starting age found that pupils starting school earlier (as the youngest in the class) had better weight outcomes than those starting later (as the oldest in the class) supporting this hypothesis. Therefore, it would appear that non-school time and school holidays in particular are a promising focus for obesity prevention interventions, especially as such interventions have the potential to influence the adult, as well as the child population [70].

## Supporting Information

S1 File School characteristics. (PDF)

S2 File Correlation matrix and factor stability. (PDF)

S3 File Detailed results and sensitivity analysis. (PDF)

S4 File Caterpillar plots. (PDF)

S1 STROBE Checklist (PDF)
