# Supplementary material for: Exploring the Potential of a School Impact on Pupil Weight Status: Exploratory Factor Analysis and Repeat Cross-Sectional Study of the National Child Measurement Programme
Source: PLoS One. 2015 Dec 23;10(12):e0145128. doi: 10.1371/journal.pone.0145128 (PMC4699206; doi:10.1371/journal.pone.0145128)
Supplement: S1 File — (PDF) [file pone.0145128.s001.pdf]

Supporting file 1 to Williams, A.J., Wyatt, K.M., Williams, C.A., Logan, S., and Henley, W.E. Exploring the potential of a school impact on pupil weight status: exploratory factor analysis and repeat cross-sectional study of the National Child Measurement Programme.

S1 file - School characteristics

Cluster	Variable		Summary statistics[a]	Missing data schools/individuals	Source
Demographic	Capacity	≤100 pupils	103 (33.99%)	3 / 60	School Census - DCC
		101-200 pupils	91 (30.03%)		
		201-400 pupils	85 (28.05%)		
		>400 pupils	24 (7.92%)		
	Gender mix (percentage female)		48.29±5.13%	1 / 74	School Census - DCC
	Ethnic mix (percentage not White – British)	No ethnic mix	16 (5.23%)	0 / 14	School Census - DCC
		>0%-5% ethnic mix	198 (64.71%)		
		5%-10% ethnic mix	75 (24.51%)		
		>10% ethnic mix	17 (5.56%)		
Socioeconomic status	Index of Multiple Deprivation (IMD) 2010[b]		0.19 (0.14, 0.24)	0 / 0	DfCLG
	Income Deprivation Affecting Children Index (IDACI) 2010[b]		0.11 (0.08, 0.16)	0 / 0	DfCLG
	Child Wellbeing Index (CWI) 2009[b]		0.21 (0.16, 0.26)	0 / 0	DfCLG
	Percentage eligible for free school meals		7.69% (4.59%, 12.70%)	1 / 74	School Census - DCC
	Mean of pupil IMD 2010[b]		0.19 (0.15, 0.22)	0 / 151	Calculated
	Mean of pupil IDACI 2010[b]		0.12 (0.10, 0.15)	0 / 151	Calculated
	Mean of pupil CWI 2009[b]		0.21 (0.17, 0.26)	0 / 151	Calculated
NCMP[d]	≥20% of eligible pupils not participating		45 (14.75%)	1 / 182	NCMP - NHS Devon
Built environment	Location	Hamlet and Isolated Dwelling -sparse	16 (5.23%)	0 / 0	EduBase 2 - DfE
		Hamlet and Isolated Dwelling -less sparse	27 (8.82%)		
		Village - sparse	39 (12.75%)		
		Village - less sparse	82 (26.80%)		
		Town and Fringe - sparse	8 (2.61%)		
		Town and Fringe - less sparse	57 (18.63%)		
		Urban > 10k - sparse	1 (0.33%)		
		Urban > 10k -less sparse	76 (24.84%)		
	Coastal		49 (16.01%)	0 / 0	Catchment area map
	Building age[d]	Pre-Victorian	6 (2.80%)	92 / 20,435	School websites
		Victorian	121 (56.54%)		
		1902-1949	16 (7.48%)		
		1950-1989	32 (14.95%)		
		1990-Present	39 (18.22%)		
	Multiple sites		62 (20.81%)	8 / 1,475	NPS - DCC
	Size of grass play area per pupil	0 m^2/pupil	37 (12.42%)	8 / 1,475	NPS - DCC
		>0-≤50 m^2/pupil	180 (60.40%)		

Built environment		>50-≤100 m^2/pupil	63 (21.14%)		
		>100 m^2/pupil	18 (6.04%)		
	Size of hard surface play area per pupil	0 m^2/pupil	21 (7.05%)	8 / 1,475	NPS - DCC
		>0-≤10 m^2/pupil	211 (70.81%)		
		>10-≤20 m^2/pupil	63 (21.14%)		
		>20 m^2/pupil	3 (1.01%)		
	Total play area per pupil	0 m^2/pupil	13 (4.36%)	8 / 1,475	NPS - DCC
		>0-≤50 m^2/pupil	184 (61.74%)		
		>50-≤100 m^2/pupil	80 (26.85%)		
		>100 m^2/pupil	21 (7.05%)		
	Total area per pupil	>0-≤100 m^2/pupil	244 (81.06%)	5 / 262	NPS - DCC
		>100 m^2/pupil	57 (18.94%)		
Physical activity	Catchment area[d]	>0-≤1250 hectares	104 (35.86%)	16 / 4,961	DCC
		>1250-≤2500 hectares	91 (31.38%)		
		>2500-≤5000 hectares	72 (24.83%)		
		>5000 hectares	23 (7.93%)		
	Travel plan[d]		306 (100.00%)[c]	0 / 0	School Census - DCC
	Percentage using active transport	≤20%	58 (19.02%)	1 / 145	School Census - DCC
		>20% to ≤40%	77 (25.25%)		
		>40% to ≤60%	72 (23.61%)		
		>60% to ≤80%	83 (27.21%)		
		>80%	15 (4.92%)		
	Physical Education, Daily Physical Activity and School Sports (PEDPASS) award	None	253 (82.68%)	0 / 0	DCC
		Pass	51 (16.67%)		
		Commendation	2 (0.65%)		
	Active Lifestyle award	None	256 (83.66%)	0 / 0	DCC
		Bronze	20 (6.54%)		
		Silver	17 (5.56%)		
		Gold	13 (4.25%)		
Diet and ethos	Governance	Community	190 (62.09%)	0 / 0	School Census - DCC
		Voluntary aided	55 (17.97%)		
		Voluntary controlled	61 (19.93%)		
	Religious denomination[d]	None	189 (61.76%)	0 / 0	School Census - DCC
		Church of England	108 (35.29%)		
		Roman Catholic	9 (2.94%)		
	Subscription	Under capacity	161 (53.31%)	4 / 134	School Census - DCC
		Around capacity	41 (13.58%)		
		Overcapacity	100 (33.11%)		
	Proportion for whom English is an	None	103 (33.66%)	0 / 14	School Census - DCC

	Additional Language	Low (≤5%)	187 (61.11%)		
		High (>5%)	16 (5.23%)		
Diet and ethos	Percentage Special Educational Needs (SEN)	<30% SEN	287 (94.10%)	1 / 161	School Census - DCC
		≥30% SEN	18 (5.90%)		
	Key stage 2 National Curriculum Test (SATs) percentage level 4+ in both English and/or Maths[d]	0-60% of Year 6	24 (11.01%)	88 / 10,292	DfE
		61-80% of Year 6	98 (44.95%)		
		81-100% of Year 6	96 (44.04%)		
	Devon Healthy Schools achievement time	No award	15 (4.90%)	0 / 0	NHS Devon
		Early (within 1 year)	46 (15.03%)		
		Mode (within 2-3 years)	206 (67.32%)		
		Late (more than 4 years)	39 (12.75%)		

CWI, Child Wellbeing Index; DCC, Devon County Council; DfCLG, Department for Communities and Local Government; DfE, Department for Education; IDACI, Income Deprivation Affecting Children Index; IMD, Index of Multiple Deprivation; NCMP, National Child Measurement Programme; NHS Devon, primary care trust; NPS; Norfolk Propoery Services who survey properties for DCC; PEDPASS, Physical Education, Daily Physical Activity and School Sports.
[a]Summary statistics are numbers (percentages (%)) for categorical variables, means±standard deviations for data which is approximately normally distributed, and median (quartile 1, quartile 4 (interquartile range)).
[b]Nationally rescaled
[c]From 2010/11 all schools were required to have a travel plan and conseuqntly this variable was excluded from the exploratory factor analysis, but used to interpret the meaning of the school-based contextual factors: 189/296 (63.85%) of schools in 2006/07 had a travel plan, 237/300 (79.00%) of schools in 2007/08 had a travel plan, 280/303 (92.41%) of schools in 2008/09 had a travel plan, 288/302 (95.36%) of schools in 2009/10 had a travel plan.
[d]Excluded from exploratory factor analysis, see the main text for the explaination.
