# Supplementary material for: Exploring the Potential of a School Impact on Pupil Weight Status: Exploratory Factor Analysis and Repeat Cross-Sectional Study of the National Child Measurement Programme
Source: PLoS One. 2015 Dec 23;10(12):e0145128. doi: 10.1371/journal.pone.0145128 (PMC4699206; doi:10.1371/journal.pone.0145128)
Supplement: S2 File — (PDF) [file pone.0145128.s002.pdf]

Supporting file 2 to Williams, A.J., Wyatt, K.M., Williams, C.A., Logan, S., and Henley, W.E. Exploring the potential of a school impact on pupil weight status: exploratory factor analysis and repeat cross-sectional study of the National Child Measurement Programme

S2 file - Correlation matrix and factor stability

Correlation matrix used to calculate factors (polychoric)

Variable	School pupil capacity	Proportion female	Proportion not White – British	School location IMD 2010 (rescaled)	School location IDACI 2010 (rescaled)	School location CWI 2009 (rescaled)	Proportion eligible for FSM	School mean IMD 2010 (rescaled)	School mean IDACI 2010 (rescaled)	School mean CWI 2009 (rescaled)	School location (from rural to urban)	Coastal catchment area	School has multiple sites	Grass play area (m^2/pupil)	Hard surface play area (m^2/pupil)	Total site area (m^2/pupil)	Proportion of pupils walking or cycling	School PEDPASS award	School Active Lifestyle award	School governance	School over- or under-subscribed	Proportion of pupils for whom EAL	Proportion with SEN	Devon Healthy School Award (timing)
School pupil capacity	1.000																							
Proportion female	0.028	1.000																						
Proportion not White – British	0.127	0.052	1.000																					
School location IMD 2010 (rescaled)	0.022	-0.024	0.011	1.000																				
School location IDACI 2010 (rescaled)	0.267	0.000	0.044	0.772	1.000																			
School location CWI 2009 (rescaled)	-0.029	-0.045	-0.017	0.784	0.687	1.000																		
Proportion eligible for FSM	0.259	-0.020	0.064	0.572	0.633	0.565	1.000																	
School mean IMD 2010 (rescaled)	0.033	-0.015	0.097	0.784	0.618	0.692	0.615	1.000																
School mean IDACI 2010 (rescaled)	0.303	-0.003	0.128	0.612	0.789	0.577	0.706	0.792	1.000															
School mean CWI 2009 (rescaled)	0.019	-0.030	0.092	0.607	0.515	0.815	0.579	0.836	0.699	1.000														
School location (from rural to urban)	0.817	0.048	0.282	0.100	0.428	0.040	0.464	0.117	0.463	0.110	1.000													
Coastal catchment area	0.135	0.046	-0.001	0.123	0.217	0.090	0.044	0.076	0.189	-0.043	0.196	1.000												
School has multiple sites	-0.321	0.032	0.016	-0.106	-0.225	-0.196	-0.158	-0.153	-0.238	-0.240	-0.242	0.025	1.000											
Grass play area (m^2/pupil)	-0.249	0.006	-0.121	-0.039	-0.068	0.010	-0.086	-0.023	-0.117	-0.046	-0.210	-0.067	0.401	1.000										
Hard surface play area (m^2/pupil)	-0.342	0.031	-0.054	0.168	0.069	0.142	-0.004	0.138	-0.043	0.106	-0.329	-0.103	0.084	0.279	1.000									
Total site area (m^2/pupil)	-0.534	0.032	-0.137	-0.013	-0.214	0.091	-0.139	0.005	-0.271	0.066	-0.395	-0.139	0.408	0.682	0.198	1.000								
Proportion of pupils walking or cycling	0.570	0.024	0.131	0.207	0.434	0.152	0.519	0.236	0.448	0.228	0.681	0.034	-0.189	-0.181	-0.116	-0.308	1.000							
School PEDPASS award	0.046	0.155	0.070	-0.075	0.025	-0.028	-0.062	-0.036	0.030	0.006	0.057	0.133	-0.023	-0.057	-0.127	-0.332	0.009	1.000						
School Active Lifestyle award	0.033	0.103	0.007	-0.039	0.052	-0.097	-0.087	-0.052	0.077	-0.046	0.049	0.190	0.131	0.028	-0.011	-0.126	-0.023	0.466	1.000					
School governance	-0.243	0.007	0.116	0.003	-0.030	-0.094	-0.021	-0.057	-0.051	-0.135	-0.054	0.186	0.302	-0.033	0.000	0.154	-0.160	0.117	0.038	1.000				
School over- or under-subscribed	-0.038	0.062	-0.025	-0.051	-0.036	-0.138	-0.167	0.030	0.054	-0.091	0.105	0.141	0.003	-0.059	-0.126	-0.102	-0.070	0.191	0.228	0.028	1.000			
Proportion of pupils for whom EAL	0.599	0.105	0.538	0.103	0.301	0.051	0.312	0.190	0.418	0.125	0.687	0.123	-0.089	-0.083	-0.190	-0.292	0.392	-0.048	0.061	-0.025	0.074	1.000		
Proportion with SEN	0.018	-0.089	0.062	0.485	0.516	0.478	0.427	0.444	0.487	0.466	0.284	0.073	-0.136	0.152	0.230	0.175	0.368	-0.087	-0.030	0.005	-0.273	0.030	1.000	
Devon Healthy School Award (timing)	0.012	0.037	-0.086	-0.127	-0.075	-0.111	0.021	-0.178	-0.096	-0.111	0.059	-0.132	0.072	0.077	-0.093	0.149	-0.038	-0.086	0.099	-0.025	0.108	-0.032	-0.224	1.000

Checking factor stability

In order to assess whether schools factor scores are fairly stable I have selected three schools from each of Factor E and tested whether the score changed across the five years

Factor 1 - Deprivation

School		2006/07	2007/08	2008/09	2009/10	2010/11
Low score school	Reception	0.2174	0.2368	0.2160	0.2198	0.0631
	Year 6	0.2173	0.2231	0.2165	0.2206	0.1765
Middle score school	Reception	0.7952	0.8005	0.7966	0.8014	0.7472
	Year 6	0.7961	0.8008	0.7968	0.8016	0.8010
High score school	Reception	1.4668	1.4847	1.4557	1.4407	1.4454
	Year 6	1.4672	1.4847	1.4951	1.4913	1.4962

Factor 2 - Location

School		2006/07	2007/08	2008/09	2009/10	2010/11
Low score school	Reception	1.0716	1.1753	1.1167	1.1665	0.8504
	Year 6	0.9699	0.9703	0.9648	1.1211	1.1097
Middle score school	Reception	5.9509	5.9877	5.9454	5.9462	5.9470
	Year 6	5.9492	5.9882	5.9475	5.9478	5.9477
High score school	Reception	7.7217	8.0952	7.9928	8.1423	8.1489
	Year 6	7.6679	7.7199	7.7905	7.9426	7.9461

Factor 3 - Resource

School		2006/07	2007/08	2008/09	2009/10	2010/11
Low score school	Reception	0.8935	0.8914	0.8920	0.9233	Non particpant
	Year 6	1.0777	0.9825	0.9836	0.9815	Non particpant
Middle score school	Reception	2.0203	2.0199	2.0217	2.0186	1.8684
	Year 6	2.0189	2.0189	2.0217	2.0185	2.0182
High score school	Reception	3.3926	3.3921	3.3907	3.5728	3.7200
	Year 6	3.5099	3.5088	3.3905	3.3884	3.5965

Factor 4 - Prioritisation of physical activity

School		2006/07	2007/08	2008/09	2009/10	2010/11
Low score school	Reception	0.1365	0.1264	0.1579	0.1525	0.3275
	Year 6	0.1354	0.1302	0.1468	0.1336	0.1539
Middle score school	Reception	3.2289	3.2344	3.2317	3.2353	3.4116
	Year 6	3.3190	3.3283	3.3265	3.3282	3.3242
High score school	Reception	4.9079	4.7904	4.7445	4.5657	4.5648
	Year 6	4.8897	4.7856	4.7248	4.7412	4.6466
