# Supplementary material for: Exploring the Potential of a School Impact on Pupil Weight Status: Exploratory Factor Analysis and Repeat Cross-Sectional Study of the National Child Measurement Programme
Source: PLoS One. 2015 Dec 23;10(12):e0145128. doi: 10.1371/journal.pone.0145128 (PMC4699206; doi:10.1371/journal.pone.0145128)
Supplement: S3 File — (PDF) [file pone.0145128.s003.pdf]

Supporting file 3 to Williams, A.J., Wyatt, K.M., Williams, C.A., Logan, S., and Henley, W.E. Exploring the potential of a school impact on pupil weight status: exploratory factor analysis and repeat cross-sectional study of the National Child Measurement Programme.

S3 file - Detailed results and sensitivity analysis

Factor 1 - Deprviation

Red shading indicates significance (p<0.05), Green shading indicates modles which had to be estimated in R

Overweight (UK90)

**Single level**		**2006/07**			**2007/08**			**2008/09**			**2009/10**			**2010/11**		
n		8747			10061			10149			10356			9604		
Log likelihood	**Null**	-3723.27			-4273.38			-4660.99			-4699.66			-4334.16		
	**Individual**	-3717.66			-4267.11			-4657.93			-4694.99			-4326.89		
	**Factor**	-3717.66			-4265.23			-4657.76			-4694.62			-4326.81		
**Individual**		**Odds Ratio**	**LCI**	**UCI**	**Odds Ratio**	**LCI**	**UCI**	**Odds Ratio**	**LCI**	**UCI**	**Odds Ratio**	**LCI**	**UCI**	**Odds Ratio**	**LCI**	**UCI**
Gender	Male	(ref)	(ref)	(ref)	(ref)	(ref)	(ref)	(ref)	(ref)	(ref)	(ref)	(ref)	(ref)	(ref)	(ref)	(ref)
	Female	0.880	0.783	0.990	0.897	0.804	1.001	0.933	0.842	1.035	0.920	0.830	1.020	0.856	0.769	0.953
Ethnicity	White - British	(ref)	(ref)	(ref)	(ref)	(ref)	(ref)	(ref)	(ref)	(ref)	(ref)	(ref)	(ref)	(ref)	(ref)	(ref)
	Any other White background	0.935	0.610	1.435	0.813	0.559	1.184	0.947	0.675	1.329	1.086	0.780	1.512	1.191	0.876	1.621
	Asian or Asian British	0.542	0.194	1.518	0.895	0.471	1.701	0.776	0.348	1.728	0.993	0.484	2.041	0.775	0.383	1.571
	Mixed/Dual background	0.998	0.563	1.767	0.733	0.441	1.220	0.816	0.523	1.275	1.011	0.688	1.484	0.868	0.555	1.357
	Any other ethnic group	1.197	0.556	2.577	0.908	0.382	2.156	0.571	0.260	1.254	0.871	0.470	1.614	1.102	0.616	1.970
SES	IMD 2010	1.873	1.055	3.324	1.888	1.095	3.256	1.163	0.698	1.936	1.719	1.033	2.858	1.356	0.792	2.320
NCMP participation (non-participation >0.2)		0.964	0.814	1.142	1.079	0.923	1.262	1.076	0.889	1.302	1.126	0.946	1.341	1.280	0.971	1.686
**Factor**		**Odds Ratio**	**LCI**	**UCI**	**Odds Ratio**	**LCI**	**UCI**	**Odds Ratio**	**LCI**	**UCI**	**Odds Ratio**	**LCI**	**UCI**	**Odds Ratio**	**LCI**	**UCI**
Gender	Male	(ref)	(ref)	(ref)	(ref)	(ref)	(ref)	(ref)	(ref)	(ref)	(ref)	(ref)	(ref)	(ref)	(ref)	(ref)
	Female	0.880	0.783	0.990	0.897	0.804	1.001	0.933	0.842	1.035	0.921	0.830	1.021	0.856	0.769	0.953
Ethnicity	White - British	(ref)	(ref)	(ref)	(ref)	(ref)	(ref)	(ref)	(ref)	(ref)	(ref)	(ref)	(ref)	(ref)	(ref)	(ref)
	Any other White background	0.935	0.610	1.435	0.817	0.561	1.189	0.947	0.675	1.330	1.090	0.783	1.519	1.190	0.874	1.619
	Asian or Asian British	0.542	0.194	1.518	0.880	0.463	1.673	0.781	0.351	1.741	1.005	0.489	2.065	0.771	0.380	1.563
	Mixed/Dual background	0.998	0.563	1.768	0.735	0.442	1.223	0.816	0.523	1.274	1.014	0.691	1.490	0.867	0.554	1.355
	Any other ethnic group	1.197	0.555	2.579	0.890	0.375	2.115	0.575	0.262	1.263	0.878	0.474	1.626	1.097	0.613	1.962
SES	IMD 2010	1.875	1.030	3.412	1.576	0.889	2.794	1.224	0.715	2.096	1.853	1.083	3.173	1.305	0.741	2.300
NCMP participation (non-participation >0.2)		0.964	0.814	1.142	1.088	0.931	1.273	1.075	0.889	1.301	1.129	0.948	1.345	1.287	0.975	1.698
Factor 1		0.999	0.788	1.265	1.243	0.997	1.550	0.939	0.762	1.157	0.913	0.744	1.122	1.048	0.836	1.314

**Reception two-level**		**2006/07**			**2007/08**			**2008/09**			**2009/10**			**2010/11**		
n		4002			4769			4889			4908			4520		
Log likelihood	**Null**	-1621.95			-1944.48			-2250.55			-2162.02			-2008.25		
	**Individual**	-1619.64			-1932.20			-2246.54			-2158.56			-2003.00		
	**Factor**	-1619.52			-1930.93			-2246.43			-2158.47			-2002.67		
School variance	**Null**	0.084	0.024	0.286	0.012	4.22E-05	3.544	0.088	0.039	0.200	0.027	0.002	0.300	0.018	4.06E-04	0.777
	**Individual**	0.080	0.022	0.288	1.64E-08	0.00E+00	.	0.087	0.038	0.197	0.025	0.002	0.338	0.016	2.79E-04	0.961
	**Factor**	0.078	0.021	0.291	1.32E-12	0	.	0.086	0.038	0.197	0.025	0.002	0.334	0.012	6.16E-05	2.537
**Individual**		**Odds Ratio**	**LCI**	**UCI**	**Odds Ratio**	**LCI**	**UCI**	**Odds Ratio**	**LCI**	**UCI**	**Odds Ratio**	**LCI**	**UCI**	**Odds Ratio**	**LCI**	**UCI**
Gender	Male	(ref)	(ref)	(ref)	(ref)	(ref)	(ref)	(ref)	(ref)	(ref)	(ref)	(ref)	(ref)	(ref)	(ref)	(ref)
	Female	0.914	0.763	1.095	0.830	0.704	0.977	0.941	0.810	1.094	0.875	0.751	1.020	0.841	0.718	0.986
Ethnicity	White - British	(ref)	(ref)	(ref)	(ref)	(ref)	(ref)	(ref)	(ref)	(ref)	(ref)	(ref)	(ref)	(ref)	(ref)	(ref)
	Any other White background	0.709	0.383	1.310	0.639	0.378	1.078	0.801	0.486	1.322	1.365	0.885	2.105	1.147	0.758	1.735
	Asian or Asian British	0.913	0.264	3.154	0.406	0.125	1.321	0.373	0.086	1.610	0.563	0.170	1.867	0.671	0.260	1.735
	Mixed/Dual background	1.011	0.421	2.427	0.322	0.117	0.884	0.817	0.457	1.462	0.854	0.491	1.485	0.808	0.436	1.496
	Any other ethnic group	1.262	0.469	3.397	0.597	0.138	2.575	1.021	0.414	2.517	0.995	0.412	2.399	0.668	0.234	1.905
SES	IMD 2010	1.336	0.535	3.337	2.754	1.257	6.038	1.534	0.706	3.332	1.407	0.659	3.006	1.493	0.672	3.319
NCMP participation (non-participation >0.2)		0.819	0.604	1.112	0.841	0.639	1.107	1.412	0.945	2.109	0.937	0.727	1.207	1.724	0.927	3.208
**Factor**		**Odds Ratio**	**LCI**	**UCI**	**Odds Ratio**	**LCI**	**UCI**	**Odds Ratio**	**LCI**	**UCI**	**Odds Ratio**	**LCI**	**UCI**	**Odds Ratio**	**LCI**	**UCI**
Gender	Male	(ref)	(ref)	(ref)	(ref)	(ref)	(ref)	(ref)	(ref)	(ref)	(ref)	(ref)	(ref)	(ref)	(ref)	(ref)
	Female	0.914	0.763	1.095	0.831	0.705	0.979	0.941	0.810	1.093	0.875	0.751	1.020	0.841	0.718	0.986
Ethnicity	White - British	(ref)	(ref)	(ref)	(ref)	(ref)	(ref)	(ref)	(ref)	(ref)	(ref)	(ref)	(ref)	(ref)	(ref)	(ref)
	Any other White background	0.710	0.384	1.312	0.647	0.383	1.092	0.801	0.486	1.322	1.368	0.887	2.109	1.140	0.753	1.724
	Asian or Asian British	0.907	0.263	3.134	0.397	0.122	1.292	0.370	0.086	1.597	0.567	0.171	1.882	0.661	0.256	1.711
	Mixed/Dual background	1.002	0.417	2.407	0.323	0.118	0.888	0.818	0.457	1.462	0.856	0.492	1.488	0.808	0.436	1.495
	Any other ethnic group	1.247	0.463	3.358	0.582	0.135	2.513	1.015	0.412	2.503	1.004	0.416	2.425	0.656	0.230	1.871
SES	IMD 2010	1.266	0.496	3.233	2.162	0.937	4.987	1.450	0.645	3.259	1.481	0.666	3.292	1.338	0.579	3.092
NCMP participation (non-participation >0.2)		0.823	0.607	1.117	0.853	0.648	1.124	1.417	0.948	2.117	0.941	0.730	1.213	1.745	0.939	3.244
Factor 1		1.105	0.745	1.638	1.313	0.939	1.836	1.084	0.771	1.525	0.936	0.682	1.286	1.154	0.817	1.630

**Year 6 two-level**		**2006/07**			**2007/08**			**2008/09**			**2009/10**			**2010/11**		
n		4745			5292			5260			5448			5084		
Log likelihood	**Null**	-2095.61			-2325.40			-2404.51			-2534.54			-2325.19		
	**Individual**	-2088.90			-2322.30			-2400.36			-2526.67			-2321.69		
	**Factor**	-2088.67			-2321.61			-2399.28			-2526.59			-2321.66		
School variance	**Null**	0.019	0.001	0.468	0.016	3.19E-04	0.763	0.043	0.009	0.200	0.026	0.003	0.216	5.17E-16	0	.
	**Individual**	0.004	1.10E-08	1830.554	0.009	9.25E-06	7.967	0.040	0.008	0.207	0.020	0.001	0.288	7.71E-19	0	.
	**Factor**	0.001	3.54E-42	1.21E+35	0.009	9.56E-06	7.712	0.040	0.008	0.204	0.019	0.001	0.310	6.58E-16	0	.
**Individual**		**Odds Ratio**	**LCI**	**UCI**	**Odds Ratio**	**LCI**	**UCI**	**Odds Ratio**	**LCI**	**UCI**	**Odds Ratio**	**LCI**	**UCI**	**Odds Ratio**	**LCI**	**UCI**
Gender	Male	(ref)	(ref)	(ref)	(ref)	(ref)	(ref)	(ref)	(ref)	(ref)	(ref)	(ref)	(ref)	(ref)	(ref)	(ref)
	Female	0.851	0.729	0.994	0.951	0.821	1.102	0.926	0.802	1.070	0.957	0.832	1.102	0.869	0.751	1.006
Ethnicity	White - British	(ref)	(ref)	(ref)	(ref)	(ref)	(ref)	(ref)	(ref)	(ref)	(ref)	(ref)	(ref)	(ref)	(ref)	(ref)
	Any other White background	1.418	0.764	2.632	1.154	0.668	1.994	1.099	0.685	1.763	0.842	0.499	1.423	1.269	0.797	2.021
	Asian or Asian British	0.241	0.032	1.791	1.604	0.721	3.570	1.232	0.458	3.317	1.633	0.638	4.182	0.897	0.308	2.613
	Mixed/Dual background	1.001	0.467	2.144	1.233	0.670	2.268	0.755	0.371	1.537	1.210	0.702	2.085	0.958	0.499	1.839
	Any other ethnic group	1.113	0.318	3.891	1.198	0.403	3.558	0.159	0.022	1.167	0.781	0.327	1.866	1.497	0.735	3.051
SES	IMD 2010	2.488	1.141	5.426	1.398	0.646	3.023	0.865	0.406	1.845	2.322	1.121	4.810	1.247	0.596	2.610
NCMP participation (non-participation >0.2)		1.047	0.845	1.296	1.199	0.986	1.459	0.977	0.768	1.242	1.487	1.143	1.933	1.172	0.858	1.602
**Factor**		**Odds Ratio**	**LCI**	**UCI**	**Odds Ratio**	**LCI**	**UCI**	**Odds Ratio**	**LCI**	**UCI**	**Odds Ratio**	**LCI**	**UCI**	**Odds Ratio**	**LCI**	**UCI**
Gender	Male	(ref)	(ref)	(ref)	(ref)	(ref)	(ref)	(ref)	(ref)	(ref)	(ref)	(ref)	(ref)	(ref)	(ref)	(ref)
	Female	0.850	0.728	0.993	0.951	0.821	1.103	0.925	0.800	1.069	0.958	0.832	1.102	0.869	0.751	1.006
Ethnicity	White - British	(ref)	(ref)	(ref)	(ref)	(ref)	(ref)	(ref)	(ref)	(ref)	(ref)	(ref)	(ref)	(ref)	(ref)	(ref)
	Any other White background	1.413	0.762	2.621	1.151	0.666	1.988	1.103	0.688	1.770	0.844	0.500	1.426	1.269	0.797	2.021
	Asian or Asian British	0.241	0.032	1.793	1.587	0.713	3.534	1.255	0.466	3.379	1.645	0.642	4.215	0.900	0.309	2.624
	Mixed/Dual background	1.002	0.468	2.146	1.236	0.672	2.273	0.752	0.370	1.532	1.214	0.704	2.092	0.959	0.500	1.842
	Any other ethnic group	1.127	0.322	3.939	1.180	0.397	3.506	0.163	0.022	1.198	0.782	0.327	1.867	1.500	0.736	3.059
SES	IMD 2010	2.737	1.200	6.242	1.223	0.550	2.722	1.015	0.461	2.234	2.426	1.132	5.198	1.283	0.589	2.793
NCMP participation (non-participation >0.2)		1.048	0.847	1.296	1.204	0.990	1.465	0.976	0.768	1.241	1.485	1.142	1.930	1.167	0.852	1.599
Factor 1		0.892	0.645	1.233	1.195	0.886	1.612	0.792	0.580	1.082	0.944	0.705	1.264	0.966	0.713	1.309

**Three-level**		**2006/07**			**2007/08**			**2008/09**			**2009/10**			**2010/11**		
n		8747			10061			10149			10356			9604		
Log likelihood	**Null**	-3721.38			-4272.82			-4655.48			-4698.53			-4334.14		
	**Individual**	-3716.23			-4266.83			-4652.40			-4693.85			-4326.84		
	**Factor**	-3716.23			-4265.02			-4652.13			-4693.55			-4326.77		
	**Interaction**	-3712.56			-4261.68			-4650.85			-4691.19			-4326.10		
School variance	**Null**	2.31E-11	0	.	0.006	2.30E-06	16.533	0.001	7.92E-22	1.86E+15	0.003	3.68E-10	21371.7	0.003		
	**Individual**	5.24E-18	0	.	0.001	2.70E-24	3.73E+17	9.97E-09	0	.	0.003	1.66E-09	5768.52	0.005	8.60E-06	2.605
	**Factor**	5.54E-12	0	.	1.52E-06	0	.	5.05E-06	0	.	0.002	3.71E-11	163765	0.004	3.55E-06	5.007
	**Interaction**	9.85E-20	0	.	0.007	6.2E-05	0.797	0.004	1.21E-08	1307.921	0.007	0.0000206	2.712	0.004	3.43E-06	5.156
Year group varia	**Null**	0.048	0.015	0.154	0.017	3.49E-04	0.813	0.066	0.023	0.190	0.026	0.002	0.304	9.93E-12		
	**Individual**	0.042	0.012	0.156	0.016	2.63E-04	0.943	0.067	0.033	0.138	0.026	0.002	0.305	7.28E-14	0	.
	**Factor**	0.042	0.012	0.156	0.015	0.001	0.333	0.068	0.033	0.141	0.026	0.002	0.305	3.09E-14	0	.
	**Interaction**	0.033	0.006	0.170	3.68E-07	0	.	0.061	0.020	0.190	0.017	4.64E-04	0.648	4.43E-15	0	.
**Individual**		**Odds Ratio**	**LCI**	**UCI**	**Odds Ratio**	**LCI**	**UCI**	**Odds Ratio**	**LCI**	**UCI**	**Odds Ratio**	**LCI**	**UCI**	**Odds Ratio**	**LCI**	**UCI**
Gender	Male	(ref)	(ref)	(ref)	(ref)	(ref)	(ref)	(ref)	(ref)	(ref)	(ref)	(ref)	(ref)	(ref)	(ref)	(ref)
	Female	0.880	0.782	0.990	0.897	0.804	1.001	0.93	0.84	1.03	0.921	0.831	1.022	0.856	0.768	0.953
Ethnicity	White - British	(ref)	(ref)	(ref)	(ref)	(ref)	(ref)	(ref)	(ref)	(ref)	(ref)	(ref)	(ref)	(ref)	(ref)	(ref)
	Any other White background	0.934	0.607	1.438	0.817	0.560	1.191	0.93	0.66	1.32	1.096	0.785	1.529	1.193	0.876	1.623
	Asian or Asian British	0.540	0.192	1.518	0.894	0.470	1.703	0.76	0.34	1.71	0.998	0.484	2.056	0.773	0.381	1.567
	Mixed/Dual background	0.992	0.558	1.762	0.734	0.441	1.222	0.80	0.51	1.25	1.011	0.687	1.487	0.867	0.555	1.356
	Any other ethnic group	1.190	0.549	2.580	0.904	0.380	2.153	0.57	0.26	1.26	0.882	0.474	1.638	1.103	0.616	1.973
SES	IMD 2010	1.789	0.984	3.253	1.873	1.074	3.267	1.15	0.67	1.98	1.764	1.040	2.991	1.357	0.789	2.333
NCMP participation (non-participation >0.2)		0.959	0.802	1.147	1.079	0.919	1.266	1.07	0.87	1.32	1.132	0.943	1.360	1.285	0.972	1.698
**Factor**		**Odds Ratio**	**LCI**	**UCI**	**Odds Ratio**	**LCI**	**UCI**	**Odds Ratio**	**LCI**	**UCI**	**Odds Ratio**	**LCI**	**UCI**	**Odds Ratio**	**LCI**	**UCI**
Gender	Male	(ref)	(ref)	(ref)	(ref)	(ref)	(ref)	(ref)	(ref)	(ref)	(ref)	(ref)	(ref)	(ref)	(ref)	(ref)
	Female	0.880	0.782	0.990	0.898	0.804	1.002	0.933	0.840	1.035	0.921	0.831	1.022	0.856	0.769	0.953
Ethnicity	White - British	(ref)	(ref)	(ref)	(ref)	(ref)	(ref)	(ref)	(ref)	(ref)	(ref)	(ref)	(ref)	(ref)	(ref)	(ref)
	Any other White background	0.934	0.607	1.439	0.820	0.562	1.195	0.935	0.663	1.319	1.099	0.788	1.534	1.191	0.875	1.621
	Asian or Asian British	0.540	0.192	1.517	0.880	0.462	1.676	0.768	0.342	1.724	1.008	0.489	2.078	0.769	0.379	1.560
	Mixed/Dual background	0.991	0.558	1.762	0.735	0.442	1.225	0.799	0.510	1.252	1.015	0.690	1.493	0.866	0.554	1.355
	Any other ethnic group	1.188	0.548	2.579	0.887	0.373	2.112	0.574	0.260	1.267	0.887	0.477	1.648	1.098	0.613	1.965
SES	IMD 2010	1.779	0.957	3.310	1.575	0.882	2.812	1.220	0.692	2.149	1.883	1.083	3.274	1.310	0.740	2.318
NCMP participation (non-participation >0.2)		0.959	0.802	1.147	1.088	0.927	1.276	1.072	0.871	1.319	1.135	0.945	1.363	1.291	0.976	1.707
Factor 1		1.009	0.784	1.298	1.245	0.994	1.560	0.918	0.728	1.157	0.918	0.739	1.139	1.046	0.831	1.315
**Interaction**		**Odds Ratio**	**LCI**	**UCI**	**Odds Ratio**	**LCI**	**UCI**	**Odds Ratio**	**LCI**	**UCI**	**Odds Ratio**	**LCI**	**UCI**	**Odds Ratio**	**LCI**	**UCI**
Gender	Male	(ref)	(ref)	(ref)	(ref)	(ref)	(ref)	(ref)	(ref)	(ref)	(ref)	(ref)	(ref)	(ref)	(ref)	(ref)
	Female	0.879	0.781	0.989	0.899	0.806	1.003	0.931	0.839	1.034	0.922	0.831	1.022	0.856	0.769	0.953
Ethnicity	White - British	(ref)	(ref)	(ref)	(ref)	(ref)	(ref)	(ref)	(ref)	(ref)	(ref)	(ref)	(ref)	(ref)	(ref)	(ref)
	Any other White background	0.962	0.625	1.482	0.839	0.576	1.223	0.937	0.664	1.321	1.107	0.793	1.545	1.197	0.879	1.630
	Asian or Asian British	0.542	0.193	1.522	0.886	0.465	1.687	0.762	0.339	1.712	1.018	0.494	2.098	0.771	0.380	1.566
	Mixed/Dual background	0.990	0.557	1.759	0.743	0.446	1.237	0.797	0.509	1.249	1.024	0.696	1.508	0.872	0.557	1.364
	Any other ethnic group	1.213	0.559	2.630	0.891	0.375	2.120	0.575	0.260	1.271	0.889	0.479	1.653	1.095	0.612	1.960
SES	IMD 2010	1.844	0.994	3.423	1.557	0.872	2.779	1.207	0.684	2.129	1.877	1.079	3.266	1.316	0.743	2.329
NCMP participation (non-participation >0.2)		0.953	0.798	1.138	1.072	0.914	1.256	1.075	0.871	1.326	1.157	0.963	1.389	1.264	0.953	1.675
Factor 1		1.099	0.763	1.581	1.388	1.001	1.925	1.104	0.798	1.527	0.887	0.652	1.207	1.142	0.821	1.588
Year group	Reception	(ref)	(ref)	(ref)	(ref)	(ref)	(ref)	(ref)	(ref)	(ref)	(ref)	(ref)	(ref)	(ref)	(ref)	(ref)
	Year 6	1.330	0.897	1.972	1.324	0.931	1.883	1.304	0.913	1.863	1.047	0.748	1.466	1.192	0.836	1.699
Year group*factor	Reception	(ref)	(ref)	(ref)	(ref)	(ref)	(ref)	(ref)	(ref)	(ref)	(ref)	(ref)	(ref)	(ref)	(ref)	(ref)
	Year 6	0.861	0.530	1.398	0.838	0.550	1.277	0.697	0.448	1.084	1.096	0.729	1.648	0.852	0.555	1.309

Supporting file 3 to Williams, A.J., Wyatt, K.M., Williams, C.A., Logan, S., and Henley, W.E. Exploring the potential of a school impact on pupil weight status: exploratory factor analysis and repeat cross-sectional study of the National Child Measurement Programme.

S3 file - Detailed results and sensitivity analysis

Factor 1 - Deprviation

Red shading indicates significance (p<0.05), Green shading indicates modles which had to be estimated in R

Obese (UK90)

**Single level**		**2006/07**			**2007/08**			**2008/09**			**2009/10**			**2010/11**		
n		9910			11353			11696			11851			11016		
Log likelihood	**Null**	-3583.69			-4023.42			-4569.33			-4491.54			-4218.10		
	**Individual**	-3567.36			-4005.35			-4548.51			-4469.42			-4184.92		
	**Factor**	-3567.05			-4003.16			-4541.22			-4468.65			-4181.27		
**Individual**		**Odds Ratio**	**LCI**	**UCI**	**Odds Ratio**	**LCI**	**UCI**	**Odds Ratio**	**LCI**	**UCI**	**Odds Ratio**	**LCI**	**UCI**	**Odds Ratio**	**LCI**	**UCI**
Gender	Male	(ref)	(ref)	(ref)	(ref)	(ref)	(ref)	(ref)	(ref)	(ref)	(ref)	(ref)	(ref)	(ref)	(ref)	(ref)
	Female	0.807	0.714	0.913	0.765	0.680	0.860	0.780	0.700	0.869	0.839	0.752	0.935	0.711	0.635	0.797
Ethnicity	White - British	(ref)	(ref)	(ref)	(ref)	(ref)	(ref)	(ref)	(ref)	(ref)	(ref)	(ref)	(ref)	(ref)	(ref)	(ref)
	Any other White background	0.880	0.551	1.404	0.986	0.679	1.433	1.154	0.830	1.604	1.000	0.698	1.433	0.786	0.542	1.140
	Asian or Asian British	0.835	0.330	2.112	1.134	0.614	2.097	1.031	0.487	2.183	1.071	0.526	2.181	1.071	0.563	2.039
	Mixed/Dual background	1.962	1.246	3.090	0.960	0.586	1.573	1.049	0.685	1.607	0.942	0.622	1.426	0.949	0.604	1.490
	Any other ethnic group	1.179	0.530	2.625	1.630	0.791	3.359	1.355	0.757	2.426	1.285	0.737	2.240	0.618	0.284	1.344
SES	IMD 2010	2.893	1.611	5.196	2.992	1.704	5.256	3.052	1.847	5.043	3.977	2.371	6.669	4.059	2.394	6.880
NCMP participation (non-participation >0.2)		1.069	0.899	1.270	0.996	0.841	1.181	1.093	0.898	1.330	0.745	0.604	0.920	1.257	0.948	1.667
**Factor**		**Odds Ratio**	**LCI**	**UCI**	**Odds Ratio**	**LCI**	**UCI**	**Odds Ratio**	**LCI**	**UCI**	**Odds Ratio**	**LCI**	**UCI**	**Odds Ratio**	**LCI**	**UCI**
Gender	Male	(ref)	(ref)	(ref)	(ref)	(ref)	(ref)	(ref)	(ref)	(ref)	(ref)	(ref)	(ref)	(ref)	(ref)	(ref)
	Female	0.807	0.714	0.913	0.765	0.680	0.860	0.779	0.699	0.868	0.838	0.751	0.935	0.712	0.635	0.797
Ethnicity	White - British	(ref)	(ref)	(ref)	(ref)	(ref)	(ref)	(ref)	(ref)	(ref)	(ref)	(ref)	(ref)	(ref)	(ref)	(ref)
	Any other White background	0.880	0.551	1.405	0.990	0.681	1.438	1.152	0.828	1.601	0.994	0.694	1.425	0.780	0.538	1.132
	Asian or Asian British	0.830	0.328	2.098	1.113	0.602	2.057	0.986	0.465	2.090	1.054	0.517	2.147	1.031	0.541	1.964
	Mixed/Dual background	1.954	1.241	3.078	0.963	0.588	1.579	1.051	0.686	1.610	0.938	0.619	1.421	0.942	0.599	1.480
	Any other ethnic group	1.165	0.523	2.593	1.596	0.774	3.289	1.299	0.726	2.328	1.269	0.728	2.213	0.599	0.276	1.303
SES	IMD 2010	2.676	1.445	4.954	2.414	1.331	4.379	2.112	1.239	3.601	3.514	2.024	6.101	3.083	1.757	5.409
NCMP participation (non-participation >0.2)		1.070	0.901	1.272	1.006	0.849	1.193	1.100	0.904	1.339	0.742	0.601	0.917	1.302	0.981	1.729
Factor 1		1.106	0.861	1.420	1.284	1.015	1.625	1.535	1.230	1.916	1.148	0.922	1.429	1.381	1.092	1.747

**Reception two-level**		**2006/07**			**2007/08**			**2008/09**			**2009/10**			**2010/11**		
n		4352			5190			5421			5373			4970		
Log likelihood	**Null**	-1217.69			-1459.60			-1732.98			-1560.98			-1507.99		
	**Individual**	-1210.86			-1447.28			-1727.23			-1551.17			-1497.76		
	**Factor**	-1209.55			-1446.28			-1723.0419			-1549.51			-1496.73		
School variance	**Null**	3.47E-20	0 .		0.091	0.023	0.364	0.145	0.071	0.298	0.305	0.175	0.530	0.106	0.032	0.351
	**Individual**	8.49E-13	0 .		0.049	0.004	0.568	0.136	0.064	0.288	0.235	0.123	0.450	0.105	0.032	0.338
	**Factor**	6.36E-13	0 .		0.042	0.002	0.742	0.123	0.056	0.273	0.225	0.115	0.442	0.095	0.027	0.341
**Individual**		**Odds Ratio**	**LCI**	**UCI**	**Odds Ratio**	**LCI**	**UCI**	**Odds Ratio**	**LCI**	**UCI**	**Odds Ratio**	**LCI**	**UCI**	**Odds Ratio**	**LCI**	**UCI**
Gender	Male	(ref)	(ref)	(ref)	(ref)	(ref)	(ref)	(ref)	(ref)	(ref)	(ref)	(ref)	(ref)	(ref)	(ref)	(ref)
	Female	0.824	0.661	1.027	0.821	0.671	1.005	0.797	0.664	0.956	0.764	0.628	0.930	0.703	0.577	0.858
Ethnicity	White - British	(ref)	(ref)	(ref)	(ref)	(ref)	(ref)	(ref)	(ref)	(ref)	(ref)	(ref)	(ref)	(ref)	(ref)	(ref)
	Any other White background	1.028	0.533	1.985	1.299	0.791	2.133	1.151	0.672	1.971	0.665	0.331	1.338	1.077	0.640	1.812
	Asian or Asian British	1.996	0.680	5.860	0.897	0.315	2.553	0.626	0.144	2.717	1.477	0.556	3.927	0.919	0.322	2.618
	Mixed/Dual background	1.872	0.834	4.203	0.443	0.139	1.415	1.082	0.569	2.056	1.064	0.560	2.020	1.135	0.581	2.217
	Any other ethnic group	0.405	0.055	2.989	0.495	0.066	3.733	1.091	0.378	3.150	0.758	0.227	2.533	1.222	0.426	3.502
SES	IMD 2010	3.599	1.308	9.900	7.312	2.861	18.687	2.478	1.010	6.082	4.185	1.559	11.238	3.945	1.511	10.298
NCMP participation (non-participation >0.2)		1.010	0.726	1.405	0.843	0.592	1.199	1.276	0.781	2.083	0.702	0.467	1.057	1.236	0.537	2.842
**Factor**		**Odds Ratio**	**LCI**	**UCI**	**Odds Ratio**	**LCI**	**UCI**	**Odds Ratio**	**LCI**	**UCI**	**Odds Ratio**	**LCI**	**UCI**	**Odds Ratio**	**LCI**	**UCI**
Gender	Male	(ref)	(ref)	(ref)	(ref)	(ref)	(ref)	(ref)	(ref)	(ref)	(ref)	(ref)	(ref)	(ref)	(ref)	(ref)
	Female	0.824	0.661	1.028	0.823	0.672	1.007	0.794	0.662	0.953	0.763	0.627	0.929	0.704	0.578	0.859
Ethnicity	White - British	(ref)	(ref)	(ref)	(ref)	(ref)	(ref)	(ref)	(ref)	(ref)	(ref)	(ref)	(ref)	(ref)	(ref)	(ref)
	Any other White background	1.033	0.535	1.994	1.314	0.801	2.156	1.152	0.673	1.972	0.656	0.326	1.319	1.066	0.633	1.793
	Asian or Asian British	1.934	0.658	5.680	0.880	0.310	2.500	0.589	0.136	2.553	1.422	0.535	3.781	0.888	0.311	2.531
	Mixed/Dual background	1.811	0.806	4.070	0.445	0.139	1.421	1.087	0.572	2.065	1.057	0.557	2.006	1.131	0.579	2.210
	Any other ethnic group	0.384	0.052	2.837	0.487	0.065	3.665	1.053	0.365	3.039	0.728	0.218	2.431	1.172	0.409	3.364
SES	IMD 2010	2.750	0.954	7.927	5.574	2.046	15.186	1.607	0.630	4.097	3.195	1.152	8.865	3.058	1.107	8.447
NCMP participation (non-participation >0.2)		1.028	0.738	1.432	0.856	0.602	1.217	1.313	0.805	2.142	0.683	0.453	1.029	1.281	0.558	2.945
Factor 1		1.440	0.923	2.245	1.360	0.888	2.083	1.872	1.222	2.867	1.545	0.967	2.471	1.383	0.889	2.149

**Year 6 two-level**		**2006/07**			**2007/08**			**2008/09**			**2009/10**			**2010/11**		
n		5558			6163			6275			6478			6046		
Log likelihood	**Null**	-2312.74			-2506.79			-2771.22			-2833.62			-2648.44		
	**Individual**	-2299.05			-2494.93			-2756.94			-2825.60			-2624.68		
	**Factor**	-2299.04			-2493.47			-2753.69			-2825.25			-2622.58		
School variance	**Null**	0.027	0.003	0.261	0.089	0.037	0.218	0.092	0.043	0.197	0.074	0.030	0.179	0.033	0.006	0.173
	**Individual**	0.014	2.27E-04	0.906	0.086	0.034	0.217	0.075	0.032	0.177	0.066	0.025	0.174	0.005	3.68E-07	71.441
	**Factor**	0.015	2.52E-04	0.861	0.082	0.031	0.214	0.070	0.028	0.171	0.065	0.025	0.174	0.001	4.05E-25	2.32E+18
**Individual**		**Odds Ratio**	**LCI**	**UCI**	**Odds Ratio**	**LCI**	**UCI**	**Odds Ratio**	**LCI**	**UCI**	**Odds Ratio**	**LCI**	**UCI**	**Odds Ratio**	**LCI**	**UCI**
Gender	Male	(ref)	(ref)	(ref)	(ref)	(ref)	(ref)	(ref)	(ref)	(ref)	(ref)	(ref)	(ref)	(ref)	(ref)	(ref)
	Female	0.794	0.683	0.923	0.737	0.636	0.854	0.778	0.679	0.893	0.886	0.774	1.014	0.717	0.623	0.826
Ethnicity	White - British	(ref)	(ref)	(ref)	(ref)	(ref)	(ref)	(ref)	(ref)	(ref)	(ref)	(ref)	(ref)	(ref)	(ref)	(ref)
	Any other White background	0.986	0.502	1.935	0.881	0.487	1.594	1.201	0.782	1.846	1.288	0.831	1.997	0.683	0.395	1.178
	Asian or Asian British	0.266	0.036	1.977	1.319	0.599	2.908	1.213	0.490	3.000	0.897	0.306	2.628	1.351	0.581	3.140
	Mixed/Dual background	2.028	1.160	3.545	1.359	0.768	2.406	1.106	0.614	1.992	1.001	0.570	1.756	0.944	0.507	1.759
	Any other ethnic group	2.111	0.826	5.395	2.222	0.970	5.088	1.588	0.769	3.282	1.571	0.816	3.024	0.367	0.113	1.188
SES	IMD 2010	2.999	1.428	6.296	1.766	0.804	3.879	3.418	1.709	6.835	3.201	1.569	6.532	4.870	2.500	9.487
NCMP participation (non-participation >0.2)		1.030	0.836	1.270	0.936	0.746	1.173	0.870	0.680	1.113	1.007	0.754	1.346	1.041	0.766	1.415
**Factor**		**Odds Ratio**	**LCI**	**UCI**	**Odds Ratio**	**LCI**	**UCI**	**Odds Ratio**	**LCI**	**UCI**	**Odds Ratio**	**LCI**	**UCI**	**Odds Ratio**	**LCI**	**UCI**
Gender	Male	(ref)	(ref)	(ref)	(ref)	(ref)	(ref)	(ref)	(ref)	(ref)	(ref)	(ref)	(ref)	(ref)	(ref)	(ref)
	Female	0.794	0.683	0.923	0.737	0.636	0.854	0.779	0.679	0.893	0.886	0.774	1.014	0.718	0.624	0.826
Ethnicity	White - British	(ref)	(ref)	(ref)	(ref)	(ref)	(ref)	(ref)	(ref)	(ref)	(ref)	(ref)	(ref)	(ref)	(ref)	(ref)
	Any other White background	0.986	0.502	1.936	0.877	0.485	1.586	1.195	0.778	1.836	1.282	0.827	1.989	0.682	0.395	1.177
	Asian or Asian British	0.266	0.036	1.976	1.301	0.590	2.865	1.183	0.479	2.925	0.884	0.302	2.593	1.304	0.561	3.031
	Mixed/Dual background	2.028	1.160	3.545	1.362	0.770	2.411	1.107	0.615	1.994	0.996	0.568	1.748	0.933	0.501	1.739
	Any other ethnic group	2.108	0.824	5.389	2.179	0.951	4.990	1.523	0.737	3.149	1.561	0.811	3.007	0.361	0.112	1.167
SES	IMD 2010	2.954	1.349	6.471	1.482	0.661	3.322	2.585	1.258	5.312	2.919	1.387	6.142	3.792	1.881	7.643
NCMP participation (non-participation >0.2)		1.030	0.835	1.270	0.944	0.753	1.182	0.869	0.680	1.111	1.011	0.756	1.351	1.077	0.792	1.465
Factor 1		1.019	0.740	1.403	1.328	0.958	1.841	1.493	1.096	2.034	1.137	0.840	1.539	1.352	1.014	1.803

**Three-level**		**2006/07**			**2007/08**			**2008/09**			**2009/10**			**2010/11**		
n		9910			11353			11696			11851			11016		
Log likelihood	**Null**	-3574.27			-4002.79			-4542.37			-4448.49			-4200.78		
	**Individual**	-3559.41			-3986.74			-4524.94			-4435.32			-4171.06		
	**Factor**	-3558.94			-3985.19			-4519.48			-4434.81			-4168.60		
	**Interaction**	-3511.46			-3944.71			-4478.56			-4380.98			-4122.02		
School variance	**Null**	2.61E-18	0 .		2.56E-14	0 .		1.74E-13	0 .		2.13E-17	0 .		1.23E-14	0 .	
	**Individual**	2.45E-21	0 .		6.01E-11	0 .		8.58E-11	0 .		7.45E-20	0 .		1.42E-12	0 .	
	**Factor**	1.14E-14	0 .		2.72E-14	0 .		8.33E-16	0 .		2.13E-20	0 .		2.98E-15	0 .	
	**Interaction**	0.026	0.004	0.160	0.055	0.016	0.192	0.032	0.005	0.194	0.043	0.009	0.196	0.026	0.006	0.115
Year group variar	**Null**	0.122	0.066	0.226	0.191	0.123	0.296	0.182	0.122	0.273	0.261	0.183	0.372	0.152	0.095	0.245
	**Individual**	0.114	0.059	0.219	0.181	0.114	0.286	0.167	0.109	0.255	0.233	0.159	0.341	0.132	0.079	0.222
	**Factor**	0.115	0.060	0.220	0.178	0.112	0.283	0.159	0.103	0.245	0.233	0.159	0.342	0.127	0.074	0.217
	**Interaction**	9.80E-13	0 .		0.021	0.001	0.834	0.059	0.017	0.200	0.072	0.023	0.231	1.46E-09	0 .	
**Individual**		**Odds Ratio**	**LCI**	**UCI**	**Odds Ratio**	**LCI**	**UCI**	**Odds Ratio**	**LCI**	**UCI**	**Odds Ratio**	**LCI**	**UCI**	**Odds Ratio**	**LCI**	**UCI**
Gender	Male	(ref)	(ref)	(ref)	(ref)	(ref)	(ref)	(ref)	(ref)	(ref)	(ref)	(ref)	(ref)	(ref)	(ref)	(ref)
	Female	0.800	0.706	0.906	0.758	0.673	0.854	0.783	0.702	0.874	0.839	0.751	0.938	0.707	0.630	0.793
Ethnicity	White - British	(ref)	(ref)	(ref)	(ref)	(ref)	(ref)	(ref)	(ref)	(ref)	(ref)	(ref)	(ref)	(ref)	(ref)	(ref)
	Any other White background	0.915	0.569	1.470	1.011	0.687	1.487	1.159	0.828	1.623	1.031	0.713	1.490	0.807	0.553	1.179
	Asian or Asian British	0.866	0.339	2.214	1.069	0.568	2.013	0.966	0.450	2.074	1.129	0.546	2.334	1.104	0.574	2.124
	Mixed/Dual background	1.991	1.254	3.159	0.983	0.595	1.623	1.066	0.691	1.645	1.014	0.664	1.548	0.965	0.610	1.524
	Any other ethnic group	1.242	0.550	2.806	1.543	0.735	3.241	1.392	0.767	2.526	1.313	0.741	2.327	0.621	0.283	1.361
SES	IMD 2010	2.669	1.413	5.042	2.756	1.471	5.167	2.895	1.646	5.094	3.216	1.762	5.871	3.856	2.156	6.895
NCMP participation (non-participation >0.2)		1.053	0.866	1.281	0.957	0.779	1.177	1.079	0.852	1.366	0.824	0.639	1.063	1.227	0.887	1.698
**Factor**		**Odds Ratio**	**LCI**	**UCI**	**Odds Ratio**	**LCI**	**UCI**	**Odds Ratio**	**LCI**	**UCI**	**Odds Ratio**	**LCI**	**UCI**	**Odds Ratio**	**LCI**	**UCI**
Gender	Male	(ref)	(ref)	(ref)	(ref)	(ref)	(ref)	(ref)	(ref)	(ref)	(ref)	(ref)	(ref)	(ref)	(ref)	(ref)
	Female	0.800	0.706	0.906	0.759	0.673	0.855	0.783	0.701	0.873	0.839	0.750	0.938	0.707	0.630	0.794
Ethnicity	White - British	(ref)	(ref)	(ref)	(ref)	(ref)	(ref)	(ref)	(ref)	(ref)	(ref)	(ref)	(ref)	(ref)	(ref)	(ref)
	Any other White background	0.916	0.570	1.472	1.012	0.688	1.488	1.156	0.826	1.619	1.026	0.710	1.484	0.802	0.550	1.171
	Asian or Asian British	0.860	0.336	2.197	1.054	0.560	1.984	0.934	0.435	2.005	1.114	0.538	2.304	1.070	0.556	2.059
	Mixed/Dual background	1.982	1.249	3.146	0.984	0.596	1.625	1.067	0.691	1.646	1.011	0.662	1.543	0.958	0.606	1.515
	Any other ethnic group	1.225	0.542	2.768	1.521	0.724	3.194	1.347	0.742	2.444	1.302	0.735	2.307	0.607	0.277	1.331
SES	IMD 2010	2.441	1.261	4.724	2.362	1.235	4.519	2.198	1.225	3.943	2.959	1.589	5.511	3.142	1.714	5.758
NCMP participation (non-participation >0.2)		1.054	0.866	1.283	0.967	0.787	1.189	1.085	0.857	1.372	0.822	0.637	1.060	1.265	0.914	1.751
Factor 1		1.154	0.865	1.538	1.289	0.971	1.711	1.569	1.200	2.050	1.158	0.871	1.538	1.356	1.036	1.775
**Interaction**		**Odds Ratio**	**LCI**	**UCI**	**Odds Ratio**	**LCI**	**UCI**	**Odds Ratio**	**LCI**	**UCI**	**Odds Ratio**	**LCI**	**UCI**	**Odds Ratio**	**LCI**	**UCI**
Gender	Male	(ref)	(ref)	(ref)	(ref)	(ref)	(ref)	(ref)	(ref)	(ref)	(ref)	(ref)	(ref)	(ref)	(ref)	(ref)
	Female	0.802	0.708	0.908	0.765	0.679	0.862	0.784	0.703	0.875	0.839	0.751	0.938	0.713	0.636	0.799
Ethnicity	White - British	(ref)	(ref)	(ref)	(ref)	(ref)	(ref)	(ref)	(ref)	(ref)	(ref)	(ref)	(ref)	(ref)	(ref)	(ref)
	Any other White background	1.013	0.632	1.625	1.127	0.769	1.652	1.178	0.842	1.647	1.041	0.721	1.502	0.852	0.585	1.240
	Asian or Asian British	0.881	0.346	2.243	1.108	0.591	2.077	0.950	0.443	2.034	1.166	0.564	2.407	1.136	0.593	2.179
	Mixed/Dual background	1.961	1.239	3.106	1.001	0.607	1.651	1.100	0.713	1.697	1.043	0.684	1.592	1.010	0.640	1.595
	Any other ethnic group	1.265	0.563	2.845	1.532	0.733	3.203	1.342	0.740	2.434	1.284	0.728	2.267	0.603	0.276	1.319
SES	IMD 2010	2.766	1.447	5.286	2.380	1.254	4.515	2.145	1.203	3.823	3.000	1.629	5.523	3.350	1.865	6.017
NCMP participation (non-participation >0.2)		1.035	0.864	1.240	0.919	0.761	1.110	0.927	0.740	1.161	0.848	0.669	1.074	1.096	0.816	1.473
Factor 1		1.439	0.928	2.232	1.530	1.005	2.329	1.757	1.181	2.613	1.538	1.015	2.331	1.373	0.920	2.047
Year group	Reception	(ref)	(ref)	(ref)	(ref)	(ref)	(ref)	(ref)	(ref)	(ref)	(ref)	(ref)	(ref)	(ref)	(ref)	(ref)
	Year 6	2.572	1.670	3.962	2.228	1.473	3.370	2.036	1.363	3.040	2.666	1.759	4.039	1.914	1.287	2.845
Year group*factor	Reception	(ref)	(ref)	(ref)	(ref)	(ref)	(ref)	(ref)	(ref)	(ref)	(ref)	(ref)	(ref)	(ref)	(ref)	(ref)
	Year 6	0.718	0.429	1.202	0.817	0.502	1.331	0.877	0.545	1.412	0.733	0.448	1.201	0.995	0.624	1.586

Supporting file 3 to Williams, A.J., Wyatt, K.M., Williams, C.A., Logan, S., and Henley, W.E. Exploring the potential of a school impact on pupil weight status: exploratory factor analysis and repeat cross-sectional study of the National Child Measurement Programme.

S3 file - Detailed results and sensitivity analysis

Factor 1 - Deprviation

Red shading indicates significance (p<0.05), Green shading indicates modles which had to be estimated in R

Overweight (IOTF)

Single level		2006/07			2007/08			2008/09			2009/10			2010/11		
n		9532			10955			11217			11345			10559		
Log likelihood	**Null**	-4160.76			-4786.96			-5421.59			-5292.83			-4916.86		
	**Individual**	-4143.36			-4767.80			-5401.96			-5271.96			-4908.28		
	**Factor**	-4143.00			-4762.20			-5401.35			-5271.24			-4906.26		
**Individual**		**Odds Ratio**	**LCI**	**UCI**	**Odds Ratio**	**LCI**	**UCI**	**Odds Ratio**	**LCI**	**UCI**	**Odds Ratio**	**LCI**	**UCI**	**Odds Ratio**	**LCI**	**UCI**
Gender	Male	(ref)	(ref)	(ref)	(ref)	(ref)	(ref)	(ref)	(ref)	(ref)	(ref)	(ref)	(ref)	(ref)	(ref)	(ref)
	Female	1.319	1.181	1.473	1.322	1.193	1.466	1.288	1.171	1.417	1.346	1.222	1.483	1.129	1.021	1.248
Ethnicity	White - British	(ref)	(ref)	(ref)	(ref)	(ref)	(ref)	(ref)	(ref)	(ref)	(ref)	(ref)	(ref)	(ref)	(ref)	(ref)
	Any other White background	1.047	0.705	1.554	0.807	0.563	1.157	1.115	0.830	1.499	1.071	0.781	1.467	1.089	0.808	1.467
	Asian or Asian British	0.738	0.312	1.744	0.779	0.411	1.475	0.910	0.458	1.809	0.964	0.486	1.912	0.761	0.388	1.490
	Mixed/Dual background	1.409	0.886	2.241	0.721	0.445	1.169	1.085	0.746	1.578	0.896	0.615	1.305	0.951	0.631	1.432
	Any other ethnic group	1.348	0.670	2.710	1.031	0.481	2.209	0.950	0.519	1.737	1.061	0.623	1.806	0.516	0.248	1.073
SES	IMD 2010	2.098	1.225	3.592	1.829	1.095	3.054	2.124	1.345	3.353	1.694	1.049	2.735	1.368	0.831	2.253
NCMP participation (non-participation >0.2)		1.071	0.917	1.250	1.077	0.930	1.247	1.099	0.923	1.308	0.963	0.810	1.144	1.358	1.058	1.743
**Factor**		**Odds Ratio**	**LCI**	**UCI**	**Odds Ratio**	**LCI**	**UCI**	**Odds Ratio**	**LCI**	**UCI**	**Odds Ratio**	**LCI**	**UCI**	**Odds Ratio**	**LCI**	**UCI**
Gender	Male	(ref)	(ref)	(ref)	(ref)	(ref)	(ref)	(ref)	(ref)	(ref)	(ref)	(ref)	(ref)	(ref)	(ref)	(ref)
	Female	1.319	1.181	1.473	1.325	1.195	1.468	1.288	1.171	1.417	1.347	1.222	1.484	1.129	1.022	1.248
Ethnicity	White - British	(ref)	(ref)	(ref)	(ref)	(ref)	(ref)	(ref)	(ref)	(ref)	(ref)	(ref)	(ref)	(ref)	(ref)	(ref)
	Any other White background	1.048	0.705	1.556	0.813	0.567	1.166	1.114	0.828	1.497	1.076	0.785	1.475	1.083	0.804	1.459
	Asian or Asian British	0.734	0.311	1.735	0.760	0.401	1.438	0.899	0.452	1.788	0.979	0.493	1.943	0.742	0.378	1.453
	Mixed/Dual background	1.401	0.881	2.228	0.725	0.447	1.176	1.086	0.747	1.579	0.899	0.617	1.310	0.947	0.629	1.428
	Any other ethnic group	1.329	0.660	2.674	0.999	0.466	2.140	0.941	0.514	1.721	1.072	0.630	1.826	0.505	0.243	1.051
SES	IMD 2010	1.951	1.112	3.423	1.359	0.793	2.329	1.939	1.195	3.144	1.870	1.127	3.104	1.151	0.681	1.946
NCMP participation (non-participation >0.2)		1.072	0.918	1.253	1.093	0.943	1.266	1.100	0.924	1.310	0.966	0.813	1.149	1.391	1.083	1.787
Factor 1		1.103	0.881	1.381	1.426	1.157	1.758	1.116	0.919	1.354	0.888	0.731	1.078	1.241	1.005	1.532

Reception two-level		2006/07			2007/08			2008/09			2009/10			2010/11		
n		4214			5017			5199			5165			4783		
Log likelihood	**Null**	-1716.68			-1998.43			-2399.35			-2231.13			-2109.18		
	**Individual**	-1704.54			-1975.03			-2381.45			-2215.84			-2098.70		
	**Factor**	-1702.84			-1974.21			-2381.36			-2215.74			-2097.78		
School variance	**Null**	0.045	0.007	0.277	0.097	0.038	0.250	0.127	0.071	0.229	0.037	0.005	0.276	0.057	0.015	0.209
	**Individual**	0.030	0.002	0.452	0.080	0.027	0.242	0.120	0.065	0.220	0.036	0.005	0.285	0.065	0.020	0.210
	**Factor**	0.022	0.001	0.815	0.073	0.022	0.241	0.119	0.064	0.219	0.037	0.005	0.281	0.059	0.017	0.212
**Individual**		**Odds Ratio**	**LCI**	**UCI**	**Odds Ratio**	**LCI**	**UCI**	**Odds Ratio**	**LCI**	**UCI**	**Odds Ratio**	**LCI**	**UCI**	**Odds Ratio**	**LCI**	**UCI**
Gender	Male	(ref)	(ref)	(ref)	(ref)	(ref)	(ref)	(ref)	(ref)	(ref)	(ref)	(ref)	(ref)	(ref)	(ref)	(ref)
	Female	1.510	1.267	1.800	1.521	1.290	1.793	1.467	1.267	1.699	1.471	1.262	1.713	1.355	1.159	1.585
Ethnicity	White - British	(ref)	(ref)	(ref)	(ref)	(ref)	(ref)	(ref)	(ref)	(ref)	(ref)	(ref)	(ref)	(ref)	(ref)	(ref)
	Any other White background	0.913	0.525	1.589	0.704	0.418	1.183	0.835	0.515	1.353	1.174	0.746	1.846	1.338	0.899	1.992
	Asian or Asian British	1.150	0.385	3.431	0.266	0.063	1.112	0.676	0.226	2.019	0.578	0.174	1.920	0.529	0.185	1.508
	Mixed/Dual background	1.236	0.572	2.674	0.334	0.121	0.922	1.189	0.722	1.959	0.877	0.513	1.502	1.270	0.751	2.149
	Any other ethnic group	1.004	0.343	2.937	0.563	0.129	2.454	1.251	0.534	2.928	0.929	0.385	2.246	0.486	0.148	1.602
SES	IMD 2010	2.056	0.862	4.905	3.447	1.515	7.841	1.738	0.810	3.728	1.793	0.845	3.802	1.169	0.519	2.632
NCMP participation (non-participation >0.2)		1.005	0.767	1.317	0.917	0.687	1.224	1.633	1.106	2.409	0.830	0.636	1.082	1.347	0.691	2.629
**Factor**		**Odds Ratio**	**LCI**	**UCI**	**Odds Ratio**	**LCI**	**UCI**	**Odds Ratio**	**LCI**	**UCI**	**Odds Ratio**	**LCI**	**UCI**	**Odds Ratio**	**LCI**	**UCI**
Gender	Male	(ref)	(ref)	(ref)	(ref)	(ref)	(ref)	(ref)	(ref)	(ref)	(ref)	(ref)	(ref)	(ref)	(ref)	(ref)
	Female	1.510	1.267	1.800	1.523	1.292	1.795	1.466	1.266	1.698	1.471	1.262	1.714	1.355	1.159	1.585
Ethnicity	White - British	(ref)	(ref)	(ref)	(ref)	(ref)	(ref)	(ref)	(ref)	(ref)	(ref)	(ref)	(ref)	(ref)	(ref)	(ref)
	Any other White background	0.915	0.526	1.592	0.710	0.422	1.193	0.835	0.515	1.354	1.177	0.748	1.852	1.325	0.891	1.972
	Asian or Asian British	1.122	0.377	3.340	0.261	0.063	1.094	0.671	0.225	2.005	0.583	0.175	1.938	0.517	0.181	1.475
	Mixed/Dual background	1.194	0.552	2.583	0.335	0.121	0.923	1.189	0.722	1.960	0.879	0.513	1.504	1.268	0.750	2.144
	Any other ethnic group	0.952	0.326	2.785	0.556	0.128	2.421	1.246	0.532	2.917	0.939	0.388	2.273	0.472	0.143	1.556
SES	IMD 2010	1.676	0.691	4.067	2.867	1.210	6.794	1.660	0.752	3.664	1.898	0.861	4.188	0.983	0.422	2.288
NCMP participation (non-participation >0.2)		1.022	0.781	1.338	0.929	0.697	1.240	1.639	1.111	2.419	0.834	0.639	1.089	1.382	0.709	2.694
Factor 1		1.407	0.979	2.022	1.267	0.884	1.816	1.077	0.760	1.526	0.927	0.672	1.279	1.278	0.898	1.821

Year 6 two-level		2006/07			2007/08			2008/09			2009/10			2010/11		
n		5318			5938			6018			6180			5776		
Log likelihood	**Null**	-2435.38			-2765.54			-3002.77			-3044.09			-2797.82		
	**Individual**	-2426.60			-2760.43			-2993.90			-3034.06			-2794.09		
	**Factor**	-2426.50			-2756.31			-2993.53			-3033.97			-2793.29		
School variance	**Null**	7.39E-16	0	.	0.075	0.029	0.196	0.051	0.017	0.155	0.049	0.016	0.153	0.038	0.009	0.160
	**Individual**	4.22E-16	0	.	0.071	0.026	0.194	0.050	0.016	0.152	0.046	0.014	0.153	0.031	0.005	0.172
	**Factor**	3.62E-13	0	.	0.057	0.018	0.186	0.049	0.016	0.151	0.045	0.013	0.153	0.030	0.005	0.172
**Individual**		**Odds Ratio**	**LCI**	**UCI**	**Odds Ratio**	**LCI**	**UCI**	**Odds Ratio**	**LCI**	**UCI**	**Odds Ratio**	**LCI**	**UCI**	**Odds Ratio**	**LCI**	**UCI**
Gender	Male	(ref)	(ref)	(ref)	(ref)	(ref)	(ref)	(ref)	(ref)	(ref)	(ref)	(ref)	(ref)	(ref)	(ref)	(ref)
	Female	1.202	1.042	1.387	1.219	1.065	1.395	1.185	1.043	1.346	1.274	1.122	1.447	0.996	0.872	1.137
Ethnicity	White - British	(ref)	(ref)	(ref)	(ref)	(ref)	(ref)	(ref)	(ref)	(ref)	(ref)	(ref)	(ref)	(ref)	(ref)	(ref)
	Any other White background	1.384	0.777	2.464	1.076	0.638	1.812	1.386	0.939	2.045	1.037	0.664	1.619	0.920	0.576	1.469
	Asian or Asian British	0.435	0.102	1.856	1.376	0.644	2.941	1.053	0.424	2.616	1.450	0.606	3.472	1.066	0.432	2.632
	Mixed/Dual background	1.533	0.854	2.752	1.109	0.625	1.969	0.942	0.522	1.698	0.964	0.564	1.648	0.691	0.352	1.358
	Any other ethnic group	1.905	0.736	4.933	1.369	0.542	3.456	0.765	0.316	1.852	1.176	0.596	2.322	0.544	0.213	1.388
SES	IMD 2010	2.182	1.074	4.435	1.025	0.485	2.163	2.423	1.264	4.645	1.758	0.888	3.480	1.538	0.775	3.052
NCMP participation (non-participation >0.2)		1.083	0.892	1.315	1.092	0.893	1.336	0.904	0.723	1.131	1.246	0.962	1.615	1.285	0.963	1.714
**Factor**		**Odds Ratio**	**LCI**	**UCI**	**Odds Ratio**	**LCI**	**UCI**	**Odds Ratio**	**LCI**	**UCI**	**Odds Ratio**	**LCI**	**UCI**	**Odds Ratio**	**LCI**	**UCI**
Gender	Male	(ref)	(ref)	(ref)	(ref)	(ref)	(ref)	(ref)	(ref)	(ref)	(ref)	(ref)	(ref)	(ref)	(ref)	(ref)
	Female	1.202	1.042	1.387	1.219	1.065	1.395	1.185	1.043	1.347	1.275	1.122	1.448	0.996	0.872	1.138
Ethnicity	White - British	(ref)	(ref)	(ref)	(ref)	(ref)	(ref)	(ref)	(ref)	(ref)	(ref)	(ref)	(ref)	(ref)	(ref)	(ref)
	Any other White background	1.383	0.777	2.462	1.068	0.634	1.798	1.382	0.937	2.040	1.038	0.665	1.622	0.919	0.575	1.468
	Asian or Asian British	0.436	0.102	1.860	1.349	0.632	2.879	1.045	0.421	2.596	1.460	0.609	3.496	1.046	0.423	2.585
	Mixed/Dual background	1.535	0.855	2.756	1.119	0.631	1.985	0.942	0.522	1.699	0.966	0.565	1.652	0.687	0.350	1.350
	Any other ethnic group	1.919	0.741	4.971	1.325	0.525	3.343	0.756	0.312	1.830	1.178	0.597	2.325	0.538	0.211	1.373
SES	IMD 2010	2.297	1.089	4.846	0.787	0.369	1.677	2.219	1.125	4.378	1.831	0.899	3.728	1.341	0.656	2.742
NCMP participation (non-participation >0.2)		1.083	0.893	1.315	1.107	0.908	1.350	0.904	0.723	1.130	1.244	0.960	1.612	1.310	0.981	1.750
Factor 1		0.936	0.696	1.259	1.555	1.152	2.100	1.133	0.854	1.503	0.944	0.714	1.248	1.205	0.902	1.610

Three-level		2006/07			2007/08			2008/09			2009/10			2010/11		
n		9532			10955			11217			11345			10559		
Log likelihood	**Null**	-4160.65			-4774.86			-5407.81			-5287.50			-4912.78		
	**Individual**	-4143.36			-4756.93			-5388.58			-5266.85			-4904.63		
	**Factor**	-4143.00			-4753.05			-5388.25			-5266.36			-4902.99		
	**Interaction**	-4133.34			-4741.04			-5383.74			-5252.77			-4897.39		
School variance	**Null**	8.23E-11	0	.	1.53E-07	0	.	2.04E-10	0	.	0.017	0.001	0.314	0.009	4.04E-05	1.870
	**Individual**	4.56E-17	0	.	7.04E-10	0	.	2.13E-15	0	.	0.014	4.15E-04	0.475	0.014	4.61E-04	0.399
	**Factor**	5.39E-18	0	.	4.94E-12	0	.	2.87E-18	0	.	0.013	3.35E-04	0.528	0.011	1.59E-04	0.730
	**Interaction**	1.76E-13	0	.	0.011	5.80E-05	1.902	1.93E-08	0	.	0.035	0.009	0.132	0.020	0.002	0.192
Year group variai	**Null**	9.30E-03	1.07E-04	0.813	0.115	0.067	0.196	0.095	0.057	0.156	0.048	0.013	0.183	0.047	0.013	0.168
	**Individual**	2.04E-14	0	.	0.109	0.062	0.190	0.093	0.056	0.154	0.050	0.014	0.184	0.038	0.008	0.172
	**Factor**	4.55E-16	0	.	0.099	0.054	0.179	0.092	0.055	0.153	0.050	0.013	0.184	0.038	0.008	0.173
	**Interaction**	1.55E-13	0	.	0.056	0.015	0.207	0.087	0.052	0.148	0.010	3.00E-05	3.025	0.021	0.002	0.285
**Individual**		**Odds Ratio**	**LCI**	**UCI**	**Odds Ratio**	**LCI**	**UCI**	**Odds Ratio**	**LCI**	**UCI**	**Odds Ratio**	**LCI**	**UCI**	**Odds Ratio**	**LCI**	**UCI**
Gender	Male	(ref)	(ref)	(ref)	(ref)	(ref)	(ref)	(ref)	(ref)	(ref)	(ref)	(ref)	(ref)	(ref)	(ref)	(ref)
	Female	1.319	1.181	1.473	1.331	1.200	1.478	1.301	1.181	1.432	1.350	1.225	1.489	1.130	1.021	1.250
Ethnicity	White - British	(ref)	(ref)	(ref)	(ref)	(ref)	(ref)	(ref)	(ref)	(ref)	(ref)	(ref)	(ref)	(ref)	(ref)	(ref)
	Any other White background	1.047	0.705	1.554	0.834	0.577	1.205	1.107	0.819	1.496	1.098	0.799	1.511	1.105	0.817	1.494
	Asian or Asian British	0.738	0.312	1.744	0.769	0.403	1.469	0.874	0.435	1.757	0.965	0.483	1.925	0.743	0.377	1.463
	Mixed/Dual background	1.409	0.886	2.241	0.727	0.446	1.184	1.076	0.736	1.574	0.909	0.622	1.329	0.958	0.634	1.448
	Any other ethnic group	1.348	0.670	2.710	1.000	0.462	2.166	0.961	0.521	1.772	1.084	0.633	1.858	0.517	0.248	1.080
SES	IMD 2010	2.098	1.225	3.592	1.657	0.947	2.902	2.099	1.273	3.461	1.699	1.013	2.849	1.310	0.770	2.229
NCMP participation (non-participation >0.2)		1.071	0.917	1.250	1.067	0.900	1.266	1.096	0.899	1.338	0.991	0.819	1.198	1.370	1.045	1.797
**Factor**		**Odds Ratio**	**LCI**	**UCI**	**Odds Ratio**	**LCI**	**UCI**	**Odds Ratio**	**LCI**	**UCI**	**Odds Ratio**	**LCI**	**UCI**	**Odds Ratio**	**LCI**	**UCI**
Gender	Male	(ref)	(ref)	(ref)	(ref)	(ref)	(ref)	(ref)	(ref)	(ref)	(ref)	(ref)	(ref)	(ref)	(ref)	(ref)
	Female	1.319	1.181	1.473	1.332	1.201	1.479	1.301	1.181	1.432	1.351	1.225	1.490	1.130	1.022	1.250
Ethnicity	White - British	(ref)	(ref)	(ref)	(ref)	(ref)	(ref)	(ref)	(ref)	(ref)	(ref)	(ref)	(ref)	(ref)	(ref)	(ref)
	Any other White background	1.048	0.705	1.556	0.836	0.579	1.207	1.105	0.818	1.494	1.102	0.801	1.516	1.099	0.813	1.486
	Asian or Asian British	0.734	0.311	1.735	0.753	0.395	1.438	0.867	0.431	1.743	0.977	0.489	1.950	0.728	0.370	1.434
	Mixed/Dual background	1.401	0.881	2.228	0.729	0.448	1.188	1.077	0.737	1.574	0.912	0.624	1.333	0.955	0.632	1.443
	Any other ethnic group	1.329	0.660	2.674	0.979	0.452	2.119	0.954	0.517	1.760	1.093	0.638	1.873	0.508	0.243	1.061
SES	IMD 2010	1.951	1.112	3.423	1.335	0.751	2.372	1.976	1.175	3.323	1.833	1.070	3.140	1.135	0.654	1.968
NCMP participation (non-participation >0.2)		1.072	0.918	1.253	1.083	0.914	1.283	1.098	0.900	1.340	0.993	0.821	1.201	1.396	1.065	1.831
Factor 1		1.103	0.881	1.381	1.408	1.108	1.789	1.098	0.878	1.374	0.894	0.717	1.114	1.236	0.983	1.554
**Interaction**		**Odds Ratio**	**LCI**	**UCI**	**Odds Ratio**	**LCI**	**UCI**	**Odds Ratio**	**LCI**	**UCI**	**Odds Ratio**	**LCI**	**UCI**	**Odds Ratio**	**LCI**	**UCI**
Gender	Male	(ref)	(ref)	(ref)	(ref)	(ref)	(ref)	(ref)	(ref)	(ref)	(ref)	(ref)	(ref)	(ref)	(ref)	(ref)
	Female	1.318	1.180	1.472	1.336	1.204	1.482	1.302	1.182	1.433	1.352	1.227	1.491	1.132	1.024	1.253
Ethnicity	White - British	(ref)	(ref)	(ref)	(ref)	(ref)	(ref)	(ref)	(ref)	(ref)	(ref)	(ref)	(ref)	(ref)	(ref)	(ref)
	Any other White background	1.095	0.737	1.628	0.868	0.602	1.253	1.109	0.820	1.498	1.115	0.811	1.532	1.121	0.829	1.516
	Asian or Asian British	0.737	0.312	1.743	0.766	0.402	1.461	0.873	0.434	1.754	0.998	0.500	1.993	0.746	0.379	1.469
	Mixed/Dual background	1.389	0.873	2.211	0.737	0.452	1.199	1.090	0.746	1.594	0.931	0.637	1.360	0.974	0.644	1.471
	Any other ethnic group	1.352	0.671	2.723	0.986	0.456	2.133	0.952	0.516	1.756	1.084	0.633	1.856	0.506	0.242	1.057
SES	IMD 2010	2.028	1.155	3.561	1.354	0.765	2.396	1.972	1.174	3.314	1.808	1.055	3.099	1.152	0.664	1.997
NCMP participation (non-participation >0.2)		1.063	0.910	1.242	1.051	0.893	1.239	1.052	0.861	1.285	1.033	0.857	1.244	1.330	1.016	1.742
Factor 1		1.378	0.979	1.940	1.392	0.984	1.969	1.049	0.757	1.455	0.912	0.667	1.249	1.255	0.898	1.754
Year group	Reception	(ref)	(ref)	(ref)	(ref)	(ref)	(ref)	(ref)	(ref)	(ref)	(ref)	(ref)	(ref)	(ref)	(ref)	(ref)
	Year 6	1.688	1.174	2.425	1.294	0.897	1.867	1.103	0.778	1.565	1.294	0.940	1.781	1.229	0.868	1.740
Year group*factor	Reception	(ref)	(ref)	(ref)	(ref)	(ref)	(ref)	(ref)	(ref)	(ref)	(ref)	(ref)	(ref)	(ref)	(ref)	(ref)
	Year 6	0.691	0.446	1.071	1.060	0.681	1.650	1.104	0.717	1.699	1.026	0.697	1.511	0.975	0.640	1.484

Supporting file 3 to Williams, A.J., Wyatt, K.M., Williams, C.A., Logan, S., and Henley, W.E. Exploring the potential of a school impact on pupil weight status: exploratory factor analysis and repeat cross-sectional study of the National Child Measurement Programme.

S3 file - Detailed results and sensitivity analysis

Factor 1 - Deprviation

Red shading indicates significance (p<0.05), Green shading indicates modles which had to be estimated in R

Obese (IOTF)

**Single level**		**2006/07**			**2007/08**			**2008/09**			**2009/10**			**2010/11**		
n		9910			11353			11696			11851			11016		
Log likelihood	**Null**	-1605.40			-1724.55			-1999.60			-2090.78			-1901.75		
	**Individual**	-1595.21			-1703.72			-1989.84			-2072.60			-1869.13		
	**Factor**	-1595.20			-1701.87			-1978.43			-2064.97			-1867.19		
**Individual**		**Odds Ratio**	**LCI**	**UCI**	**Odds Ratio**	**LCI**	**UCI**	**Odds Ratio**	**LCI**	**UCI**	**Odds Ratio**	**LCI**	**UCI**	**Odds Ratio**	**LCI**	**UCI**
Gender	Male	(ref)	(ref)	(ref)	(ref)	(ref)	(ref)	(ref)	(ref)	(ref)	(ref)	(ref)	(ref)	(ref)	(ref)	(ref)
	Female	1.066	0.868	1.310	1.207	0.987	1.475	1.255	1.045	1.508	1.220	1.020	1.459	1.308	1.083	1.580
Ethnicity	White - British	(ref)	(ref)	(ref)	(ref)	(ref)	(ref)	(ref)	(ref)	(ref)	(ref)	(ref)	(ref)	(ref)	(ref)	(ref)
	Any other White background	0.977	0.455	2.094	1.994	1.236	3.219	1.097	0.624	1.929	1.293	0.761	2.195	0.720	0.368	1.411
	Asian or Asian British	1.522	0.470	4.930	1.761	0.759	4.086	0.796	0.193	3.275	1.410	0.507	3.918	2.012	0.911	4.442
	Mixed/Dual background	1.619	0.747	3.504	1.083	0.476	2.466	1.078	0.527	2.205	0.752	0.352	1.609	1.467	0.768	2.804
	Any other ethnic group	1.503	0.465	4.855	2.289	0.819	6.401	2.249	1.027	4.925	1.443	0.627	3.323	1.456	0.586	3.621
SES	IMD 2010	7.403	2.939	18.646	7.276	2.922	18.121	3.863	1.682	8.873	9.268	4.174	20.580	19.715	8.886	43.742
NCMP participation (non-participation >0.2)		0.877	0.644	1.195	0.601	0.421	0.858	0.930	0.651	1.328	0.823	0.587	1.154	0.678	0.369	1.245
**Factor**		**Odds Ratio**	**LCI**	**UCI**	**Odds Ratio**	**LCI**	**UCI**	**Odds Ratio**	**LCI**	**UCI**	**Odds Ratio**	**LCI**	**UCI**	**Odds Ratio**	**LCI**	**UCI**
Gender	Male	(ref)	(ref)	(ref)	(ref)	(ref)	(ref)	(ref)	(ref)	(ref)	(ref)	(ref)	(ref)	(ref)	(ref)	(ref)
	Female	1.066	0.868	1.310	1.208	0.988	1.476	1.255	1.044	1.508	1.217	1.018	1.456	1.309	1.084	1.581
Ethnicity	White - British	(ref)	(ref)	(ref)	(ref)	(ref)	(ref)	(ref)	(ref)	(ref)	(ref)	(ref)	(ref)	(ref)	(ref)	(ref)
	Any other White background	0.977	0.456	2.095	2.012	1.246	3.247	1.092	0.621	1.922	1.251	0.736	2.126	0.716	0.366	1.402
	Asian or Asian British	1.519	0.469	4.922	1.723	0.743	3.996	0.725	0.176	2.988	1.312	0.472	3.644	1.943	0.880	4.286
	Mixed/Dual background	1.616	0.746	3.501	1.091	0.479	2.484	1.086	0.530	2.222	0.738	0.345	1.580	1.456	0.762	2.784
	Any other ethnic group	1.497	0.463	4.843	2.216	0.792	6.199	2.087	0.953	4.575	1.357	0.589	3.126	1.401	0.563	3.486
SES	IMD 2010	7.232	2.707	19.317	5.085	1.915	13.502	1.701	0.702	4.123	4.679	1.978	11.066	13.820	5.789	32.991
NCMP participation (non-participation >0.2)		0.878	0.645	1.195	0.610	0.427	0.871	0.943	0.660	1.347	0.802	0.571	1.125	0.706	0.383	1.299
Factor 1		1.030	0.678	1.565	1.491	0.990	2.246	2.535	1.720	3.735	2.073	1.431	3.003	1.484	1.001	2.201

**Reception two-level**		**2006/07**			**2007/08**			**2008/09**			**2009/10**			**2010/11**		
n		4352			5190			5421			5373			4970		
Log likelihood	**Null**	-612.05			-756.86			-925.76			-854.75			-796.40		
	**Individual**	-605.01			-742.63			-914.05			-848.53			-786.91		
	**Factor**	-604.94			-740.44			-906.68			-846.46			-786.31		
School variance	**Null**	7.88E-12	0	.	0.205	0.056	0.750	0.117	0.024	0.577	0.458	0.255	0.823	0.110	0.010	1.184
	**Individual**	3.72E-16	0	.	0.151			0.126			0.384	0.202	0.732	0.117	0.015	0.929
	**Factor**	1.39E-14	0	.	0.127			0.082	0.009	0.756	0.346	0.174	0.687	0.105	0.011	1.031
**Individual**		**Odds Ratio**	**LCI**	**UCI**	**Odds Ratio**	**LCI**	**UCI**	**Odds Ratio**	**LCI**	**UCI**	**Odds Ratio**	**LCI**	**UCI**	**Odds Ratio**	**LCI**	**UCI**
Gender	Male	(ref)	(ref)	(ref)	(ref)	(ref)	(ref)	(ref)	(ref)	(ref)	(ref)	(ref)	(ref)	(ref)	(ref)	(ref)
	Female	1.443	1.023	2.034	1.369	1.000	1.876	1.675	1.264	2.221	1.139	0.857	1.515	1.236	0.920	1.660
Ethnicity	White - British	(ref)	(ref)	(ref)	(ref)	(ref)	(ref)	(ref)	(ref)	(ref)	(ref)	(ref)	(ref)	(ref)	(ref)	(ref)
	Any other White background	1.375	0.551	3.428	2.273	1.224	4.222	1.339	0.634	2.826	1.149	0.513	2.574	0.951	0.412	2.197
	Asian or Asian British	3.585	1.053	12.208	1.743	0.511	5.943	3.52E-06	0	.	3.548	1.191	10.567	2.297	0.795	6.635
	Mixed/Dual background	1.999	0.613	6.520	7.40E-07	0	.	0.683	0.209	2.232	0.647	0.201	2.085	0.820	0.255	2.631
	Any other ethnic group	1.063	0.143	7.903	6.68E-07	0	.	1.423	0.327	6.199	1.260	0.291	5.457	2.223	0.668	7.398
SES	IMD 2010	5.261	1.155	23.970	6.738	1.563	29.048	4.295	1.217	15.162	6.086	1.539	24.074	12.443	3.260	47.488
NCMP participation (non-participation >0.2)		0.861	0.499	1.484	0.558	0.289	1.077	0.726	0.304	1.736	0.782	0.437	1.398	0.822	0.192	3.519
**Factor**		**Odds Ratio**	**LCI**	**UCI**	**Odds Ratio**	**LCI**	**UCI**	**Odds Ratio**	**LCI**	**UCI**	**Odds Ratio**	**LCI**	**UCI**	**Odds Ratio**	**LCI**	**UCI**
Gender	Male	(ref)	(ref)	(ref)	(ref)	(ref)	(ref)	(ref)	(ref)	(ref)	(ref)	(ref)	(ref)	(ref)	(ref)	(ref)
	Female	1.443	1.024	2.035	1.378	1.007	1.885	1.667	1.263	2.200	1.136	0.855	1.510	1.237	0.921	1.662
Ethnicity	White - British	(ref)	(ref)	(ref)	(ref)	(ref)	(ref)	(ref)	(ref)	(ref)	(ref)	(ref)	(ref)	(ref)	(ref)	(ref)
	Any other White background	1.377	0.552	3.435	2.343	1.265	4.339	1.346	0.646	2.807	1.125	0.503	2.520	0.943	0.409	2.178
	Asian or Asian British	3.547	1.041	12.089	1.670	0.493	5.659	0.000	0.000	.	3.300	1.108	9.825	2.217	0.767	6.406
	Mixed/Dual background	1.973	0.604	6.447	7.50E-07	0	.	0.694	0.217	2.219	0.641	0.199	2.067	0.817	0.255	2.623
	Any other ethnic group	1.041	0.140	7.762	6.32E-07	0	.	1.326	0.312	5.647	1.175	0.272	5.077	2.122	0.636	7.076
SES	IMD 2010	4.778	0.971	23.504	3.627	0.768	17.116	1.657	0.447	6.145	3.886	0.940	16.070	9.112	2.140	38.799
NCMP participation (non-participation >0.2)		0.865	0.501	1.494	0.572	0.297	1.102	0.759	0.324	1.777	0.748	0.418	1.340	0.848	0.198	3.633
Factor 1		1.142	0.576	2.265	2.054	1.029	4.101	3.250	1.765	5.984	2.029	1.027	4.010	1.432	0.750	2.734

**Year 6 two-level**		**2006/07**			**2007/08**			**2008/09**			**2009/10**			**2010/11**		
n		5558			6163			6275			6478			6046		
Log likelihood	**Null**	-987.29			-963.28			-1068.95			-1206.63			-1102.13		
	**Individual**	-979.22			-950.71			-1064.68			-1197.20			-1076.18		
	**Factor**	-979.16			-950.32			-1061.10			-1194.18			-1075.06		
School variance	**Null**	0.123	0.032	0.474	0.213	0.072	0.627	0.253	0.101	0.633	0.151	0.046	0.494	0.119	0.027	0.535
	**Individual**	0.057	0.004	0.758	0.185	0.056	0.616	0.233	0.088	0.618	0.112			0.007	4.94E-11	8.76E+05
	**Factor**	0.066			0.190	0.059	0.618	0.222	0.079	0.624	0.070			8.11E-09	0	.
**Individual**		**Odds Ratio**	**LCI**	**UCI**	**Odds Ratio**	**LCI**	**UCI**	**Odds Ratio**	**LCI**	**UCI**	**Odds Ratio**	**LCI**	**UCI**	**Odds Ratio**	**LCI**	**UCI**
Gender	Male	(ref)	(ref)	(ref)	(ref)	(ref)	(ref)	(ref)	(ref)	(ref)	(ref)	(ref)	(ref)	(ref)	(ref)	(ref)
	Female	0.891	0.686	1.156	1.089	0.832	1.426	0.996	0.774	1.283	1.675	1.264	2.221	1.367	1.068	1.750
Ethnicity	White - British	(ref)	(ref)	(ref)	(ref)	(ref)	(ref)	(ref)	(ref)	(ref)	(ref)	(ref)	(ref)	(ref)	(ref)	(ref)
	Any other White background	0.684	0.166	2.820	1.636	0.700	3.821	0.877	0.353	2.178	1.339	0.634	2.826	0.501	0.157	1.593
	Asian or Asian British	0.000	0	.	1.715	0.504	5.838	1.807	0.419	7.796	3.52E-06	0	.	1.790	0.531	6.031
	Mixed/Dual background	1.426	0.511	3.974	2.136	0.909	5.020	1.591	0.630	4.020	0.683	0.209	2.232	2.321	1.049	5.133
	Any other ethnic group	2.142	0.494	9.287	4.246	1.426	12.646	3.255	1.235	8.577	1.423	0.327	6.199	0.957	0.229	3.990
SES	IMD 2010	8.900	2.628	30.136	7.916	2.112	29.672	2.819	0.789	10.075	4.295	1.217	15.162	31.501	11.229	88.371
NCMP participation (non-participation >0.2)		0.853	0.579	1.257	0.585	0.364	0.940	0.970	0.620	1.516	0.726	0.304	1.736	0.597	0.303	1.176
**Factor**		**Odds Ratio**	**LCI**	**UCI**	**Odds Ratio**	**LCI**	**UCI**	**Odds Ratio**	**LCI**	**UCI**	**Odds Ratio**	**LCI**	**UCI**	**Odds Ratio**	**LCI**	**UCI**
Gender	Male	(ref)	(ref)	(ref)	(ref)	(ref)	(ref)	(ref)	(ref)	(ref)	(ref)	(ref)	(ref)	(ref)	(ref)	(ref)
	Female	0.890	0.684	1.159	1.089	0.832	1.426	0.998	0.775	1.285	1.319	1.040	1.672	1.367	1.068	1.750
Ethnicity	White - British	(ref)	(ref)	(ref)	(ref)	(ref)	(ref)	(ref)	(ref)	(ref)	(ref)	(ref)	(ref)	(ref)	(ref)	(ref)
	Any other White background	0.685	0.163	2.871	1.629	0.697	3.806	0.870	0.351	2.160	1.317	0.631	2.748	0.499	0.157	1.586
	Asian or Asian British	4.03E-06	0	.	1.701	0.500	5.781	1.723	0.399	7.430	3.01E-06	0	.	1.730	0.517	5.797
	Mixed/Dual background	1.425	0.507	4.011	2.145	0.912	5.042	1.601	0.634	4.046	0.881	0.316	2.457	2.292	1.036	5.070
	Any other ethnic group	2.138	0.487	9.381	4.167	1.398	12.424	3.021	1.146	7.966	1.576	0.556	4.467	0.930	0.223	3.874
SES	IMD 2010	8.753	2.381	32.183	6.554	1.647	26.091	1.584	0.421	5.961	3.788	1.117	12.848	21.929	7.107	67.662
NCMP participation (non-participation >0.2)		0.853	0.576	1.265	0.587	0.365	0.945	0.967	0.618	1.514	1.134	0.705	1.824	0.623	0.316	1.228
Factor 1		1.006	0.573	1.768	1.305	0.724	2.352	2.199	1.225	3.949	1.890	1.137	3.141	1.471	0.886	2.444

**Three-level**		**2006/07**			**2007/08**			**2008/09**			**2009/10**			**2010/11**		
n		9910			11353			11696			11851			11016		
Log likelihood	**Null**	-1602.53			-1719.64			-1992.75			-2063.11			-1898.27		
	**Individual**	-1594.02			-1700.41			-1984.67			-2053.82			-1868.08		
	**Factor**	-1593.99			-1698.72			-1974.89			-2049.79			-1866.56		
	**Interaction**	-1589.24			-1697.25			-1974.33			-2046.80			-1864.09		
School variance	**Null**	0.122	0.034	0.435	0.108	0.023	0.508	0.164	0.064	0.419	0.124			0.140	0.047	0.418
	**Individual**	0.074	0.010	0.551	0.098	0.020	0.484	0.135	0.045	0.411	0.081	0.013	0.499	0.057	0.006	0.533
	**Factor**	0.075	0.010	0.547	0.086	0.014	0.537	0.099	0.023	0.436	0.056	0.004	0.718	0.044	0.003	0.714
	**Interaction**	0.069	0.012	0.382	0.098	0.020	0.477	0.098	0.022	0.441	0.064	0.007	0.552	0.043	0.005	0.374
Year group variar	**Null**	0.007	1.29E-11	3.64E+06	0.097	0.010	0.914	0.035	4.31E-04	2.823	0.206			0.002	3.05E-43	1.03E+37
	**Individual**	0.012	1.75E-07	883.061	0.063	0.003	1.488	0.041	0.001	1.801	0.173	0.059	0.506	0.007	2.17E-13	2.01E+08
	**Factor**	0.013	3.00E-07	562.578	0.075	0.005	1.173	0.050	0.002	1.330	0.171	0.058	0.502	0.003	3.99E-25	2.44E+19
	**Interaction**	3.39E-13	0	.	0.052	0.001	2.416	0.052	0.002	1.208	0.158	0.053	0.472	3.46E-10	0	.
**Individual**		**Odds Ratio**	**LCI**	**UCI**	**Odds Ratio**	**LCI**	**UCI**	**Odds Ratio**	**LCI**	**UCI**	**Odds Ratio**	**LCI**	**UCI**	**Odds Ratio**	**LCI**	**UCI**
Gender	Male	(ref)	(ref)	(ref)	(ref)	(ref)	(ref)	(ref)	(ref)	(ref)	(ref)	(ref)	(ref)	(ref)	(ref)	(ref)
	Female	1.064	0.866	1.308	1.200	0.981	1.469	1.262	1.049	1.518	1.234	1.030	1.479	1.310	1.084	1.583
Ethnicity	White - British	(ref)	(ref)	(ref)	(ref)	(ref)	(ref)	(ref)	(ref)	(ref)	(ref)	(ref)	(ref)	(ref)	(ref)	(ref)
	Any other White background	0.979	0.455	2.108	2.011	1.229	3.290	1.120	0.633	1.982	1.255	0.730	2.156	0.730	0.372	1.432
	Asian or Asian British	1.550	0.473	5.075	1.689	0.713	4.002	0.796	0.191	3.313	1.560	0.554	4.393	2.056	0.926	4.566
	Mixed/Dual background	1.654	0.760	3.597	1.113	0.486	2.546	1.093	0.532	2.249	0.779	0.362	1.677	1.453	0.758	2.784
	Any other ethnic group	1.537	0.470	5.023	2.280	0.801	6.488	2.341	1.054	5.198	1.518	0.650	3.547	1.483	0.593	3.705
SES	IMD 2010	6.827	2.568	18.149	7.068	2.639	18.934	3.241	1.292	8.127	5.636	2.250	14.115	20.503	8.866	47.418
NCMP participation (non-participation >0.2)		0.885	0.642	1.219	0.574	0.394	0.837	0.932	0.637	1.364	0.902	0.621	1.310	0.679	0.366	1.262
**Factor**		**Odds Ratio**	**LCI**	**UCI**	**Odds Ratio**	**LCI**	**UCI**	**Odds Ratio**	**LCI**	**UCI**	**Odds Ratio**	**LCI**	**UCI**	**Odds Ratio**	**LCI**	**UCI**
Gender	Male	(ref)	(ref)	(ref)	(ref)	(ref)	(ref)	(ref)	(ref)	(ref)	(ref)	(ref)	(ref)	(ref)	(ref)	(ref)
	Female	1.064	0.866	1.309	1.201	0.982	1.470	1.260	1.048	1.516	1.230	1.026	1.474	1.310	1.085	1.583
Ethnicity	White - British	(ref)	(ref)	(ref)	(ref)	(ref)	(ref)	(ref)	(ref)	(ref)	(ref)	(ref)	(ref)	(ref)	(ref)	(ref)
	Any other White background	0.980	0.455	2.109	2.018	1.234	3.302	1.114	0.630	1.970	1.229	0.715	2.112	0.724	0.369	1.420
	Asian or Asian British	1.545	0.472	5.060	1.661	0.702	3.930	0.731	0.175	3.044	1.464	0.520	4.122	1.984	0.894	4.400
	Mixed/Dual background	1.650	0.759	3.591	1.117	0.488	2.557	1.098	0.534	2.258	0.766	0.356	1.649	1.445	0.754	2.769
	Any other ethnic group	1.527	0.466	4.996	2.224	0.782	6.329	2.187	0.986	4.854	1.450	0.621	3.386	1.428	0.571	3.569
SES	IMD 2010	6.540	2.332	18.343	5.177	1.845	14.532	1.647	0.639	4.242	3.774	1.472	9.672	14.916	6.035	36.866
NCMP participation (non-participation >0.2)		0.885	0.642	1.219	0.583	0.399	0.851	0.941	0.643	1.376	0.887	0.611	1.288	0.703	0.379	1.304
Factor 1		1.060	0.676	1.662	1.528	0.970	2.405	2.635	1.706	4.071	1.854	1.213	2.835	1.442	0.957	2.173
**Interaction**		**Odds Ratio**	**LCI**	**UCI**	**Odds Ratio**	**LCI**	**UCI**	**Odds Ratio**	**LCI**	**UCI**	**Odds Ratio**	**LCI**	**UCI**	**Odds Ratio**	**LCI**	**UCI**
Gender	Male	(ref)	(ref)	(ref)	(ref)	(ref)	(ref)	(ref)	(ref)	(ref)	(ref)	(ref)	(ref)	(ref)	(ref)	(ref)
	Female	1.064	0.865	1.308	1.205	0.984	1.474	1.260	1.047	1.516	1.231	1.027	1.475	1.313	1.087	1.587
Ethnicity	White - British	(ref)	(ref)	(ref)	(ref)	(ref)	(ref)	(ref)	(ref)	(ref)	(ref)	(ref)	(ref)	(ref)	(ref)	(ref)
	Any other White background	1.051	0.488	2.263	2.089	1.276	3.421	1.115	0.630	1.973	1.245	0.725	2.138	0.741	0.378	1.456
	Asian or Asian British	1.601	0.490	5.231	1.669	0.706	3.948	0.720	0.173	3.004	1.490	0.529	4.196	2.042	0.920	4.532
	Mixed/Dual background	1.643	0.755	3.575	1.123	0.491	2.571	1.101	0.535	2.265	0.783	0.363	1.685	1.483	0.773	2.843
	Any other ethnic group	1.580	0.483	5.166	2.212	0.778	6.290	2.197	0.990	4.877	1.445	0.619	3.374	1.433	0.573	3.582
SES	IMD 2010	6.956	2.487	19.455	5.077	1.807	14.268	1.630	0.632	4.201	3.723	1.451	9.557	15.153	6.141	37.386
NCMP participation (non-participation >0.2)		0.866	0.629	1.193	0.571	0.391	0.833	0.932	0.634	1.370	0.912	0.628	1.325	0.663	0.356	1.233
Factor 1		1.095	0.560	2.141	1.939	1.007	3.731	3.262	1.788	5.950	2.111	1.131	3.941	1.301	0.716	2.363
Year group	Reception	(ref)	(ref)	(ref)	(ref)	(ref)	(ref)	(ref)	(ref)	(ref)	(ref)	(ref)	(ref)	(ref)	(ref)	(ref)
	Year 6	1.442	0.727	2.863	1.614	0.803	3.245	1.426	0.739	2.750	1.475	0.752	2.891	1.074	0.569	2.030
Year group*factor	Reception	(ref)	(ref)	(ref)	(ref)	(ref)	(ref)	(ref)	(ref)	(ref)	(ref)	(ref)	(ref)	(ref)	(ref)	(ref)
	Year 6	0.964	0.426	2.182	0.675	0.301	1.512	0.680	0.322	1.435	0.850	0.391	1.847	1.185	0.572	2.453

Supporting file 3 to Williams, A.J., Wyatt, K.M., Williams, C.A., Logan, S., and Henley, W.E. Exploring the potential of a school impact on pupil weight status: exploratory factor analysis and repeat cross-sectional study of the National Child Measurement Programme.

S3 file - Detailed results and sensitivity analysis

Factor 1 - Depvriation

Red shading indicates significance (p<0.05), Green shading indicates modles which had to be estimated in R

BMI-SDS

**Single level**		**2006/07**			**2007/08**			**2008/09**			**2009/10**			**2010/11**		
n		9910			11353			11696			11851			11016		
Log likelihood	**Null**	-14914.99			-16983.74			-17154.20			-17558.83			-16084.62		
	**Individual**	-14907.46			-16971.41			-17128.16			-17540.88			-16059.60		
	**Factor**	-14909.63			-16971.73			-17125.90			-17543.14			-16059.20		
Residual varianc	**Null**	1.187	1.155	1.221	1.166	1.136	1.197	1.100	1.072	1.128	1.133	1.104	1.162	1.085	1.057	1.114
	**Individual**	1.183	1.151	1.217	1.162	1.132	1.192	1.093	1.065	1.121	1.128	1.099	1.157	1.079	1.050	1.107
	**Factor**	1.184	1.151	1.217	1.161	1.131	1.192	1.092	1.065	1.121	1.128	1.100	1.157	1.078	1.050	1.107
**Null**		**Mean difference**	**LCI**	**UCI**	**Mean difference**	**LCI**	**UCI**	**Mean difference**	**LCI**	**UCI**	**Mean difference**	**LCI**	**UCI**	**Mean difference**	**LCI**	**UCI**
Constant		0.340	0.319	0.362	0.327	0.307	0.347	0.463	0.444	0.482	0.434	0.415	0.453	0.457	0.438	0.477
**Individual**		**Mean difference**	**LCI**	**UCI**	**Mean difference**	**LCI**	**UCI**	**Mean difference**	**LCI**	**UCI**	**Mean difference**	**LCI**	**UCI**	**Mean difference**	**LCI**	**UCI**
Constant		0.289	0.236	0.342	0.322	0.273	0.370	0.434	0.388	0.480	0.395	0.348	0.441	0.440	0.393	0.486
Gender	Male	(ref)	(ref)	(ref)	(ref)	(ref)	(ref)	(ref)	(ref)	(ref)	(ref)	(ref)	(ref)	(ref)	(ref)	(ref)
	Female	-0.085	-0.127	-0.042	-0.123	-0.162	-0.083	-0.136	-0.173	-0.098	-0.112	-0.150	-0.073	-0.143	-0.182	-0.104
Ethnicity	White - British	(ref)	(ref)	(ref)	(ref)	(ref)	(ref)	(ref)	(ref)	(ref)	(ref)	(ref)	(ref)	(ref)	(ref)	(ref)
	Any other White background	0.040	-0.115	0.194	-0.035	-0.163	0.092	0.083	-0.038	0.205	0.009	-0.118	0.135	0.031	-0.089	0.150
	Asian or Asian British	-0.164	-0.466	0.139	-0.183	-0.409	0.042	-0.205	-0.475	0.064	-0.096	-0.361	0.169	-0.035	-0.268	0.198
	Mixed/Dual background	0.204	0.005	0.402	-0.032	-0.198	0.135	0.021	-0.132	0.174	-0.135	-0.279	0.009	-0.058	-0.213	0.097
	Any other ethnic group	0.034	-0.262	0.331	0.007	-0.287	0.300	0.037	-0.194	0.268	0.156	-0.058	0.371	-0.145	-0.366	0.077
SES	IMD 2010	0.462	0.248	0.676	0.343	0.141	0.545	0.464	0.278	0.650	0.476	0.283	0.668	0.440	0.247	0.633
NCMP participation (non-participation >0.2)		-0.007	-0.068	0.054	-0.007	-0.065	0.051	0.027	-0.044	0.098	0.005	-0.063	0.073	0.038	-0.068	0.144
**Factor**		**Mean difference**	**LCI**	**UCI**	**Mean difference**	**LCI**	**UCI**	**Mean difference**	**LCI**	**UCI**	**Mean difference**	**LCI**	**UCI**	**Mean difference**	**LCI**	**UCI**
Constant		0.295	0.218	0.371	0.271	0.201	0.341	0.360	0.294	0.426	0.386	0.320	0.453	0.380	0.310	0.449
Gender	Male	(ref)	(ref)	(ref)	(ref)	(ref)	(ref)	(ref)	(ref)	(ref)	(ref)	(ref)	(ref)	(ref)	(ref)	(ref)
	Female	-0.085	-0.127	-0.042	-0.122	-0.162	-0.083	-0.136	-0.174	-0.098	-0.112	-0.150	-0.074	-0.143	-0.182	-0.104
Ethnicity	White - British	(ref)	(ref)	(ref)	(ref)	(ref)	(ref)	(ref)	(ref)	(ref)	(ref)	(ref)	(ref)	(ref)	(ref)	(ref)
	Any other White background	0.040	-0.115	0.194	-0.034	-0.161	0.093	0.083	-0.039	0.204	0.008	-0.119	0.135	0.028	-0.091	0.148
	Asian or Asian British	-0.163	-0.466	0.139	-0.190	-0.416	0.035	-0.218	-0.488	0.052	-0.098	-0.363	0.168	-0.047	-0.280	0.186
	Mixed/Dual background	0.204	0.006	0.403	-0.031	-0.197	0.136	0.021	-0.132	0.174	-0.135	-0.279	0.009	-0.060	-0.214	0.095
	Any other ethnic group	0.036	-0.261	0.332	-0.001	-0.294	0.293	0.025	-0.207	0.256	0.155	-0.060	0.370	-0.154	-0.376	0.067
SES	IMD 2010	0.469	0.245	0.692	0.278	0.066	0.490	0.368	0.172	0.564	0.464	0.261	0.667	0.363	0.160	0.567
NCMP participation (non-participation >0.2)		-0.007	-0.069	0.054	-0.003	-0.061	0.055	0.029	-0.042	0.100	0.005	-0.063	0.072	0.049	-0.058	0.155
Factor 1		-0.009	-0.097	0.078	0.081	0.001	0.161	0.119	0.042	0.196	0.014	-0.063	0.091	0.096	0.014	0.177

**Reception two-level**		**2006/07**			**2007/08**			**2008/09**			**2009/10**			**2010/11**		
n		4352			5190			5421			5373			4970		
Log likelihood	**Null**	-6239.98			-7379.80			-7429.67			-7484.45			-6734.36		
	**Individual**	-6245.04			-7371.92			-7428.49			-7486.78			-6735.96		
	**Factor**	-6246.46			-7372.87			-7427.75			-7488.46			-6734.81		
School variance	**Null**	0.041	0.026	0.065	0.042	0.028	0.063	0.025	0.016	0.039	0.040	0.028	0.059	0.014	0.006	0.030
	**Individual**	0.040	0.025	0.064	0.040	0.026	0.062	0.024	0.015	0.038	0.038	0.026	0.057	0.014	0.006	0.029
	**Factor**	0.041	0.025	0.065	0.040	0.026	0.062	0.023	0.014	0.036	0.039	0.026	0.057	0.012	5.38E-03	0.028
Residual varianc	**Null**	1.000	0.958	1.045	0.977	0.939	1.016	0.888	0.855	0.923	0.922	0.887	0.958	0.867	0.833	0.903
	**Individual**	1.001	0.958	1.045	0.972	0.934	1.011	0.886	0.853	0.921	0.921	0.886	0.957	0.866	0.832	0.901
	**Factor**	1.001	0.958	1.045	0.972	0.934	1.011	0.886	0.853	0.921	0.921	0.886	0.957	0.866	0.832	0.902
**Null**		**Mean difference**	**LCI**	**UCI**	**Mean difference**	**LCI**	**UCI**	**Mean difference**	**LCI**	**UCI**	**Mean difference**	**LCI**	**UCI**	**Mean difference**	**LCI**	**UCI**
Constant		0.292	0.251	0.334	0.293	0.255	0.332	0.466	0.433	0.500	0.411	0.373	0.448	0.460	0.429	0.491
**Individual**		**Mean difference**	**LCI**	**UCI**	**Mean difference**	**LCI**	**UCI**	**Mean difference**	**LCI**	**UCI**	**Mean difference**	**LCI**	**UCI**	**Mean difference**	**LCI**	**UCI**
Constant		0.297	0.212	0.381	0.261	0.185	0.338	0.408	0.340	0.477	0.404	0.332	0.477	0.433	0.365	0.501
Gender	Male	(ref)	(ref)	(ref)	(ref)	(ref)	(ref)	(ref)	(ref)	(ref)	(ref)	(ref)	(ref)	(ref)	(ref)	(ref)
	Female	-0.041	-0.101	0.019	-0.087	-0.141	-0.032	-0.054	-0.104	-0.003	-0.063	-0.115	-0.011	-0.071	-0.124	-0.019
Ethnicity	White - British	(ref)	(ref)	(ref)	(ref)	(ref)	(ref)	(ref)	(ref)	(ref)	(ref)	(ref)	(ref)	(ref)	(ref)	(ref)
	Any other White background	-0.049	-0.234	0.137	-0.078	-0.232	0.077	-0.008	-0.166	0.150	-0.024	-0.187	0.140	0.057	-0.086	0.200
	Asian or Asian British	-0.060	-0.454	0.334	-0.550	-0.839	-0.261	-0.511	-0.872	-0.151	-0.166	-0.489	0.157	-0.025	-0.304	0.255
	Mixed/Dual background	0.157	-0.125	0.438	-0.130	-0.350	0.090	-0.029	-0.214	0.157	-0.227	-0.404	-0.050	-0.151	-0.339	0.036
	Any other ethnic group	-0.062	-0.437	0.312	-0.255	-0.674	0.164	-0.003	-0.313	0.306	-0.183	-0.483	0.118	-0.048	-0.344	0.248
SES	IMD 2010	0.174	-0.156	0.504	0.480	0.173	0.788	0.430	0.156	0.704	0.232	-0.057	0.520	0.313	0.037	0.589
NCMP participation (non-participation >0.2)		-0.113	-0.227	0.000	-0.071	-0.185	0.043	0.083	-0.074	0.241	-0.008	-0.121	0.104	0.140	-0.107	0.386
**Factor**		**Mean difference**	**LCI**	**UCI**	**Mean difference**	**LCI**	**UCI**	**Mean difference**	**LCI**	**UCI**	**Mean difference**	**LCI**	**UCI**	**Mean difference**	**LCI**	**UCI**
Constant		0.265	0.128	0.402	0.202	0.078	0.326	0.312	0.205	0.419	0.394	0.275	0.513	0.333	0.229	0.437
Gender	Male	(ref)	(ref)	(ref)	(ref)	(ref)	(ref)	(ref)	(ref)	(ref)	(ref)	(ref)	(ref)	(ref)	(ref)	(ref)
	Female	-0.041	-0.102	0.019	-0.086	-0.141	-0.032	-0.054	-0.105	-0.004	-0.063	-0.115	-0.011	-0.071	-0.123	-0.019
Ethnicity	White - British	(ref)	(ref)	(ref)	(ref)	(ref)	(ref)	(ref)	(ref)	(ref)	(ref)	(ref)	(ref)	(ref)	(ref)	(ref)
	Any other White background	-0.049	-0.234	0.137	-0.076	-0.231	0.079	-0.007	-0.165	0.151	-0.024	-0.187	0.139	0.053	-0.090	0.196
	Asian or Asian British	-0.063	-0.457	0.331	-0.556	-0.845	-0.266	-0.525	-0.886	-0.165	-0.167	-0.490	0.156	-0.040	-0.319	0.240
	Mixed/Dual background	0.154	-0.128	0.436	-0.130	-0.350	0.091	-0.028	-0.213	0.157	-0.228	-0.405	-0.051	-0.153	-0.340	0.034
	Any other ethnic group	-0.066	-0.441	0.309	-0.259	-0.678	0.160	-0.010	-0.320	0.299	-0.184	-0.485	0.117	-0.066	-0.362	0.230
SES	IMD 2010	0.155	-0.181	0.491	0.435	0.118	0.751	0.348	0.065	0.630	0.223	-0.073	0.520	0.210	-0.076	0.496
NCMP participation (non-participation >0.2)		-0.112	-0.225	0.002	-0.066	-0.180	0.048	0.090	-0.067	0.247	-0.009	-0.122	0.104	0.156	-0.090	0.401
Factor 1		0.047	-0.113	0.207	0.090	-0.058	0.237	0.149	0.020	0.277	0.016	-0.126	0.158	0.156	0.032	0.279

**Year 6 two-level**		**2006/07**			**2007/08**			**2008/09**			**2009/10**			**2010/11**		
n		5558			6163			6275			6478			6046		
Log likelihood	**Null**	-8607.29			-9526.30			-9610.21			-9959.39			-9263.90		
	**Individual**	-8598.02			-9519.79			-9588.28			-9944.15			-9240.02		
	**Factor**	-8599.49			-9520.92			-9589.46			-9945.88			-9241.68		
School variance	**Null**	0.021	0.011	0.041	0.018	0.009	0.036	0.031	0.019	0.051	0.023	0.013	0.041	0.003	2.01E-04	0.045
	**Individual**	0.019	0.009	0.039	0.018	0.009	0.036	0.029	0.018	0.048	0.022	0.012	0.040	0.001	1.73E-07	4.743
	**Factor**	0.019	0.009	0.039	0.018	0.009	0.036	0.029	0.018	0.047	0.023	0.013	0.040	0.001	5.39E-07	1.991
Residual varianc	**Null**	1.278	1.230	1.327	1.272	1.227	1.319	1.228	1.185	1.273	1.248	1.205	1.293	1.250	1.206	1.296
	**Individual**	1.273	1.225	1.322	1.267	1.222	1.314	1.219	1.176	1.263	1.240	1.198	1.285	1.240	1.196	1.286
	**Factor**	1.273	1.226	1.322	1.267	1.223	1.314	1.219	1.176	1.263	1.240	1.198	1.285	1.240	1.196	1.286
**Null**		**Mean difference**	**LCI**	**UCI**	**Mean difference**	**LCI**	**UCI**	**Mean difference**	**LCI**	**UCI**	**Mean difference**	**LCI**	**UCI**	**Mean difference**	**LCI**	**UCI**
Constant		0.385	0.348	0.422	0.349	0.315	0.383	0.456	0.420	0.493	0.469	0.435	0.503	0.457	0.428	0.487
**Individual**		**Mean difference**	**LCI**	**UCI**	**Mean difference**	**LCI**	**UCI**	**Mean difference**	**LCI**	**UCI**	**Mean difference**	**LCI**	**UCI**	**Mean difference**	**LCI**	**UCI**
Constant		0.320	0.240	0.400	0.379	0.303	0.456	0.460	0.385	0.536	0.423	0.348	0.497	0.442	0.374	0.510
Gender	Male	(ref)	(ref)	(ref)	(ref)	(ref)	(ref)	(ref)	(ref)	(ref)	(ref)	(ref)	(ref)	(ref)	(ref)	(ref)
	Female	-0.123	-0.183	-0.064	-0.155	-0.212	-0.098	-0.202	-0.257	-0.147	-0.145	-0.199	-0.090	-0.202	-0.259	-0.146
Ethnicity	White - British	(ref)	(ref)	(ref)	(ref)	(ref)	(ref)	(ref)	(ref)	(ref)	(ref)	(ref)	(ref)	(ref)	(ref)	(ref)
	Any other White background	0.184	-0.080	0.448	0.067	-0.154	0.287	0.168	-0.015	0.352	0.060	-0.131	0.250	-0.013	-0.210	0.184
	Asian or Asian British	-0.295	-0.751	0.160	0.166	-0.179	0.511	0.011	-0.382	0.404	0.031	-0.393	0.455	-0.100	-0.482	0.281
	Mixed/Dual background	0.236	-0.037	0.509	0.068	-0.176	0.312	0.053	-0.192	0.299	0.020	-0.208	0.247	0.069	-0.180	0.318
	Any other ethnic group	0.154	-0.312	0.620	0.175	-0.231	0.581	0.056	-0.282	0.394	0.463	0.162	0.763	-0.221	-0.544	0.102
SES	IMD 2010	0.595	0.268	0.922	0.192	-0.125	0.510	0.451	0.148	0.754	0.523	0.214	0.831	0.574	0.291	0.857
NCMP participation (non-participation >0.2)		0.034	-0.062	0.130	0.010	-0.079	0.100	0.005	-0.102	0.111	0.104	-0.021	0.228	0.018	-0.109	0.144
**Factor**		**Mean difference**	**LCI**	**UCI**	**Mean difference**	**LCI**	**UCI**	**Mean difference**	**LCI**	**UCI**	**Mean difference**	**LCI**	**UCI**	**Mean difference**	**LCI**	**UCI**
Constant		0.351	0.229	0.473	0.330	0.216	0.444	0.412	0.295	0.529	0.412	0.299	0.525	0.416	0.315	0.516
Gender	Male	(ref)	(ref)	(ref)	(ref)	(ref)	(ref)	(ref)	(ref)	(ref)	(ref)	(ref)	(ref)	(ref)	(ref)	(ref)
	Female	-0.124	-0.183	-0.064	-0.155	-0.212	-0.098	-0.202	-0.257	-0.147	-0.145	-0.200	-0.090	-0.202	-0.259	-0.146
Ethnicity	White - British	(ref)	(ref)	(ref)	(ref)	(ref)	(ref)	(ref)	(ref)	(ref)	(ref)	(ref)	(ref)	(ref)	(ref)	(ref)
	Any other White background	0.184	-0.080	0.448	0.065	-0.155	0.286	0.167	-0.016	0.351	0.059	-0.131	0.250	-0.013	-0.210	0.184
	Asian or Asian British	-0.294	-0.749	0.162	0.162	-0.183	0.506	0.007	-0.386	0.400	0.029	-0.395	0.454	-0.105	-0.487	0.277
	Mixed/Dual background	0.236	-0.037	0.510	0.069	-0.175	0.312	0.054	-0.192	0.299	0.019	-0.208	0.247	0.067	-0.182	0.316
	Any other ethnic group	0.158	-0.308	0.624	0.170	-0.236	0.576	0.050	-0.289	0.388	0.462	0.161	0.763	-0.224	-0.547	0.100
SES	IMD 2010	0.623	0.286	0.961	0.149	-0.177	0.475	0.411	0.099	0.723	0.513	0.195	0.831	0.541	0.243	0.839
NCMP participation (non-participation >0.2)		0.034	-0.062	0.130	0.012	-0.077	0.101	0.004	-0.102	0.111	0.104	-0.020	0.229	0.022	-0.105	0.149
Factor 1		-0.049	-0.194	0.096	0.076	-0.054	0.207	0.075	-0.065	0.214	0.017	-0.116	0.150	0.042	-0.076	0.159

**Three-level**		**2006/07**			**2007/08**			**2008/09**			**2009/10**			**2010/11**		
n		9910			11353			11696			11851			11016		
Log likelihood	**Null**	-14878.53			-16951.41			-17108.34			-17502.07			-16081.32		
	**Individual**	-14874.00			-16939.27			-17086.60			-17489.98			-16057.03		
	**Factor**	-14875.89			-16940.19			-17086.43			-17491.96			-16056.94		
	**Interaction**	-14871.98			-16941.84			-17090.31			-17492.34			-16061.05		
School variance	**Null**	0.021	0.012	0.038	0.008	0.002	0.029	0.013	0.006	0.027	0.019	0.010	0.034	0.003	3.15E-04	0.028
	**Individual**	0.020	0.011	0.037	0.007	0.002	0.030	0.011	0.005	0.026	0.017	0.009	0.032	0.002	6.69E-05	0.054
	**Factor**	0.021	0.011	0.037	0.007	0.002	0.030	0.010	0.004	0.025	0.018	0.010	0.033	0.001	1.64E-05	0.121
	**Interaction**	0.022	0.013	0.038	0.007	0.002	0.029	0.010	0.004	0.025	0.018	0.010	0.033	0.001	8.73E-06	0.182
Year group varia	**Null**	0.009	0.003	0.034	0.021	0.011	0.038	0.016	0.008	0.030	0.014	0.007	0.030	0.004	5.01E-04	0.031
	**Individual**	0.009	0.002	0.034	0.021	0.012	0.039	0.016	0.008	0.031	0.014	0.007	0.030	0.004	5.71E-04	0.030
	**Factor**	0.009	0.002	0.034	0.021	0.012	0.039	0.016	0.008	0.031	0.014	0.007	0.030	0.004	6.20E-04	0.030
	**Interaction**	0.005	3.89E-04	0.053	0.020	0.011	0.038	0.016	0.008	0.031	0.013	0.006	0.028	0.005	7.47E-04	0.028
Residual varianc	**Null**	1.157	1.125	1.191	1.138	1.108	1.169	1.071	1.043	1.099	1.100	1.072	1.129	1.078	1.050	1.108
	**Individual**	1.155	1.122	1.188	1.134	1.104	1.164	1.066	1.038	1.094	1.097	1.068	1.125	1.073	1.044	1.102
	**Factor**	1.155	1.122	1.188	1.134	1.104	1.164	1.066	1.038	1.094	1.097	1.068	1.125	1.072	1.044	1.102
	**Interaction**	1.155	1.123	1.189	1.134	1.104	1.164	1.066	1.039	1.094	1.096	1.068	1.125	1.072	1.044	1.102
**Null**		**Mean difference**	**LCI**	**UCI**	**Mean difference**	**LCI**	**UCI**	**Mean difference**	**LCI**	**UCI**	**Mean difference**	**LCI**	**UCI**	**Mean difference**	**LCI**	**UCI**
Constant		0.345	0.314	0.376	0.322	0.295	0.349	0.461	0.434	0.488	0.444	0.415	0.472	0.459	0.437	0.480
**Individual**		**Mean difference**	**LCI**	**UCI**	**Mean difference**	**LCI**	**UCI**	**Mean difference**	**LCI**	**UCI**	**Mean difference**	**LCI**	**UCI**	**Mean difference**	**LCI**	**UCI**
Constant		0.318	0.257	0.379	0.324	0.268	0.379	0.439	0.387	0.492	0.421	0.366	0.475	0.442	0.393	0.491
Gender	Male	(ref)	(ref)	(ref)	(ref)	(ref)	(ref)	(ref)	(ref)	(ref)	(ref)	(ref)	(ref)	(ref)	(ref)	(ref)
	Female	-0.089	-0.132	-0.047	-0.124	-0.163	-0.084	-0.133	-0.171	-0.095	-0.109	-0.148	-0.071	-0.144	-0.183	-0.105
Ethnicity	White - British	(ref)	(ref)	(ref)	(ref)	(ref)	(ref)	(ref)	(ref)	(ref)	(ref)	(ref)	(ref)	(ref)	(ref)	(ref)
	Any other White background	0.028	-0.127	0.183	-0.030	-0.160	0.099	0.081	-0.041	0.203	0.027	-0.100	0.153	0.029	-0.091	0.149
	Asian or Asian British	-0.178	-0.481	0.125	-0.215	-0.440	0.011	-0.222	-0.491	0.047	-0.079	-0.343	0.186	-0.041	-0.274	0.192
	Mixed/Dual background	0.199	0.001	0.396	-0.032	-0.198	0.133	0.011	-0.141	0.163	-0.114	-0.257	0.029	-0.057	-0.212	0.098
	Any other ethnic group	0.030	-0.268	0.328	-0.017	-0.310	0.276	0.023	-0.208	0.255	0.197	-0.018	0.411	-0.141	-0.363	0.081
SES	IMD 2010	0.371	0.130	0.612	0.324	0.098	0.549	0.432	0.222	0.643	0.375	0.156	0.594	0.436	0.234	0.638
NCMP participation (non-participation >0.2)		-0.023	-0.096	0.050	-0.017	-0.087	0.054	0.011	-0.073	0.096	0.027	-0.056	0.109	0.042	-0.068	0.153
**Factor**		**Mean difference**	**LCI**	**UCI**	**Mean difference**	**LCI**	**UCI**	**Mean difference**	**LCI**	**UCI**	**Mean difference**	**LCI**	**UCI**	**Mean difference**	**LCI**	**UCI**
Constant		0.316	0.217	0.415	0.273	0.186	0.360	0.368	0.284	0.452	0.417	0.328	0.506	0.382	0.308	0.455
Gender	Male	(ref)	(ref)	(ref)	(ref)	(ref)	(ref)	(ref)	(ref)	(ref)	(ref)	(ref)	(ref)	(ref)	(ref)	(ref)
	Female	-0.089	-0.132	-0.047	-0.124	-0.163	-0.084	-0.133	-0.171	-0.095	-0.110	-0.148	-0.071	-0.144	-0.182	-0.105
Ethnicity	White - British	(ref)	(ref)	(ref)	(ref)	(ref)	(ref)	(ref)	(ref)	(ref)	(ref)	(ref)	(ref)	(ref)	(ref)	(ref)
	Any other White background	0.028	-0.127	0.183	-0.030	-0.160	0.099	0.081	-0.041	0.203	0.027	-0.100	0.153	0.027	-0.092	0.147
	Asian or Asian British	-0.178	-0.481	0.125	-0.219	-0.444	0.007	-0.229	-0.498	0.040	-0.079	-0.343	0.185	-0.052	-0.285	0.182
	Mixed/Dual background	0.199	0.001	0.396	-0.032	-0.198	0.134	0.012	-0.140	0.164	-0.114	-0.257	0.029	-0.059	-0.214	0.096
	Any other ethnic group	0.030	-0.269	0.328	-0.020	-0.313	0.272	0.017	-0.215	0.248	0.196	-0.018	0.411	-0.150	-0.372	0.072
SES	IMD 2010	0.370	0.124	0.616	0.285	0.054	0.516	0.380	0.164	0.596	0.372	0.148	0.596	0.369	0.159	0.579
NCMP participation (non-participation >0.2)		-0.023	-0.096	0.050	-0.014	-0.085	0.056	0.013	-0.071	0.098	0.026	-0.056	0.109	0.051	-0.059	0.162
Factor 1		0.003	-0.116	0.122	0.078	-0.024	0.180	0.109	0.008	0.210	0.007	-0.101	0.114	0.095	0.008	0.181
**Interaction**		**Mean difference**	**LCI**	**UCI**	**Mean difference**	**LCI**	**UCI**	**Mean difference**	**LCI**	**UCI**	**Mean difference**	**LCI**	**UCI**	**Mean difference**	**LCI**	**UCI**
Constant		0.225	0.098	0.351	0.226	0.109	0.343	0.349	0.237	0.461	0.381	0.264	0.497	0.352	0.248	0.456
Gender	Male	(ref)	(ref)	(ref)	(ref)	(ref)	(ref)	(ref)	(ref)	(ref)	(ref)	(ref)	(ref)	(ref)	(ref)	(ref)
	Female	-0.090	-0.132	-0.047	-0.123	-0.163	-0.083	-0.133	-0.171	-0.096	-0.109	-0.147	-0.071	-0.144	-0.182	-0.105
Ethnicity	White - British	(ref)	(ref)	(ref)	(ref)	(ref)	(ref)	(ref)	(ref)	(ref)	(ref)	(ref)	(ref)	(ref)	(ref)	(ref)
	Any other White background	0.044	-0.111	0.200	-0.023	-0.152	0.107	0.081	-0.041	0.203	0.029	-0.097	0.155	0.026	-0.094	0.146
	Asian or Asian British	-0.175	-0.478	0.128	-0.216	-0.442	0.009	-0.230	-0.499	0.039	-0.074	-0.339	0.190	-0.054	-0.287	0.180
	Mixed/Dual background	0.196	-0.002	0.393	-0.030	-0.196	0.135	0.011	-0.141	0.163	-0.110	-0.253	0.033	-0.059	-0.214	0.096
	Any other ethnic group	0.037	-0.262	0.335	-0.021	-0.314	0.272	0.018	-0.214	0.249	0.195	-0.019	0.409	-0.151	-0.373	0.071
SES	IMD 2010	0.386	0.140	0.632	0.285	0.054	0.516	0.379	0.163	0.595	0.370	0.146	0.594	0.367	0.157	0.578
NCMP participation (non-participation >0.2)		-0.025	-0.096	0.047	-0.020	-0.091	0.050	0.018	-0.068	0.103	0.036	-0.047	0.118	0.050	-0.062	0.161
Factor 1		0.050	-0.102	0.201	0.101	-0.041	0.242	0.144	0.007	0.280	0.007	-0.133	0.147	0.135	0.010	0.260
Year group	Reception	(ref)	(ref)	(ref)	(ref)	(ref)	(ref)	(ref)	(ref)	(ref)	(ref)	(ref)	(ref)	(ref)	(ref)	(ref)
	Year 6	0.155	0.011	0.299	0.082	-0.064	0.228	0.037	-0.102	0.175	0.051	-0.086	0.188	0.054	-0.079	0.186
Year group*factor	Reception	(ref)	(ref)	(ref)	(ref)	(ref)	(ref)	(ref)	(ref)	(ref)	(ref)	(ref)	(ref)	(ref)	(ref)	(ref)
	Year 6	-0.078	-0.256	0.101	-0.035	-0.217	0.148	-0.067	-0.239	0.105	0.018	-0.151	0.186	-0.073	-0.235	0.089

Supporting file 3 to Williams, A.J., Wyatt, K.M., Williams, C.A., Logan, S., and Henley, W.E. Exploring the potential of a school impact on pupil weight status: exploratory factor analysis and repeat cross-sectional study of the National Child Measurement Programme.

S3 file - Detailed results and sensitivity analysis

Factor 2 - Location

Red shading indicates significance (p<0.05), Green shading indicates modles which had to be estimated in R

Overweight (UK90)

**Single level**		**2006/07**			**2007/08**			**2008/09**			**2009/10**			**2010/11**		
n		8747			10061			10149			10356			9604		
Log likelihood	**Null**	-3723.27			-4273.38			-4660.99			-4699.66			-4334.16		
	**Individual**	-3717.66			-4267.11			-4657.93			-4694.99			-4326.89		
	**Factor**	-3717.56			-4265.75			-4657.72			-4692.86			-4326.71		
**Individual**		**Odds Ratio**	**LCI**	**UCI**	**Odds Ratio**	**LCI**	**UCI**	**Odds Ratio**	**LCI**	**UCI**	**Odds Ratio**	**LCI**	**UCI**	**Odds Ratio**	**LCI**	**UCI**
Gender	Male	(ref)	(ref)	(ref)	(ref)	(ref)	(ref)	(ref)	(ref)	(ref)	(ref)	(ref)	(ref)	(ref)	(ref)	(ref)
	Female	0.880	0.783	0.990	0.897	0.804	1.001	0.933	0.842	1.035	0.920	0.830	1.020	0.856	0.769	0.953
Ethnicity	White - British	(ref)	(ref)	(ref)	(ref)	(ref)	(ref)	(ref)	(ref)	(ref)	(ref)	(ref)	(ref)	(ref)	(ref)	(ref)
	Any other White background	0.935	0.610	1.435	0.813	0.559	1.184	0.947	0.675	1.329	1.086	0.780	1.512	1.191	0.876	1.621
	Asian or Asian British	0.542	0.194	1.518	0.895	0.471	1.701	0.776	0.348	1.728	0.993	0.484	2.041	0.775	0.383	1.571
	Mixed/Dual background	0.998	0.563	1.767	0.733	0.441	1.220	0.816	0.523	1.275	1.011	0.688	1.484	0.868	0.555	1.357
	Any other ethnic group	1.197	0.556	2.577	0.908	0.382	2.156	0.571	0.260	1.254	0.871	0.470	1.614	1.102	0.616	1.970
SES	IMD 2010	1.873	1.055	3.324	1.888	1.095	3.256	1.163	0.698	1.936	1.719	1.033	2.858	1.356	0.792	2.320
NCMP participation (non-participation >0.2)		0.964	0.814	1.142	1.079	0.923	1.262	1.076	0.889	1.302	1.126	0.946	1.341	1.280	0.971	1.686
**Factor**		**Odds Ratio**	**LCI**	**UCI**	**Odds Ratio**	**LCI**	**UCI**	**Odds Ratio**	**LCI**	**UCI**	**Odds Ratio**	**LCI**	**UCI**	**Odds Ratio**	**LCI**	**UCI**
Gender	Male	(ref)	(ref)	(ref)	(ref)	(ref)	(ref)	(ref)	(ref)	(ref)	(ref)	(ref)	(ref)	(ref)	(ref)	(ref)
	Female	0.880	0.783	0.990	0.897	0.804	1.001	0.933	0.842	1.035	0.922	0.832	1.022	0.856	0.769	0.953
Ethnicity	White - British	(ref)	(ref)	(ref)	(ref)	(ref)	(ref)	(ref)	(ref)	(ref)	(ref)	(ref)	(ref)	(ref)	(ref)	(ref)
	Any other White background	0.935	0.609	1.434	0.813	0.558	1.183	0.947	0.675	1.330	1.098	0.788	1.529	1.188	0.873	1.616
	Asian or Asian British	0.546	0.195	1.528	0.876	0.461	1.667	0.783	0.351	1.744	1.028	0.500	2.113	0.767	0.378	1.555
	Mixed/Dual background	1.001	0.565	1.774	0.731	0.439	1.216	0.817	0.523	1.275	1.022	0.696	1.502	0.866	0.554	1.353
	Any other ethnic group	1.207	0.560	2.602	0.888	0.374	2.111	0.576	0.262	1.266	0.892	0.481	1.653	1.093	0.611	1.955
SES	IMD 2010	1.909	1.069	3.410	1.758	1.016	3.043	1.192	0.711	1.997	1.871	1.115	3.140	1.321	0.767	2.274
NCMP participation (non-participation >0.2)		0.961	0.811	1.140	1.090	0.932	1.275	1.072	0.886	1.298	1.123	0.943	1.338	1.292	0.978	1.705
Factor 2		0.993	0.963	1.023	1.024	0.995	1.054	0.991	0.965	1.018	0.973	0.948	0.998	1.009	0.980	1.038

**Reception two-level**		**2006/07**			**2007/08**			**2008/09**			**2009/10**			**2010/11**		
n		4002			4769			4889			4908			4520		
Log likelihood	**Null**	-1621.95			-1944.48			-2250.55			-2162.02			-2008.25		
	**Individual**	-1619.64			-1932.20			-2246.54			-2158.56			-2003.00		
	**Factor**	-1619.63			-1932.18			-2246.26			-2156.92			-2002.63		
School variance	**Null**	0.084	0.024	0.286	0.012	4.22E-05	3.544	0.088	0.039	0.200	0.027	0.002	0.300	0.018	4.06E-04	0.777
	**Individual**	0.080	0.022	0.288	1.64E-08	0	.	0.087	0.038	0.197	0.025	0.002	0.338	0.016	2.79E-04	0.961
	**Factor**	0.080	0.022	0.290	7.00E-12	0	.	0.085	0.037	0.197	0.021	0.001	0.426	0.012	0.0000593	2.610
**Individual**		**Odds Ratio**	**LCI**	**UCI**	**Odds Ratio**	**LCI**	**UCI**	**Odds Ratio**	**LCI**	**UCI**	**Odds Ratio**	**LCI**	**UCI**	**Odds Ratio**	**LCI**	**UCI**
Gender	Male	(ref)	(ref)	(ref)	(ref)	(ref)	(ref)	(ref)	(ref)	(ref)	(ref)	(ref)	(ref)	(ref)	(ref)	(ref)
	Female	0.914	0.763	1.095	0.830	0.704	0.977	0.941	0.810	1.094	0.875	0.751	1.020	0.841	0.718	0.986
Ethnicity	White - British	(ref)	(ref)	(ref)	(ref)	(ref)	(ref)	(ref)	(ref)	(ref)	(ref)	(ref)	(ref)	(ref)	(ref)	(ref)
	Any other White background	0.709	0.383	1.310	0.639	0.378	1.078	0.801	0.486	1.322	1.365	0.885	2.105	1.147	0.758	1.735
	Asian or Asian British	0.913	0.264	3.154	0.406	0.125	1.321	0.373	0.086	1.610	0.563	0.170	1.867	0.671	0.260	1.735
	Mixed/Dual background	1.011	0.421	2.427	0.322	0.117	0.884	0.817	0.457	1.462	0.854	0.491	1.485	0.808	0.436	1.496
	Any other ethnic group	1.262	0.469	3.397	0.597	0.138	2.575	1.021	0.414	2.517	0.995	0.412	2.399	0.668	0.234	1.905
SES	IMD 2010	1.336	0.535	3.337	2.754	1.257	6.038	1.534	0.706	3.332	1.407	0.659	3.006	1.493	0.672	3.319
NCMP participation (non-participation >0.2)		0.819	0.604	1.112	0.841	0.639	1.107	1.412	0.945	2.109	0.937	0.727	1.207	1.724	0.927	3.208
**Factor**		**Odds Ratio**	**LCI**	**UCI**	**Odds Ratio**	**LCI**	**UCI**	**Odds Ratio**	**LCI**	**UCI**	**Odds Ratio**	**LCI**	**UCI**	**Odds Ratio**	**LCI**	**UCI**
Gender	Male	(ref)	(ref)	(ref)	(ref)	(ref)	(ref)	(ref)	(ref)	(ref)	(ref)	(ref)	(ref)	(ref)	(ref)	(ref)
	Female	0.914	0.763	1.095	0.830	0.704	0.978	0.941	0.810	1.093	0.877	0.752	1.022	0.842	0.718	0.987
Ethnicity	White - British	(ref)	(ref)	(ref)	(ref)	(ref)	(ref)	(ref)	(ref)	(ref)	(ref)	(ref)	(ref)	(ref)	(ref)	(ref)
	Any other White background	0.709	0.383	1.310	0.639	0.378	1.078	0.801	0.486	1.322	1.382	0.896	2.131	1.136	0.750	1.719
	Asian or Asian British	0.911	0.264	3.150	0.405	0.124	1.317	0.368	0.085	1.590	0.587	0.177	1.949	0.657	0.254	1.699
	Mixed/Dual background	1.009	0.420	2.424	0.322	0.117	0.884	0.817	0.457	1.460	0.864	0.497	1.502	0.805	0.435	1.491
	Any other ethnic group	1.259	0.467	3.392	0.594	0.138	2.565	1.008	0.409	2.486	1.043	0.432	2.518	0.654	0.229	1.865
SES	IMD 2010	1.329	0.530	3.335	2.710	1.219	6.023	1.472	0.674	3.215	1.565	0.724	3.381	1.409	0.630	3.150
NCMP participation (non-participation >0.2)		0.820	0.604	1.114	0.843	0.640	1.111	1.431	0.957	2.141	0.949	0.737	1.221	1.779	0.954	3.319
Factor 2		1.003	0.954	1.054	1.005	0.962	1.049	1.017	0.973	1.062	0.963	0.925	1.003	1.019	0.976	1.064

**Year 6 two-level**		**2006/07**			**2007/08**			**2008/09**			**2009/10**			**2010/11**		
n		4745			5292			5260			5448			5084		
Log likelihood	**Null**	-2095.61			-2325.40			-2404.51			-2534.54			-2325.19		
	**Individual**	-2088.90			-2322.30			-2400.36			-2526.67			-2321.69		
	**Factor**	-2088.47			-2320.39			-2398.72			-2526.48			-2321.69		
School variance	**Null**	0.019	0.001	0.468	0.016	3.19E-04	0.763	0.043	0.009	0.200	0.026	0.003	0.216	5.17E-16	0	.
	**Individual**	0.004	1.10E-08	1830.554	0.009	9.25E-06	7.967	0.040	0.008	0.207	0.020	0.001	0.288	7.71E-19	0	.
	**Factor**	0.001	3.42E-31	2.34E+24	0.005	1.65E-07	182.656	0.041	0.008	0.200	0.019	0.001	0.336	6.55E-21	0	.
**Individual**		**Odds Ratio**	**LCI**	**UCI**	**Odds Ratio**	**LCI**	**UCI**	**Odds Ratio**	**LCI**	**UCI**	**Odds Ratio**	**LCI**	**UCI**	**Odds Ratio**	**LCI**	**UCI**
Gender	Male	(ref)	(ref)	(ref)	(ref)	(ref)	(ref)	(ref)	(ref)	(ref)	(ref)	(ref)	(ref)	(ref)	(ref)	(ref)
	Female	0.851	0.729	0.994	0.951	0.821	1.102	0.926	0.802	1.070	0.957	0.832	1.102	0.869	0.751	1.006
Ethnicity	White - British	(ref)	(ref)	(ref)	(ref)	(ref)	(ref)	(ref)	(ref)	(ref)	(ref)	(ref)	(ref)	(ref)	(ref)	(ref)
	Any other White background	1.418	0.764	2.632	1.154	0.668	1.994	1.099	0.685	1.763	0.842	0.499	1.423	1.269	0.797	2.021
	Asian or Asian British	0.241	0.032	1.791	1.604	0.721	3.570	1.232	0.458	3.317	1.633	0.638	4.182	0.897	0.308	2.613
	Mixed/Dual background	1.001	0.467	2.144	1.233	0.670	2.268	0.755	0.371	1.537	1.210	0.702	2.085	0.958	0.499	1.839
	Any other ethnic group	1.113	0.318	3.891	1.198	0.403	3.558	0.159	0.022	1.167	0.781	0.327	1.866	1.497	0.735	3.051
SES	IMD 2010	2.488	1.141	5.426	1.398	0.646	3.023	0.865	0.406	1.845	2.322	1.121	4.810	1.247	0.596	2.610
NCMP participation (non-participation >0.2)		1.047	0.845	1.296	1.199	0.986	1.459	0.977	0.768	1.242	1.487	1.143	1.933	1.172	0.858	1.602
**Factor**		**Odds Ratio**	**LCI**	**UCI**	**Odds Ratio**	**LCI**	**UCI**	**Odds Ratio**	**LCI**	**UCI**	**Odds Ratio**	**LCI**	**UCI**	**Odds Ratio**	**LCI**	**UCI**
Gender	Male	(ref)	(ref)	(ref)	(ref)	(ref)	(ref)	(ref)	(ref)	(ref)	(ref)	(ref)	(ref)	(ref)	(ref)	(ref)
	Female	0.850	0.728	0.993	0.951	0.820	1.102	0.925	0.800	1.069	0.958	0.833	1.102	0.869	0.751	1.006
Ethnicity	White - British	(ref)	(ref)	(ref)	(ref)	(ref)	(ref)	(ref)	(ref)	(ref)	(ref)	(ref)	(ref)	(ref)	(ref)	(ref)
	Any other White background	1.411	0.761	2.618	1.151	0.666	1.988	1.103	0.687	1.770	0.845	0.500	1.428	1.269	0.796	2.021
	Asian or Asian British	0.244	0.033	1.815	1.557	0.699	3.466	1.274	0.473	3.431	1.656	0.646	4.241	0.896	0.307	2.615
	Mixed/Dual background	1.004	0.469	2.150	1.224	0.665	2.251	0.753	0.370	1.534	1.218	0.706	2.099	0.958	0.499	1.839
	Any other ethnic group	1.134	0.324	3.966	1.162	0.391	3.450	0.163	0.022	1.203	0.784	0.328	1.872	1.497	0.734	3.051
SES	IMD 2010	2.644	1.200	5.826	1.278	0.593	2.752	0.937	0.435	2.018	2.392	1.147	4.988	1.246	0.590	2.628
NCMP participation (non-participation >0.2)		1.041	0.841	1.288	1.212	0.997	1.474	0.971	0.763	1.234	1.473	1.132	1.918	1.173	0.856	1.606
Factor 2		0.981	0.941	1.022	1.039	1.000	1.081	0.964	0.927	1.003	0.989	0.952	1.026	1.000	0.962	1.040

**Three-level**		**2006/07**			**2007/08**			**2008/09**			**2009/10**			**2010/11**		
n		8747			10061			10149			10356			9604		
Log likelihood	**Null**	-3721.38			-4272.82			-4655.48			-4698.53			-4334.14		
	**Individual**	-3716.23			-4266.83			-4652.40			-4693.85			-4326.84		
	**Factor**	-3716.14			-4265.52			-4652.06			-4692.01			-4326.68		
	**Interaction**	-3712.41			-4262.17			-4650.36			-4689.30			-4326.15		
School variance	**Null**	2.31E-11	0	.	0.006	2.30E-06	16.533	0.001	7.92E-22	1.86E+15	0.003	3.68E-10	21371.7	0.003		
	**Individual**	5.24E-18	0	.	0.001	2.70E-24	3.73E+17	9.97E-09	0	.	0.003	1.66E-09	5768.52	0.005	8.60E-06	2.605
	**Factor**	5.07E-16	0	.	0.001	5.48E-43	5.12E+35	2.51E-07			1.32E-05			0.004	1.50E-06	9.611
	**Interaction**	1.42E-13	0	.	0.007	0.0000814	0.670	0.003	3.77E-11	206249.7	0.005	1.54E-06	18.307	0.004	1.52E-06	9.551
Year group variance	**Null**	0.048	0.015	0.154	0.017	3.49E-04	0.813	0.066	0.023	0.190	0.026	0.002	0.304	9.93E-12		
	**Individual**	0.042	0.012	0.156	0.016	2.63E-04	0.943	0.067	0.033	0.138	0.026	0.002	0.305	7.28E-14	0	.
	**Factor**	0.042	0.011	0.156	0.015	1.68E-04	1.254	0.068			0.026			1.89E-14	0	.
	**Interaction**	0.032	0.006	0.170	9.37E-08	0	.	0.063	0.021	0.189	0.017	4.27E-04	0.666	1.70E-14	0	.
**Individual**		**Odds Ratio**	**LCI**	**UCI**	**Odds Ratio**	**LCI**	**UCI**	**Odds Ratio**	**LCI**	**UCI**	**Odds Ratio**	**LCI**	**UCI**	**Odds Ratio**	**LCI**	**UCI**
Gender	Male	(ref)	(ref)	(ref)	(ref)	(ref)	(ref)	(ref)	(ref)	(ref)	(ref)	(ref)	(ref)	(ref)	(ref)	(ref)
	Female	0.880	0.782	0.990	0.897	0.804	1.001	0.933	0.841	1.035	0.921	0.831	1.022	0.856	0.768	0.953
Ethnicity	White - British	(ref)	(ref)	(ref)	(ref)	(ref)	(ref)	(ref)	(ref)	(ref)	(ref)	(ref)	(ref)	(ref)	(ref)	(ref)
	Any other White background	0.934	0.607	1.438	0.817	0.560	1.191	0.935	0.663	1.318	1.096	0.785	1.529	1.193	0.876	1.623
	Asian or Asian British	0.540	0.192	1.518	0.894	0.470	1.703	0.762	0.339	1.710	0.998	0.484	2.056	0.773	0.381	1.567
	Mixed/Dual background	0.992	0.558	1.762	0.734	0.441	1.222	0.799	0.510	1.253	1.011	0.687	1.487	0.867	0.555	1.356
	Any other ethnic group	1.190	0.549	2.580	0.904	0.380	2.153	0.569	0.258	1.257	0.882	0.474	1.638	1.103	0.616	1.973
SES	IMD 2010	1.789	0.984	3.253	1.873	1.074	3.267	1.150	0.668	1.979	1.764	1.040	2.991	1.357	0.789	2.333
NCMP participation (non-participation >0.2)		0.959	0.802	1.147	1.079	0.919	1.266	1.073	0.872	1.321	1.132	0.943	1.360	1.285	0.972	1.698
**Factor**		**Odds Ratio**	**LCI**	**UCI**	**Odds Ratio**	**LCI**	**UCI**	**Odds Ratio**	**LCI**	**UCI**	**Odds Ratio**	**LCI**	**UCI**	**Odds Ratio**	**LCI**	**UCI**
Gender	Male	(ref)	(ref)	(ref)	(ref)	(ref)	(ref)	(ref)	(ref)	(ref)	(ref)	(ref)	(ref)	(ref)	(ref)	(ref)
	Female	0.880	0.782	0.989	0.897	0.804	1.001	0.933	0.840	1.035	0.923	0.832	1.023	0.856	0.769	0.953
Ethnicity	White - British	(ref)	(ref)	(ref)	(ref)	(ref)	(ref)	(ref)	(ref)	(ref)	(ref)	(ref)	(ref)	(ref)	(ref)	(ref)
	Any other White background	0.934	0.607	1.438	0.816	0.560	1.190	0.935	0.663	1.319	1.105	0.792	1.541	1.189	0.873	1.618
	Asian or Asian British	0.543	0.193	1.526	0.877	0.460	1.670	0.770	0.342	1.732	1.029	0.499	2.121	0.765	0.377	1.553
	Mixed/Dual background	0.995	0.560	1.768	0.732	0.439	1.218	0.800	0.510	1.254	1.022	0.694	1.504	0.865	0.553	1.353
	Any other ethnic group	1.199	0.552	2.601	0.886	0.372	2.109	0.575	0.259	1.273	0.898	0.483	1.670	1.094	0.611	1.959
SES	IMD 2010	1.820	0.995	3.328	1.750	1.002	3.059	1.183	0.684	2.049	1.898	1.115	3.233	1.323	0.766	2.288
NCMP participation (non-participation >0.2)		0.957	0.800	1.144	1.089	0.928	1.279	1.069	0.868	1.315	1.129	0.941	1.354	1.295	0.979	1.713
Factor 2		0.993	0.962	1.025	1.024	0.995	1.055	0.988	0.959	1.017	0.973	0.947	1.000	1.008	0.980	1.038
**Interaction**		**Odds Ratio**	**LCI**	**UCI**	**Odds Ratio**	**LCI**	**UCI**	**Odds Ratio**	**LCI**	**UCI**	**Odds Ratio**	**LCI**	**UCI**	**Odds Ratio**	**LCI**	**UCI**
Gender	Male	(ref)	(ref)	(ref)	(ref)	(ref)	(ref)	(ref)	(ref)	(ref)	(ref)	(ref)	(ref)	(ref)	(ref)	(ref)
	Female	0.878	0.781	0.988	0.898	0.805	1.002	0.931	0.839	1.034	0.923	0.832	1.024	0.856	0.769	0.954
Ethnicity	White - British	(ref)	(ref)	(ref)	(ref)	(ref)	(ref)	(ref)	(ref)	(ref)	(ref)	(ref)	(ref)	(ref)	(ref)	(ref)
	Any other White background	0.961	0.624	1.480	0.833	0.571	1.214	0.936	0.664	1.320	1.114	0.798	1.554	1.194	0.877	1.627
	Asian or Asian British	0.546	0.194	1.533	0.887	0.466	1.690	0.767	0.341	1.722	1.041	0.505	2.146	0.769	0.379	1.561
	Mixed/Dual background	0.993	0.559	1.764	0.737	0.443	1.227	0.797	0.509	1.250	1.031	0.701	1.518	0.870	0.556	1.361
	Any other ethnic group	1.223	0.564	2.651	0.895	0.376	2.128	0.573	0.260	1.267	0.905	0.487	1.681	1.092	0.610	1.955
SES	IMD 2010	1.887	1.035	3.441	1.769	1.013	3.089	1.172	0.677	2.030	1.909	1.120	3.255	1.331	0.770	2.301
NCMP participation (non-participation >0.2)		0.949	0.795	1.133	1.073	0.915	1.258	1.073	0.870	1.324	1.156	0.963	1.387	1.272	0.959	1.686
Factor 2		1.006	0.960	1.054	1.011	0.968	1.055	1.016	0.974	1.060	0.960	0.922	0.999	1.016	0.974	1.060
Year group	Reception	(ref)	(ref)	(ref)	(ref)	(ref)	(ref)	(ref)	(ref)	(ref)	(ref)	(ref)	(ref)	(ref)	(ref)	(ref)
	Year 6	1.355	0.917	2.002	0.983	0.688	1.403	1.357	0.952	1.934	0.948	0.680	1.321	1.145	0.804	1.631
Year group*factor	Reception	(ref)	(ref)	(ref)	(ref)	(ref)	(ref)	(ref)	(ref)	(ref)	(ref)	(ref)	(ref)	(ref)	(ref)	(ref)
	Year 6	0.977	0.918	1.041	1.027	0.970	1.087	0.947	0.894	1.003	1.029	0.975	1.086	0.986	0.932	1.043

Supporting file 3 to Williams, A.J., Wyatt, K.M., Williams, C.A., Logan, S., and Henley, W.E. Exploring the potential of a school impact on pupil weight status: exploratory factor analysis and repeat cross-sectional study of the National Child Measurement Programme.

S3 file - Detailed results and sensitivity analysis

Factor 2 - Location

Red shading indicates significance (p<0.05), Green shading indicates modles which had to be estimated in R

Obese (UK90)

**Single level**		**2006/07**			**2007/08**			**2008/09**			**2009/10**			**2010/11**		
n		9910			11353			11696			11851			11016		
Log likelihood	**Null**	-3583.69			-4023.42			-4569.33			-4491.54			-4218.10		
	**Individual**	-3567.36			-4005.35			-4548.51			-4469.42			-4184.92		
	**Factor**	-3567.23			-4003.48			-4542.71			-4469.40			-4184.69		
**Individual**		**Odds Ratio**	**LCI**	**UCI**	**Odds Ratio**	**LCI**	**UCI**	**Odds Ratio**	**LCI**	**UCI**	**Odds Ratio**	**LCI**	**UCI**	**Odds Ratio**	**LCI**	**UCI**
Gender	Male	(ref)	(ref)	(ref)	(ref)	(ref)	(ref)	(ref)	(ref)	(ref)	(ref)	(ref)	(ref)	(ref)	(ref)	(ref)
	Female	0.807	0.714	0.913	0.765	0.680	0.860	0.780	0.700	0.869	0.839	0.752	0.935	0.711	0.635	0.797
Ethnicity	White - British	(ref)	(ref)	(ref)	(ref)	(ref)	(ref)	(ref)	(ref)	(ref)	(ref)	(ref)	(ref)	(ref)	(ref)	(ref)
	Any other White background	0.880	0.551	1.404	0.986	0.679	1.433	1.154	0.830	1.604	1.000	0.698	1.433	0.786	0.542	1.140
	Asian or Asian British	0.835	0.330	2.112	1.134	0.614	2.097	1.031	0.487	2.183	1.071	0.526	2.181	1.071	0.563	2.039
	Mixed/Dual background	1.962	1.246	3.090	0.960	0.586	1.573	1.049	0.685	1.607	0.942	0.622	1.426	0.949	0.604	1.490
	Any other ethnic group	1.179	0.530	2.625	1.630	0.791	3.359	1.355	0.757	2.426	1.285	0.737	2.240	0.618	0.284	1.344
SES	IMD 2010	2.893	1.611	5.196	2.992	1.704	5.256	3.052	1.847	5.043	3.977	2.371	6.669	4.059	2.394	6.880
NCMP participation (non-participation >0.2)		1.069	0.899	1.270	0.996	0.841	1.181	1.093	0.898	1.330	0.745	0.604	0.920	1.257	0.948	1.667
**Factor**		**Odds Ratio**	**LCI**	**UCI**	**Odds Ratio**	**LCI**	**UCI**	**Odds Ratio**	**LCI**	**UCI**	**Odds Ratio**	**LCI**	**UCI**	**Odds Ratio**	**LCI**	**UCI**
Gender	Male	(ref)	(ref)	(ref)	(ref)	(ref)	(ref)	(ref)	(ref)	(ref)	(ref)	(ref)	(ref)	(ref)	(ref)	(ref)
	Female	0.807	0.714	0.913	0.764	0.680	0.860	0.779	0.699	0.868	0.839	0.752	0.936	0.712	0.635	0.797
Ethnicity	White - British	(ref)	(ref)	(ref)	(ref)	(ref)	(ref)	(ref)	(ref)	(ref)	(ref)	(ref)	(ref)	(ref)	(ref)	(ref)
	Any other White background	0.880	0.551	1.404	0.983	0.677	1.428	1.152	0.829	1.602	1.002	0.699	1.435	0.783	0.539	1.136
	Asian or Asian British	0.842	0.333	2.129	1.105	0.597	2.044	0.983	0.464	2.084	1.074	0.527	2.190	1.057	0.555	2.014
	Mixed/Dual background	1.970	1.251	3.103	0.957	0.584	1.569	1.045	0.682	1.601	0.943	0.622	1.428	0.946	0.602	1.486
	Any other ethnic group	1.190	0.534	2.651	1.589	0.771	3.275	1.293	0.722	2.316	1.288	0.738	2.247	0.613	0.282	1.332
SES	IMD 2010	2.968	1.638	5.380	2.717	1.539	4.795	2.615	1.577	4.335	4.017	2.372	6.804	3.918	2.290	6.701
NCMP participation (non-participation >0.2)		1.065	0.896	1.266	1.009	0.851	1.196	1.110	0.912	1.351	0.745	0.603	0.920	1.270	0.957	1.687
Factor 2		0.992	0.961	1.024	1.031	0.999	1.063	1.051	1.021	1.081	0.997	0.969	1.026	1.010	0.981	1.041

**Reception two-level**		**2006/07**			**2007/08**			**2008/09**			**2009/10**			**2010/11**		
n		4352			5190			5421			5373			4970		
Log likelihood	**Null**	-1217.69			-1459.60			-1732.98			-1560.98			-1507.99		
	**Individual**	-1210.86			-1447.28			-1727.23			-1551.17			-1497.76		
	**Factor**	-1210.86			-1446.94			-1721.88			-1550.65			-1497.73		
School variance	**Null**	3.47E-20	0 .		0.091	0.023	0.364	0.145	0.071	0.298	0.305	0.175	0.530	0.106	0.032	0.351
	**Individual**	8.49E-13	0 .		0.049	0.004	0.568	0.136	0.064	0.288	0.235	0.123	0.450	0.105	0.032	0.338
	**Factor**	2.97E-15	0 .		0.046	0.003	0.645	0.111	0.046	0.264	0.231	0.119	0.448	0.104	0.032	0.336
**Individual**		**Odds Ratio**	**LCI**	**UCI**	**Odds Ratio**	**LCI**	**UCI**	**Odds Ratio**	**LCI**	**UCI**	**Odds Ratio**	**LCI**	**UCI**	**Odds Ratio**	**LCI**	**UCI**
Gender	Male	(ref)	(ref)	(ref)	(ref)	(ref)	(ref)	(ref)	(ref)	(ref)	(ref)	(ref)	(ref)	(ref)	(ref)	(ref)
	Female	0.824	0.661	1.027	0.821	0.671	1.005	0.797	0.664	0.956	0.764	0.628	0.930	0.703	0.577	0.858
Ethnicity	White - British	(ref)	(ref)	(ref)	(ref)	(ref)	(ref)	(ref)	(ref)	(ref)	(ref)	(ref)	(ref)	(ref)	(ref)	(ref)
	Any other White background	1.028	0.533	1.985	1.299	0.791	2.133	1.151	0.672	1.971	0.665	0.331	1.338	1.077	0.640	1.812
	Asian or Asian British	1.996	0.680	5.860	0.897	0.315	2.553	0.626	0.144	2.717	1.477	0.556	3.927	0.919	0.322	2.618
	Mixed/Dual background	1.872	0.834	4.203	0.443	0.139	1.415	1.082	0.569	2.056	1.064	0.560	2.020	1.135	0.581	2.217
	Any other ethnic group	0.405	0.055	2.989	0.495	0.066	3.733	1.091	0.378	3.150	0.758	0.227	2.533	1.222	0.426	3.502
SES	IMD 2010	3.599	1.308	9.900	7.312	2.861	18.687	2.478	1.010	6.082	4.185	1.559	11.238	3.945	1.511	10.298
NCMP participation (non-participation >0.2)		1.010	0.726	1.405	0.843	0.592	1.199	1.276	0.781	2.083	0.702	0.467	1.057	1.236	0.537	2.842
**Factor**		**Odds Ratio**	**LCI**	**UCI**	**Odds Ratio**	**LCI**	**UCI**	**Odds Ratio**	**LCI**	**UCI**	**Odds Ratio**	**LCI**	**UCI**	**Odds Ratio**	**LCI**	**UCI**
Gender	Male	(ref)	(ref)	(ref)	(ref)	(ref)	(ref)	(ref)	(ref)	(ref)	(ref)	(ref)	(ref)	(ref)	(ref)	(ref)
	Female	0.824	0.661	1.027	0.822	0.671	1.006	0.795	0.662	0.954	0.763	0.627	0.928	0.703	0.577	0.858
Ethnicity	White - British	(ref)	(ref)	(ref)	(ref)	(ref)	(ref)	(ref)	(ref)	(ref)	(ref)	(ref)	(ref)	(ref)	(ref)	(ref)
	Any other White background	1.028	0.533	1.985	1.297	0.790	2.129	1.153	0.674	1.973	0.659	0.328	1.326	1.079	0.641	1.817
	Asian or Asian British	1.998	0.680	5.874	0.883	0.310	2.512	0.593	0.137	2.567	1.439	0.541	3.829	0.926	0.324	2.645
	Mixed/Dual background	1.874	0.833	4.215	0.442	0.138	1.414	1.077	0.567	2.047	1.057	0.557	2.007	1.136	0.581	2.219
	Any other ethnic group	0.405	0.055	2.997	0.486	0.064	3.667	1.025	0.356	2.955	0.738	0.221	2.465	1.231	0.429	3.533
SES	IMD 2010	3.611	1.292	10.091	6.712	2.585	17.431	1.951	0.801	4.751	3.868	1.432	10.451	4.034	1.518	10.717
NCMP participation (non-participation >0.2)		1.009	0.724	1.406	0.854	0.600	1.216	1.372	0.842	2.236	0.697	0.462	1.049	1.221	0.528	2.823
Factor 2		0.999	0.945	1.056	1.024	0.968	1.082	1.097	1.037	1.159	1.032	0.971	1.097	0.993	0.939	1.050

**Year 6 two-level**		**2006/07**			**2007/08**			**2008/09**			**2009/10**			**2010/11**		
n		5558			6163			6275			6478			6046		
Log likelihood	**Null**	-2312.74			-2506.79			-2771.22			-2833.62			-2648.44		
	**Individual**	-2299.05			-2494.93			-2756.94			-2825.60			-2624.68		
	**Factor**	-2298.90			-2493.67			-2755.46			-2825.58			-2624.17		
School variance	**Null**	0.027	0.003	0.261	0.089	0.037	0.218	0.092	0.043	0.197	0.074	0.030	0.179	0.033	0.006	0.173
	**Individual**	0.014	2.27E-04	0.906	0.086	0.034	0.217	0.075	0.032	0.177	0.066	0.025	0.174	0.005	3.68E-07	71.441
	**Factor**	0.012	1.00E-04	1.512	0.078	0.029	0.215	0.074	0.031	0.175	0.066	0.025	0.174	0.004	2.21E-08	731.573
**Individual**		**Odds Ratio**	**LCI**	**UCI**	**Odds Ratio**	**LCI**	**UCI**	**Odds Ratio**	**LCI**	**UCI**	**Odds Ratio**	**LCI**	**UCI**	**Odds Ratio**	**LCI**	**UCI**
Gender	Male	(ref)	(ref)	(ref)	(ref)	(ref)	(ref)	(ref)	(ref)	(ref)	(ref)	(ref)	(ref)	(ref)	(ref)	(ref)
	Female	0.794	0.683	0.923	0.737	0.636	0.854	0.778	0.679	0.893	0.886	0.774	1.014	0.717	0.623	0.826
Ethnicity	White - British	(ref)	(ref)	(ref)	(ref)	(ref)	(ref)	(ref)	(ref)	(ref)	(ref)	(ref)	(ref)	(ref)	(ref)	(ref)
	Any other White background	0.986	0.502	1.935	0.881	0.487	1.594	1.201	0.782	1.846	1.288	0.831	1.997	0.683	0.395	1.178
	Asian or Asian British	0.266	0.036	1.977	1.319	0.599	2.908	1.213	0.490	3.000	0.897	0.306	2.628	1.351	0.581	3.140
	Mixed/Dual background	2.028	1.160	3.545	1.359	0.768	2.406	1.106	0.614	1.992	1.001	0.570	1.756	0.944	0.507	1.759
	Any other ethnic group	2.111	0.826	5.395	2.222	0.970	5.088	1.588	0.769	3.282	1.571	0.816	3.024	0.367	0.113	1.188
SES	IMD 2010	2.999	1.428	6.296	1.766	0.804	3.879	3.418	1.709	6.835	3.201	1.569	6.532	4.870	2.500	9.487
NCMP participation (non-participation >0.2)		1.030	0.836	1.270	0.936	0.746	1.173	0.870	0.680	1.113	1.007	0.754	1.346	1.041	0.766	1.415
**Factor**		**Odds Ratio**	**LCI**	**UCI**	**Odds Ratio**	**LCI**	**UCI**	**Odds Ratio**	**LCI**	**UCI**	**Odds Ratio**	**LCI**	**UCI**	**Odds Ratio**	**LCI**	**UCI**
Gender	Male	(ref)	(ref)	(ref)	(ref)	(ref)	(ref)	(ref)	(ref)	(ref)	(ref)	(ref)	(ref)	(ref)	(ref)	(ref)
	Female	0.794	0.683	0.923	0.737	0.636	0.854	0.778	0.678	0.892	0.887	0.775	1.015	0.717	0.623	0.826
Ethnicity	White - British	(ref)	(ref)	(ref)	(ref)	(ref)	(ref)	(ref)	(ref)	(ref)	(ref)	(ref)	(ref)	(ref)	(ref)	(ref)
	Any other White background	0.984	0.501	1.933	0.878	0.486	1.588	1.197	0.779	1.839	1.289	0.831	2.000	0.679	0.393	1.172
	Asian or Asian British	0.268	0.036	1.992	1.292	0.586	2.847	1.182	0.478	2.926	0.900	0.307	2.641	1.315	0.565	3.063
	Mixed/Dual background	2.030	1.162	3.547	1.352	0.764	2.393	1.105	0.614	1.991	1.002	0.571	1.759	0.938	0.503	1.747
	Any other ethnic group	2.128	0.832	5.441	2.177	0.951	4.984	1.542	0.746	3.188	1.574	0.817	3.031	0.363	0.112	1.174
SES	IMD 2010	3.104	1.462	6.587	1.653	0.755	3.617	3.130	1.560	6.279	3.239	1.575	6.661	4.581	2.336	8.985
NCMP participation (non-participation >0.2)		1.028	0.834	1.266	0.945	0.755	1.184	0.872	0.681	1.115	1.004	0.750	1.344	1.058	0.777	1.440
Factor 2		0.989	0.949	1.030	1.035	0.992	1.079	1.036	0.995	1.078	0.996	0.958	1.035	1.019	0.982	1.058

**Three-level**		**2006/07**			**2007/08**			**2008/09**			**2009/10**			**2010/11**		
n		9910			11353			11696			11851			11016		
Log likelihood	**Null**	-3574.27			-4002.79			-4542.37			-4448.49			-4200.78		
	**Individual**	-3559.41			-3986.74			-4524.94			-4435.32			-4171.06		
	**Factor**	-3559.38			-3985.84			-4520.78			-4435.30			-4170.92		
	**Interaction**	-3512.67			-3945.79			-4479.08			-4382.43			-4124.47		
School variance	**Null**	2.61E-18	0 .		2.56E-14	0 .		1.74E-13	0 .		2.13E-17	0 .		1.23E-14	0 .	
	**Individual**	2.45E-21	0 .		6.01E-11	0 .		8.58E-11	0 .		7.45E-20	0 .		1.42E-12	0 .	
	**Factor**	1.38E-14	0 .		4.20E-12	0 .		3.00E-16	0 .		8.21E-19	0 .		1.02E-12	0 .	
	**Interaction**	0.025	0.004	0.169	0.057	0.017	0.191	0.035	0.007	0.184	0.045	0.011	0.191	0.031	0.009	0.112
Year group varia	**Null**	0.122	0.066	0.226	0.191	0.123	0.296	0.182	0.122	0.273	0.261	0.183	0.372	0.152	0.095	0.245
	**Individual**	0.114	0.059	0.219	0.181	0.114	0.286	0.167	0.109	0.255	0.233	0.159	0.341	0.132	0.079	0.222
	**Factor**	0.113	0.059	0.218	0.177	0.111	0.283	0.160	0.104	0.248	0.233	0.159	0.341	0.132	0.079	0.223
	**Interaction**	4.74E-14	0 .		0.020	4.22E-04	0.928	0.054	0.015	0.201	0.073	0.023	0.229	6.23E-11	0 .	
**Individual**		**Odds Ratio**	**LCI**	**UCI**	**Odds Ratio**	**LCI**	**UCI**	**Odds Ratio**	**LCI**	**UCI**	**Odds Ratio**	**LCI**	**UCI**	**Odds Ratio**	**LCI**	**UCI**
Gender	Male	(ref)	(ref)	(ref)	(ref)	(ref)	(ref)	(ref)	(ref)	(ref)	(ref)	(ref)	(ref)	(ref)	(ref)	(ref)
	Female	0.800	0.706	0.906	0.758	0.673	0.854	0.783	0.702	0.874	0.839	0.751	0.938	0.707	0.630	0.793
Ethnicity	White - British	(ref)	(ref)	(ref)	(ref)	(ref)	(ref)	(ref)	(ref)	(ref)	(ref)	(ref)	(ref)	(ref)	(ref)	(ref)
	Any other White background	0.915	0.569	1.470	1.011	0.687	1.487	1.159	0.828	1.623	1.031	0.713	1.490	0.807	0.553	1.179
	Asian or Asian British	0.866	0.339	2.214	1.069	0.568	2.013	0.966	0.450	2.074	1.129	0.546	2.334	1.104	0.574	2.124
	Mixed/Dual background	1.991	1.254	3.159	0.983	0.595	1.623	1.066	0.691	1.645	1.014	0.664	1.548	0.965	0.610	1.524
	Any other ethnic group	1.242	0.550	2.806	1.543	0.735	3.241	1.392	0.767	2.526	1.313	0.741	2.327	0.621	0.283	1.361
SES	IMD 2010	2.669	1.413	5.042	2.756	1.471	5.167	2.895	1.646	5.094	3.216	1.762	5.871	3.856	2.156	6.895
NCMP participation (non-participation >0.2)		1.053	0.866	1.281	0.957	0.779	1.177	1.079	0.852	1.366	0.824	0.639	1.063	1.227	0.887	1.698
**Factor**		**Odds Ratio**	**LCI**	**UCI**	**Odds Ratio**	**LCI**	**UCI**	**Odds Ratio**	**LCI**	**UCI**	**Odds Ratio**	**LCI**	**UCI**	**Odds Ratio**	**LCI**	**UCI**
Gender	Male	(ref)	(ref)	(ref)	(ref)	(ref)	(ref)	(ref)	(ref)	(ref)	(ref)	(ref)	(ref)	(ref)	(ref)	(ref)
	Female	0.800	0.706	0.906	0.758	0.673	0.855	0.783	0.701	0.873	0.840	0.751	0.939	0.707	0.630	0.793
Ethnicity	White - British	(ref)	(ref)	(ref)	(ref)	(ref)	(ref)	(ref)	(ref)	(ref)	(ref)	(ref)	(ref)	(ref)	(ref)	(ref)
	Any other White background	0.914	0.569	1.470	1.008	0.685	1.483	1.157	0.826	1.619	1.032	0.714	1.492	0.805	0.552	1.176
	Asian or Asian British	0.869	0.340	2.222	1.053	0.559	1.983	0.934	0.435	2.006	1.131	0.547	2.340	1.094	0.568	2.106
	Mixed/Dual background	1.994	1.256	3.164	0.981	0.594	1.620	1.063	0.689	1.641	1.014	0.664	1.549	0.963	0.609	1.522
	Any other ethnic group	1.247	0.552	2.818	1.522	0.724	3.196	1.346	0.741	2.442	1.316	0.742	2.331	0.617	0.282	1.353
SES	IMD 2010	2.700	1.420	5.134	2.608	1.388	4.900	2.583	1.467	4.547	3.237	1.763	5.943	3.761	2.090	6.770
NCMP participation (non-participation >0.2)		1.051	0.864	1.279	0.966	0.786	1.188	1.093	0.863	1.384	0.824	0.639	1.062	1.237	0.893	1.715
Factor 2		0.995	0.960	1.032	1.025	0.988	1.063	1.052	1.016	1.089	0.997	0.962	1.034	1.009	0.975	1.045
**Interaction**		**Odds Ratio**	**LCI**	**UCI**	**Odds Ratio**	**LCI**	**UCI**	**Odds Ratio**	**LCI**	**UCI**	**Odds Ratio**	**LCI**	**UCI**	**Odds Ratio**	**LCI**	**UCI**
Gender	Male	(ref)	(ref)	(ref)	(ref)	(ref)	(ref)	(ref)	(ref)	(ref)	(ref)	(ref)	(ref)	(ref)	(ref)	(ref)
	Female	0.802	0.708	0.908	0.765	0.679	0.861	0.784	0.703	0.875	0.839	0.751	0.938	0.712	0.635	0.799
Ethnicity	White - British	(ref)	(ref)	(ref)	(ref)	(ref)	(ref)	(ref)	(ref)	(ref)	(ref)	(ref)	(ref)	(ref)	(ref)	(ref)
	Any other White background	1.011	0.631	1.622	1.119	0.763	1.640	1.180	0.844	1.650	1.046	0.725	1.510	0.855	0.587	1.245
	Asian or Asian British	0.900	0.353	2.291	1.106	0.589	2.076	0.948	0.443	2.031	1.182	0.572	2.441	1.166	0.607	2.240
	Mixed/Dual background	1.983	1.252	3.141	0.996	0.604	1.641	1.094	0.710	1.688	1.046	0.686	1.596	1.014	0.642	1.601
	Any other ethnic group	1.300	0.578	2.926	1.531	0.732	3.202	1.341	0.740	2.431	1.297	0.735	2.291	0.616	0.281	1.347
SES	IMD 2010	3.121	1.665	5.853	2.704	1.451	5.039	2.563	1.468	4.477	3.421	1.885	6.207	4.128	2.338	7.288
NCMP participation (non-participation >0.2)		1.028	0.859	1.231	0.920	0.761	1.111	0.936	0.747	1.171	0.850	0.671	1.078	1.077	0.800	1.449
Factor 2		1.001	0.946	1.059	1.031	0.975	1.090	1.090	1.033	1.150	1.033	0.977	1.092	0.992	0.942	1.045
Year group	Reception	(ref)	(ref)	(ref)	(ref)	(ref)	(ref)	(ref)	(ref)	(ref)	(ref)	(ref)	(ref)	(ref)	(ref)	(ref)
	Year 6	2.114	1.385	3.227	1.878	1.238	2.849	2.452	1.623	3.704	2.606	1.722	3.945	1.617	1.095	2.389
Year group*factor	Reception	(ref)	(ref)	(ref)	(ref)	(ref)	(ref)	(ref)	(ref)	(ref)	(ref)	(ref)	(ref)	(ref)	(ref)	(ref)
	Year 6	0.989	0.924	1.058	1.001	0.937	1.069	0.953	0.893	1.017	0.961	0.900	1.028	1.028	0.966	1.093

Supporting file 3 to Williams, A.J., Wyatt, K.M., Williams, C.A., Logan, S., and Henley, W.E. Exploring the potential of a school impact on pupil weight status: exploratory factor analysis and repeat cross-sectional study of the National Child Measurement Programme.

S3 file - Detailed results and sensitivity analysis

Factor 2 - Location

Red shading indicates significance (p<0.05), Green shading indicates modles which had to be estimated in R

Overweight (IOTF)

Single level

		2006/07			2007/08			2008/09			2009/10			2010/11		
n		9532			10955			11217			11345			10559		
Log likelihood	**Null**	-4160.76			-4786.96			-5421.59			-5292.83			-4916.86		
	**Individual**	-4143.36			-4767.80			-5401.96			-5271.96			-4908.28		
	**Factor**	-4143.32			-4764.22			-5401.56			-5269.54			-4907.48		
**Individual**		**Odds Ratio**	**LCI**	**UCI**	**Odds Ratio**	**LCI**	**UCI**	**Odds Ratio**	**LCI**	**UCI**	**Odds Ratio**	**LCI**	**UCI**	**Odds Ratio**	**LCI**	**UCI**
Gender	Male	(ref)	(ref)	(ref)	(ref)	(ref)	(ref)	(ref)	(ref)	(ref)	(ref)	(ref)	(ref)	(ref)	(ref)	(ref)
	Female	1.319	1.181	1.473	1.322	1.193	1.466	1.288	1.171	1.417	1.346	1.222	1.483	1.129	1.021	1.248
Ethnicity	White - British	(ref)	(ref)	(ref)	(ref)	(ref)	(ref)	(ref)	(ref)	(ref)	(ref)	(ref)	(ref)	(ref)	(ref)	(ref)
	Any other White background	1.047	0.705	1.554	0.807	0.563	1.157	1.115	0.830	1.499	1.071	0.781	1.467	1.089	0.808	1.467
	Asian or Asian British	0.738	0.312	1.744	0.779	0.411	1.475	0.910	0.458	1.809	0.964	0.486	1.912	0.761	0.388	1.490
	Mixed/Dual background	1.409	0.886	2.241	0.721	0.445	1.169	1.085	0.746	1.578	0.896	0.615	1.305	0.951	0.631	1.432
	Any other ethnic group	1.348	0.670	2.710	1.031	0.481	2.209	0.950	0.519	1.737	1.061	0.623	1.806	0.516	0.248	1.073
SES	IMD 2010	2.098	1.225	3.592	1.829	1.095	3.054	2.124	1.345	3.353	1.694	1.049	2.735	1.368	0.831	2.253
NCMP participation (non-participation >0.2)		1.071	0.917	1.250	1.077	0.930	1.247	1.099	0.923	1.308	0.963	0.810	1.144	1.358	1.058	1.743
**Factor**		**Odds Ratio**	**LCI**	**UCI**	**Odds Ratio**	**LCI**	**UCI**	**Odds Ratio**	**LCI**	**UCI**	**Odds Ratio**	**LCI**	**UCI**	**Odds Ratio**	**LCI**	**UCI**
Gender	Male	(ref)	(ref)	(ref)	(ref)	(ref)	(ref)	(ref)	(ref)	(ref)	(ref)	(ref)	(ref)	(ref)	(ref)	(ref)
	Female	1.319	1.181	1.473	1.323	1.194	1.467	1.288	1.171	1.417	1.349	1.224	1.486	1.129	1.021	1.248
Ethnicity	White - British	(ref)	(ref)	(ref)	(ref)	(ref)	(ref)	(ref)	(ref)	(ref)	(ref)	(ref)	(ref)	(ref)	(ref)	(ref)
	Any other White background	1.046	0.705	1.554	0.806	0.562	1.156	1.114	0.829	1.498	1.082	0.790	1.483	1.081	0.802	1.457
	Asian or Asian British	0.740	0.313	1.750	0.755	0.399	1.430	0.900	0.453	1.790	0.998	0.503	1.981	0.743	0.379	1.457
	Mixed/Dual background	1.412	0.888	2.246	0.719	0.444	1.165	1.085	0.746	1.577	0.904	0.621	1.318	0.947	0.629	1.427
	Any other ethnic group	1.354	0.673	2.726	0.999	0.466	2.142	0.940	0.514	1.721	1.089	0.639	1.854	0.507	0.244	1.056
SES	IMD 2010	2.121	1.231	3.655	1.640	0.979	2.745	2.053	1.294	3.259	1.845	1.133	3.004	1.300	0.785	2.151
NCMP participation (non-participation >0.2)		1.069	0.915	1.249	1.094	0.944	1.267	1.103	0.926	1.313	0.960	0.807	1.141	1.383	1.076	1.777
Factor 2		0.996	0.968	1.025	1.037	1.010	1.066	1.011	0.987	1.037	0.973	0.949	0.997	1.017	0.991	1.045

Reception two-level

		2006/07			2007/08			2008/09			2009/10			2010/11		
n		4214			5017			5199			5165			4783		
Log likelihood	**Null**	-1716.68			-1998.43			-2399.35			-2231.13			-2109.18		
	**Individual**	-1704.54			-1975.03			-2381.45			-2215.84			-2098.70		
	**Factor**	-1704.35			-1975.00			-2381.35			-2214.94			-2098.59		
School variance	**Null**	0.045	0.007	0.277	0.097	0.038	0.250	0.127	0.071	0.229	0.037	0.005	0.276	0.057	0.015	0.209
	**Individual**	0.030	0.002	0.452	0.080	0.027	0.242	0.120	0.065	0.220	0.036	0.005	0.285	0.065	0.020	0.210
	**Factor**	0.028	0.002	0.486	0.080	0.026	0.242	0.118	0.064	0.219	0.036	0.004	0.283	0.064	0.020	0.211
**Individual**		**Odds Ratio**	**LCI**	**UCI**	**Odds Ratio**	**LCI**	**UCI**	**Odds Ratio**	**LCI**	**UCI**	**Odds Ratio**	**LCI**	**UCI**	**Odds Ratio**	**LCI**	**UCI**
Gender	Male	(ref)	(ref)	(ref)	(ref)	(ref)	(ref)	(ref)	(ref)	(ref)	(ref)	(ref)	(ref)	(ref)	(ref)	(ref)
	Female	1.510	1.267	1.800	1.521	1.290	1.793	1.467	1.267	1.699	1.471	1.262	1.713	1.355	1.159	1.585
Ethnicity	White - British	(ref)	(ref)	(ref)	(ref)	(ref)	(ref)	(ref)	(ref)	(ref)	(ref)	(ref)	(ref)	(ref)	(ref)	(ref)
	Any other White background	0.913	0.525	1.589	0.704	0.418	1.183	0.835	0.515	1.353	1.174	0.746	1.846	1.338	0.899	1.992
	Asian or Asian British	1.150	0.385	3.431	0.266	0.063	1.112	0.676	0.226	2.019	0.578	0.174	1.920	0.529	0.185	1.508
	Mixed/Dual background	1.236	0.572	2.674	0.334	0.121	0.922	1.189	0.722	1.959	0.877	0.513	1.502	1.270	0.751	2.149
	Any other ethnic group	1.004	0.343	2.937	0.563	0.129	2.454	1.251	0.534	2.928	0.929	0.385	2.246	0.486	0.148	1.602
SES	IMD 2010	2.056	0.862	4.905	3.447	1.515	7.841	1.738	0.810	3.728	1.793	0.845	3.802	1.169	0.519	2.632
NCMP participation (non-participation >0.2)		1.005	0.767	1.317	0.917	0.687	1.224	1.633	1.106	2.409	0.830	0.636	1.082	1.347	0.691	2.629
**Factor**		**Odds Ratio**	**LCI**	**UCI**	**Odds Ratio**	**LCI**	**UCI**	**Odds Ratio**	**LCI**	**UCI**	**Odds Ratio**	**LCI**	**UCI**	**Odds Ratio**	**LCI**	**UCI**
Gender	Male	(ref)	(ref)	(ref)	(ref)	(ref)	(ref)	(ref)	(ref)	(ref)	(ref)	(ref)	(ref)	(ref)	(ref)	(ref)
	Female	1.510	1.266	1.799	1.521	1.291	1.793	1.466	1.266	1.698	1.473	1.264	1.717	1.355	1.159	1.585
Ethnicity	White - British	(ref)	(ref)	(ref)	(ref)	(ref)	(ref)	(ref)	(ref)	(ref)	(ref)	(ref)	(ref)	(ref)	(ref)	(ref)
	Any other White background	0.912	0.524	1.588	0.704	0.418	1.183	0.835	0.516	1.354	1.186	0.754	1.865	1.332	0.895	1.984
	Asian or Asian British	1.137	0.381	3.392	0.264	0.063	1.107	0.671	0.225	2.005	0.597	0.179	1.986	0.522	0.183	1.491
	Mixed/Dual background	1.220	0.564	2.641	0.334	0.121	0.922	1.189	0.721	1.958	0.883	0.516	1.512	1.269	0.750	2.146
	Any other ethnic group	0.986	0.337	2.888	0.561	0.129	2.445	1.243	0.531	2.911	0.964	0.398	2.333	0.481	0.146	1.587
SES	IMD 2010	1.988	0.831	4.758	3.383	1.469	7.788	1.699	0.788	3.661	1.942	0.905	4.169	1.136	0.501	2.576
NCMP participation (non-participation >0.2)		1.012	0.772	1.328	0.920	0.688	1.230	1.648	1.115	2.436	0.838	0.643	1.093	1.373	0.700	2.689
Factor 2		1.015	0.969	1.063	1.006	0.960	1.054	1.010	0.966	1.056	0.972	0.933	1.013	1.011	0.967	1.057

Year 6 two-level

		2006/07			2007/08			2008/09			2009/10			2010/11		
n		5318			5938			6018			6180			5776		
Log likelihood	**Null**	-2435.38			-2765.54			-3002.77			-3044.09			-2797.82		
	**Individual**	-2426.60			-2760.43			-2993.90			-3034.06			-2794.09		
	**Factor**	-2426.17			-2756.46			-2993.79			-3033.65			-2793.45		
School variance	**Null**	7.39E-16	0	.	0.075	0.029	0.196	0.051	0.017	0.155	0.049	0.016	0.153	0.038	0.009	0.160
	**Individual**	4.22E-16	0	.	0.071	0.026	0.194	0.050	0.016	0.152	0.046	0.014	0.153	0.031	0.005	0.172
	**Factor**	1.04E-16	0	.	0.053	0.015	0.191	0.050	0.016	0.152	0.043	0.012	0.154	0.029	0.005	0.175
**Individual**		**Odds Ratio**	**LCI**	**UCI**	**Odds Ratio**	**LCI**	**UCI**	**Odds Ratio**	**LCI**	**UCI**	**Odds Ratio**	**LCI**	**UCI**	**Odds Ratio**	**LCI**	**UCI**
Gender	Male	(ref)	(ref)	(ref)	(ref)	(ref)	(ref)	(ref)	(ref)	(ref)	(ref)	(ref)	(ref)	(ref)	(ref)	(ref)
	Female	1.202	1.042	1.387	1.219	1.065	1.395	1.185	1.043	1.346	1.274	1.122	1.447	0.996	0.872	1.137
Ethnicity	White - British	(ref)	(ref)	(ref)	(ref)	(ref)	(ref)	(ref)	(ref)	(ref)	(ref)	(ref)	(ref)	(ref)	(ref)	(ref)
	Any other White background	1.384	0.777	2.464	1.076	0.638	1.812	1.386	0.939	2.045	1.037	0.664	1.619	0.920	0.576	1.469
	Asian or Asian British	0.435	0.102	1.856	1.376	0.644	2.941	1.053	0.424	2.616	1.450	0.606	3.472	1.066	0.432	2.632
	Mixed/Dual background	1.533	0.854	2.752	1.109	0.625	1.969	0.942	0.522	1.698	0.964	0.564	1.648	0.691	0.352	1.358
	Any other ethnic group	1.905	0.736	4.933	1.369	0.542	3.456	0.765	0.316	1.852	1.176	0.596	2.322	0.544	0.213	1.388
SES	IMD 2010	2.182	1.074	4.435	1.025	0.485	2.163	2.423	1.264	4.645	1.758	0.888	3.480	1.538	0.775	3.052
NCMP participation (non-participation >0.2)		1.083	0.892	1.315	1.092	0.893	1.336	0.904	0.723	1.131	1.246	0.962	1.615	1.285	0.963	1.714
**Factor**		**Odds Ratio**	**LCI**	**UCI**	**Odds Ratio**	**LCI**	**UCI**	**Odds Ratio**	**LCI**	**UCI**	**Odds Ratio**	**LCI**	**UCI**	**Odds Ratio**	**LCI**	**UCI**
Gender	Male	(ref)	(ref)	(ref)	(ref)	(ref)	(ref)	(ref)	(ref)	(ref)	(ref)	(ref)	(ref)	(ref)	(ref)	(ref)
	Female	1.202	1.042	1.387	1.219	1.065	1.395	1.185	1.043	1.346	1.275	1.123	1.448	0.996	0.872	1.138
Ethnicity	White - British	(ref)	(ref)	(ref)	(ref)	(ref)	(ref)	(ref)	(ref)	(ref)	(ref)	(ref)	(ref)	(ref)	(ref)	(ref)
	Any other White background	1.379	0.775	2.456	1.070	0.635	1.801	1.384	0.938	2.043	1.041	0.666	1.625	0.915	0.573	1.461
	Asian or Asian British	0.441	0.103	1.882	1.331	0.624	2.840	1.047	0.421	2.600	1.475	0.616	3.532	1.038	0.420	2.566
	Mixed/Dual background	1.538	0.857	2.761	1.106	0.624	1.961	0.942	0.522	1.698	0.970	0.567	1.658	0.687	0.350	1.350
	Any other ethnic group	1.939	0.748	5.023	1.323	0.525	3.337	0.760	0.314	1.840	1.183	0.599	2.336	0.536	0.210	1.369
SES	IMD 2010	2.294	1.117	4.710	0.937	0.450	1.952	2.369	1.229	4.567	1.830	0.919	3.643	1.456	0.731	2.899
NCMP participation (non-participation >0.2)		1.078	0.888	1.309	1.111	0.913	1.354	0.905	0.724	1.132	1.230	0.948	1.594	1.308	0.979	1.746
Factor 2		0.982	0.946	1.020	1.058	1.017	1.099	1.009	0.973	1.046	0.984	0.950	1.019	1.022	0.984	1.060

Three-level

		2006/07			2007/08			2008/09			2009/10			2010/11		
n		9532			10955			11217			11345			10559		
Log likelihood	**Null**	-4160.65			-4774.86			-5407.81			-5287.50			-4912.78		
	**Individual**	-4143.36			-4756.93			-5388.58			-5266.85			-4904.63		
	**Factor**	-4143.32			-4754.68			-5388.44			-5265.06			-4904.02		
	**Interaction**	-4134.50			-4742.01			-5384.05			-5251.58			-4898.34		
School variance	**Null**	8.23E-11	0	.	1.53E-07	0	.	2.04E-10	0	.	0.017	0.001	0.314	0.009	4.04E-05	1.870
	**Individual**	4.56E-17	0	.	7.04E-10	0	.	2.13E-15	0	.	0.014	4.15E-04	0.475	0.014	4.61E-04	0.399
	**Factor**	2.03E-13	0	.	8.82E-15	0	.	4.28E-15	0	.	0.011	1.50E-04	0.84037	0.012	3.14E-04	0.494
	**Interaction**	6.07E-19	0	.	0.019	0.001	0.335	2.13E-08	0	.	0.033	0.008	0.133	0.021	0.003	0.178
Year group variai	**Null**	9.30E-03	1.07E-04	0.813	0.115	0.067	0.196	0.095	0.057	0.156	0.048	0.013	0.183	0.047	0.013	0.168
	**Individual**	2.04E-14	0	.	0.109	0.062	0.190	0.093	0.056	0.154	0.050	0.014	0.184	0.038	0.008	0.172
	**Factor**	4.02E-11	0	.	0.102	0.057	0.184	0.092	0.055	0.154	0.049	0.013	0.183	0.038	0.008	0.172
	**Interaction**	5.85E-19	0	.	0.050	0.012	0.212	0.088	0.052	0.148	0.009	2.94E-05	3.060	0.021	0.002	0.282
**Individual**		**Odds Ratio**	**LCI**	**UCI**	**Odds Ratio**	**LCI**	**UCI**	**Odds Ratio**	**LCI**	**UCI**	**Odds Ratio**	**LCI**	**UCI**	**Odds Ratio**	**LCI**	**UCI**
Gender	Male	(ref)	(ref)	(ref)	(ref)	(ref)	(ref)	(ref)	(ref)	(ref)	(ref)	(ref)	(ref)	(ref)	(ref)	(ref)
	Female	1.319	1.181	1.473	1.331	1.200	1.478	1.301	1.181	1.432	1.350	1.225	1.489	1.130	1.021	1.250
Ethnicity	White - British	(ref)	(ref)	(ref)	(ref)	(ref)	(ref)	(ref)	(ref)	(ref)	(ref)	(ref)	(ref)	(ref)	(ref)	(ref)
	Any other White background	1.047	0.705	1.554	0.834	0.577	1.205	1.107	0.819	1.496	1.098	0.799	1.511	1.105	0.817	1.494
	Asian or Asian British	0.738	0.312	1.744	0.769	0.403	1.469	0.874	0.435	1.757	0.965	0.483	1.925	0.743	0.377	1.463
	Mixed/Dual background	1.409	0.886	2.241	0.727	0.446	1.184	1.076	0.736	1.574	0.909	0.622	1.329	0.958	0.634	1.448
	Any other ethnic group	1.348	0.670	2.710	1.000	0.462	2.166	0.961	0.521	1.772	1.084	0.633	1.858	0.517	0.248	1.080
SES	IMD 2010	2.098	1.225	3.592	1.657	0.947	2.902	2.099	1.273	3.461	1.699	1.013	2.849	1.310	0.770	2.229
NCMP participation (non-participation >0.2)		1.071	0.917	1.250	1.067	0.900	1.266	1.096	0.899	1.338	0.991	0.819	1.198	1.370	1.045	1.797
**Factor**		**Odds Ratio**	**LCI**	**UCI**	**Odds Ratio**	**LCI**	**UCI**	**Odds Ratio**	**LCI**	**UCI**	**Odds Ratio**	**LCI**	**UCI**	**Odds Ratio**	**LCI**	**UCI**
Gender	Male	(ref)	(ref)	(ref)	(ref)	(ref)	(ref)	(ref)	(ref)	(ref)	(ref)	(ref)	(ref)	(ref)	(ref)	(ref)
	Female	1.319	1.181	1.473	1.332	1.200	1.478	1.300	1.181	1.432	1.352	1.227	1.491	1.130	1.021	1.250
Ethnicity	White - British	(ref)	(ref)	(ref)	(ref)	(ref)	(ref)	(ref)	(ref)	(ref)	(ref)	(ref)	(ref)	(ref)	(ref)	(ref)
	Any other White background	1.046	0.705	1.554	0.832	0.576	1.202	1.106	0.818	1.495	1.106	0.805	1.521	1.099	0.812	1.486
	Asian or Asian British	0.740	0.313	1.750	0.751	0.393	1.435	0.869	0.432	1.747	0.992	0.497	1.980	0.730	0.370	1.438
	Mixed/Dual background	1.412	0.888	2.246	0.725	0.445	1.182	1.076	0.736	1.573	0.916	0.627	1.339	0.955	0.632	1.444
	Any other ethnic group	1.354	0.673	2.726	0.980	0.453	2.123	0.955	0.518	1.763	1.105	0.645	1.894	0.510	0.244	1.066
SES	IMD 2010	2.121	1.231	3.655	1.541	0.881	2.697	2.061	1.245	3.410	1.819	1.080	3.064	1.258	0.737	2.147
NCMP participation (non-participation >0.2)		1.069	0.915	1.249	1.082	0.912	1.283	1.099	0.901	1.342	0.985	0.816	1.190	1.390	1.059	1.825
Factor 2		0.996	0.968	1.025	1.034	1.003	1.067	1.008	0.980	1.037	0.973	0.947	1.001	1.017	0.987	1.047
**Interaction**		**Odds Ratio**	**LCI**	**UCI**	**Odds Ratio**	**LCI**	**UCI**	**Odds Ratio**	**LCI**	**UCI**	**Odds Ratio**	**LCI**	**UCI**	**Odds Ratio**	**LCI**	**UCI**
Gender	Male	(ref)	(ref)	(ref)	(ref)	(ref)	(ref)	(ref)	(ref)	(ref)	(ref)	(ref)	(ref)	(ref)	(ref)	(ref)
	Female	1.317	1.179	1.471	1.335	1.203	1.481	1.301	1.182	1.433	1.354	1.228	1.492	1.132	1.023	1.252
Ethnicity	White - British	(ref)	(ref)	(ref)	(ref)	(ref)	(ref)	(ref)	(ref)	(ref)	(ref)	(ref)	(ref)	(ref)	(ref)	(ref)
	Any other White background	1.092	0.735	1.623	0.866	0.600	1.250	1.110	0.821	1.500	1.119	0.814	1.538	1.122	0.830	1.517
	Asian or Asian British	0.748	0.316	1.768	0.766	0.401	1.462	0.873	0.435	1.755	1.014	0.508	2.025	0.749	0.380	1.476
	Mixed/Dual background	1.403	0.882	2.232	0.732	0.450	1.192	1.090	0.745	1.593	0.935	0.640	1.366	0.974	0.645	1.472
	Any other ethnic group	1.384	0.687	2.788	0.992	0.458	2.145	0.955	0.517	1.761	1.097	0.640	1.879	0.509	0.244	1.064
SES	IMD 2010	2.207	1.280	3.806	1.590	0.910	2.778	2.063	1.248	3.411	1.822	1.081	3.072	1.279	0.750	2.182
NCMP participation (non-participation >0.2)		1.056	0.904	1.234	1.049	0.890	1.237	1.054	0.862	1.287	1.027	0.853	1.237	1.327	1.013	1.740
Factor 2		1.015	0.971	1.060	1.012	0.967	1.059	1.006	0.965	1.050	0.971	0.932	1.012	1.009	0.967	1.053
Year group	Reception	(ref)	(ref)	(ref)	(ref)	(ref)	(ref)	(ref)	(ref)	(ref)	(ref)	(ref)	(ref)	(ref)	(ref)	(ref)
	Year 6	1.525	1.065	2.185	1.058	0.736	1.519	1.157	0.819	1.635	1.248	0.909	1.715	1.119	0.794	1.577
Year group*factor	Reception	(ref)	(ref)	(ref)	(ref)	(ref)	(ref)	(ref)	(ref)	(ref)	(ref)	(ref)	(ref)	(ref)	(ref)	(ref)
	Year 6	0.968	0.914	1.026	1.043	0.983	1.106	1.005	0.949	1.063	1.009	0.959	1.062	1.012	0.958	1.070

Supporting file 3 to Williams, A.J., Wyatt, K.M., Williams, C.A., Logan, S., and Henley, W.E. Exploring the potential of a school impact on pupil weight status: exploratory factor analysis and repeat cross-sectional study of the National Child Measurement Programme.

S3 file - Detailed results and sensitivity analysis

Factor 2 - Location

Red shading indicates significance (p<0.05), Green shading indicates modles which had to be estimated in R

Obese (IOTF)

**Single level**		**2006/07**			**2007/08**			**2008/09**			**2009/10**			**2010/11**		
n		9910			11353			11696			11851			11016		
Log likelihood	**Null**	-1605.40			-1724.55			-1999.60			-2090.78			-1901.75		
	**Individual**	-1595.21			-1703.72			-1989.84			-2072.60			-1869.13		
	**Factor**	-1594.57			-1701.90			-1980.50			-2070.81			-1868.98		
**Individual**		**Odds Ratio**	**LCI**	**UCI**	**Odds Ratio**	**LCI**	**UCI**	**Odds Ratio**	**LCI**	**UCI**	**Odds Ratio**	**LCI**	**UCI**	**Odds Ratio**	**LCI**	**UCI**
Gender	Male	(ref)	(ref)	(ref)	(ref)	(ref)	(ref)	(ref)	(ref)	(ref)	(ref)	(ref)	(ref)	(ref)	(ref)	(ref)
	Female	1.066	0.868	1.310	1.207	0.987	1.475	1.255	1.045	1.508	1.220	1.020	1.459	1.308	1.083	1.580
Ethnicity	White - British	(ref)	(ref)	(ref)	(ref)	(ref)	(ref)	(ref)	(ref)	(ref)	(ref)	(ref)	(ref)	(ref)	(ref)	(ref)
	Any other White background	0.977	0.455	2.094	1.994	1.236	3.219	1.097	0.624	1.929	1.293	0.761	2.195	0.720	0.368	1.411
	Asian or Asian British	1.522	0.470	4.930	1.761	0.759	4.086	0.796	0.193	3.275	1.410	0.507	3.918	2.012	0.911	4.442
	Mixed/Dual background	1.619	0.747	3.504	1.083	0.476	2.466	1.078	0.527	2.205	0.752	0.352	1.609	1.467	0.768	2.804
	Any other ethnic group	1.503	0.465	4.855	2.289	0.819	6.401	2.249	1.027	4.925	1.443	0.627	3.323	1.456	0.586	3.621
SES	IMD 2010	7.403	2.939	18.646	7.276	2.922	18.121	3.863	1.682	8.873	9.268	4.174	20.580	19.715	8.886	43.742
NCMP participation (non-participation >0.2)		0.877	0.644	1.195	0.601	0.421	0.858	0.930	0.651	1.328	0.823	0.587	1.154	0.678	0.369	1.245
**Factor**		**Odds Ratio**	**LCI**	**UCI**	**Odds Ratio**	**LCI**	**UCI**	**Odds Ratio**	**LCI**	**UCI**	**Odds Ratio**	**LCI**	**UCI**	**Odds Ratio**	**LCI**	**UCI**
Gender	Male	(ref)	(ref)	(ref)	(ref)	(ref)	(ref)	(ref)	(ref)	(ref)	(ref)	(ref)	(ref)	(ref)	(ref)	(ref)
	Female	1.066	0.867	1.309	1.207	0.987	1.475	1.255	1.044	1.508	1.217	1.018	1.455	1.308	1.083	1.579
Ethnicity	White - British	(ref)	(ref)	(ref)	(ref)	(ref)	(ref)	(ref)	(ref)	(ref)	(ref)	(ref)	(ref)	(ref)	(ref)	(ref)
	Any other White background	0.977	0.456	2.095	1.985	1.230	3.205	1.095	0.622	1.927	1.270	0.748	2.157	0.724	0.369	1.418
	Asian or Asian British	1.565	0.483	5.076	1.695	0.730	3.935	0.724	0.176	2.981	1.341	0.482	3.731	2.044	0.923	4.523
	Mixed/Dual background	1.643	0.759	3.560	1.078	0.473	2.454	1.072	0.524	2.194	0.742	0.347	1.588	1.472	0.770	2.815
	Any other ethnic group	1.557	0.481	5.040	2.189	0.782	6.127	2.055	0.938	4.502	1.389	0.603	3.201	1.475	0.592	3.673
SES	IMD 2010	8.264	3.199	21.347	5.998	2.382	15.102	2.720	1.188	6.229	7.762	3.448	17.477	20.863	9.151	47.565
NCMP participation (non-participation >0.2)		0.868	0.637	1.183	0.614	0.430	0.877	0.961	0.673	1.373	0.828	0.590	1.160	0.669	0.363	1.231
Factor 2		0.970	0.920	1.022	1.054	0.998	1.113	1.119	1.061	1.179	1.047	0.998	1.099	0.986	0.938	1.037

**Reception two-level**		**2006/07**			**2007/08**			**2008/09**			**2009/10**			**2010/11**		
n		4352			5190			5421			5373			4970		
Log likelihood	**Null**	-612.05			-756.86			-925.76			-854.75			-796.40		
	**Individual**	-605.01			-742.63			-914.05			-848.53			-786.91		
	**Factor**	-604.92			-741.50			-906.30			-847.61			-786.80		
School variance	**Null**	7.88E-12	0	.	0.205	0.056	0.750	0.117	0.024	0.577	0.458	0.255	0.823	0.110	0.010	1.184
	**Individual**	3.72E-16	0	.	0.151			0.126			0.384	0.202	0.732	0.117	0.015	0.929
	**Factor**	6.47E-17	0	.	0.128			0.092			0.365	0.187	0.711	0.116	0.015	0.918
**Individual**		**Odds Ratio**	**LCI**	**UCI**	**Odds Ratio**	**LCI**	**UCI**	**Odds Ratio**	**LCI**	**UCI**	**Odds Ratio**	**LCI**	**UCI**	**Odds Ratio**	**LCI**	**UCI**
Gender	Male	(ref)	(ref)	(ref)	(ref)	(ref)	(ref)	(ref)	(ref)	(ref)	(ref)	(ref)	(ref)	(ref)	(ref)	(ref)
	Female	1.443	1.023	2.034	1.369	1.000	1.876	1.675	1.264	2.221	1.139	0.857	1.515	1.236	0.920	1.660
Ethnicity	White - British	(ref)	(ref)	(ref)	(ref)	(ref)	(ref)	(ref)	(ref)	(ref)	(ref)	(ref)	(ref)	(ref)	(ref)	(ref)
	Any other White background	1.375	0.551	3.428	2.273	1.224	4.222	1.339	0.634	2.826	1.149	0.513	2.574	0.951	0.412	2.197
	Asian or Asian British	3.585	1.053	12.208	1.743	0.511	5.943	3.52E-06	0	.	3.548	1.191	10.567	2.297	0.795	6.635
	Mixed/Dual background	1.999	0.613	6.520	7.40E-07	0.000	.	0.683	0.209	2.232	0.647	0.201	2.085	0.820	0.255	2.631
	Any other ethnic group	1.063	0.143	7.903	6.68E-07	0.000	.	1.423	0.327	6.199	1.260	0.291	5.457	2.223	0.668	7.398
SES	IMD 2010	5.261	1.155	23.970	6.738	1.563	29.048	4.295	1.217	15.162	6.086	1.539	24.074	12.443	3.260	47.488
NCMP participation (non-participation >0.2)		0.861	0.499	1.484	0.558	0.289	1.077	0.726	0.304	1.736	0.782	0.437	1.398	0.822	0.192	3.519
**Factor**		**Odds Ratio**	**LCI**	**UCI**	**Odds Ratio**	**LCI**	**UCI**	**Odds Ratio**	**LCI**	**UCI**	**Odds Ratio**	**LCI**	**UCI**	**Odds Ratio**	**LCI**	**UCI**
Gender	Male	(ref)	(ref)	(ref)	(ref)	(ref)	(ref)	(ref)	(ref)	(ref)	(ref)	(ref)	(ref)	(ref)	(ref)	(ref)
	Female	1.443	1.024	2.034	1.372	1.003	1.877	1.670	1.261	2.212	1.134	0.853	1.508	1.235	0.919	1.659
Ethnicity	White - British	(ref)	(ref)	(ref)	(ref)	(ref)	(ref)	(ref)	(ref)	(ref)	(ref)	(ref)	(ref)	(ref)	(ref)	(ref)
	Any other White background	1.376	0.552	3.432	2.268	1.225	4.200	1.353	0.643	2.845	1.130	0.504	2.530	0.958	0.415	2.214
	Asian or Asian British	3.651	1.069	12.473	1.667	0.491	5.656	3.08E-06	0	.	3.346	1.122	9.981	2.347	0.809	6.808
	Mixed/Dual background	2.037	0.622	6.663	7.39E-07	0	.	0.682	0.210	2.219	0.640	0.199	2.063	0.822	0.256	2.640
	Any other ethnic group	1.092	0.146	8.146	6.29E-07	0	.	1.271	0.294	5.501	1.189	0.274	5.152	2.272	0.680	7.586
SES	IMD 2010	5.606	1.191	26.386	5.276	1.214	22.926	2.689	0.777	9.304	5.189	1.309	20.570	13.370	3.374	52.979
NCMP participation (non-participation >0.2)		0.853	0.494	1.473	0.577	0.300	1.112	0.809	0.340	1.929	0.768	0.429	1.377	0.797	0.185	3.427
Factor 2		0.981	0.900	1.069	1.070	0.977	1.172	1.177	1.080	1.283	1.064	0.972	1.165	0.981	0.904	1.064

**Year 6 two-level**		**2006/07**			**2007/08**			**2008/09**			**2009/10**			**2010/11**		
n		5558			6163			6275			6478			6046		
Log likelihood	**Null**	-987.29			-963.28			-1068.95			-1206.63			-1102.13		
	**Individual**	-979.22			-950.71			-1064.68			-1197.20			-1076.18		
	**Factor**	-978.63			-950.06			-1062.46			-1196.89			-1076.12		
School variance	**Null**	0.123	0.032	0.474	0.213	0.072	0.627	0.253	0.101	0.633	0.151	0.046	0.494	0.119	0.027	0.535
	**Individual**	0.057	0.004	0.758	0.185	0.056	0.616	0.233	0.088	0.618	0.112			0.007	4.94E-11	8.76E+05
	**Factor**	0.055			0.184	0.055	0.618	0.233	0.086	0.633	0.106			0.007	1.50E-10	3.25E+05
**Individual**		**Odds Ratio**	**LCI**	**UCI**	**Odds Ratio**	**LCI**	**UCI**	**Odds Ratio**	**LCI**	**UCI**	**Odds Ratio**	**LCI**	**UCI**	**Odds Ratio**	**LCI**	**UCI**
Gender	Male	(ref)	(ref)	(ref)	(ref)	(ref)	(ref)	(ref)	(ref)	(ref)	(ref)	(ref)	(ref)	(ref)	(ref)	(ref)
	Female	0.891	0.686	1.156	1.089	0.832	1.426	0.996	0.774	1.283	1.675	1.264	2.221	1.367	1.068	1.750
Ethnicity	White - British	(ref)	(ref)	(ref)	(ref)	(ref)	(ref)	(ref)	(ref)	(ref)	(ref)	(ref)	(ref)	(ref)	(ref)	(ref)
	Any other White background	0.684	0.166	2.820	1.636	0.700	3.821	0.877	0.353	2.178	1.339	0.634	2.826	0.501	0.157	1.593
	Asian or Asian British	0.000	0	.	1.715	0.504	5.838	1.807	0.419	7.796	3.52E-06	0	.	1.790	0.531	6.031
	Mixed/Dual background	1.426	0.511	3.974	2.136	0.909	5.020	1.591	0.630	4.020	0.683	0.209	2.232	2.321	1.049	5.133
	Any other ethnic group	2.142	0.494	9.287	4.246	1.426	12.646	3.255	1.235	8.577	1.423	0.327	6.199	0.957	0.229	3.990
SES	IMD 2010	8.900	2.628	30.136	7.916	2.112	29.672	2.819	0.789	10.075	4.295	1.217	15.162	31.501	11.229	88.371
NCMP participation (non-participation >0.2)		0.853	0.579	1.257	0.585	0.364	0.940	0.970	0.620	1.516	0.726	0.304	1.736	0.597	0.303	1.176
**Factor**		**Odds Ratio**	**LCI**	**UCI**	**Odds Ratio**	**LCI**	**UCI**	**Odds Ratio**	**LCI**	**UCI**	**Odds Ratio**	**LCI**	**UCI**	**Odds Ratio**	**LCI**	**UCI**
Gender	Male	(ref)	(ref)	(ref)	(ref)	(ref)	(ref)	(ref)	(ref)	(ref)	(ref)	(ref)	(ref)	(ref)	(ref)	(ref)
	Female	0.890	0.684	1.157	1.089	0.832	1.425	0.997	0.774	1.283	1.322	1.041	1.678	1.367	1.068	1.750
Ethnicity	White - British	(ref)	(ref)	(ref)	(ref)	(ref)	(ref)	(ref)	(ref)	(ref)	(ref)	(ref)	(ref)	(ref)	(ref)	(ref)
	Any other White background	0.682	0.163	2.849	1.628	0.697	3.803	0.872	0.351	2.166	1.344	0.642	2.815	0.502	0.158	1.598
	Asian or Asian British	4.17E-06	0	.	1.674	0.492	5.691	1.708	0.395	7.376	3.12E-06	0	.	1.816	0.537	6.136
	Mixed/Dual background	1.430	0.509	4.018	2.124	0.903	4.991	1.592	0.630	4.025	0.902	0.322	2.527	2.331	1.053	5.157
	Any other ethnic group	2.204	0.504	9.647	4.120	1.382	12.285	3.059	1.160	8.066	1.619	0.568	4.610	0.965	0.231	4.027
SES	IMD 2010	10.144	2.912	35.338	6.969	1.847	26.285	2.275	0.637	8.122	5.821	1.775	19.094	33.106	11.367	96.415
NCMP participation (non-participation >0.2)		0.847	0.573	1.251	0.590	0.366	0.949	0.974	0.621	1.530	1.144	0.703	1.860	0.591	0.299	1.167
Factor 2		0.963	0.897	1.034	1.046	0.968	1.130	1.085	1.004	1.173	1.026	0.960	1.097	0.988	0.925	1.055

**Three-level**		**2006/07**			**2007/08**			**2008/09**			**2009/10**			**2010/11**		
n		9910			11353			11696			11851			11016		
Log likelihood	**Null**	-1602.53			-1719.64			-1992.75			-2063.11			-1898.27		
	**Individual**	-1594.02			-1700.41			-1984.67			-2053.82			-1868.08		
	**Factor**	-1593.60			-1698.99			-1976.34			-2053.07			-1867.92		
	**Interaction**	-1588.80			-1697.95			-1975.18			-2050.03			-1865.49		
School variance	**Null**	0.122	0.034	0.435	0.108	0.023	0.508	0.164	0.064	0.419	0.124			0.140	0.047	0.418
	**Individual**	0.074	0.010	0.551	0.098	0.020	0.484	0.135	0.045	0.411	0.081	0.013	0.499	0.057	0.006	0.533
	**Factor**	0.066	0.007	0.623	0.086	0.014	0.536	0.114	0.031	0.418	0.077	0.011	0.524	0.056	0.006	0.540
	**Interaction**	0.059	0.009	0.415	0.092	0.017	0.498	0.115	0.032	0.417	0.086	0.016	0.454	0.057	0.011	0.306
Year group variai	**Null**	0.007	1.29E-11	3.64E+06	0.097	0.010	0.914	0.035	4.31E-04	2.823	0.206			0.002	3.05E-43	1.03E+37
	**Individual**	0.012	1.75E-07	883.061	0.063	0.003	1.488	0.041	0.001	1.801	0.173	0.059	0.506	0.007	2.17E-13	2.01E+08
	**Factor**	0.013	2.70E-07	621.618	0.068	0.003	1.374	0.043	0.001	1.802	0.171	0.058	0.508	0.008	6.25E-12	9.33E+06
	**Interaction**	3.33E-18	0	.	0.055	0.001	2.083	0.041	0.001	1.936	0.154	0.050	0.475	1.36E-09	0	.
**Individual**		**Odds Ratio**	**LCI**	**UCI**	**Odds Ratio**	**LCI**	**UCI**	**Odds Ratio**	**LCI**	**UCI**	**Odds Ratio**	**LCI**	**UCI**	**Odds Ratio**	**LCI**	**UCI**
Gender	Male	(ref)	(ref)	(ref)	(ref)	(ref)	(ref)	(ref)	(ref)	(ref)	(ref)	(ref)	(ref)	(ref)	(ref)	(ref)
	Female	1.064	0.866	1.308	1.200	0.981	1.469	1.262	1.049	1.518	1.234	1.030	1.479	1.310	1.084	1.583
Ethnicity	White - British	(ref)	(ref)	(ref)	(ref)	(ref)	(ref)	(ref)	(ref)	(ref)	(ref)	(ref)	(ref)	(ref)	(ref)	(ref)
	Any other White background	0.979	0.455	2.108	2.011	1.229	3.290	1.120	0.633	1.982	1.255	0.730	2.156	0.730	0.372	1.432
	Asian or Asian British	1.550	0.473	5.075	1.689	0.713	4.002	0.796	0.191	3.313	1.560	0.554	4.393	2.056	0.926	4.566
	Mixed/Dual background	1.654	0.760	3.597	1.113	0.486	2.546	1.093	0.532	2.249	0.779	0.362	1.677	1.453	0.758	2.784
	Any other ethnic group	1.537	0.470	5.023	2.280	0.801	6.488	2.341	1.054	5.198	1.518	0.650	3.547	1.483	0.593	3.705
SES	IMD 2010	6.827	2.568	18.149	7.068	2.639	18.934	3.241	1.292	8.127	5.636	2.250	14.115	20.503	8.866	47.418
NCMP participation (non-participation >0.2)		0.885	0.642	1.219	0.574	0.394	0.837	0.932	0.637	1.364	0.902	0.621	1.310	0.679	0.366	1.262
**Factor**		**Odds Ratio**	**LCI**	**UCI**	**Odds Ratio**	**LCI**	**UCI**	**Odds Ratio**	**LCI**	**UCI**	**Odds Ratio**	**LCI**	**UCI**	**Odds Ratio**	**LCI**	**UCI**
Gender	Male	(ref)	(ref)	(ref)	(ref)	(ref)	(ref)	(ref)	(ref)	(ref)	(ref)	(ref)	(ref)	(ref)	(ref)	(ref)
	Female	1.064	0.866	1.308	1.200	0.981	1.469	1.260	1.047	1.516	1.231	1.027	1.476	1.310	1.084	1.583
Ethnicity	White - British	(ref)	(ref)	(ref)	(ref)	(ref)	(ref)	(ref)	(ref)	(ref)	(ref)	(ref)	(ref)	(ref)	(ref)	(ref)
	Any other White background	0.979	0.455	2.108	1.999	1.222	3.271	1.116	0.631	1.974	1.242	0.723	2.135	0.733	0.373	1.439
	Asian or Asian British	1.583	0.483	5.184	1.643	0.694	3.888	0.732	0.176	3.049	1.511	0.536	4.260	2.088	0.938	4.648
	Mixed/Dual background	1.670	0.768	3.632	1.107	0.484	2.533	1.090	0.530	2.242	0.773	0.359	1.663	1.458	0.760	2.795
	Any other ethnic group	1.578	0.483	5.159	2.206	0.775	6.277	2.167	0.976	4.810	1.485	0.635	3.470	1.501	0.600	3.756
SES	IMD 2010	7.481	2.756	20.304	6.070	2.259	16.314	2.408	0.972	5.967	5.142	2.044	12.936	21.695	9.147	51.459
NCMP participation (non-participation >0.2)		0.877	0.637	1.208	0.586	0.401	0.854	0.956	0.652	1.402	0.908	0.624	1.320	0.670	0.360	1.248
Factor 2		0.973	0.919	1.031	1.052	0.991	1.117	1.127	1.062	1.196	1.035	0.979	1.094	0.985	0.935	1.038
**Interaction**		**Odds Ratio**	**LCI**	**UCI**	**Odds Ratio**	**LCI**	**UCI**	**Odds Ratio**	**LCI**	**UCI**	**Odds Ratio**	**LCI**	**UCI**	**Odds Ratio**	**LCI**	**UCI**
Gender	Male	(ref)	(ref)	(ref)	(ref)	(ref)	(ref)	(ref)	(ref)	(ref)	(ref)	(ref)	(ref)	(ref)	(ref)	(ref)
	Female	1.063	0.865	1.307	1.203	0.983	1.472	1.260	1.047	1.516	1.232	1.028	1.476	1.313	1.086	1.586
Ethnicity	White - British	(ref)	(ref)	(ref)	(ref)	(ref)	(ref)	(ref)	(ref)	(ref)	(ref)	(ref)	(ref)	(ref)	(ref)	(ref)
	Any other White background	1.048	0.487	2.258	2.053	1.254	3.362	1.119	0.632	1.980	1.256	0.731	2.158	0.751	0.382	1.475
	Asian or Asian British	1.638	0.501	5.350	1.657	0.700	3.921	0.726	0.174	3.025	1.530	0.543	4.314	2.146	0.964	4.780
	Mixed/Dual background	1.657	0.762	3.605	1.111	0.486	2.543	1.093	0.531	2.249	0.789	0.366	1.699	1.496	0.780	2.870
	Any other ethnic group	1.631	0.499	5.327	2.197	0.772	6.249	2.174	0.979	4.828	1.470	0.629	3.436	1.504	0.601	3.764
SES	IMD 2010	8.055	2.981	21.770	6.082	2.262	16.349	2.385	0.962	5.912	5.116	2.032	12.880	22.041	9.311	52.178
NCMP participation (non-participation >0.2)		0.859	0.625	1.181	0.576	0.394	0.840	0.953	0.647	1.402	0.924	0.635	1.345	0.631	0.338	1.175
Factor 2		0.982	0.901	1.071	1.064	0.974	1.162	1.180	1.083	1.287	1.069	0.982	1.165	0.975	0.902	1.053
Year group	Reception	(ref)	(ref)	(ref)	(ref)	(ref)	(ref)	(ref)	(ref)	(ref)	(ref)	(ref)	(ref)	(ref)	(ref)	(ref)
	Year 6	1.540	0.786	3.019	1.289	0.629	2.641	1.727	0.849	3.514	1.701	0.866	3.341	1.107	0.593	2.064
Year group*factor	Reception	(ref)	(ref)	(ref)	(ref)	(ref)	(ref)	(ref)	(ref)	(ref)	(ref)	(ref)	(ref)	(ref)	(ref)	(ref)
	Year 6	0.984	0.883	1.096	0.984	0.880	1.100	0.921	0.827	1.026	0.953	0.857	1.060	1.019	0.924	1.124

Supporting file 3 to Williams, A.J., Wyatt, K.M., Williams, C.A., Logan, S., and Henley, W.E. Exploring the potential of a school impact on pupil weight status: exploratory factor analysis and repeat cross-sectional study of the National Child Measurement Programme.

S3 file - Detailed results and sensitivity analysis

Factor 2 - Location

Red shading indicates significance (p<0.05), Green shading indicates modles which had to be estimated in R

BMI-SDS

Single level		2006/07			2007/08			2008/09			2009/10			2010/11		
n		9910			11353			11696			11851			11016		
Log likelihood	**Null**	-14914.99			-16983.74			-17154.20			-17558.83			-16084.62		
	**Individual**	-14907.46			-16971.41			-17128.16			-17540.88			-16059.60		
	**Factor**	-14909.19			-16973.53			-17129.51			-17542.45			-16063.88		
Residual varianc	**Null**	1.187	1.155	1.221	1.166	1.136	1.197	1.100	1.072	1.128	1.133	1.104	1.162	1.085	1.057	1.114
	**Individual**	1.183	1.151	1.217	1.162	1.132	1.192	1.093	1.065	1.121	1.128	1.099	1.157	1.079	1.050	1.107
	**Factor**	1.183	1.150	1.216	1.161	1.131	1.192	1.093	1.065	1.121	1.127	1.099	1.156	1.079	1.051	1.108
**Null**		**Mean difference**	**LCI**	**UCI**	**Mean difference**	**LCI**	**UCI**	**Mean difference**	**LCI**	**UCI**	**Mean difference**	**LCI**	**UCI**	**Mean difference**	**LCI**	**UCI**
Constant		0.340	0.319	0.362	0.327	0.307	0.347	0.463	0.444	0.482	0.434	0.415	0.453	0.457	0.438	0.477
**Individual**		**Mean difference**	**LCI**	**UCI**	**Mean difference**	**LCI**	**UCI**	**Mean difference**	**LCI**	**UCI**	**Mean difference**	**LCI**	**UCI**	**Mean difference**	**LCI**	**UCI**
Constant		0.289	0.236	0.342	0.322	0.273	0.370	0.434	0.388	0.480	0.395	0.348	0.441	0.440	0.393	0.486
Gender	Male	(ref)	(ref)	(ref)	(ref)	(ref)	(ref)	(ref)	(ref)	(ref)	(ref)	(ref)	(ref)	(ref)	(ref)	(ref)
	Female	-0.085	-0.127	-0.042	-0.123	-0.162	-0.083	-0.136	-0.173	-0.098	-0.112	-0.150	-0.073	-0.143	-0.182	-0.104
Ethnicity	White - British	(ref)	(ref)	(ref)	(ref)	(ref)	(ref)	(ref)	(ref)	(ref)	(ref)	(ref)	(ref)	(ref)	(ref)	(ref)
	Any other White background	0.040	-0.115	0.194	-0.035	-0.163	0.092	0.083	-0.038	0.205	0.009	-0.118	0.135	0.031	-0.089	0.150
	Asian or Asian British	-0.164	-0.466	0.139	-0.183	-0.409	0.042	-0.205	-0.475	0.064	-0.096	-0.361	0.169	-0.035	-0.268	0.198
	Mixed/Dual background	0.204	0.005	0.402	-0.032	-0.198	0.135	0.021	-0.132	0.174	-0.135	-0.279	0.009	-0.058	-0.213	0.097
	Any other ethnic group	0.034	-0.262	0.331	0.007	-0.287	0.300	0.037	-0.194	0.268	0.156	-0.058	0.371	-0.145	-0.366	0.077
SES	IMD 2010	0.462	0.248	0.676	0.343	0.141	0.545	0.464	0.278	0.650	0.476	0.283	0.668	0.440	0.247	0.633
NCMP participation (non-participation >0.2)		-0.007	-0.068	0.054	-0.007	-0.065	0.051	0.027	-0.044	0.098	0.005	-0.063	0.073	0.038	-0.068	0.144
**Factor**		**Mean difference**	**LCI**	**UCI**	**Mean difference**	**LCI**	**UCI**	**Mean difference**	**LCI**	**UCI**	**Mean difference**	**LCI**	**UCI**	**Mean difference**	**LCI**	**UCI**
Constant		0.358	0.278	0.439	0.262	0.188	0.336	0.367	0.296	0.437	0.458	0.388	0.528	0.431	0.358	0.503
Gender	Male	(ref)	(ref)	(ref)	(ref)	(ref)	(ref)	(ref)	(ref)	(ref)	(ref)	(ref)	(ref)	(ref)	(ref)	(ref)
	Female	-0.085	-0.127	-0.042	-0.123	-0.162	-0.083	-0.136	-0.174	-0.098	-0.111	-0.149	-0.073	-0.143	-0.182	-0.104
Ethnicity	White - British	(ref)	(ref)	(ref)	(ref)	(ref)	(ref)	(ref)	(ref)	(ref)	(ref)	(ref)	(ref)	(ref)	(ref)	(ref)
	Any other White background	0.039	-0.115	0.194	-0.036	-0.164	0.091	0.083	-0.039	0.205	0.014	-0.113	0.140	0.030	-0.089	0.149
	Asian or Asian British	-0.152	-0.455	0.150	-0.194	-0.419	0.032	-0.218	-0.488	0.052	-0.081	-0.347	0.184	-0.037	-0.270	0.197
	Mixed/Dual background	0.210	0.011	0.408	-0.033	-0.199	0.133	0.020	-0.133	0.173	-0.131	-0.275	0.013	-0.058	-0.213	0.096
	Any other ethnic group	0.049	-0.248	0.346	-0.003	-0.297	0.291	0.025	-0.207	0.256	0.167	-0.047	0.382	-0.146	-0.368	0.076
SES	IMD 2010	0.495	0.279	0.711	0.312	0.109	0.516	0.430	0.242	0.618	0.511	0.317	0.706	0.435	0.240	0.631
NCMP participation (non-participation >0.2)		-0.012	-0.073	0.050	-0.002	-0.060	0.056	0.031	-0.040	0.102	0.003	-0.065	0.071	0.040	-0.067	0.146
Factor 2		-0.013	-0.024	-0.002	0.011	0.001	0.021	0.012	0.003	0.022	-0.012	-0.022	-0.002	0.002	-0.009	0.012

Reception two-level		2006/07			2007/08			2008/09			2009/10			2010/11		
n		4352			5190			5421			5373			4970		
Log likelihood	**Null**	-6239.98			-7379.80			-7429.67			-7484.45			-6734.36		
	**Individual**	-6245.04			-7371.92			-7428.49			-7486.78			-6735.96		
	**Factor**	-6248.40			-7375.63			-7430.66			-7489.46			-6739.56		
School variance	**Null**	0.041	0.026	0.065	0.042	0.028	0.063	0.025	0.016	0.039	0.040	0.028	0.059	0.014	0.006	0.030
	**Individual**	0.040	0.025	0.064	0.040	0.026	0.062	0.024	0.015	0.038	0.038	0.026	0.057	0.014	0.006	0.029
	**Factor**	0.041	0.026	0.065	0.041	0.027	0.062	0.023	0.014	0.036	0.039	0.026	0.057	0.014	0.006	0.029
Residual varianc	**Null**	1.000	0.958	1.045	0.977	0.939	1.016	0.888	0.855	0.923	0.922	0.887	0.958	0.867	0.833	0.903
	**Individual**	1.001	0.958	1.045	0.972	0.934	1.011	0.886	0.853	0.921	0.921	0.886	0.957	0.866	0.832	0.901
	**Factor**	1.000	0.958	1.045	0.972	0.934	1.011	0.887	0.853	0.921	0.921	0.886	0.957	0.866	0.832	0.901
**Null**		**Mean difference**	**LCI**	**UCI**	**Mean difference**	**LCI**	**UCI**	**Mean difference**	**LCI**	**UCI**	**Mean difference**	**LCI**	**UCI**	**Mean difference**	**LCI**	**UCI**
Constant		0.292	0.251	0.334	0.293	0.255	0.332	0.466	0.433	0.500	0.411	0.373	0.448	0.460	0.429	0.491
**Individual**		**Mean difference**	**LCI**	**UCI**	**Mean difference**	**LCI**	**UCI**	**Mean difference**	**LCI**	**UCI**	**Mean difference**	**LCI**	**UCI**	**Mean difference**	**LCI**	**UCI**
Constant		0.297	0.212	0.381	0.261	0.185	0.338	0.408	0.340	0.477	0.404	0.332	0.477	0.433	0.365	0.501
Gender	Male	(ref)	(ref)	(ref)	(ref)	(ref)	(ref)	(ref)	(ref)	(ref)	(ref)	(ref)	(ref)	(ref)	(ref)	(ref)
	Female	-0.041	-0.101	0.019	-0.087	-0.141	-0.032	-0.054	-0.104	-0.003	-0.063	-0.115	-0.011	-0.071	-0.124	-0.019
Ethnicity	White - British	(ref)	(ref)	(ref)	(ref)	(ref)	(ref)	(ref)	(ref)	(ref)	(ref)	(ref)	(ref)	(ref)	(ref)	(ref)
	Any other White background	-0.049	-0.234	0.137	-0.078	-0.232	0.077	-0.008	-0.166	0.150	-0.024	-0.187	0.140	0.057	-0.086	0.200
	Asian or Asian British	-0.060	-0.454	0.334	-0.550	-0.839	-0.261	-0.511	-0.872	-0.151	-0.166	-0.489	0.157	-0.025	-0.304	0.255
	Mixed/Dual background	0.157	-0.125	0.438	-0.130	-0.350	0.090	-0.029	-0.214	0.157	-0.227	-0.404	-0.050	-0.151	-0.339	0.036
	Any other ethnic group	-0.062	-0.437	0.312	-0.255	-0.674	0.164	-0.003	-0.313	0.306	-0.183	-0.483	0.118	-0.048	-0.344	0.248
SES	IMD 2010	0.174	-0.156	0.504	0.480	0.173	0.788	0.430	0.156	0.704	0.232	-0.057	0.520	0.313	0.037	0.589
NCMP participation (non-participation >0.2)		-0.113	-0.227	0.000	-0.071	-0.185	0.043	0.083	-0.074	0.241	-0.008	-0.121	0.104	0.140	-0.107	0.386
**Factor**		**Mean difference**	**LCI**	**UCI**	**Mean difference**	**LCI**	**UCI**	**Mean difference**	**LCI**	**UCI**	**Mean difference**	**LCI**	**UCI**	**Mean difference**	**LCI**	**UCI**
Constant		0.338	0.202	0.474	0.252	0.127	0.377	0.326	0.216	0.437	0.475	0.356	0.594	0.400	0.293	0.507
Gender	Male	(ref)	(ref)	(ref)	(ref)	(ref)	(ref)	(ref)	(ref)	(ref)	(ref)	(ref)	(ref)	(ref)	(ref)	(ref)
	Female	-0.041	-0.101	0.019	-0.087	-0.141	-0.032	-0.054	-0.104	-0.003	-0.062	-0.114	-0.010	-0.071	-0.123	-0.019
Ethnicity	White - British	(ref)	(ref)	(ref)	(ref)	(ref)	(ref)	(ref)	(ref)	(ref)	(ref)	(ref)	(ref)	(ref)	(ref)	(ref)
	Any other White background	-0.049	-0.234	0.137	-0.078	-0.233	0.077	-0.007	-0.165	0.151	-0.020	-0.183	0.143	0.055	-0.089	0.198
	Asian or Asian British	-0.056	-0.450	0.338	-0.551	-0.841	-0.262	-0.522	-0.882	-0.161	-0.156	-0.479	0.167	-0.031	-0.311	0.248
	Mixed/Dual background	0.161	-0.121	0.443	-0.130	-0.350	0.090	-0.029	-0.215	0.156	-0.225	-0.402	-0.048	-0.152	-0.339	0.036
	Any other ethnic group	-0.057	-0.432	0.318	-0.256	-0.675	0.163	-0.012	-0.321	0.298	-0.172	-0.473	0.129	-0.054	-0.350	0.242
SES	IMD 2010	0.184	-0.147	0.516	0.477	0.167	0.786	0.400	0.125	0.675	0.254	-0.036	0.544	0.297	0.018	0.576
NCMP participation (non-participation >0.2)		-0.116	-0.230	-0.003	-0.070	-0.184	0.045	0.096	-0.061	0.253	-0.005	-0.118	0.108	0.150	-0.097	0.398
Factor 2		-0.008	-0.028	0.012	0.002	-0.017	0.021	0.016	-0.001	0.032	-0.014	-0.032	0.005	0.006	-0.009	0.022

Year 6 two-level		2006/07			2007/08			2008/09			2009/10			2010/11		
n		5558			6163			6275			6478			6046		
Log likelihood	**Null**	-8607.29			-9526.30			-9610.21			-9959.39			-9263.90		
	**Individual**	-8598.02			-9519.79			-9588.28			-9944.15			-9240.02		
	**Factor**	-8600.20			-9521.89			-9591.85			-9947.41			-9243.94		
School variance	**Null**	0.021	0.011	0.041	0.018	0.009	0.036	0.031	0.019	0.051	0.023	0.013	0.041	0.003	2.01E-04	0.045
	**Individual**	0.019	0.009	0.039	0.018	0.009	0.036	0.029	0.018	0.048	0.022	0.012	0.040	0.001	1.73E-07	4.743
	**Factor**	0.018	0.008	0.038	0.017	0.008	0.035	0.029	0.018	0.048	0.022	0.012	0.040	0.001	7.44E-07	1.562
Residual varianc	**Null**	1.278	1.230	1.327	1.272	1.227	1.319	1.228	1.185	1.273	1.248	1.205	1.293	1.250	1.206	1.296
	**Individual**	1.273	1.225	1.322	1.267	1.222	1.314	1.219	1.176	1.263	1.240	1.198	1.285	1.240	1.196	1.286
	**Factor**	1.273	1.226	1.322	1.268	1.223	1.314	1.219	1.176	1.263	1.241	1.198	1.285	1.240	1.196	1.286
**Null**		**Mean difference**	**LCI**	**UCI**	**Mean difference**	**LCI**	**UCI**	**Mean difference**	**LCI**	**UCI**	**Mean difference**	**LCI**	**UCI**	**Mean difference**	**LCI**	**UCI**
Constant		0.385	0.348	0.422	0.349	0.315	0.383	0.456	0.420	0.493	0.469	0.435	0.503	0.457	0.428	0.487
**Individual**		**Mean difference**	**LCI**	**UCI**	**Mean difference**	**LCI**	**UCI**	**Mean difference**	**LCI**	**UCI**	**Mean difference**	**LCI**	**UCI**	**Mean difference**	**LCI**	**UCI**
Constant		0.320	0.240	0.400	0.379	0.303	0.456	0.460	0.385	0.536	0.423	0.348	0.497	0.442	0.374	0.510
Gender	Male	(ref)	(ref)	(ref)	(ref)	(ref)	(ref)	(ref)	(ref)	(ref)	(ref)	(ref)	(ref)	(ref)	(ref)	(ref)
	Female	-0.123	-0.183	-0.064	-0.155	-0.212	-0.098	-0.202	-0.257	-0.147	-0.145	-0.199	-0.090	-0.202	-0.259	-0.146
Ethnicity	White - British	(ref)	(ref)	(ref)	(ref)	(ref)	(ref)	(ref)	(ref)	(ref)	(ref)	(ref)	(ref)	(ref)	(ref)	(ref)
	Any other White background	0.184	-0.080	0.448	0.067	-0.154	0.287	0.168	-0.015	0.352	0.060	-0.131	0.250	-0.013	-0.210	0.184
	Asian or Asian British	-0.295	-0.751	0.160	0.166	-0.179	0.511	0.011	-0.382	0.404	0.031	-0.393	0.455	-0.100	-0.482	0.281
	Mixed/Dual background	0.236	-0.037	0.509	0.068	-0.176	0.312	0.053	-0.192	0.299	0.020	-0.208	0.247	0.069	-0.180	0.318
	Any other ethnic group	0.154	-0.312	0.620	0.175	-0.231	0.581	0.056	-0.282	0.394	0.463	0.162	0.763	-0.221	-0.544	0.102
SES	IMD 2010	0.595	0.268	0.922	0.192	-0.125	0.510	0.451	0.148	0.754	0.523	0.214	0.831	0.574	0.291	0.857
NCMP participation (non-participation >0.2)		0.034	-0.062	0.130	0.010	-0.079	0.100	0.005	-0.102	0.111	0.104	-0.021	0.228	0.018	-0.109	0.144
**Factor**		**Mean difference**	**LCI**	**UCI**	**Mean difference**	**LCI**	**UCI**	**Mean difference**	**LCI**	**UCI**	**Mean difference**	**LCI**	**UCI**	**Mean difference**	**LCI**	**UCI**
Constant		0.409	0.282	0.536	0.294	0.177	0.411	0.429	0.309	0.549	0.471	0.355	0.587	0.454	0.348	0.560
Gender	Male	(ref)	(ref)	(ref)	(ref)	(ref)	(ref)	(ref)	(ref)	(ref)	(ref)	(ref)	(ref)	(ref)	(ref)	(ref)
	Female	-0.124	-0.183	-0.064	-0.155	-0.212	-0.098	-0.202	-0.257	-0.147	-0.144	-0.199	-0.090	-0.202	-0.259	-0.146
Ethnicity	White - British	(ref)	(ref)	(ref)	(ref)	(ref)	(ref)	(ref)	(ref)	(ref)	(ref)	(ref)	(ref)	(ref)	(ref)	(ref)
	Any other White background	0.183	-0.081	0.446	0.066	-0.155	0.286	0.168	-0.016	0.351	0.062	-0.129	0.252	-0.012	-0.209	0.184
	Asian or Asian British	-0.286	-0.742	0.170	0.156	-0.188	0.501	0.008	-0.386	0.401	0.039	-0.386	0.464	-0.097	-0.480	0.285
	Mixed/Dual background	0.238	-0.035	0.511	0.066	-0.178	0.310	0.053	-0.192	0.299	0.022	-0.205	0.250	0.069	-0.180	0.319
	Any other ethnic group	0.166	-0.300	0.632	0.167	-0.239	0.573	0.052	-0.287	0.390	0.466	0.165	0.767	-0.220	-0.543	0.104
SES	IMD 2010	0.629	0.301	0.957	0.164	-0.153	0.482	0.441	0.136	0.745	0.540	0.230	0.850	0.579	0.293	0.866
NCMP participation (non-participation >0.2)		0.030	-0.065	0.126	0.014	-0.074	0.103	0.005	-0.101	0.112	0.097	-0.028	0.221	0.016	-0.111	0.143
Factor 2		-0.017	-0.035	0.002	0.016	-0.001	0.033	0.006	-0.012	0.024	-0.009	-0.026	0.008	-0.002	-0.017	0.013

Three-level		2006/07			2007/08			2008/09			2009/10			2010/11		
n		9910			11353			11696			11851			11016		
Log likelihood	**Null**	-14878.53			-16951.41			-17108.34			-17502.07			-16081.32		
	**Individual**	-14874.00			-16939.27			-17086.60			-17489.98			-16057.03		
	**Factor**	-14876.77			-16942.54			-17089.58			-17492.47			-16061.26		
	**Interaction**	-14874.91			-16945.77			-17095.49			-17495.11			-16067.64		
School variance	**Null**	0.021	0.012	0.038	0.008	0.002	0.029	0.013	0.006	0.027	0.019	0.010	0.034	0.003	3.15E-04	0.028
	**Individual**	0.020	0.011	0.037	0.007	0.002	0.030	0.011	0.005	0.026	0.017	0.009	0.032	0.002	6.69E-05	0.054
	**Factor**	0.020	0.011	0.037	0.007	0.002	0.030	0.010	0.004	0.025	0.017	0.009	0.032	0.002	7.76E-05	0.050
	**Interaction**	0.022	0.013	0.037	0.008	0.002	0.029	0.010	0.004	0.025	0.018	0.010	0.032	0.002	5.27E-05	0.062
Year group variai	**Null**	0.009	0.003	0.034	0.021	0.011	0.038	0.016	0.008	0.030	0.014	0.007	0.030	0.004	0.001	0.031
	**Individual**	0.009	0.002	0.034	0.021	0.012	0.039	0.016	0.008	0.031	0.014	0.007	0.030	0.004	0.001	0.030
	**Factor**	0.009	0.002	0.034	0.021	0.012	0.039	0.016	0.008	0.031	0.014	0.007	0.030	0.004	0.001	0.030
	**Interaction**	0.005	0.000	0.052	0.019	0.010	0.037	0.016	0.008	0.031	0.013	0.006	0.028	0.005	0.001	0.029
Residual varianc	**Null**	1.157	1.125	1.191	1.138	1.108	1.169	1.071	1.043	1.099	1.100	1.072	1.129	1.078	1.050	1.108
	**Individual**	1.155	1.122	1.188	1.134	1.104	1.164	1.066	1.038	1.094	1.097	1.068	1.125	1.073	1.044	1.102
	**Factor**	1.155	1.122	1.188	1.134	1.104	1.164	1.066	1.038	1.094	1.096	1.068	1.125	1.073	1.044	1.102
	**Interaction**	1.155	1.123	1.189	1.134	1.104	1.165	1.066	1.039	1.094	1.096	1.068	1.125	1.073	1.044	1.102
**Null**		**Mean difference**	**LCI**	**UCI**	**Mean difference**	**LCI**	**UCI**	**Mean difference**	**LCI**	**UCI**	**Mean difference**	**LCI**	**UCI**	**Mean difference**	**LCI**	**UCI**
Constant		0.345	0.314	0.376	0.322	0.295	0.349	0.461	0.434	0.488	0.444	0.415	0.472	0.459	0.437	0.480
**Individual**		**Mean difference**	**LCI**	**UCI**	**Mean difference**	**LCI**	**UCI**	**Mean difference**	**LCI**	**UCI**	**Mean difference**	**LCI**	**UCI**	**Mean difference**	**LCI**	**UCI**
Constant		0.318	0.257	0.379	0.324	0.268	0.379	0.439	0.387	0.492	0.421	0.366	0.475	0.442	0.393	0.491
Gender	Male	(ref)	(ref)	(ref)	(ref)	(ref)	(ref)	(ref)	(ref)	(ref)	(ref)	(ref)	(ref)	(ref)	(ref)	(ref)
	Female	-0.089	-0.132	-0.047	-0.124	-0.163	-0.084	-0.133	-0.171	-0.095	-0.109	-0.148	-0.071	-0.144	-0.183	-0.105
Ethnicity	White - British	(ref)	(ref)	(ref)	(ref)	(ref)	(ref)	(ref)	(ref)	(ref)	(ref)	(ref)	(ref)	(ref)	(ref)	(ref)
	Any other White background	0.028	-0.127	0.183	-0.030	-0.160	0.099	0.081	-0.041	0.203	0.027	-0.100	0.153	0.029	-0.091	0.149
	Asian or Asian British	-0.178	-0.481	0.125	-0.215	-0.440	0.011	-0.222	-0.491	0.047	-0.079	-0.343	0.186	-0.041	-0.274	0.192
	Mixed/Dual background	0.199	0.001	0.396	-0.032	-0.198	0.133	0.011	-0.141	0.163	-0.114	-0.257	0.029	-0.057	-0.212	0.098
	Any other ethnic group	0.030	-0.268	0.328	-0.017	-0.310	0.276	0.023	-0.208	0.255	0.197	-0.018	0.411	-0.141	-0.363	0.081
SES	IMD 2010	0.371	0.130	0.612	0.324	0.098	0.549	0.432	0.222	0.643	0.375	0.156	0.594	0.436	0.234	0.638
NCMP participation (non-participation >0.2)		-0.023	-0.096	0.050	-0.017	-0.087	0.054	0.011	-0.073	0.096	0.027	-0.056	0.109	0.042	-0.068	0.153
**Factor**		**Mean difference**	**LCI**	**UCI**	**Mean difference**	**LCI**	**UCI**	**Mean difference**	**LCI**	**UCI**	**Mean difference**	**LCI**	**UCI**	**Mean difference**	**LCI**	**UCI**
Constant		0.380	0.280	0.480	0.279	0.191	0.367	0.387	0.302	0.473	0.484	0.395	0.573	0.435	0.358	0.511
Gender	Male	(ref)	(ref)	(ref)	(ref)	(ref)	(ref)	(ref)	(ref)	(ref)	(ref)	(ref)	(ref)	(ref)	(ref)	(ref)
	Female	-0.089	-0.132	-0.047	-0.124	-0.163	-0.084	-0.133	-0.171	-0.095	-0.109	-0.147	-0.071	-0.144	-0.183	-0.105
Ethnicity	White - British	(ref)	(ref)	(ref)	(ref)	(ref)	(ref)	(ref)	(ref)	(ref)	(ref)	(ref)	(ref)	(ref)	(ref)	(ref)
	Any other White background	0.028	-0.127	0.183	-0.031	-0.161	0.098	0.081	-0.041	0.203	0.029	-0.097	0.155	0.029	-0.091	0.148
	Asian or Asian British	-0.172	-0.475	0.131	-0.219	-0.445	0.006	-0.228	-0.497	0.041	-0.070	-0.335	0.194	-0.043	-0.276	0.191
	Mixed/Dual background	0.202	0.004	0.399	-0.033	-0.198	0.133	0.011	-0.141	0.163	-0.112	-0.255	0.032	-0.058	-0.212	0.097
	Any other ethnic group	0.037	-0.261	0.335	-0.021	-0.314	0.272	0.018	-0.214	0.249	0.202	-0.012	0.417	-0.142	-0.364	0.080
SES	IMD 2010	0.388	0.146	0.630	0.310	0.084	0.536	0.417	0.206	0.628	0.395	0.175	0.614	0.432	0.228	0.636
NCMP participation (non-participation >0.2)		-0.026	-0.099	0.047	-0.014	-0.085	0.057	0.015	-0.070	0.099	0.025	-0.057	0.107	0.044	-0.067	0.155
Factor 2		-0.012	-0.027	0.003	0.009	-0.005	0.022	0.010	-0.003	0.023	-0.012	-0.026	0.001	0.001	-0.010	0.012
**Interaction**		**Mean difference**	**LCI**	**UCI**	**Mean difference**	**LCI**	**UCI**	**Mean difference**	**LCI**	**UCI**	**Mean difference**	**LCI**	**UCI**	**Mean difference**	**LCI**	**UCI**
Constant		0.293	0.168	0.419	0.287	0.169	0.404	0.369	0.257	0.482	0.455	0.339	0.571	0.417	0.313	0.522
Gender	Male	(ref)	(ref)	(ref)	(ref)	(ref)	(ref)	(ref)	(ref)	(ref)	(ref)	(ref)	(ref)	(ref)	(ref)	(ref)
	Female	-0.090	-0.132	-0.047	-0.123	-0.163	-0.084	-0.133	-0.171	-0.096	-0.109	-0.147	-0.071	-0.144	-0.183	-0.105
Ethnicity	White - British	(ref)	(ref)	(ref)	(ref)	(ref)	(ref)	(ref)	(ref)	(ref)	(ref)	(ref)	(ref)	(ref)	(ref)	(ref)
	Any other White background	0.044	-0.111	0.199	-0.024	-0.154	0.106	0.081	-0.041	0.203	0.031	-0.095	0.157	0.028	-0.092	0.148
	Asian or Asian British	-0.168	-0.470	0.135	-0.215	-0.441	0.010	-0.228	-0.497	0.041	-0.066	-0.330	0.199	-0.044	-0.278	0.190
	Mixed/Dual background	0.199	0.001	0.396	-0.031	-0.197	0.134	0.010	-0.142	0.162	-0.108	-0.251	0.036	-0.058	-0.213	0.097
	Any other ethnic group	0.044	-0.254	0.342	-0.021	-0.314	0.272	0.018	-0.213	0.250	0.201	-0.013	0.415	-0.143	-0.365	0.079
SES	IMD 2010	0.406	0.164	0.647	0.315	0.089	0.542	0.416	0.205	0.627	0.395	0.175	0.614	0.431	0.227	0.635
NCMP participation (non-participation >0.2)		-0.028	-0.100	0.044	-0.021	-0.092	0.050	0.019	-0.066	0.105	0.034	-0.048	0.117	0.044	-0.068	0.156
Factor 2		-0.007	-0.026	0.013	0.002	-0.016	0.020	0.014	-0.003	0.032	-0.013	-0.031	0.005	0.005	-0.011	0.021
Year group	Reception	(ref)	(ref)	(ref)	(ref)	(ref)	(ref)	(ref)	(ref)	(ref)	(ref)	(ref)	(ref)	(ref)	(ref)	(ref)
	Year 6	0.153	0.011	0.296	-0.018	-0.161	0.125	0.034	-0.103	0.171	0.042	-0.093	0.177	0.032	-0.099	0.164
Year group*factor	Reception	(ref)	(ref)	(ref)	(ref)	(ref)	(ref)	(ref)	(ref)	(ref)	(ref)	(ref)	(ref)	(ref)	(ref)	(ref)
	Year 6	-0.010	-0.033	0.013	0.013	-0.011	0.037	-0.008	-0.031	0.014	0.004	-0.019	0.026	-0.006	-0.027	0.015

Supporting file 3 to Williams, A.J., Wyatt, K.M., Williams, C.A., Logan, S., and Henley, W.E. Exploring the potential of a school impact on pupil weight status: exploratory factor analysis and repeat cross-sectional study of the National Child Measurement Programme.

S3 file - Detailed results and sensitivity analysis

Factor 3 - Resource

Red shading indicates significance (p<0.05), Green shading indicates modles which had to be estimated in R

Overweight (UK90)

Single level

		2006/07			2007/08			2008/09			2009/10			2010/11		
n		8747			10061			10149			10356			9604		
Log likelihood	**Null**	-3723.27			-4273.38			-4660.99			-4699.66			-4334.16		
	**Individual**	-3717.66			-4267.11			-4657.93			-4694.99			-4326.89		
	**Factor**	-3717.62			-4266.57			-4657.82			-4694.98			-4326.82		
**Individual**		**Odds Ratio**	**LCI**	**UCI**	**Odds Ratio**	**LCI**	**UCI**	**Odds Ratio**	**LCI**	**UCI**	**Odds Ratio**	**LCI**	**UCI**	**Odds Ratio**	**LCI**	**UCI**
Gender	Male	(ref)	(ref)	(ref)	(ref)	(ref)	(ref)	(ref)	(ref)	(ref)	(ref)	(ref)	(ref)	(ref)	(ref)	(ref)
	Female	0.880	0.783	0.990	0.897	0.804	1.001	0.933	0.842	1.035	0.920	0.830	1.020	0.856	0.769	0.953
Ethnicity	White - British	(ref)	(ref)	(ref)	(ref)	(ref)	(ref)	(ref)	(ref)	(ref)	(ref)	(ref)	(ref)	(ref)	(ref)	(ref)
	Any other White background	0.935	0.610	1.435	0.813	0.559	1.184	0.947	0.675	1.329	1.086	0.780	1.512	1.191	0.876	1.621
	Asian or Asian British	0.542	0.194	1.518	0.895	0.471	1.701	0.776	0.348	1.728	0.993	0.484	2.041	0.775	0.383	1.571
	Mixed/Dual background	0.998	0.563	1.767	0.733	0.441	1.220	0.816	0.523	1.275	1.011	0.688	1.484	0.868	0.555	1.357
	Any other ethnic group	1.197	0.556	2.577	0.908	0.382	2.156	0.571	0.260	1.254	0.871	0.470	1.614	1.102	0.616	1.970
SES	IMD 2010	1.873	1.055	3.324	1.888	1.095	3.256	1.163	0.698	1.936	1.719	1.033	2.858	1.356	0.792	2.320
NCMP participation (non-participation >0.2)		0.964	0.814	1.142	1.079	0.923	1.262	1.076	0.889	1.302	1.126	0.946	1.341	1.280	0.971	1.686
**Factor**		**Odds Ratio**	**LCI**	**UCI**	**Odds Ratio**	**LCI**	**UCI**	**Odds Ratio**	**LCI**	**UCI**	**Odds Ratio**	**LCI**	**UCI**	**Odds Ratio**	**LCI**	**UCI**
Gender	Male	(ref)	(ref)	(ref)	(ref)	(ref)	(ref)	(ref)	(ref)	(ref)	(ref)	(ref)	(ref)	(ref)	(ref)	(ref)
	Female	0.881	0.783	0.990	0.897	0.804	1.001	0.933	0.842	1.035	0.920	0.830	1.020	0.856	0.769	0.953
Ethnicity	White - British	(ref)	(ref)	(ref)	(ref)	(ref)	(ref)	(ref)	(ref)	(ref)	(ref)	(ref)	(ref)	(ref)	(ref)	(ref)
	Any other White background	0.933	0.608	1.432	0.810	0.557	1.179	0.949	0.676	1.332	1.086	0.780	1.512	1.191	0.875	1.620
	Asian or Asian British	0.541	0.193	1.514	0.886	0.466	1.686	0.779	0.349	1.734	0.992	0.483	2.038	0.772	0.381	1.565
	Mixed/Dual background	0.996	0.562	1.765	0.732	0.440	1.217	0.818	0.524	1.277	1.010	0.688	1.484	0.867	0.555	1.356
	Any other ethnic group	1.192	0.554	2.569	0.898	0.378	2.134	0.573	0.261	1.258	0.871	0.470	1.613	1.100	0.615	1.967
SES	IMD 2010	1.857	1.044	3.303	1.837	1.063	3.175	1.179	0.706	1.971	1.711	1.025	2.857	1.341	0.781	2.301
NCMP participation (non-participation >0.2)		0.965	0.814	1.143	1.084	0.927	1.268	1.076	0.889	1.302	1.125	0.945	1.340	1.279	0.971	1.686
Factor 3		1.023	0.885	1.183	1.071	0.942	1.217	0.971	0.860	1.097	1.008	0.893	1.138	1.025	0.900	1.166

Reception two-level

		2006/07			2007/08			2008/09			2009/10			2010/11		
n		4002			4769			4889			4908			4520		
Log likelihood	**Null**	-1621.95			-1944.48			-2250.55			-2162.02			-2008.25		
	**Individual**	-1619.64			-1932.20			-2246.54			-2158.56			-2003.00		
	**Factor**	-1617.17			-1931.23			-2246.54			-2158.39			-2002.99		
School variance	**Null**	0.084	0.024	0.286	0.012	0.000	3.544	0.088	0.039	0.200	0.027	0.002	0.300	0.018	4.06E-04	0.777
	**Individual**	0.080	0.022	0.288	1.64E-08	0	.	0.087	0.038	0.197	0.025	0.002	0.338	0.016	2.79E-04	0.961
	**Factor**	0.071	0.017	0.289	1.36E-10	0	.	0.087	0.038	0.197	0.025	0.002	0.325	0.017	3.15E-04	0.904
**Individual**		**Odds Ratio**	**LCI**	**UCI**	**Odds Ratio**	**LCI**	**UCI**	**Odds Ratio**	**LCI**	**UCI**	**Odds Ratio**	**LCI**	**UCI**	**Odds Ratio**	**LCI**	**UCI**
Gender	Male	(ref)	(ref)	(ref)	(ref)	(ref)	(ref)	(ref)	(ref)	(ref)	(ref)	(ref)	(ref)	(ref)	(ref)	(ref)
	Female	0.914	0.763	1.095	0.830	0.704	0.977	0.941	0.810	1.094	0.875	0.751	1.020	0.841	0.718	0.986
Ethnicity	White - British	(ref)	(ref)	(ref)	(ref)	(ref)	(ref)	(ref)	(ref)	(ref)	(ref)	(ref)	(ref)	(ref)	(ref)	(ref)
	Any other White background	0.709	0.383	1.310	0.639	0.378	1.078	0.801	0.486	1.322	1.365	0.885	2.105	1.147	0.758	1.735
	Asian or Asian British	0.913	0.264	3.154	0.406	0.125	1.321	0.373	0.086	1.610	0.563	0.170	1.867	0.671	0.260	1.735
	Mixed/Dual background	1.011	0.421	2.427	0.322	0.117	0.884	0.817	0.457	1.462	0.854	0.491	1.485	0.808	0.436	1.496
	Any other ethnic group	1.262	0.469	3.397	0.597	0.138	2.575	1.021	0.414	2.517	0.995	0.412	2.399	0.668	0.234	1.905
SES	IMD 2010	1.336	0.535	3.337	2.754	1.257	6.038	1.534	0.706	3.332	1.407	0.659	3.006	1.493	0.672	3.319
NCMP participation (non-participation >0.2)		0.819	0.604	1.112	0.841	0.639	1.107	1.412	0.945	2.109	0.937	0.727	1.207	1.724	0.927	3.208
**Factor**		**Odds Ratio**	**LCI**	**UCI**	**Odds Ratio**	**LCI**	**UCI**	**Odds Ratio**	**LCI**	**UCI**	**Odds Ratio**	**LCI**	**UCI**	**Odds Ratio**	**LCI**	**UCI**
Gender	Male	(ref)	(ref)	(ref)	(ref)	(ref)	(ref)	(ref)	(ref)	(ref)	(ref)	(ref)	(ref)	(ref)	(ref)	(ref)
	Female	0.920	0.768	1.101	0.830	0.704	0.977	0.941	0.810	1.094	0.875	0.751	1.020	0.841	0.718	0.986
Ethnicity	White - British	(ref)	(ref)	(ref)	(ref)	(ref)	(ref)	(ref)	(ref)	(ref)	(ref)	(ref)	(ref)	(ref)	(ref)	(ref)
	Any other White background	0.683	0.369	1.262	0.637	0.377	1.076	0.801	0.486	1.322	1.366	0.886	2.106	1.147	0.758	1.736
	Asian or Asian British	0.886	0.257	3.056	0.399	0.123	1.300	0.373	0.086	1.610	0.566	0.171	1.880	0.672	0.260	1.738
	Mixed/Dual background	0.987	0.411	2.368	0.322	0.117	0.885	0.817	0.457	1.462	0.857	0.493	1.490	0.808	0.436	1.496
	Any other ethnic group	1.226	0.456	3.295	0.592	0.137	2.552	1.021	0.414	2.517	1.002	0.415	2.419	0.669	0.235	1.907
SES	IMD 2010	1.226	0.493	3.048	2.563	1.163	5.647	1.534	0.702	3.350	1.448	0.673	3.117	1.502	0.672	3.353
NCMP participation (non-participation >0.2)		0.826	0.610	1.119	0.856	0.650	1.128	1.412	0.944	2.111	0.941	0.730	1.213	1.728	0.928	3.218
Factor 3		1.322	1.035	1.690	1.157	0.943	1.420	0.999	0.818	1.222	0.944	0.776	1.147	0.986	0.804	1.209

Year 6 two-level

		2006/07			2007/08			2008/09			2009/10			2010/11		
n		4745			5292			5260			5448			5084		
Log likelihood	**Null**	-2095.61			-2325.40			-2404.51			-2534.54			-2325.19		
	**Individual**	-2088.90			-2322.30			-2400.36			-2526.67			-2321.69		
	**Factor**	-2087.65			-2322.30			-2399.88			-2526.30			-2321.58		
School variance	**Null**	0.019	0.001	0.468	0.016	3.19E-04	0.763	0.043	0.009	0.200	0.026	0.003	0.216	5.17E-16	0	.
	**Individual**	0.004	1.10E-08	1830.55	0.009	9.25E-06	7.967	0.040	0.008	0.207	0.020	0.001	0.288	7.71E-19	0	.
	**Factor**	4.99E-10	0	.	0.009	8.91E-06	8.219	0.043	0.009	0.201	0.023	0.002	0.242	1.29E-14	0	.
**Individual**		**Odds Ratio**	**LCI**	**UCI**	**Odds Ratio**	**LCI**	**UCI**	**Odds Ratio**	**LCI**	**UCI**	**Odds Ratio**	**LCI**	**UCI**	**Odds Ratio**	**LCI**	**UCI**
Gender	Male	(ref)	(ref)	(ref)	(ref)	(ref)	(ref)	(ref)	(ref)	(ref)	(ref)	(ref)	(ref)	(ref)	(ref)	(ref)
	Female	0.851	0.729	0.994	0.951	0.821	1.102	0.926	0.802	1.070	0.957	0.832	1.102	0.869	0.751	1.006
Ethnicity	White - British	(ref)	(ref)	(ref)	(ref)	(ref)	(ref)	(ref)	(ref)	(ref)	(ref)	(ref)	(ref)	(ref)	(ref)	(ref)
	Any other White background	1.418	0.764	2.632	1.154	0.668	1.994	1.099	0.685	1.763	0.842	0.499	1.423	1.269	0.797	2.021
	Asian or Asian British	0.241	0.032	1.791	1.604	0.721	3.570	1.232	0.458	3.317	1.633	0.638	4.182	0.897	0.308	2.613
	Mixed/Dual background	1.001	0.467	2.144	1.233	0.670	2.268	0.755	0.371	1.537	1.210	0.702	2.085	0.958	0.499	1.839
	Any other ethnic group	1.113	0.318	3.891	1.198	0.403	3.558	0.159	0.022	1.167	0.781	0.327	1.866	1.497	0.735	3.051
SES	IMD 2010	2.488	1.141	5.426	1.398	0.646	3.023	0.865	0.406	1.845	2.322	1.121	4.810	1.247	0.596	2.610
NCMP participation (non-participation >0.2)		1.047	0.845	1.296	1.199	0.986	1.459	0.977	0.768	1.242	1.487	1.143	1.933	1.172	0.858	1.602
**Factor**		**Odds Ratio**	**LCI**	**UCI**	**Odds Ratio**	**LCI**	**UCI**	**Odds Ratio**	**LCI**	**UCI**	**Odds Ratio**	**LCI**	**UCI**	**Odds Ratio**	**LCI**	**UCI**
Gender	Male	(ref)	(ref)	(ref)	(ref)	(ref)	(ref)	(ref)	(ref)	(ref)	(ref)	(ref)	(ref)	(ref)	(ref)	(ref)
	Female	0.851	0.729	0.994	0.951	0.821	1.102	0.926	0.801	1.070	0.957	0.832	1.102	0.869	0.751	1.007
Ethnicity	White - British	(ref)	(ref)	(ref)	(ref)	(ref)	(ref)	(ref)	(ref)	(ref)	(ref)	(ref)	(ref)	(ref)	(ref)	(ref)
	Any other White background	1.423	0.767	2.640	1.155	0.668	1.995	1.105	0.689	1.773	0.838	0.496	1.416	1.268	0.796	2.020
	Asian or Asian British	0.245	0.033	1.819	1.604	0.721	3.572	1.241	0.461	3.343	1.601	0.624	4.104	0.887	0.304	2.588
	Mixed/Dual background	1.003	0.469	2.147	1.233	0.670	2.269	0.759	0.373	1.545	1.202	0.697	2.073	0.955	0.497	1.834
	Any other ethnic group	1.134	0.324	3.964	1.198	0.403	3.562	0.160	0.022	1.178	0.777	0.325	1.858	1.493	0.732	3.042
SES	IMD 2010	2.648	1.222	5.739	1.398	0.645	3.030	0.893	0.417	1.913	2.247	1.078	4.681	1.222	0.581	2.569
NCMP participation (non-participation >0.2)		1.044	0.844	1.291	1.199	0.986	1.459	0.972	0.764	1.237	1.481	1.137	1.928	1.174	0.859	1.604
Factor 3		0.856	0.706	1.038	0.998	0.843	1.182	0.915	0.764	1.095	1.075	0.910	1.270	1.042	0.878	1.236

Three-level

		2006/07			2007/08			2008/09			2009/10			2010/11		
n		8747			10061			10149			10356			9604		
Log likelihood	**Null**	-3721.38			-4272.82			-4655.48			-4698.53			-4334.14		
	**Individual**	-3716.23			-4266.83			-4652.40			-4693.85			-4326.84		
	**Factor**	-3716.14			-4266.31			-4652.17			-4693.83			-4326.78		
	**Interaction**	-3709.37			-4262.78			-4651.82			-4690.67			-4326.33		
School variance	**Null**	2.31E-11	0	.	0.006	2.30E-06	16.533	0.001	7.92E-22	1.86E+15	0.003	3.68E-10	21371.7	0.003		
	**Individual**	5.24E-18	0	.	0.001	2.70E-24	3.73E+17	9.97E-09	0	.	0.003	1.66E-09	5768.52	0.005	8.60E-06	2.605
	**Factor**	3.05E-21	0	.	5.44E-07	0	.	1.24E-07			0.003	1.02E-09	8808.61	0.004	4.05E-06	4.532
	**Interaction**	3.32E-14	0	.	0.007	0.0000715	0.734	0.001	1.54E-29	4.83E+22	0.007	2.10E-05	2.656	0.004	3.02E-06	5.640
Year group variai	**Null**	0.048	0.015	0.154	0.017	3.49E-04	0.813	0.066	0.023	0.190	0.026	0.002	0.304	9.93E-12		
	**Individual**	0.042	0.012	0.156	0.016	2.63E-04	0.943	0.067	0.033	0.138	0.026	0.002	0.305	7.28E-14	0	.
	**Factor**	0.043	0.012	0.156	0.016	0.001	0.280	0.068			0.027	0.002	0.301	1.10E-09	0	.
	**Interaction**	0.027	0.004	0.189	7.11E-09	0	.	0.067	0.023	0.192	0.020	0.001	0.471	1.82E-15	0	.
**Individual**		**Odds Ratio**	**LCI**	**UCI**	**Odds Ratio**	**LCI**	**UCI**	**Odds Ratio**	**LCI**	**UCI**	**Odds Ratio**	**LCI**	**UCI**	**Odds Ratio**	**LCI**	**UCI**
Gender	Male	(ref)	(ref)	(ref)	(ref)	(ref)	(ref)	(ref)	(ref)	(ref)	(ref)	(ref)	(ref)	(ref)	(ref)	(ref)
	Female	0.880	0.782	0.990	0.897	0.804	1.001	0.933	0.841	1.035	0.921	0.831	1.022	0.856	0.768	0.953
Ethnicity	White - British	(ref)	(ref)	(ref)	(ref)	(ref)	(ref)	(ref)	(ref)	(ref)	(ref)	(ref)	(ref)	(ref)	(ref)	(ref)
	Any other White background	0.934	0.607	1.438	0.817	0.560	1.191	0.935	0.663	1.318	1.096	0.785	1.529	1.193	0.876	1.623
	Asian or Asian British	0.540	0.192	1.518	0.894	0.470	1.703	0.762	0.339	1.710	0.998	0.484	2.056	0.773	0.381	1.567
	Mixed/Dual background	0.992	0.558	1.762	0.734	0.441	1.222	0.799	0.510	1.253	1.011	0.687	1.487	0.867	0.555	1.356
	Any other ethnic group	1.190	0.549	2.580	0.904	0.380	2.153	0.569	0.258	1.257	0.882	0.474	1.638	1.103	0.616	1.973
SES	IMD 2010	1.789	0.984	3.253	1.873	1.074	3.267	1.150	0.668	1.979	1.764	1.040	2.991	1.357	0.789	2.333
NCMP participation (non-participation >0.2)		0.959	0.802	1.147	1.079	0.919	1.266	1.073	0.872	1.321	1.132	0.943	1.360	1.285	0.972	1.698
**Factor**		**Odds Ratio**	**LCI**	**UCI**	**Odds Ratio**	**LCI**	**UCI**	**Odds Ratio**	**LCI**	**UCI**	**Odds Ratio**	**LCI**	**UCI**	**Odds Ratio**	**LCI**	**UCI**
Gender	Male	(ref)	(ref)	(ref)	(ref)	(ref)	(ref)	(ref)	(ref)	(ref)	(ref)	(ref)	(ref)	(ref)	(ref)	(ref)
	Female	0.880	0.782	0.990	0.897	0.804	1.001	0.933	0.840	1.035	0.921	0.831	1.022	0.856	0.769	0.953
Ethnicity	White - British	(ref)	(ref)	(ref)	(ref)	(ref)	(ref)	(ref)	(ref)	(ref)	(ref)	(ref)	(ref)	(ref)	(ref)	(ref)
	Any other White background	0.931	0.605	1.434	0.814	0.558	1.186	0.937	0.664	1.321	1.095	0.785	1.528	1.192	0.875	1.622
	Asian or Asian British	0.538	0.191	1.512	0.887	0.465	1.689	0.765	0.340	1.721	0.995	0.483	2.052	0.769	0.379	1.561
	Mixed/Dual background	0.990	0.557	1.760	0.732	0.440	1.219	0.801	0.511	1.257	1.010	0.686	1.486	0.867	0.554	1.356
	Any other ethnic group	1.185	0.546	2.570	0.895	0.376	2.131	0.571	0.258	1.265	0.881	0.474	1.637	1.101	0.615	1.971
SES	IMD 2010	1.768	0.970	3.223	1.826	1.047	3.185	1.171	0.678	2.021	1.752	1.029	2.981	1.343	0.778	2.316
NCMP participation (non-participation >0.2)		0.960	0.802	1.148	1.083	0.923	1.272	1.073	0.872	1.321	1.131	0.942	1.359	1.284	0.972	1.696
Factor 3		1.034	0.886	1.207	1.071	0.939	1.222	0.956	0.835	1.094	1.014	0.893	1.153	1.023	0.897	1.167
**Interaction**		**Odds Ratio**	**LCI**	**UCI**	**Odds Ratio**	**LCI**	**UCI**	**Odds Ratio**	**LCI**	**UCI**	**Odds Ratio**	**LCI**	**UCI**	**Odds Ratio**	**LCI**	**UCI**
Gender	Male	(ref)	(ref)	(ref)	(ref)	(ref)	(ref)	(ref)	(ref)	(ref)	(ref)	(ref)	(ref)	(ref)	(ref)	(ref)
	Female	0.881	0.783	0.991	0.898	0.805	1.002	0.932	0.840	1.034	0.921	0.831	1.022	0.856	0.769	0.953
Ethnicity	White - British	(ref)	(ref)	(ref)	(ref)	(ref)	(ref)	(ref)	(ref)	(ref)	(ref)	(ref)	(ref)	(ref)	(ref)	(ref)
	Any other White background	0.940	0.610	1.447	0.832	0.570	1.212	0.937	0.664	1.321	1.101	0.789	1.537	1.199	0.881	1.633
	Asian or Asian British	0.537	0.191	1.509	0.894	0.469	1.702	0.763	0.340	1.712	1.003	0.486	2.067	0.776	0.382	1.575
	Mixed/Dual background	0.983	0.554	1.747	0.741	0.445	1.233	0.800	0.510	1.254	1.021	0.694	1.502	0.871	0.557	1.362
	Any other ethnic group	1.202	0.555	2.603	0.907	0.381	2.157	0.571	0.259	1.262	0.884	0.476	1.643	1.100	0.615	1.968
SES	IMD 2010	1.828	1.009	3.312	1.820	1.044	3.173	1.164	0.673	2.011	1.772	1.040	3.020	1.352	0.784	2.332
NCMP participation (non-participation >0.2)		0.952	0.798	1.136	1.071	0.914	1.256	1.070	0.867	1.322	1.153	0.960	1.385	1.266	0.956	1.675
Factor 3		1.299	1.029	1.638	1.177	0.960	1.443	1.016	0.835	1.235	0.927	0.763	1.126	0.996	0.816	1.216
Year group	Reception	(ref)	(ref)	(ref)	(ref)	(ref)	(ref)	(ref)	(ref)	(ref)	(ref)	(ref)	(ref)	(ref)	(ref)	(ref)
	Year 6	2.678	1.424	5.037	1.601	0.923	2.777	1.256	0.715	2.206	0.806	0.471	1.381	0.961	0.556	1.660
Year group*factor	Reception	(ref)	(ref)	(ref)	(ref)	(ref)	(ref)	(ref)	(ref)	(ref)	(ref)	(ref)	(ref)	(ref)	(ref)	(ref)
	Year 6	0.668	0.491	0.907	0.850	0.655	1.104	0.892	0.681	1.166	1.176	0.91202	1.51574	1.044	0.805	1.355

Supporting file 3 to Williams, A.J., Wyatt, K.M., Williams, C.A., Logan, S., and Henley, W.E. Exploring the potential of a school impact on pupil weight status: exploratory factor analysis and repeat cross-sectional study of the National Child Measurement Programme.

S3 file - Detailed results and sensitivity analysis

Factor 3 - Resource

Red shading indicates significance (p<0.05), Green shading indicates modles which had to be estimated in R

Obese (UK90)

**Single level**		**2006/07**			**2007/08**			**2008/09**			**2009/10**			**2010/11**		
n		9910			11353			11696			11851			11016		
Log likelihood	**Null**	-3583.69			-4023.42			-4569.33			-4491.54			-4218.10		
	**Individual**	-3567.36			-4005.35			-4548.51			-4469.42			-4184.92		
	**Factor**	-3566.83			-4004.94			-4548.43			-4469.16			-4184.91		
**Individual**		**Odds Ratio**	**LCI**	**UCI**	**Odds Ratio**	**LCI**	**UCI**	**Odds Ratio**	**LCI**	**UCI**	**Odds Ratio**	**LCI**	**UCI**	**Odds Ratio**	**LCI**	**UCI**
Gender	Male	(ref)	(ref)	(ref)	(ref)	(ref)	(ref)	(ref)	(ref)	(ref)	(ref)	(ref)	(ref)	(ref)	(ref)	(ref)
	Female	0.807	0.714	0.913	0.765	0.680	0.860	0.780	0.700	0.869	0.839	0.752	0.935	0.711	0.635	0.797
Ethnicity	White - British	(ref)	(ref)	(ref)	(ref)	(ref)	(ref)	(ref)	(ref)	(ref)	(ref)	(ref)	(ref)	(ref)	(ref)	(ref)
	Any other White background	0.880	0.551	1.404	0.986	0.679	1.433	1.154	0.830	1.604	1.000	0.698	1.433	0.786	0.542	1.140
	Asian or Asian British	0.835	0.330	2.112	1.134	0.614	2.097	1.031	0.487	2.183	1.071	0.526	2.181	1.071	0.563	2.039
	Mixed/Dual background	1.962	1.246	3.090	0.960	0.586	1.573	1.049	0.685	1.607	0.942	0.622	1.426	0.949	0.604	1.490
	Any other ethnic group	1.179	0.530	2.625	1.630	0.791	3.359	1.355	0.757	2.426	1.285	0.737	2.240	0.618	0.284	1.344
SES	IMD 2010	2.893	1.611	5.196	2.992	1.704	5.256	3.052	1.847	5.043	3.977	2.371	6.669	4.059	2.394	6.880
NCMP participation (non-participation >0.2)		1.069	0.899	1.270	0.996	0.841	1.181	1.093	0.898	1.330	0.745	0.604	0.920	1.257	0.948	1.667
**Factor**		**Odds Ratio**	**LCI**	**UCI**	**Odds Ratio**	**LCI**	**UCI**	**Odds Ratio**	**LCI**	**UCI**	**Odds Ratio**	**LCI**	**UCI**	**Odds Ratio**	**LCI**	**UCI**
Gender	Male	(ref)	(ref)	(ref)	(ref)	(ref)	(ref)	(ref)	(ref)	(ref)	(ref)	(ref)	(ref)	(ref)	(ref)	(ref)
	Female	0.807	0.713	0.913	0.765	0.680	0.860	0.780	0.700	0.869	0.839	0.752	0.935	0.711	0.635	0.797
Ethnicity	White - British	(ref)	(ref)	(ref)	(ref)	(ref)	(ref)	(ref)	(ref)	(ref)	(ref)	(ref)	(ref)	(ref)	(ref)	(ref)
	Any other White background	0.888	0.556	1.418	0.982	0.676	1.427	1.152	0.829	1.601	1.003	0.700	1.437	0.786	0.542	1.140
	Asian or Asian British	0.845	0.334	2.137	1.124	0.608	2.079	1.028	0.485	2.177	1.080	0.530	2.200	1.073	0.563	2.044
	Mixed/Dual background	1.969	1.251	3.102	0.958	0.585	1.571	1.047	0.684	1.604	0.945	0.624	1.431	0.949	0.604	1.490
	Any other ethnic group	1.191	0.535	2.652	1.617	0.785	3.332	1.353	0.756	2.421	1.292	0.741	2.253	0.619	0.285	1.345
SES	IMD 2010	2.990	1.658	5.393	2.913	1.654	5.130	3.013	1.817	4.997	4.090	2.424	6.900	4.074	2.393	6.935
NCMP participation (non-participation >0.2)		1.066	0.898	1.267	1.000	0.844	1.186	1.093	0.898	1.331	0.748	0.605	0.923	1.257	0.948	1.667
Factor 3		0.922	0.791	1.076	1.066	0.930	1.222	1.027	0.905	1.165	0.954	0.839	1.085	0.993	0.867	1.137

**Reception two-level**		**2006/07**			**2007/08**			**2008/09**			**2009/10**			**2010/11**		
n		4352			5190			5421			5373			4970		
Log likelihood	**Null**	-1217.69			-1459.60			-1732.98			-1560.98			-1507.99		
	**Individual**	-1210.86			-1447.28			-1727.23			-1551.17			-1497.76		
	**Factor**	-1210.85			-1447.01			-1727.15			-1550.84			-1497.74		
School variance	**Null**	3.47E-20	0	.	0.091	0.023	0.364	0.145	0.071	0.298	0.305	0.175	0.530	0.106	0.032	0.351
	**Individual**	8.49E-13	0	.	0.049	0.004	0.568	0.136	0.064	0.288	0.235	0.123	0.450	0.105	0.032	0.338
	**Factor**	4.94E-13	0	.	0.048	0.004	0.599	0.136	0.064	0.288	0.234	0.122	0.449	0.104	0.032	0.339
**Individual**		**Odds Ratio**	**LCI**	**UCI**	**Odds Ratio**	**LCI**	**UCI**	**Odds Ratio**	**LCI**	**UCI**	**Odds Ratio**	**LCI**	**UCI**	**Odds Ratio**	**LCI**	**UCI**
Gender	Male	(ref)	(ref)	(ref)	(ref)	(ref)	(ref)	(ref)	(ref)	(ref)	(ref)	(ref)	(ref)	(ref)	(ref)	(ref)
	Female	0.824	0.661	1.027	0.821	0.671	1.005	0.797	0.664	0.956	0.764	0.628	0.930	0.703	0.577	0.858
Ethnicity	White - British	(ref)	(ref)	(ref)	(ref)	(ref)	(ref)	(ref)	(ref)	(ref)	(ref)	(ref)	(ref)	(ref)	(ref)	(ref)
	Any other White background	1.028	0.533	1.985	1.299	0.791	2.133	1.151	0.672	1.971	0.665	0.331	1.338	1.077	0.640	1.812
	Asian or Asian British	1.996	0.680	5.860	0.897	0.315	2.553	0.626	0.144	2.717	1.477	0.556	3.927	0.919	0.322	2.618
	Mixed/Dual background	1.872	0.834	4.203	0.443	0.139	1.415	1.082	0.569	2.056	1.064	0.560	2.020	1.135	0.581	2.217
	Any other ethnic group	0.405	0.055	2.989	0.495	0.066	3.733	1.091	0.378	3.150	0.758	0.227	2.533	1.222	0.426	3.502
SES	IMD 2010	3.599	1.308	9.900	7.312	2.861	18.687	2.478	1.010	6.082	4.185	1.559	11.238	3.945	1.511	10.298
NCMP participation (non-participation >0.2)		1.010	0.726	1.405	0.843	0.592	1.199	1.276	0.781	2.083	0.702	0.467	1.057	1.236	0.537	2.842
**Factor**		**Odds Ratio**	**LCI**	**UCI**	**Odds Ratio**	**LCI**	**UCI**	**Odds Ratio**	**LCI**	**UCI**	**Odds Ratio**	**LCI**	**UCI**	**Odds Ratio**	**LCI**	**UCI**
Gender	Male	(ref)	(ref)	(ref)	(ref)	(ref)	(ref)	(ref)	(ref)	(ref)	(ref)	(ref)	(ref)	(ref)	(ref)	(ref)
	Female	0.824	0.661	1.028	0.821	0.671	1.005	0.797	0.664	0.956	0.764	0.628	0.931	0.704	0.577	0.858
Ethnicity	White - British	(ref)	(ref)	(ref)	(ref)	(ref)	(ref)	(ref)	(ref)	(ref)	(ref)	(ref)	(ref)	(ref)	(ref)	(ref)
	Any other White background	1.026	0.531	1.982	1.296	0.789	2.128	1.149	0.671	1.968	0.665	0.331	1.336	1.076	0.639	1.810
	Asian or Asian British	1.991	0.678	5.849	0.889	0.312	2.530	0.622	0.143	2.698	1.465	0.551	3.893	0.916	0.321	2.611
	Mixed/Dual background	1.870	0.833	4.199	0.443	0.139	1.415	1.080	0.568	2.051	1.059	0.557	2.010	1.134	0.581	2.216
	Any other ethnic group	0.403	0.055	2.982	0.494	0.066	3.723	1.090	0.378	3.145	0.751	0.225	2.509	1.220	0.425	3.497
SES	IMD 2010	3.571	1.288	9.903	6.960	2.706	17.902	2.427	0.984	5.990	3.967	1.468	10.718	3.900	1.483	10.259
NCMP participation (non-participation >0.2)		1.010	0.726	1.406	0.853	0.599	1.215	1.268	0.776	2.073	0.699	0.464	1.052	1.232	0.535	2.834
Factor 3		1.017	0.769	1.345	1.104	0.850	1.434	1.051	0.819	1.349	1.123	0.848	1.488	1.024	0.787	1.334

**Year 6 two-level**		**2006/07**			**2007/08**			**2008/09**			**2009/10**			**2010/11**		
n		5558			6163			6275			6478			6046		
Log likelihood	**Null**	-2312.74			-2506.79			-2771.22			-2833.62			-2648.44		
	**Individual**	-2299.05			-2494.93			-2756.94			-2825.60			-2624.68		
	**Factor**	-2298.13			-2494.77			-2756.93			-2825.21			-2624.40		
School variance	**Null**	0.027	0.003	0.261	0.089	0.037	0.218	0.092	0.043	0.197	0.074	0.030	0.179	0.033	0.006	0.173
	**Individual**	0.014	2.27E-04	0.906	0.086	0.034	0.217	0.075	0.032	0.177	0.066	0.025	0.174	0.005	3.68E-07	71.441
	**Factor**	0.005	1.07E-07	272.466	0.087	0.035	0.218	0.075	0.032	0.177	0.064	0.024	0.174	0.005	9.91E-08	208.307
**Individual**		**Odds Ratio**	**LCI**	**UCI**	**Odds Ratio**	**LCI**	**UCI**	**Odds Ratio**	**LCI**	**UCI**	**Odds Ratio**	**LCI**	**UCI**	**Odds Ratio**	**LCI**	**UCI**
Gender	Male	(ref)	(ref)	(ref)	(ref)	(ref)	(ref)	(ref)	(ref)	(ref)	(ref)	(ref)	(ref)	(ref)	(ref)	(ref)
	Female	0.794	0.683	0.923	0.737	0.636	0.854	0.778	0.679	0.893	0.886	0.774	1.014	0.717	0.623	0.826
Ethnicity	White - British	(ref)	(ref)	(ref)	(ref)	(ref)	(ref)	(ref)	(ref)	(ref)	(ref)	(ref)	(ref)	(ref)	(ref)	(ref)
	Any other White background	0.986	0.502	1.935	0.881	0.487	1.594	1.201	0.782	1.846	1.288	0.831	1.997	0.683	0.395	1.178
	Asian or Asian British	0.266	0.036	1.977	1.319	0.599	2.908	1.213	0.490	3.000	0.897	0.306	2.628	1.351	0.581	3.140
	Mixed/Dual background	2.028	1.160	3.545	1.359	0.768	2.406	1.106	0.614	1.992	1.001	0.570	1.756	0.944	0.507	1.759
	Any other ethnic group	2.111	0.826	5.395	2.222	0.970	5.088	1.588	0.769	3.282	1.571	0.816	3.024	0.367	0.113	1.188
SES	IMD 2010	2.999	1.428	6.296	1.766	0.804	3.879	3.418	1.709	6.835	3.201	1.569	6.532	4.870	2.500	9.487
NCMP participation (non-participation >0.2)		1.030	0.836	1.270	0.936	0.746	1.173	0.870	0.680	1.113	1.007	0.754	1.346	1.041	0.766	1.415
**Factor**		**Odds Ratio**	**LCI**	**UCI**	**Odds Ratio**	**LCI**	**UCI**	**Odds Ratio**	**LCI**	**UCI**	**Odds Ratio**	**LCI**	**UCI**	**Odds Ratio**	**LCI**	**UCI**
Gender	Male	(ref)	(ref)	(ref)	(ref)	(ref)	(ref)	(ref)	(ref)	(ref)	(ref)	(ref)	(ref)	(ref)	(ref)	(ref)
	Female	0.795	0.684	0.925	0.737	0.636	0.854	0.778	0.679	0.893	0.886	0.775	1.015	0.717	0.623	0.825
Ethnicity	White - British	(ref)	(ref)	(ref)	(ref)	(ref)	(ref)	(ref)	(ref)	(ref)	(ref)	(ref)	(ref)	(ref)	(ref)	(ref)
	Any other White background	0.992	0.505	1.947	0.876	0.484	1.584	1.200	0.781	1.845	1.293	0.834	2.006	0.683	0.396	1.179
	Asian or Asian British	0.269	0.036	1.998	1.312	0.595	2.892	1.213	0.490	2.999	0.909	0.310	2.666	1.376	0.591	3.202
	Mixed/Dual background	2.030	1.163	3.546	1.356	0.766	2.401	1.105	0.614	1.991	1.006	0.573	1.764	0.947	0.508	1.763
	Any other ethnic group	2.131	0.835	5.440	2.206	0.963	5.055	1.587	0.768	3.280	1.579	0.820	3.041	0.368	0.114	1.190
SES	IMD 2010	3.173	1.513	6.652	1.740	0.791	3.829	3.404	1.697	6.828	3.318	1.619	6.800	5.040	2.573	9.875
NCMP participation (non-participation >0.2)		1.028	0.837	1.264	0.937	0.747	1.175	0.870	0.680	1.113	1.011	0.757	1.352	1.038	0.764	1.411
Factor 3		0.876	0.725	1.060	1.055	0.875	1.272	1.011	0.846	1.207	0.924	0.777	1.100	0.939	0.795	1.109

**Three-level**		**2006/07**			**2007/08**			**2008/09**			**2009/10**			**2010/11**		
n		9910			11353			11696			11851			11016		
Log likelihood	**Null**	-3574.27			-4002.79			-4542.37			-4448.49			-4200.78		
	**Individual**	-3559.41			-3986.74			-4524.94			-4435.32			-4171.06		
	**Factor**	-3559.19			-3986.17			-4524.88			-4435.21			-4171.03		
	**Interaction**	-3511.99			-3946.67			-4485.21			-4382.49			-4124.81		
School variance	**Null**	2.61E-18	0	.	2.56E-14	0	.	1.74E-13	0	.	2.13E-17	0	.	1.23E-14	0	.
	**Individual**	2.45E-21	0	.	6.01E-11	0	.	8.58E-11	0	.	7.45E-20	0	.	1.42E-12	0	.
	**Factor**	1.28E-12	0	.	9.07E-12	0	.	2.12E-17	0	.	1.55E-19	0	.	2.14E-19	0	.
	**Interaction**	0.021	0.002	0.199	0.062	0.020	0.190	0.043	0.011	0.171	0.041	0.009	0.199	0.031	0.009	0.112
Year group variai	**Null**	0.122	0.066	0.226	0.191	0.123	0.296	0.182	0.122	0.273	0.261	0.183	0.372	0.152	0.095	0.245
	**Individual**	0.114	0.059	0.219	0.181	0.114	0.286	0.167	0.109	0.255	0.233	0.159	0.341	0.132	0.079	0.222
	**Factor**	0.112	0.058	0.218	0.181	0.115	0.286	0.167	0.109	0.254	0.233	0.159	0.341	0.132	0.079	0.222
	**Interaction**	9.82E-14	0	.	0.020	0.000	0.867	0.056	0.016	0.198	0.078	0.026	0.229	9.18E-10	0	.
**Individual**		**Odds Ratio**	**LCI**	**UCI**	**Odds Ratio**	**LCI**	**UCI**	**Odds Ratio**	**LCI**	**UCI**	**Odds Ratio**	**LCI**	**UCI**	**Odds Ratio**	**LCI**	**UCI**
Gender	Male	(ref)	(ref)	(ref)	(ref)	(ref)	(ref)	(ref)	(ref)	(ref)	(ref)	(ref)	(ref)	(ref)	(ref)	(ref)
	Female	0.800	0.706	0.906	0.758	0.673	0.854	0.783	0.702	0.874	0.839	0.751	0.938	0.707	0.630	0.793
Ethnicity	White - British	(ref)	(ref)	(ref)	(ref)	(ref)	(ref)	(ref)	(ref)	(ref)	(ref)	(ref)	(ref)	(ref)	(ref)	(ref)
	Any other White background	0.915	0.569	1.470	1.011	0.687	1.487	1.159	0.828	1.623	1.031	0.713	1.490	0.807	0.553	1.179
	Asian or Asian British	0.866	0.339	2.214	1.069	0.568	2.013	0.966	0.450	2.074	1.129	0.546	2.334	1.104	0.574	2.124
	Mixed/Dual background	1.991	1.254	3.159	0.983	0.595	1.623	1.066	0.691	1.645	1.014	0.664	1.548	0.965	0.610	1.524
	Any other ethnic group	1.242	0.550	2.806	1.543	0.735	3.241	1.392	0.767	2.526	1.313	0.741	2.327	0.621	0.283	1.361
SES	IMD 2010	2.669	1.413	5.042	2.756	1.471	5.167	2.895	1.646	5.094	3.216	1.762	5.871	3.856	2.156	6.895
NCMP participation (non-participation >0.2)		1.053	0.866	1.281	0.957	0.779	1.177	1.079	0.852	1.366	0.824	0.639	1.063	1.227	0.887	1.698
**Factor**		**Odds Ratio**	**LCI**	**UCI**	**Odds Ratio**	**LCI**	**UCI**	**Odds Ratio**	**LCI**	**UCI**	**Odds Ratio**	**LCI**	**UCI**	**Odds Ratio**	**LCI**	**UCI**
Gender	Male	(ref)	(ref)	(ref)	(ref)	(ref)	(ref)	(ref)	(ref)	(ref)	(ref)	(ref)	(ref)	(ref)	(ref)	(ref)
	Female	0.800	0.706	0.906	0.758	0.673	0.854	0.783	0.702	0.874	0.839	0.751	0.938	0.707	0.630	0.793
Ethnicity	White - British	(ref)	(ref)	(ref)	(ref)	(ref)	(ref)	(ref)	(ref)	(ref)	(ref)	(ref)	(ref)	(ref)	(ref)	(ref)
	Any other White background	0.920	0.572	1.478	1.006	0.683	1.480	1.158	0.827	1.622	1.032	0.714	1.492	0.808	0.553	1.179
	Asian or Asian British	0.871	0.341	2.226	1.062	0.564	1.999	0.964	0.449	2.070	1.133	0.548	2.343	1.108	0.576	2.131
	Mixed/Dual background	1.994	1.256	3.163	0.981	0.594	1.621	1.064	0.690	1.643	1.016	0.665	1.551	0.965	0.610	1.525
	Any other ethnic group	1.247	0.552	2.817	1.534	0.730	3.221	1.391	0.766	2.523	1.317	0.743	2.332	0.621	0.284	1.362
SES	IMD 2010	2.725	1.438	5.163	2.684	1.430	5.038	2.869	1.627	5.059	3.265	1.782	5.980	3.889	2.166	6.981
NCMP participation (non-participation >0.2)		1.051	0.864	1.279	0.963	0.783	1.184	1.079	0.852	1.366	0.826	0.640	1.064	1.228	0.887	1.699
Factor 3		0.942	0.790	1.123	1.094	0.927	1.291	1.027	0.879	1.200	0.962	0.814	1.136	0.980	0.837	1.147
**Interaction**		**Odds Ratio**	**LCI**	**UCI**	**Odds Ratio**	**LCI**	**UCI**	**Odds Ratio**	**LCI**	**UCI**	**Odds Ratio**	**LCI**	**UCI**	**Odds Ratio**	**LCI**	**UCI**
Gender	Male	(ref)	(ref)	(ref)	(ref)	(ref)	(ref)	(ref)	(ref)	(ref)	(ref)	(ref)	(ref)	(ref)	(ref)	(ref)
	Female	0.803	0.709	0.909	0.764	0.679	0.861	0.785	0.703	0.875	0.840	0.751	0.939	0.712	0.635	0.799
Ethnicity	White - British	(ref)	(ref)	(ref)	(ref)	(ref)	(ref)	(ref)	(ref)	(ref)	(ref)	(ref)	(ref)	(ref)	(ref)	(ref)
	Any other White background	1.013	0.631	1.625	1.119	0.763	1.640	1.180	0.844	1.651	1.052	0.729	1.518	0.857	0.588	1.247
	Asian or Asian British	0.900	0.354	2.290	1.118	0.596	2.099	0.984	0.459	2.109	1.196	0.579	2.471	1.181	0.616	2.266
	Mixed/Dual background	1.981	1.251	3.137	0.999	0.606	1.647	1.095	0.710	1.689	1.048	0.687	1.600	1.018	0.645	1.607
	Any other ethnic group	1.295	0.576	2.911	1.552	0.742	3.247	1.392	0.768	2.524	1.308	0.741	2.311	0.618	0.282	1.351
SES	IMD 2010	3.165	1.699	5.895	2.813	1.507	5.252	2.890	1.645	5.078	3.499	1.932	6.337	4.295	2.441	7.560
NCMP participation (non-participation >0.2)		1.028	0.859	1.229	0.914	0.757	1.105	0.920	0.734	1.154	0.858	0.677	1.087	1.061	0.790	1.427
Factor 3		1.019	0.769	1.351	1.147	0.881	1.494	1.045	0.822	1.330	1.112	0.859	1.440	1.033	0.806	1.324
Year group	Reception	(ref)	(ref)	(ref)	(ref)	(ref)	(ref)	(ref)	(ref)	(ref)	(ref)	(ref)	(ref)	(ref)	(ref)	(ref)
	Year 6	2.638	1.325	5.250	2.271	1.184	4.357	1.894	1.024	3.501	3.012	1.561	5.812	2.277	1.227	4.227
Year group*factor	Reception	(ref)	(ref)	(ref)	(ref)	(ref)	(ref)	(ref)	(ref)	(ref)	(ref)	(ref)	(ref)	(ref)	(ref)	(ref)
	Year 6	0.866	0.619	1.212	0.913	0.671	1.243	0.981	0.732	1.313	0.834	0.613	1.135	0.917	0.683	1.231

Supporting file 3 to Williams, A.J., Wyatt, K.M., Williams, C.A., Logan, S., and Henley, W.E. Exploring the potential of a school impact on pupil weight status: exploratory factor analysis and repeat cross-sectional study of the National Child Measurement Programme.

S3 file - Detailed results and sensitivity analysis

Factor 3 - Resource

Red shading indicates significance (p<0.05), Green shading indicates modles which had to be estimated in R

Overweight (IOTF)

Single level

		2006/07			2007/08			2008/09			2009/10			2010/11		
n		9532			10955			11217			11345			10559		
Log likelihood	**Null**	-4160.76			-4786.96			-5421.59			-5292.83			-4916.86		
	**Individual**	-4143.36			-4767.80			-5401.96			-5271.96			-4908.28		
	**Factor**	-4143.36			-4765.69			-5401.70			-5270.58			-4908.24		
**Individual**		**Odds Ratio**	**LCI**	**UCI**	**Odds Ratio**	**LCI**	**UCI**	**Odds Ratio**	**LCI**	**UCI**	**Odds Ratio**	**LCI**	**UCI**	**Odds Ratio**	**LCI**	**UCI**
Gender	Male	(ref)	(ref)	(ref)	(ref)	(ref)	(ref)	(ref)	(ref)	(ref)	(ref)	(ref)	(ref)	(ref)	(ref)	(ref)
	Female	1.319	1.181	1.473	1.322	1.193	1.466	1.288	1.171	1.417	1.346	1.222	1.483	1.129	1.021	1.248
Ethnicity	White - British	(ref)	(ref)	(ref)	(ref)	(ref)	(ref)	(ref)	(ref)	(ref)	(ref)	(ref)	(ref)	(ref)	(ref)	(ref)
	Any other White background	1.047	0.705	1.554	0.807	0.563	1.157	1.115	0.830	1.499	1.071	0.781	1.467	1.089	0.808	1.467
	Asian or Asian British	0.738	0.312	1.744	0.779	0.411	1.475	0.910	0.458	1.809	0.964	0.486	1.912	0.761	0.388	1.490
	Mixed/Dual background	1.409	0.886	2.241	0.721	0.445	1.169	1.085	0.746	1.578	0.896	0.615	1.305	0.951	0.631	1.432
	Any other ethnic group	1.348	0.670	2.710	1.031	0.481	2.209	0.950	0.519	1.737	1.061	0.623	1.806	0.516	0.248	1.073
SES	IMD 2010	2.098	1.225	3.592	1.829	1.095	3.054	2.124	1.345	3.353	1.694	1.049	2.735	1.368	0.831	2.253
NCMP participation (non-participation >0.2)		1.071	0.917	1.250	1.077	0.930	1.247	1.099	0.923	1.308	0.963	0.810	1.144	1.358	1.058	1.743
**Factor**		**Odds Ratio**	**LCI**	**UCI**	**Odds Ratio**	**LCI**	**UCI**	**Odds Ratio**	**LCI**	**UCI**	**Odds Ratio**	**LCI**	**UCI**	**Odds Ratio**	**LCI**	**UCI**
Gender	Male	(ref)	(ref)	(ref)	(ref)	(ref)	(ref)	(ref)	(ref)	(ref)	(ref)	(ref)	(ref)	(ref)	(ref)	(ref)
	Female	1.319	1.181	1.473	1.323	1.194	1.467	1.288	1.171	1.416	1.346	1.222	1.483	1.129	1.021	1.248
Ethnicity	White - British	(ref)	(ref)	(ref)	(ref)	(ref)	(ref)	(ref)	(ref)	(ref)	(ref)	(ref)	(ref)	(ref)	(ref)	(ref)
	Any other White background	1.047	0.705	1.555	0.800	0.558	1.147	1.119	0.832	1.504	1.076	0.785	1.475	1.088	0.808	1.466
	Asian or Asian British	0.738	0.312	1.744	0.766	0.404	1.451	0.915	0.460	1.818	0.984	0.496	1.952	0.758	0.387	1.485
	Mixed/Dual background	1.409	0.886	2.241	0.718	0.443	1.165	1.088	0.748	1.582	0.902	0.619	1.314	0.950	0.631	1.432
	Any other ethnic group	1.348	0.670	2.711	1.011	0.472	2.167	0.952	0.521	1.743	1.073	0.631	1.828	0.515	0.248	1.072
SES	IMD 2010	2.098	1.222	3.601	1.737	1.038	2.906	2.167	1.367	3.435	1.785	1.101	2.895	1.357	0.822	2.242
NCMP participation (non-participation >0.2)		1.071	0.917	1.251	1.086	0.937	1.258	1.098	0.923	1.307	0.969	0.815	1.152	1.358	1.058	1.743
Factor 3		1.000	0.872	1.146	1.135	1.006	1.280	0.959	0.858	1.073	0.907	0.809	1.018	1.018	0.902	1.148

Reception two-level

		2006/07			2007/08			2008/09			2009/10			2010/11		
n		4214			5017			5199			5165			4783		
Log likelihood	**Null**	-1716.68			-1998.43			-2399.35			-2231.13			-2109.18		
	**Individual**	-1704.54			-1975.03			-2381.45			-2215.84			-2098.70		
	**Factor**	-1703.79			-1974.07			-2381.18			-2215.76			-2098.69		
School variance	**Null**	0.045	0.007	0.277	0.097	0.038	0.250	0.127	0.071	0.229	0.037	0.005	0.276	0.057	0.015	0.209
	**Individual**	0.030	0.002	0.452	0.080	0.027	0.242	0.120	0.065	0.220	0.036	0.005	0.285	0.065	0.020	0.210
	**Factor**	0.025	0.001	0.623	0.073	0.022	0.240	0.120	0.065	0.219	0.037	0.005	0.282	0.065	0.020	0.212
**Individual**		**Odds Ratio**	**LCI**	**UCI**	**Odds Ratio**	**LCI**	**UCI**	**Odds Ratio**	**LCI**	**UCI**	**Odds Ratio**	**LCI**	**UCI**	**Odds Ratio**	**LCI**	**UCI**
Gender	Male	(ref)	(ref)	(ref)	(ref)	(ref)	(ref)	(ref)	(ref)	(ref)	(ref)	(ref)	(ref)	(ref)	(ref)	(ref)
	Female	1.510	1.267	1.800	1.521	1.290	1.793	1.467	1.267	1.699	1.471	1.262	1.713	1.355	1.159	1.585
Ethnicity	White - British	(ref)	(ref)	(ref)	(ref)	(ref)	(ref)	(ref)	(ref)	(ref)	(ref)	(ref)	(ref)	(ref)	(ref)	(ref)
	Any other White background	0.913	0.525	1.589	0.704	0.418	1.183	0.835	0.515	1.353	1.174	0.746	1.846	1.338	0.899	1.992
	Asian or Asian British	1.150	0.385	3.431	0.266	0.063	1.112	0.676	0.226	2.019	0.578	0.174	1.920	0.529	0.185	1.508
	Mixed/Dual background	1.236	0.572	2.674	0.334	0.121	0.922	1.189	0.722	1.959	0.877	0.513	1.502	1.270	0.751	2.149
	Any other ethnic group	1.004	0.343	2.937	0.563	0.129	2.454	1.251	0.534	2.928	0.929	0.385	2.246	0.486	0.148	1.602
SES	IMD 2010	2.056	0.862	4.905	3.447	1.515	7.841	1.738	0.810	3.728	1.793	0.845	3.802	1.169	0.519	2.632
NCMP participation (non-participation >0.2)		1.005	0.767	1.317	0.917	0.687	1.224	1.633	1.106	2.409	0.830	0.636	1.082	1.347	0.691	2.629
**Factor**		**Odds Ratio**	**LCI**	**UCI**	**Odds Ratio**	**LCI**	**UCI**	**Odds Ratio**	**LCI**	**UCI**	**Odds Ratio**	**LCI**	**UCI**	**Odds Ratio**	**LCI**	**UCI**
Gender	Male	(ref)	(ref)	(ref)	(ref)	(ref)	(ref)	(ref)	(ref)	(ref)	(ref)	(ref)	(ref)	(ref)	(ref)	(ref)
	Female	1.514	1.271	1.805	1.521	1.290	1.792	1.467	1.267	1.699	1.470	1.262	1.713	1.355	1.159	1.585
Ethnicity	White - British	(ref)	(ref)	(ref)	(ref)	(ref)	(ref)	(ref)	(ref)	(ref)	(ref)	(ref)	(ref)	(ref)	(ref)	(ref)
	Any other White background	0.892	0.512	1.554	0.699	0.416	1.176	0.837	0.517	1.357	1.175	0.747	1.848	1.338	0.899	1.991
	Asian or Asian British	1.126	0.378	3.359	0.262	0.063	1.095	0.683	0.228	2.042	0.580	0.174	1.929	0.528	0.185	1.506
	Mixed/Dual background	1.218	0.563	2.634	0.334	0.121	0.920	1.193	0.724	1.965	0.879	0.514	1.505	1.270	0.751	2.148
	Any other ethnic group	0.982	0.336	2.871	0.561	0.129	2.444	1.253	0.535	2.933	0.934	0.386	2.258	0.486	0.147	1.601
SES	IMD 2010	1.972	0.829	4.693	3.227	1.419	7.342	1.790	0.831	3.858	1.831	0.857	3.911	1.163	0.515	2.629
NCMP participation (non-participation >0.2)		1.010	0.771	1.321	0.936	0.701	1.249	1.649	1.116	2.436	0.832	0.638	1.086	1.345	0.689	2.625
Factor 3		1.154	0.918	1.452	1.169	0.939	1.455	0.926	0.754	1.137	0.960	0.788	1.170	1.013	0.820	1.251

Year 6 two-level

		2006/07			2007/08			2008/09			2009/10			2010/11		
n		5318			5938			6018			6180			5776		
Log likelihood	**Null**	-2435.38			-2765.54			-3002.77			-3044.09			-2797.82		
	**Individual**	-2426.60			-2760.43			-2993.90			-3034.06			-2794.09		
	**Factor**	-2426.06			-2759.83			-2993.72			-3033.21			-2794.08		
School variance	**Null**	7.39E-16	0	.	0.075	0.029	0.196	0.051	0.017	0.155	0.049	0.016	0.153	0.038	0.009	0.160
	**Individual**	4.22E-16	0	.	0.071	0.026	0.194	0.050	0.016	0.152	0.046	0.014	0.153	0.031	0.005	0.172
	**Factor**	1.07E-23	0	.	0.071	0.026	0.192	0.050	0.016	0.151	0.042	0.011	0.155	0.031	0.006	0.172
**Individual**		**Odds Ratio**	**LCI**	**UCI**	**Odds Ratio**	**LCI**	**UCI**	**Odds Ratio**	**LCI**	**UCI**	**Odds Ratio**	**LCI**	**UCI**	**Odds Ratio**	**LCI**	**UCI**
Gender	Male	(ref)	(ref)	(ref)	(ref)	(ref)	(ref)	(ref)	(ref)	(ref)	(ref)	(ref)	(ref)	(ref)	(ref)	(ref)
	Female	1.202	1.042	1.387	1.219	1.065	1.395	1.185	1.043	1.346	1.274	1.122	1.447	0.996	0.872	1.137
Ethnicity	White - British	(ref)	(ref)	(ref)	(ref)	(ref)	(ref)	(ref)	(ref)	(ref)	(ref)	(ref)	(ref)	(ref)	(ref)	(ref)
	Any other White background	1.384	0.777	2.464	1.076	0.638	1.812	1.386	0.939	2.045	1.037	0.664	1.619	0.920	0.576	1.469
	Asian or Asian British	0.435	0.102	1.856	1.376	0.644	2.941	1.053	0.424	2.616	1.450	0.606	3.472	1.066	0.432	2.632
	Mixed/Dual background	1.533	0.854	2.752	1.109	0.625	1.969	0.942	0.522	1.698	0.964	0.564	1.648	0.691	0.352	1.358
	Any other ethnic group	1.905	0.736	4.933	1.369	0.542	3.456	0.765	0.316	1.852	1.176	0.596	2.322	0.544	0.213	1.388
SES	IMD 2010	2.182	1.074	4.435	1.025	0.485	2.163	2.423	1.264	4.645	1.758	0.888	3.480	1.538	0.775	3.052
NCMP participation (non-participation >0.2)		1.083	0.892	1.315	1.092	0.893	1.336	0.904	0.723	1.131	1.246	0.962	1.615	1.285	0.963	1.714
**Factor**		**Odds Ratio**	**LCI**	**UCI**	**Odds Ratio**	**LCI**	**UCI**	**Odds Ratio**	**LCI**	**UCI**	**Odds Ratio**	**LCI**	**UCI**	**Odds Ratio**	**LCI**	**UCI**
Gender	Male	(ref)	(ref)	(ref)	(ref)	(ref)	(ref)	(ref)	(ref)	(ref)	(ref)	(ref)	(ref)	(ref)	(ref)	(ref)
	Female	1.203	1.043	1.388	1.219	1.065	1.395	1.184	1.042	1.346	1.275	1.123	1.447	0.996	0.872	1.137
Ethnicity	White - British	(ref)	(ref)	(ref)	(ref)	(ref)	(ref)	(ref)	(ref)	(ref)	(ref)	(ref)	(ref)	(ref)	(ref)	(ref)
	Any other White background	1.391	0.781	2.476	1.064	0.631	1.793	1.391	0.943	2.054	1.042	0.667	1.628	0.920	0.576	1.469
	Asian or Asian British	0.439	0.103	1.873	1.366	0.639	2.918	1.056	0.425	2.622	1.483	0.619	3.550	1.069	0.432	2.642
	Mixed/Dual background	1.538	0.857	2.761	1.106	0.623	1.962	0.945	0.524	1.704	0.970	0.567	1.659	0.692	0.352	1.359
	Any other ethnic group	1.926	0.743	4.988	1.349	0.534	3.406	0.768	0.317	1.860	1.182	0.599	2.332	0.544	0.213	1.389
SES	IMD 2010	2.253	1.105	4.595	1.001	0.474	2.114	2.468	1.283	4.748	1.840	0.928	3.650	1.545	0.776	3.080
NCMP participation (non-participation >0.2)		1.081	0.890	1.312	1.095	0.895	1.339	0.902	0.721	1.128	1.253	0.968	1.621	1.285	0.963	1.714
Factor 3		0.912	0.765	1.086	1.100	0.927	1.307	0.951	0.809	1.119	0.899	0.767	1.055	0.990	0.840	1.167

Three-level

		2006/07			2007/08			2008/09			2009/10			2010/11		
n		9532			10955			11217			11345			10559		
Log likelihood	**Null**	-4160.65			-4774.86			-5407.81			-5287.50			-4912.78		
	**Individual**	-4143.36			-4756.93			-5388.58			-5266.85			-4904.63		
	**Factor**	-4143.36			-4755.28			-5388.24			-5265.85			-4904.63		
	**Interaction**	-4133.89			-4743.88			-5383.90			-5252.02			-4899.03		
School variance	**Null**	8.23E-11	0	.	1.53E-07	0	.	2.04E-10	0	.	0.017	0.001	0.314	0.009	4.04E-05	1.870
	**Individual**	4.56E-17	0	.	7.04E-10	0	.	2.13E-15	0	.	0.014	4.15E-04	0.475	0.014	4.61E-04	0.399
	**Factor**	3.80E-15	0	.	4.57E-15	0	.	2.91E-12	0	.	0.015	0.001	0.384	0.014	4.62E-04	0.398
	**Interaction**	3.38E-12	0	.	0.018	0.001	0.406	3.80E-09	0	.	0.038	0.010886	0.130	0.023	3.14E-03	0.165
Year group variai	**Null**	9.30E-03	1.07E-04	0.813	0.115	0.067	0.196	0.095	0.057	0.156	0.048	0.013	0.183	0.047	0.013	0.168
	**Individual**	2.04E-14	0	.	0.109	0.062	0.190	0.093	0.056	0.154	0.050	0.014	0.184	0.038	0.008	0.172
	**Factor**	6.54E-12	0	.	0.105	0.060	0.186	0.093	0.056	0.154	0.047	0.012	0.187	0.038	0.008	0.172
	**Interaction**	7.97E-14	0	.	0.056	0.015	0.206	0.088	0.052	0.148	0.005	4.65E-08	479.997	0.020	0.001	0.300
**Individual**		**Odds Ratio**	**LCI**	**UCI**	**Odds Ratio**	**LCI**	**UCI**	**Odds Ratio**	**LCI**	**UCI**	**Odds Ratio**	**LCI**	**UCI**	**Odds Ratio**	**LCI**	**UCI**
Gender	Male	(ref)	(ref)	(ref)	(ref)	(ref)	(ref)	(ref)	(ref)	(ref)	(ref)	(ref)	(ref)	(ref)	(ref)	(ref)
	Female	1.319	1.181	1.473	1.331	1.200	1.478	1.301	1.181	1.432	1.350	1.225	1.489	1.130	1.021	1.250
Ethnicity	White - British	(ref)	(ref)	(ref)	(ref)	(ref)	(ref)	(ref)	(ref)	(ref)	(ref)	(ref)	(ref)	(ref)	(ref)	(ref)
	Any other White background	1.047	0.705	1.554	0.834	0.577	1.205	1.107	0.819	1.496	1.098	0.799	1.511	1.105	0.817	1.494
	Asian or Asian British	0.738	0.312	1.744	0.769	0.403	1.469	0.874	0.435	1.757	0.965	0.483	1.925	0.743	0.377	1.463
	Mixed/Dual background	1.409	0.886	2.241	0.727	0.446	1.184	1.076	0.736	1.574	0.909	0.622	1.329	0.958	0.634	1.448
	Any other ethnic group	1.348	0.670	2.710	1.000	0.462	2.166	0.961	0.521	1.772	1.084	0.633	1.858	0.517	0.248	1.080
SES	IMD 2010	2.098	1.225	3.592	1.657	0.947	2.902	2.099	1.273	3.461	1.699	1.013	2.849	1.310	0.770	2.229
NCMP participation (non-participation >0.2)		1.071	0.917	1.250	1.067	0.900	1.266	1.096	0.899	1.338	0.991	0.819	1.198	1.370	1.045	1.797
**Factor**		**Odds Ratio**	**LCI**	**UCI**	**Odds Ratio**	**LCI**	**UCI**	**Odds Ratio**	**LCI**	**UCI**	**Odds Ratio**	**LCI**	**UCI**	**Odds Ratio**	**LCI**	**UCI**
Gender	Male	(ref)	(ref)	(ref)	(ref)	(ref)	(ref)	(ref)	(ref)	(ref)	(ref)	(ref)	(ref)	(ref)	(ref)	(ref)
	Female	1.319	1.181	1.473	1.331	1.200	1.478	1.301	1.181	1.432	1.350	1.225	1.489	1.130	1.021	1.250
Ethnicity	White - British	(ref)	(ref)	(ref)	(ref)	(ref)	(ref)	(ref)	(ref)	(ref)	(ref)	(ref)	(ref)	(ref)	(ref)	(ref)
	Any other White background	1.047	0.705	1.555	0.826	0.572	1.194	1.110	0.821	1.500	1.102	0.801	1.515	1.105	0.817	1.494
	Asian or Asian British	0.738	0.312	1.744	0.760	0.398	1.452	0.878	0.437	1.766	0.979	0.490	1.954	0.742	0.377	1.463
	Mixed/Dual background	1.409	0.886	2.241	0.725	0.445	1.181	1.079	0.738	1.578	0.914	0.625	1.336	0.958	0.634	1.448
	Any other ethnic group	1.348	0.670	2.711	0.989	0.457	2.142	0.963	0.522	1.777	1.092	0.637	1.871	0.517	0.247	1.080
SES	IMD 2010	2.098	1.222	3.601	1.596	0.912	2.794	2.143	1.296	3.542	1.769	1.052	2.976	1.308	0.766	2.231
NCMP participation (non-participation >0.2)		1.071	0.917	1.251	1.077	0.908	1.277	1.097	0.899	1.338	0.996	0.824	1.204	1.370	1.044	1.797
Factor 3		1.000	0.872	1.146	1.139	0.990	1.310	0.946	0.830	1.078	0.910	0.799	1.037	1.004	0.880	1.147
**Interaction**		**Odds Ratio**	**LCI**	**UCI**	**Odds Ratio**	**LCI**	**UCI**	**Odds Ratio**	**LCI**	**UCI**	**Odds Ratio**	**LCI**	**UCI**	**Odds Ratio**	**LCI**	**UCI**
Gender	Male	(ref)	(ref)	(ref)	(ref)	(ref)	(ref)	(ref)	(ref)	(ref)	(ref)	(ref)	(ref)	(ref)	(ref)	(ref)
	Female	1.320	1.182	1.474	1.335	1.203	1.481	1.301	1.182	1.433	1.352	1.226	1.490	1.132	1.023	1.252
Ethnicity	White - British	(ref)	(ref)	(ref)	(ref)	(ref)	(ref)	(ref)	(ref)	(ref)	(ref)	(ref)	(ref)	(ref)	(ref)	(ref)
	Any other White background	1.082	0.728	1.609	0.860	0.596	1.242	1.114	0.824	1.505	1.116	0.812	1.534	1.127	0.834	1.524
	Asian or Asian British	0.743	0.314	1.758	0.772	0.405	1.474	0.884	0.440	1.776	1.006	0.504	2.007	0.761	0.386	1.499
	Mixed/Dual background	1.403	0.882	2.231	0.733	0.450	1.193	1.093	0.747	1.598	0.934	0.639	1.365	0.977	0.647	1.476
	Any other ethnic group	1.378	0.684	2.775	1.001	0.463	2.165	0.963	0.522	1.776	1.084	0.633	1.855	0.515	0.247	1.076
SES	IMD 2010	2.171	1.264	3.729	1.634	0.934	2.858	2.151	1.303	3.553	1.779	1.057	2.995	1.329	0.779	2.268
NCMP participation (non-participation >0.2)		1.056	0.904	1.234	1.046	0.887	1.234	1.052	0.861	1.285	1.035	0.860	1.245	1.306	0.998	1.710
Factor 3		1.143	0.918	1.424	1.189	0.958	1.476	0.934	0.767	1.139	0.944	0.776	1.149	1.018	0.830	1.248
Year group	Reception	(ref)	(ref)	(ref)	(ref)	(ref)	(ref)	(ref)	(ref)	(ref)	(ref)	(ref)	(ref)	(ref)	(ref)	(ref)
	Year 6	1.987	1.117	3.534	1.577	0.888	2.802	1.134	0.656	1.961	1.466	0.885	2.428	1.272	0.743	2.180
Year group*factor	Reception	(ref)	(ref)	(ref)	(ref)	(ref)	(ref)	(ref)	(ref)	(ref)	(ref)	(ref)	(ref)	(ref)	(ref)	(ref)
	Year 6	0.798	0.603	1.056	0.926	0.705	1.215	1.023	0.788	1.329	0.951	0.748	1.208	0.974	0.753	1.258

Supporting file 3 to Williams, A.J., Wyatt, K.M., Williams, C.A., Logan, S., and Henley, W.E. Exploring the potential of a school impact on pupil weight status: exploratory factor analysis and repeat cross-sectional study of the National Child Measurement Programme.

S3 file - Detailed results and sensitivity analysis

Factor 3 - Resource

Red shading indicates significance (p<0.05), Green shading indicates modles which had to be estimated in R

Obese (IOTF)

**Single level**		**2006/07**			**2007/08**			**2008/09**			**2009/10**			**2010/11**		
n		9910			11353			11696			11851			11016		
Log likelihood	**Null**	-1605.40			-1724.55			-1999.60			-2090.78			-1901.75		
	**Individual**	-1595.21			-1703.72			-1989.84			-2072.60			-1869.13		
	**Factor**	-1593.71			-1703.70			-1988.89			-2070.63			-1869.01		
**Individual**		**Odds Ratio**	**LCI**	**UCI**	**Odds Ratio**	**LCI**	**UCI**	**Odds Ratio**	**LCI**	**UCI**	**Odds Ratio**	**LCI**	**UCI**	**Odds Ratio**	**LCI**	**UCI**
Gender	Male	(ref)	(ref)	(ref)	(ref)	(ref)	(ref)	(ref)	(ref)	(ref)	(ref)	(ref)	(ref)	(ref)	(ref)	(ref)
	Female	1.066	0.868	1.310	1.207	0.987	1.475	1.255	1.045	1.508	1.220	1.020	1.459	1.308	1.083	1.580
Ethnicity	White - British	(ref)	(ref)	(ref)	(ref)	(ref)	(ref)	(ref)	(ref)	(ref)	(ref)	(ref)	(ref)	(ref)	(ref)	(ref)
	Any other White background	0.977	0.455	2.094	1.994	1.236	3.219	1.097	0.624	1.929	1.293	0.761	2.195	0.720	0.368	1.411
	Asian or Asian British	1.522	0.470	4.930	1.761	0.759	4.086	0.796	0.193	3.275	1.410	0.507	3.918	2.012	0.911	4.442
	Mixed/Dual background	1.619	0.747	3.504	1.083	0.476	2.466	1.078	0.527	2.205	0.752	0.352	1.609	1.467	0.768	2.804
	Any other ethnic group	1.503	0.465	4.855	2.289	0.819	6.401	2.249	1.027	4.925	1.443	0.627	3.323	1.456	0.586	3.621
SES	IMD 2010	7.403	2.939	18.646	7.276	2.922	18.121	3.863	1.682	8.873	9.268	4.174	20.580	19.715	8.886	43.742
NCMP participation (non-participation >0.2)		0.877	0.644	1.195	0.601	0.421	0.858	0.930	0.651	1.328	0.823	0.587	1.154	0.678	0.369	1.245
**Factor**		**Odds Ratio**	**LCI**	**UCI**	**Odds Ratio**	**LCI**	**UCI**	**Odds Ratio**	**LCI**	**UCI**	**Odds Ratio**	**LCI**	**UCI**	**Odds Ratio**	**LCI**	**UCI**
Gender	Male	(ref)	(ref)	(ref)	(ref)	(ref)	(ref)	(ref)	(ref)	(ref)	(ref)	(ref)	(ref)	(ref)	(ref)	(ref)
	Female	1.064	0.866	1.308	1.207	0.987	1.475	1.256	1.045	1.510	1.220	1.020	1.459	1.308	1.083	1.579
Ethnicity	White - British	(ref)	(ref)	(ref)	(ref)	(ref)	(ref)	(ref)	(ref)	(ref)	(ref)	(ref)	(ref)	(ref)	(ref)	(ref)
	Any other White background	1.002	0.467	2.150	1.997	1.237	3.225	1.085	0.617	1.909	1.280	0.753	2.173	0.721	0.368	1.411
	Asian or Asian British	1.573	0.485	5.099	1.766	0.761	4.101	0.780	0.189	3.214	1.351	0.485	3.760	2.033	0.920	4.496
	Mixed/Dual background	1.637	0.756	3.546	1.084	0.476	2.468	1.067	0.521	2.182	0.742	0.347	1.587	1.469	0.769	2.808
	Any other ethnic group	1.549	0.479	5.008	2.296	0.821	6.421	2.229	1.018	4.882	1.408	0.612	3.244	1.462	0.588	3.635
SES	IMD 2010	8.198	3.220	20.868	7.355	2.934	18.442	3.587	1.554	8.280	8.148	3.641	18.236	20.280	9.065	45.371
NCMP participation (non-participation >0.2)		0.873	0.641	1.189	0.600	0.420	0.857	0.932	0.653	1.331	0.813	0.580	1.140	0.678	0.369	1.246
Factor 3		0.794	0.610	1.033	0.976	0.769	1.240	1.163	0.939	1.439	1.236	1.004	1.521	0.945	0.750	1.190

**Reception two-level**		**2006/07**			**2007/08**			**2008/09**			**2009/10**			**2010/11**		
n		4352			5190			5421			5373			4970		
Log likelihood	**Null**	-612.05			-756.86			-925.76			-854.75			-796.40		
	**Individual**	-605.01			-742.63			-914.05			-848.53			-786.91		
	**Factor**	-604.85			-742.61			-913.42			-847.54			-786.83		
School variance	**Null**	7.88E-12	0	.	0.205	0.056	0.750	0.117	0.024	0.577	0.458	0.255	0.823	0.110	0.010	1.184
	**Individual**	3.72E-16	0	.	0.151			0.126			0.384	0.202	0.732	0.117	0.015	0.929
	**Factor**	1.26E-15	0	.	0.151			0.126			0.365	0.189	0.704	0.114	0.014	0.958
**Individual**		**Odds Ratio**	**LCI**	**UCI**	**Odds Ratio**	**LCI**	**UCI**	**Odds Ratio**	**LCI**	**UCI**	**Odds Ratio**	**LCI**	**UCI**	**Odds Ratio**	**LCI**	**UCI**
Gender	Male	(ref)	(ref)	(ref)	(ref)	(ref)	(ref)	(ref)	(ref)	(ref)	(ref)	(ref)	(ref)	(ref)	(ref)	(ref)
	Female	1.443	1.023	2.034	1.369	1.000	1.876	1.675	1.264	2.221	1.139	0.857	1.515	1.236	0.920	1.660
Ethnicity	White - British	(ref)	(ref)	(ref)	(ref)	(ref)	(ref)	(ref)	(ref)	(ref)	(ref)	(ref)	(ref)	(ref)	(ref)	(ref)
	Any other White background	1.375	0.551	3.428	2.273	1.224	4.222	1.339	0.634	2.826	1.149	0.513	2.574	0.951	0.412	2.197
	Asian or Asian British	3.585	1.053	12.208	1.743	0.511	5.943	3.52E-06	0	.	3.548	1.191	10.567	2.297	0.795	6.635
	Mixed/Dual background	1.999	0.613	6.520	7.40E-07	0.000	.	0.683	0.209	2.232	0.647	0.201	2.085	0.820	0.255	2.631
	Any other ethnic group	1.063	0.143	7.903	6.68E-07	0.000	.	1.423	0.327	6.199	1.260	0.291	5.457	2.223	0.668	7.398
SES	IMD 2010	5.261	1.155	23.970	6.738	1.563	29.048	4.295	1.217	15.162	6.086	1.539	24.074	12.443	3.260	47.488
NCMP participation (non-participation >0.2)		0.861	0.499	1.484	0.558	0.289	1.077	0.726	0.304	1.736	0.782	0.437	1.398	0.822	0.192	3.519
**Factor**		**Odds Ratio**	**LCI**	**UCI**	**Odds Ratio**	**LCI**	**UCI**	**Odds Ratio**	**LCI**	**UCI**	**Odds Ratio**	**LCI**	**UCI**	**Odds Ratio**	**LCI**	**UCI**
Gender	Male	(ref)	(ref)	(ref)	(ref)	(ref)	(ref)	(ref)	(ref)	(ref)	(ref)	(ref)	(ref)	(ref)	(ref)	(ref)
	Female	1.446	1.026	2.039	1.369	1.000	1.876	1.675	1.264	2.221	1.139	0.857	1.515	1.237	0.920	1.661
Ethnicity	White - British	(ref)	(ref)	(ref)	(ref)	(ref)	(ref)	(ref)	(ref)	(ref)	(ref)	(ref)	(ref)	(ref)	(ref)	(ref)
	Any other White background	1.350	0.540	3.374	2.271	1.223	4.219	1.327	0.628	2.801	1.148	0.513	2.572	0.950	0.411	2.194
	Asian or Asian British	3.521	1.032	12.004	1.738	0.510	5.928	3.39E-06	0	.	3.459	1.162	10.295	2.276	0.788	6.580
	Mixed/Dual background	1.979	0.607	6.459	7.40E-07	0	.	0.677	0.207	2.214	0.639	0.198	2.059	0.818	0.255	2.627
	Any other ethnic group	1.041	0.140	7.752	6.67E-07	0	.	1.419	0.326	6.178	1.230	0.284	5.319	2.210	0.664	7.356
SES	IMD 2010	4.976	1.082	22.878	6.611	1.511	28.924	3.895	1.093	13.880	5.336	1.348	21.128	11.931	3.083	46.169
NCMP participation (non-participation >0.2)		0.862	0.500	1.486	0.560	0.290	1.084	0.711	0.297	1.701	0.771	0.432	1.377	0.811	0.189	3.476
Factor 3		1.132	0.732	1.750	1.038	0.683	1.579	1.223	0.856	1.746	1.342	0.894	2.014	1.081	0.736	1.590

**Year 6 two-level**		**2006/07**			**2007/08**			**2008/09**			**2009/10**			**2010/11**		
n		5558			6163			6275			6478			6046		
Log likelihood	**Null**	-987.29			-963.28			-1068.95			-1206.63			-1102.13		
	**Individual**	-979.22			-950.71			-1064.68			-1197.20			-1076.18		
	**Factor**	-976.21			-950.70			-1064.28			-1196.86			-1075.55		
School variance	**Null**	0.123	0.032	0.474	0.213	0.072	0.627	0.253	0.101	0.633	0.151	0.046	0.494	0.119	0.027	0.535
	**Individual**	0.057	0.004	0.758	0.185	0.056	0.616	0.233	0.088	0.618	0.112			0.007	4.94E-11	8.76E+05
	**Factor**	0.016			0.184	0.054	0.621	0.233	0.088	0.617	0.104			0.010	3.14E-08	3015.437
**Individual**		**Odds Ratio**	**LCI**	**UCI**	**Odds Ratio**	**LCI**	**UCI**	**Odds Ratio**	**LCI**	**UCI**	**Odds Ratio**	**LCI**	**UCI**	**Odds Ratio**	**LCI**	**UCI**
Gender	Male	(ref)	(ref)	(ref)	(ref)	(ref)	(ref)	(ref)	(ref)	(ref)	(ref)	(ref)	(ref)	(ref)	(ref)	(ref)
	Female	0.891	0.686	1.156	1.089	0.832	1.426	0.996	0.774	1.283	1.675	1.264	2.221	1.367	1.068	1.750
Ethnicity	White - British	(ref)	(ref)	(ref)	(ref)	(ref)	(ref)	(ref)	(ref)	(ref)	(ref)	(ref)	(ref)	(ref)	(ref)	(ref)
	Any other White background	0.684	0.166	2.820	1.636	0.700	3.821	0.877	0.353	2.178	1.339	0.634	2.826	0.501	0.157	1.593
	Asian or Asian British	0.000	0	.	1.715	0.504	5.838	1.807	0.419	7.796	3.52E-06	0	.	1.790	0.531	6.031
	Mixed/Dual background	1.426	0.511	3.974	2.136	0.909	5.020	1.591	0.630	4.020	0.683	0.209	2.232	2.321	1.049	5.133
	Any other ethnic group	2.142	0.494	9.287	4.246	1.426	12.646	3.255	1.235	8.577	1.423	0.327	6.199	0.957	0.229	3.990
SES	IMD 2010	8.900	2.628	30.136	7.916	2.112	29.672	2.819	0.789	10.075	4.295	1.217	15.162	31.501	11.229	88.371
NCMP participation (non-participation >0.2)		0.853	0.579	1.257	0.585	0.364	0.940	0.970	0.620	1.516	0.726	0.304	1.736	0.597	0.303	1.176
**Factor**		**Odds Ratio**	**LCI**	**UCI**	**Odds Ratio**	**LCI**	**UCI**	**Odds Ratio**	**LCI**	**UCI**	**Odds Ratio**	**LCI**	**UCI**	**Odds Ratio**	**LCI**	**UCI**
Gender	Male	(ref)	(ref)	(ref)	(ref)	(ref)	(ref)	(ref)	(ref)	(ref)	(ref)	(ref)	(ref)	(ref)	(ref)	(ref)
	Female	0.893	0.688	1.160	1.089	0.832	1.426	0.997	0.775	1.283	1.324	1.043	1.680	1.366	1.067	1.749
Ethnicity	White - British	(ref)	(ref)	(ref)	(ref)	(ref)	(ref)	(ref)	(ref)	(ref)	(ref)	(ref)	(ref)	(ref)	(ref)	(ref)
	Any other White background	0.694	0.168	2.870	1.642	0.702	3.839	0.870	0.350	2.159	1.343	0.641	2.811	0.502	0.158	1.596
	Asian or Asian British	4.27E-06	0	.	1.720	0.505	5.859	1.798	0.417	7.757	3.10E-06	0	.	1.875	0.555	6.340
	Mixed/Dual background	1.435	0.514	4.002	2.138	0.910	5.023	1.576	0.623	3.982	0.902	0.322	2.526	2.334	1.055	5.164
	Any other ethnic group	2.211	0.511	9.572	4.258	1.429	12.690	3.223	1.223	8.494	1.621	0.570	4.615	0.964	0.231	4.022
SES	IMD 2010	11.090	3.345	36.772	7.984	2.121	30.053	2.660	0.741	9.542	5.971	1.835	19.433	34.499	12.109	98.287
NCMP participation (non-participation >0.2)		0.847	0.579	1.239	0.585	0.364	0.940	0.977	0.625	1.528	1.111	0.686	1.801	0.592	0.300	1.169
Factor 3		0.648	0.458	0.916	0.973	0.695	1.363	1.162	0.838	1.611	1.128	0.845	1.506	0.842	0.620	1.142

**Three-level**		**2006/07**			**2007/08**			**2008/09**			**2009/10**			**2010/11**		
n		9910			11353			11696			11851			11016		
Log likelihood	**Null**	-1602.53			-1719.64			-1992.75			-2063.11			-1898.27		
	**Individual**	-1594.02			-1700.41			-1984.67			-2053.82			-1868.08		
	**Factor**	-1592.85			-1700.41			-1983.55			-2052.89			-1867.91		
	**Interaction**	-1586.34			-1699.44			-1983.52			-2049.99			-1865.16		
School variance	**Null**	0.122	0.034	0.435	0.108	0.023	0.508	0.164	0.064	0.419	0.124			0.140	0.047	0.418
	**Individual**	0.074	0.010	0.551	0.098	0.020	0.484	0.135	0.045	0.411	0.081	0.013	0.499	0.057	0.006	0.533
	**Factor**	0.071	0.009	0.559	0.098	0.020	0.484	0.142	0.049	0.411	0.078	0.012	0.498	0.058	0.007	0.519
	**Interaction**	0.045	0.003	0.592	0.105	0.024	0.459	0.141	0.048	0.413	0.082	0.015	0.458	0.057	0.011	0.310
Year group varia	**Null**	0.007	1.29E-11	3.64E+06	0.097	0.010	0.914	0.035	4.31E-04	2.823	0.206			0.002	3.05E-43	1.03E+37
	**Individual**	0.012	1.75E-07	883.061	0.063	0.003	1.488	0.041	0.001	1.801	0.173	0.059	0.506	0.007	2.17E-13	2.01E+08
	**Factor**	0.001	9.37E-71	7.97E+63	0.063	0.003	1.501	0.033	3.23E-04	3.417	0.166	0.055	0.500	0.007	5.36E-13	8.73E+07
	**Interaction**	1.63E-11	0	.	0.048	0.001	2.739	0.035	4.13E-04	2.956	0.158	0.052	0.473	2.54E-10	0	.
**Individual**		**Odds Ratio**	**LCI**	**UCI**	**Odds Ratio**	**LCI**	**UCI**	**Odds Ratio**	**LCI**	**UCI**	**Odds Ratio**	**LCI**	**UCI**	**Odds Ratio**	**LCI**	**UCI**
Gender	Male	(ref)	(ref)	(ref)	(ref)	(ref)	(ref)	(ref)	(ref)	(ref)	(ref)	(ref)	(ref)	(ref)	(ref)	(ref)
	Female	1.064	0.866	1.308	1.200	0.981	1.469	1.262	1.049	1.518	1.234	1.030	1.479	1.310	1.084	1.583
Ethnicity	White - British	(ref)	(ref)	(ref)	(ref)	(ref)	(ref)	(ref)	(ref)	(ref)	(ref)	(ref)	(ref)	(ref)	(ref)	(ref)
	Any other White background	0.979	0.455	2.108	2.011	1.229	3.290	1.120	0.633	1.982	1.255	0.730	2.156	0.730	0.372	1.432
	Asian or Asian British	1.550	0.473	5.075	1.689	0.713	4.002	0.796	0.191	3.313	1.560	0.554	4.393	2.056	0.926	4.566
	Mixed/Dual background	1.654	0.760	3.597	1.113	0.486	2.546	1.093	0.532	2.249	0.779	0.362	1.677	1.453	0.758	2.784
	Any other ethnic group	1.537	0.470	5.023	2.280	0.801	6.488	2.341	1.054	5.198	1.518	0.650	3.547	1.483	0.593	3.705
SES	IMD 2010	6.827	2.568	18.149	7.068	2.639	18.934	3.241	1.292	8.127	5.636	2.250	14.115	20.503	8.866	47.418
NCMP participation (non-participation >0.2)		0.885	0.642	1.219	0.574	0.394	0.837	0.932	0.637	1.364	0.902	0.621	1.310	0.679	0.366	1.262
**Factor**		**Odds Ratio**	**LCI**	**UCI**	**Odds Ratio**	**LCI**	**UCI**	**Odds Ratio**	**LCI**	**UCI**	**Odds Ratio**	**LCI**	**UCI**	**Odds Ratio**	**LCI**	**UCI**
Gender	Male	(ref)	(ref)	(ref)	(ref)	(ref)	(ref)	(ref)	(ref)	(ref)	(ref)	(ref)	(ref)	(ref)	(ref)	(ref)
	Female	1.063	0.865	1.307	1.200	0.981	1.469	1.262	1.049	1.519	1.234	1.029	1.479	1.310	1.084	1.583
Ethnicity	White - British	(ref)	(ref)	(ref)	(ref)	(ref)	(ref)	(ref)	(ref)	(ref)	(ref)	(ref)	(ref)	(ref)	(ref)	(ref)
	Any other White background	1.001	0.465	2.155	2.012	1.229	3.292	1.110	0.627	1.966	1.248	0.726	2.144	0.730	0.372	1.434
	Asian or Asian British	1.581	0.483	5.175	1.689	0.713	4.005	0.782	0.188	3.257	1.522	0.540	4.288	2.081	0.936	4.627
	Mixed/Dual background	1.666	0.766	3.622	1.113	0.486	2.547	1.083	0.527	2.228	0.771	0.358	1.660	1.454	0.759	2.788
	Any other ethnic group	1.564	0.479	5.106	2.280	0.801	6.491	2.327	1.048	5.168	1.498	0.641	3.499	1.487	0.595	3.718
SES	IMD 2010	7.527	2.811	20.159	7.080	2.629	19.070	3.004	1.194	7.561	5.238	2.090	13.129	21.278	9.104	49.732
NCMP participation (non-participation >0.2)		0.881	0.640	1.212	0.574	0.394	0.837	0.935	0.639	1.368	0.893	0.615	1.296	0.681	0.366	1.265
Factor 3		0.803	0.606	1.064	0.996	0.764	1.298	1.209	0.943	1.549	1.190	0.928	1.524	0.930	0.727	1.189
**Interaction**		**Odds Ratio**	**LCI**	**UCI**	**Odds Ratio**	**LCI**	**UCI**	**Odds Ratio**	**LCI**	**UCI**	**Odds Ratio**	**LCI**	**UCI**	**Odds Ratio**	**LCI**	**UCI**
Gender	Male	(ref)	(ref)	(ref)	(ref)	(ref)	(ref)	(ref)	(ref)	(ref)	(ref)	(ref)	(ref)	(ref)	(ref)	(ref)
	Female	1.066	0.867	1.311	1.203	0.983	1.472	1.263	1.049	1.519	1.235	1.031	1.480	1.313	1.087	1.587
Ethnicity	White - British	(ref)	(ref)	(ref)	(ref)	(ref)	(ref)	(ref)	(ref)	(ref)	(ref)	(ref)	(ref)	(ref)	(ref)	(ref)
	Any other White background	1.049	0.487	2.259	2.068	1.262	3.386	1.111	0.628	1.967	1.266	0.737	2.176	0.749	0.381	1.470
	Asian or Asian British	1.614	0.495	5.260	1.706	0.720	4.044	0.782	0.188	3.255	1.554	0.551	4.381	2.136	0.960	4.752
	Mixed/Dual background	1.640	0.754	3.563	1.117	0.488	2.557	1.085	0.527	2.232	0.786	0.365	1.694	1.494	0.779	2.866
	Any other ethnic group	1.587	0.487	5.170	2.276	0.800	6.476	2.328	1.048	5.168	1.493	0.640	3.487	1.482	0.593	3.705
SES	IMD 2010	8.031	3.038	21.236	7.112	2.639	19.169	3.003	1.193	7.559	5.242	2.090	13.152	21.646	9.288	50.446
NCMP participation (non-participation >0.2)		0.861	0.627	1.181	0.565	0.388	0.824	0.929	0.631	1.366	0.918	0.633	1.333	0.633	0.340	1.179
Factor 3		1.105	0.713	1.714	1.042	0.693	1.566	1.228	0.862	1.751	1.348	0.917	1.980	1.042	0.716	1.516
Year group	Reception	(ref)	(ref)	(ref)	(ref)	(ref)	(ref)	(ref)	(ref)	(ref)	(ref)	(ref)	(ref)	(ref)	(ref)	(ref)
	Year 6	3.958	1.279	12.254	1.356	0.463	3.972	1.081	0.414	2.822	1.872	0.664	5.281	1.875	0.690	5.095
Year group*factor	Reception	(ref)	(ref)	(ref)	(ref)	(ref)	(ref)	(ref)	(ref)	(ref)	(ref)	(ref)	(ref)	(ref)	(ref)	(ref)
	Year 6	0.595	0.343	1.034	0.927	0.557	1.543	0.972	0.619	1.525	0.832	0.515	1.346	0.819	0.510	1.315

Supporting file 3 to Williams, A.J., Wyatt, K.M., Williams, C.A., Logan, S., and Henley, W.E. Exploring the potential of a school impact on pupil weight status: exploratory factor analysis and repeat cross-sectional study of the National Child Measurement Programme.

S3 file - Detailed results and sensitivity analysis

Factor 3 - Resource

Red shading indicates significance (p<0.05), Green shading indicates modles which had to be estimated in R

BMI-SDS

Single level		2006/07			2007/08			2008/09			2009/10			2010/11		
n		9910			11353			11696			11851			11016		
Log likelihood	**Null**	-14914.99			-16983.74			-17154.20			-17558.83			-16084.62		
	**Individual**	-14907.46			-16971.41			-17128.16			-17540.88			-16059.60		
	**Factor**	-14910.00			-16973.04			-17131.01			-17542.67			-16061.19		
Residual varianc	**Null**	1.187	1.155	1.221	1.166	1.136	1.197	1.100	1.072	1.128	1.133	1.104	1.162	1.085	1.057	1.114
	**Individual**	1.183	1.151	1.217	1.162	1.132	1.192	1.093	1.065	1.121	1.128	1.099	1.157	1.079	1.050	1.107
	**Factor**	1.184	1.151	1.217	1.161	1.132	1.192	1.093	1.065	1.121	1.128	1.099	1.157	1.078	1.050	1.107
**Null**		**Mean difference**	**LCI**	**UCI**	**Mean difference**	**LCI**	**UCI**	**Mean difference**	**LCI**	**UCI**	**Mean difference**	**LCI**	**UCI**	**Mean difference**	**LCI**	**UCI**
Constant		0.340	0.319	0.362	0.327	0.307	0.347	0.463	0.444	0.482	0.434	0.415	0.453	0.457	0.438	0.477
**Individual**		**Mean difference**	**LCI**	**UCI**	**Mean difference**	**LCI**	**UCI**	**Mean difference**	**LCI**	**UCI**	**Mean difference**	**LCI**	**UCI**	**Mean difference**	**LCI**	**UCI**
Constant		0.289	0.236	0.342	0.322	0.273	0.370	0.434	0.388	0.480	0.395	0.348	0.441	0.440	0.393	0.486
Gender	Male	(ref)	(ref)	(ref)	(ref)	(ref)	(ref)	(ref)	(ref)	(ref)	(ref)	(ref)	(ref)	(ref)	(ref)	(ref)
	Female	-0.085	-0.127	-0.042	-0.123	-0.162	-0.083	-0.136	-0.173	-0.098	-0.112	-0.150	-0.073	-0.143	-0.182	-0.104
Ethnicity	White - British	(ref)	(ref)	(ref)	(ref)	(ref)	(ref)	(ref)	(ref)	(ref)	(ref)	(ref)	(ref)	(ref)	(ref)	(ref)
	Any other White background	0.040	-0.115	0.194	-0.035	-0.163	0.092	0.083	-0.038	0.205	0.009	-0.118	0.135	0.031	-0.089	0.150
	Asian or Asian British	-0.164	-0.466	0.139	-0.183	-0.409	0.042	-0.205	-0.475	0.064	-0.096	-0.361	0.169	-0.035	-0.268	0.198
	Mixed/Dual background	0.204	0.005	0.402	-0.032	-0.198	0.135	0.021	-0.132	0.174	-0.135	-0.279	0.009	-0.058	-0.213	0.097
	Any other ethnic group	0.034	-0.262	0.331	0.007	-0.287	0.300	0.037	-0.194	0.268	0.156	-0.058	0.371	-0.145	-0.366	0.077
SES	IMD 2010	0.462	0.248	0.676	0.343	0.141	0.545	0.464	0.278	0.650	0.476	0.283	0.668	0.440	0.247	0.633
NCMP participation (non-participation >0.2)		-0.007	-0.068	0.054	-0.007	-0.065	0.051	0.027	-0.044	0.098	0.005	-0.063	0.073	0.038	-0.068	0.144
**Factor**		**Mean difference**	**LCI**	**UCI**	**Mean difference**	**LCI**	**UCI**	**Mean difference**	**LCI**	**UCI**	**Mean difference**	**LCI**	**UCI**	**Mean difference**	**LCI**	**UCI**
Constant		0.317	0.201	0.433	0.248	0.143	0.353	0.440	0.341	0.539	0.329	0.229	0.429	0.366	0.262	0.469
Gender	Male	(ref)	(ref)	(ref)	(ref)	(ref)	(ref)	(ref)	(ref)	(ref)	(ref)	(ref)	(ref)	(ref)	(ref)	(ref)
	Female	-0.085	-0.127	-0.042	-0.122	-0.162	-0.083	-0.136	-0.173	-0.098	-0.112	-0.150	-0.073	-0.143	-0.182	-0.104
Ethnicity	White - British	(ref)	(ref)	(ref)	(ref)	(ref)	(ref)	(ref)	(ref)	(ref)	(ref)	(ref)	(ref)	(ref)	(ref)	(ref)
	Any other White background	0.041	-0.114	0.196	-0.038	-0.165	0.090	0.084	-0.038	0.205	0.007	-0.120	0.133	0.029	-0.090	0.149
	Asian or Asian British	-0.162	-0.464	0.141	-0.189	-0.414	0.037	-0.205	-0.475	0.065	-0.102	-0.368	0.163	-0.043	-0.276	0.190
	Mixed/Dual background	0.204	0.006	0.403	-0.033	-0.199	0.134	0.021	-0.132	0.174	-0.137	-0.281	0.007	-0.059	-0.213	0.096
	Any other ethnic group	0.036	-0.260	0.333	0.001	-0.292	0.295	0.037	-0.194	0.269	0.152	-0.062	0.367	-0.147	-0.369	0.075
SES	IMD 2010	0.467	0.252	0.682	0.328	0.126	0.531	0.465	0.278	0.653	0.458	0.264	0.652	0.422	0.228	0.617
NCMP participation (non-participation >0.2)		-0.007	-0.069	0.054	-0.004	-0.062	0.054	0.027	-0.044	0.098	0.003	-0.065	0.071	0.038	-0.068	0.144
Factor 3		-0.015	-0.068	0.039	0.037	-0.010	0.084	-0.003	-0.048	0.042	0.034	-0.012	0.079	0.038	-0.010	0.085

Reception two-level		2006/07			2007/08			2008/09			2009/10			2010/11		
n		4352			5190			5421			5373			4970		
Log likelihood	**Null**	-6239.98			-7379.80			-7429.67			-7484.45			-6734.36		
	**Individual**	-6245.04			-7371.92			-7428.49			-7486.78			-6735.96		
	**Factor**	-6246.71			-7373.35			-7430.63			-7488.19			-6736.95		
School variance	**Null**	0.041	0.026	0.065	0.042	0.028	0.063	0.025	0.016	0.039	0.040	0.028	0.059	0.014	0.006	0.030
	**Individual**	0.040	0.025	0.064	0.040	0.026	0.062	0.024	0.015	0.038	0.038	0.026	0.057	0.014	0.006	0.029
	**Factor**	0.040	0.025	0.064	0.040	0.026	0.061	0.024	0.016	0.038	0.038	0.026	0.056	0.013	0.006	0.029
Residual varianc	**Null**	1.000	0.958	1.045	0.977	0.939	1.016	0.888	0.855	0.923	0.922	0.887	0.958	0.867	0.833	0.903
	**Individual**	1.001	0.958	1.045	0.972	0.934	1.011	0.886	0.853	0.921	0.921	0.886	0.957	0.866	0.832	0.901
	**Factor**	1.001	0.958	1.045	0.972	0.934	1.011	0.886	0.853	0.921	0.921	0.886	0.957	0.866	0.832	0.902
**Null**		**Mean difference**	**LCI**	**UCI**	**Mean difference**	**LCI**	**UCI**	**Mean difference**	**LCI**	**UCI**	**Mean difference**	**LCI**	**UCI**	**Mean difference**	**LCI**	**UCI**
Constant		0.292	0.251	0.334	0.293	0.255	0.332	0.466	0.433	0.500	0.411	0.373	0.448	0.460	0.429	0.491
**Individual**		**Mean difference**	**LCI**	**UCI**	**Mean difference**	**LCI**	**UCI**	**Mean difference**	**LCI**	**UCI**	**Mean difference**	**LCI**	**UCI**	**Mean difference**	**LCI**	**UCI**
Constant		0.297	0.212	0.381	0.261	0.185	0.338	0.408	0.340	0.477	0.404	0.332	0.477	0.433	0.365	0.501
Gender	Male	(ref)	(ref)	(ref)	(ref)	(ref)	(ref)	(ref)	(ref)	(ref)	(ref)	(ref)	(ref)	(ref)	(ref)	(ref)
	Female	-0.041	-0.101	0.019	-0.087	-0.141	-0.032	-0.054	-0.104	-0.003	-0.063	-0.115	-0.011	-0.071	-0.124	-0.019
Ethnicity	White - British	(ref)	(ref)	(ref)	(ref)	(ref)	(ref)	(ref)	(ref)	(ref)	(ref)	(ref)	(ref)	(ref)	(ref)	(ref)
	Any other White background	-0.049	-0.234	0.137	-0.078	-0.232	0.077	-0.008	-0.166	0.150	-0.024	-0.187	0.140	0.057	-0.086	0.200
	Asian or Asian British	-0.060	-0.454	0.334	-0.550	-0.839	-0.261	-0.511	-0.872	-0.151	-0.166	-0.489	0.157	-0.025	-0.304	0.255
	Mixed/Dual background	0.157	-0.125	0.438	-0.130	-0.350	0.090	-0.029	-0.214	0.157	-0.227	-0.404	-0.050	-0.151	-0.339	0.036
	Any other ethnic group	-0.062	-0.437	0.312	-0.255	-0.674	0.164	-0.003	-0.313	0.306	-0.183	-0.483	0.118	-0.048	-0.344	0.248
SES	IMD 2010	0.174	-0.156	0.504	0.480	0.173	0.788	0.430	0.156	0.704	0.232	-0.057	0.520	0.313	0.037	0.589
NCMP participation (non-participation >0.2)		-0.113	-0.227	0.000	-0.071	-0.185	0.043	0.083	-0.074	0.241	-0.008	-0.121	0.104	0.140	-0.107	0.386
**Factor**		**Mean difference**	**LCI**	**UCI**	**Mean difference**	**LCI**	**UCI**	**Mean difference**	**LCI**	**UCI**	**Mean difference**	**LCI**	**UCI**	**Mean difference**	**LCI**	**UCI**
Constant		0.208	-0.002	0.419	0.150	-0.042	0.343	0.362	0.197	0.526	0.293	0.108	0.478	0.310	0.150	0.469
Gender	Male	(ref)	(ref)	(ref)	(ref)	(ref)	(ref)	(ref)	(ref)	(ref)	(ref)	(ref)	(ref)	(ref)	(ref)	(ref)
	Female	-0.041	-0.101	0.019	-0.087	-0.141	-0.032	-0.054	-0.104	-0.003	-0.063	-0.115	-0.011	-0.071	-0.123	-0.019
Ethnicity	White - British	(ref)	(ref)	(ref)	(ref)	(ref)	(ref)	(ref)	(ref)	(ref)	(ref)	(ref)	(ref)	(ref)	(ref)	(ref)
	Any other White background	-0.054	-0.239	0.132	-0.079	-0.234	0.076	-0.009	-0.167	0.149	-0.024	-0.187	0.139	0.055	-0.088	0.198
	Asian or Asian British	-0.064	-0.457	0.330	-0.553	-0.843	-0.264	-0.514	-0.875	-0.154	-0.169	-0.492	0.154	-0.031	-0.310	0.248
	Mixed/Dual background	0.155	-0.127	0.436	-0.130	-0.350	0.090	-0.030	-0.215	0.156	-0.229	-0.406	-0.052	-0.152	-0.339	0.036
	Any other ethnic group	-0.065	-0.440	0.309	-0.256	-0.675	0.163	-0.004	-0.313	0.306	-0.187	-0.487	0.114	-0.052	-0.348	0.244
SES	IMD 2010	0.163	-0.168	0.493	0.465	0.157	0.773	0.422	0.147	0.697	0.214	-0.075	0.504	0.288	0.012	0.565
NCMP participation (non-participation >0.2)		-0.112	-0.225	0.001	-0.063	-0.178	0.051	0.080	-0.078	0.238	-0.010	-0.123	0.102	0.130	-0.116	0.376
Factor 3		0.045	-0.053	0.143	0.055	-0.033	0.143	0.024	-0.052	0.099	0.055	-0.029	0.140	0.062	-0.011	0.136

Year 6 two-level		2006/07			2007/08			2008/09			2009/10			2010/11		
n		5558			6163			6275			6478			6046		
Log likelihood	**Null**	-8607.29			-9526.30			-9610.21			-9959.39			-9263.90		
	**Individual**	-8598.02			-9519.79			-9588.28			-9944.15			-9240.02		
	**Factor**	-8599.08			-9521.92			-9590.11			-9946.19			-9242.41		
School variance	**Null**	0.021	0.011	0.041	0.018	0.009	0.036	0.031	0.019	0.051	0.023	0.013	0.041	0.003	2.01E-04	0.045
	**Individual**	0.019	0.009	0.039	0.018	0.009	0.036	0.029	0.018	0.048	0.022	0.012	0.040	9.06E-04	1.73E-07	4.743
	**Factor**	0.018	0.009	0.038	0.019	0.009	0.036	0.029	0.018	0.048	0.023	0.013	0.041	1.03E-03	5.03E-07	2.087
Residual varianc	**Null**	1.278	1.230	1.327	1.272	1.227	1.319	1.228	1.185	1.273	1.248	1.205	1.293	1.250	1.206	1.296
	**Individual**	1.273	1.225	1.322	1.267	1.222	1.314	1.219	1.176	1.263	1.240	1.198	1.285	1.240	1.196	1.286
	**Factor**	1.273	1.225	1.322	1.267	1.222	1.314	1.218	1.176	1.263	1.240	1.197	1.285	1.240	1.196	1.286
**Null**		**Mean difference**	**LCI**	**UCI**	**Mean difference**	**LCI**	**UCI**	**Mean difference**	**LCI**	**UCI**	**Mean difference**	**LCI**	**UCI**	**Mean difference**	**LCI**	**UCI**
Constant		0.385	0.348	0.422	0.349	0.315	0.383	0.456	0.420	0.493	0.469	0.435	0.503	0.457	0.428	0.487
**Individual**		**Mean difference**	**LCI**	**UCI**	**Mean difference**	**LCI**	**UCI**	**Mean difference**	**LCI**	**UCI**	**Mean difference**	**LCI**	**UCI**	**Mean difference**	**LCI**	**UCI**
Constant		0.320	0.240	0.400	0.379	0.303	0.456	0.460	0.385	0.536	0.423	0.348	0.497	0.442	0.374	0.510
Gender	Male	(ref)	(ref)	(ref)	(ref)	(ref)	(ref)	(ref)	(ref)	(ref)	(ref)	(ref)	(ref)	(ref)	(ref)	(ref)
	Female	-0.123	-0.183	-0.064	-0.155	-0.212	-0.098	-0.202	-0.257	-0.147	-0.145	-0.199	-0.090	-0.202	-0.259	-0.146
Ethnicity	White - British	(ref)	(ref)	(ref)	(ref)	(ref)	(ref)	(ref)	(ref)	(ref)	(ref)	(ref)	(ref)	(ref)	(ref)	(ref)
	Any other White background	0.184	-0.080	0.448	0.067	-0.154	0.287	0.168	-0.015	0.352	0.060	-0.131	0.250	-0.013	-0.210	0.184
	Asian or Asian British	-0.295	-0.751	0.160	0.166	-0.179	0.511	0.011	-0.382	0.404	0.031	-0.393	0.455	-0.100	-0.482	0.281
	Mixed/Dual background	0.236	-0.037	0.509	0.068	-0.176	0.312	0.053	-0.192	0.299	0.020	-0.208	0.247	0.069	-0.180	0.318
	Any other ethnic group	0.154	-0.312	0.620	0.175	-0.231	0.581	0.056	-0.282	0.394	0.463	0.162	0.763	-0.221	-0.544	0.102
SES	IMD 2010	0.595	0.268	0.922	0.192	-0.125	0.510	0.451	0.148	0.754	0.523	0.214	0.831	0.574	0.291	0.857
NCMP participation (non-participation >0.2)		0.034	-0.062	0.130	0.010	-0.079	0.100	0.005	-0.102	0.111	0.104	-0.021	0.228	0.018	-0.109	0.144
**Factor**		**Mean difference**	**LCI**	**UCI**	**Mean difference**	**LCI**	**UCI**	**Mean difference**	**LCI**	**UCI**	**Mean difference**	**LCI**	**UCI**	**Mean difference**	**LCI**	**UCI**
Constant		0.451	0.264	0.637	0.329	0.160	0.499	0.538	0.361	0.716	0.364	0.197	0.532	0.417	0.269	0.565
Gender	Male	(ref)	(ref)	(ref)	(ref)	(ref)	(ref)	(ref)	(ref)	(ref)	(ref)	(ref)	(ref)	(ref)	(ref)	(ref)
	Female	-0.123	-0.183	-0.063	-0.155	-0.212	-0.098	-0.202	-0.257	-0.147	-0.145	-0.199	-0.090	-0.202	-0.259	-0.146
Ethnicity	White - British	(ref)	(ref)	(ref)	(ref)	(ref)	(ref)	(ref)	(ref)	(ref)	(ref)	(ref)	(ref)	(ref)	(ref)	(ref)
	Any other White background	0.187	-0.077	0.451	0.064	-0.157	0.285	0.170	-0.014	0.354	0.058	-0.132	0.249	-0.013	-0.210	0.184
	Asian or Asian British	-0.292	-0.748	0.164	0.163	-0.181	0.508	0.013	-0.380	0.406	0.027	-0.398	0.451	-0.104	-0.486	0.278
	Mixed/Dual background	0.236	-0.037	0.509	0.067	-0.177	0.311	0.055	-0.190	0.301	0.018	-0.209	0.246	0.068	-0.181	0.317
	Any other ethnic group	0.157	-0.308	0.623	0.172	-0.235	0.578	0.058	-0.280	0.396	0.461	0.160	0.762	-0.222	-0.545	0.102
SES	IMD 2010	0.613	0.286	0.941	0.186	-0.132	0.505	0.461	0.157	0.765	0.512	0.202	0.822	0.567	0.282	0.853
NCMP participation (non-participation >0.2)		0.032	-0.063	0.128	0.011	-0.079	0.100	0.003	-0.104	0.110	0.102	-0.022	0.227	0.018	-0.108	0.144
Factor 3		-0.066	-0.152	0.019	0.025	-0.050	0.100	-0.039	-0.119	0.041	0.030	-0.046	0.106	0.013	-0.054	0.080

Three-level		2006/07			2007/08			2008/09			2009/10			2010/11		
n		9910			11353			11696			11851			11016		
Log likelihood	**Null**	-14878.53			-16951.41			-17108.34			-17502.07			-16081.32		
	**Individual**	-14874.00			-16939.27			-17086.60			-17489.98			-16057.03		
	**Factor**	-14876.37			-16940.82			-17089.14			-17491.91			-16058.88		
	**Interaction**	-14871.46			-16943.08			-17093.22			-17492.75			-16063.42		
School variance	**Null**	0.021	0.012	0.038	0.008	0.002	0.029	0.013	0.006	0.027	0.019	0.010	0.034	0.003	3.15E-04	0.028
	**Individual**	0.020	0.011	0.037	0.007	0.002	0.030	0.011	0.005	0.026	0.017	0.009	0.032	0.002	6.69E-05	0.054
	**Factor**	0.020	0.011	0.037	0.007	0.002	0.030	0.011	0.005	0.026	0.017	0.009	0.032	0.002	2.92E-05	0.085
	**Interaction**	0.023	0.013	0.038	0.008	0.002	0.029	0.011	0.005	0.026	0.018	0.010	0.032	1.38E-03	1.38E-05	0.139
Year group variai	**Null**	0.009	0.003	0.034	0.021	0.011	0.038	0.016	0.008	0.030	0.014	0.007	0.030	0.004	5.01E-04	0.031
	**Individual**	0.009	0.002	0.034	0.021	0.012	0.039	0.016	0.008	0.031	0.014	0.007	0.030	0.004	5.71E-04	0.030
	**Factor**	0.009	0.003	0.034	0.021	0.012	0.039	0.016	0.008	0.031	0.014	0.007	0.030	0.004	5.87E-04	0.030
	**Interaction**	0.004	1.75E-04	0.076	0.020	0.011	0.038	0.016	0.008	0.031	0.013	0.006	0.028	0.004	6.84E-04	0.029
Residual varianc	**Null**	1.157	1.125	1.191	1.138	1.108	1.169	1.071	1.043	1.099	1.100	1.072	1.129	1.078	1.050	1.108
	**Individual**	1.155	1.122	1.188	1.134	1.104	1.164	1.066	1.038	1.094	1.097	1.068	1.125	1.073	1.044	1.102
	**Factor**	1.155	1.122	1.188	1.134	1.104	1.164	1.066	1.038	1.094	1.096	1.068	1.125	1.073	1.044	1.102
	**Interaction**	1.156	1.123	1.189	1.134	1.104	1.164	1.066	1.038	1.094	1.096	1.068	1.125	1.073	1.044	1.102
**Null**		**Mean difference**	**LCI**	**UCI**	**Mean difference**	**LCI**	**UCI**	**Mean difference**	**LCI**	**UCI**	**Mean difference**	**LCI**	**UCI**	**Mean difference**	**LCI**	**UCI**
Constant		0.345	0.314	0.376	0.322	0.295	0.349	0.461	0.434	0.488	0.444	0.415	0.472	0.459	0.437	0.480
**Individual**		**Mean difference**	**LCI**	**UCI**	**Mean difference**	**LCI**	**UCI**	**Mean difference**	**LCI**	**UCI**	**Mean difference**	**LCI**	**UCI**	**Mean difference**	**LCI**	**UCI**
Constant		0.318	0.257	0.379	0.324	0.268	0.379	0.439	0.387	0.492	0.421	0.366	0.475	0.442	0.393	0.491
Gender	Male	(ref)	(ref)	(ref)	(ref)	(ref)	(ref)	(ref)	(ref)	(ref)	(ref)	(ref)	(ref)	(ref)	(ref)	(ref)
	Female	-0.089	-0.132	-0.047	-0.124	-0.163	-0.084	-0.133	-0.171	-0.095	-0.109	-0.148	-0.071	-0.144	-0.183	-0.105
Ethnicity	White - British	(ref)	(ref)	(ref)	(ref)	(ref)	(ref)	(ref)	(ref)	(ref)	(ref)	(ref)	(ref)	(ref)	(ref)	(ref)
	Any other White background	0.028	-0.127	0.183	-0.030	-0.160	0.099	0.081	-0.041	0.203	0.027	-0.100	0.153	0.029	-0.091	0.149
	Asian or Asian British	-0.178	-0.481	0.125	-0.215	-0.440	0.011	-0.222	-0.491	0.047	-0.079	-0.343	0.186	-0.041	-0.274	0.192
	Mixed/Dual background	0.199	0.001	0.396	-0.032	-0.198	0.133	0.011	-0.141	0.163	-0.114	-0.257	0.029	-0.057	-0.212	0.098
	Any other ethnic group	0.030	-0.268	0.328	-0.017	-0.310	0.276	0.023	-0.208	0.255	0.197	-0.018	0.411	-0.141	-0.363	0.081
SES	IMD 2010	0.371	0.130	0.612	0.324	0.098	0.549	0.432	0.222	0.643	0.375	0.156	0.594	0.436	0.234	0.638
NCMP participation (non-participation >0.2)		-0.023	-0.096	0.050	-0.017	-0.087	0.054	0.011	-0.073	0.096	0.027	-0.056	0.109	0.042	-0.068	0.153
**Factor**		**Mean difference**	**LCI**	**UCI**	**Mean difference**	**LCI**	**UCI**	**Mean difference**	**LCI**	**UCI**	**Mean difference**	**LCI**	**UCI**	**Mean difference**	**LCI**	**UCI**
Constant		0.340	0.188	0.491	0.237	0.105	0.369	0.460	0.332	0.588	0.351	0.217	0.485	0.374	0.264	0.484
Gender	Male	(ref)	(ref)	(ref)	(ref)	(ref)	(ref)	(ref)	(ref)	(ref)	(ref)	(ref)	(ref)	(ref)	(ref)	(ref)
	Female	-0.089	-0.132	-0.047	-0.124	-0.163	-0.084	-0.133	-0.171	-0.095	-0.109	-0.147	-0.071	-0.144	-0.182	-0.105
Ethnicity	White - British	(ref)	(ref)	(ref)	(ref)	(ref)	(ref)	(ref)	(ref)	(ref)	(ref)	(ref)	(ref)	(ref)	(ref)	(ref)
	Any other White background	0.029	-0.127	0.184	-0.033	-0.162	0.097	0.082	-0.040	0.204	0.026	-0.101	0.152	0.028	-0.091	0.148
	Asian or Asian British	-0.178	-0.481	0.125	-0.217	-0.443	0.008	-0.221	-0.490	0.048	-0.082	-0.346	0.183	-0.047	-0.280	0.187
	Mixed/Dual background	0.199	0.002	0.396	-0.033	-0.199	0.133	0.012	-0.140	0.164	-0.115	-0.258	0.028	-0.058	-0.212	0.097
	Any other ethnic group	0.030	-0.268	0.328	-0.019	-0.312	0.274	0.024	-0.208	0.255	0.195	-0.020	0.409	-0.143	-0.365	0.079
SES	IMD 2010	0.373	0.132	0.615	0.314	0.088	0.540	0.435	0.224	0.646	0.366	0.146	0.585	0.421	0.219	0.624
NCMP participation (non-participation >0.2)		-0.023	-0.097	0.050	-0.014	-0.085	0.056	0.011	-0.074	0.096	0.025	-0.057	0.107	0.041	-0.069	0.151
Factor 3		-0.011	-0.081	0.059	0.043	-0.016	0.103	-0.010	-0.068	0.048	0.035	-0.027	0.096	0.034	-0.016	0.085
**Interaction**		**Mean difference**	**LCI**	**UCI**	**Mean difference**	**LCI**	**UCI**	**Mean difference**	**LCI**	**UCI**	**Mean difference**	**LCI**	**UCI**	**Mean difference**	**LCI**	**UCI**
Constant		0.162	-0.037	0.361	0.177	-0.009	0.362	0.405	0.231	0.579	0.301	0.117	0.485	0.320	0.158	0.482
Gender	Male	(ref)	(ref)	(ref)	(ref)	(ref)	(ref)	(ref)	(ref)	(ref)	(ref)	(ref)	(ref)	(ref)	(ref)	(ref)
	Female	-0.089	-0.132	-0.047	-0.123	-0.163	-0.084	-0.133	-0.171	-0.095	-0.109	-0.147	-0.071	-0.143	-0.182	-0.105
Ethnicity	White - British	(ref)	(ref)	(ref)	(ref)	(ref)	(ref)	(ref)	(ref)	(ref)	(ref)	(ref)	(ref)	(ref)	(ref)	(ref)
	Any other White background	0.041	-0.114	0.197	-0.025	-0.155	0.104	0.081	-0.041	0.204	0.028	-0.098	0.154	0.027	-0.093	0.147
	Asian or Asian British	-0.176	-0.479	0.127	-0.214	-0.440	0.011	-0.223	-0.492	0.046	-0.077	-0.341	0.187	-0.048	-0.281	0.186
	Mixed/Dual background	0.195	-0.002	0.393	-0.031	-0.197	0.134	0.010	-0.142	0.163	-0.111	-0.254	0.032	-0.058	-0.213	0.097
	Any other ethnic group	0.035	-0.263	0.333	-0.018	-0.311	0.275	0.024	-0.207	0.256	0.193	-0.021	0.407	-0.143	-0.365	0.079
SES	IMD 2010	0.388	0.147	0.630	0.316	0.090	0.542	0.433	0.222	0.644	0.366	0.147	0.586	0.421	0.218	0.623
NCMP participation (non-participation >0.2)		-0.025	-0.097	0.046	-0.020	-0.091	0.051	0.014	-0.072	0.100	0.033	-0.049	0.115	0.041	-0.070	0.152
Factor 3		0.050	-0.045	0.145	0.059	-0.028	0.145	0.020	-0.061	0.102	0.041	-0.044	0.126	0.062	-0.014	0.138
Year group	Reception	(ref)	(ref)	(ref)	(ref)	(ref)	(ref)	(ref)	(ref)	(ref)	(ref)	(ref)	(ref)	(ref)	(ref)	(ref)
	Year 6	0.318	0.086	0.550	0.112	-0.121	0.345	0.100	-0.119	0.320	0.081	-0.138	0.299	0.094	-0.114	0.302
Year group*factor	Reception	(ref)	(ref)	(ref)	(ref)	(ref)	(ref)	(ref)	(ref)	(ref)	(ref)	(ref)	(ref)	(ref)	(ref)	(ref)
	Year 6	-0.111	-0.224	0.002	-0.028	-0.139	0.083	-0.056	-0.161	0.048	-0.007	-0.111	0.096	-0.048	-0.147	0.051

Supporting file 3 to Williams, A.J., Wyatt, K.M., Williams, C.A., Logan, S., and Henley, W.E. Exploring the potential of a school impact on pupil weight status: exploratory factor analysis and repeat cross-sectional study of the National Child Measurement Programme.

S3 file - Detailed results and sensitivity analysis

Factor 4 - Prioritisation of physical activity

Red shading indicates significance (p<0.05), Green shading indicates modles which had to be estimated in R

Overweight (UK90)

**Single level**		**2006/07**			**2007/08**			**2008/09**			**2009/10**			**2010/11**		
n		8747			10061			10149			10356			9604		
Log likelihood	**Null**	-3723.27			-4273.38			-4660.99			-4699.66			-4334.16		
	**Individual**	-3717.66			-4267.11			-4657.93			-4694.99			-4326.89		
	**Factor**	-3717.53			-4266.39			-4657.58			-4694.47			-4326.43		
**Individual**		**Odds Ratio**	**LCI**	**UCI**	**Odds Ratio**	**LCI**	**UCI**	**Odds Ratio**	**LCI**	**UCI**	**Odds Ratio**	**LCI**	**UCI**	**Odds Ratio**	**LCI**	**UCI**
Gender	Male	(ref)	(ref)	(ref)	(ref)	(ref)	(ref)	(ref)	(ref)	(ref)	(ref)	(ref)	(ref)	(ref)	(ref)	(ref)
	Female	0.880	0.783	0.990	0.897	0.804	1.001	0.933	0.842	1.035	0.920	0.830	1.020	0.856	0.769	0.953
Ethnicity	White - British	(ref)	(ref)	(ref)	(ref)	(ref)	(ref)	(ref)	(ref)	(ref)	(ref)	(ref)	(ref)	(ref)	(ref)	(ref)
	Any other White background	0.935	0.610	1.435	0.813	0.559	1.184	0.947	0.675	1.329	1.086	0.780	1.512	1.191	0.876	1.621
	Asian or Asian British	0.542	0.194	1.518	0.895	0.471	1.701	0.776	0.348	1.728	0.993	0.484	2.041	0.775	0.383	1.571
	Mixed/Dual background	0.998	0.563	1.767	0.733	0.441	1.220	0.816	0.523	1.275	1.011	0.688	1.484	0.868	0.555	1.357
	Any other ethnic group	1.197	0.556	2.577	0.908	0.382	2.156	0.571	0.260	1.254	0.871	0.470	1.614	1.102	0.616	1.970
SES	IMD 2010	1.873	1.055	3.324	1.888	1.095	3.256	1.163	0.698	1.936	1.719	1.033	2.858	1.356	0.792	2.320
NCMP participation (non-participation >0.2)		0.964	0.814	1.142	1.079	0.923	1.262	1.076	0.889	1.302	1.126	0.946	1.341	1.280	0.971	1.686
**Factor**		**Odds Ratio**	**LCI**	**UCI**	**Odds Ratio**	**LCI**	**UCI**	**Odds Ratio**	**LCI**	**UCI**	**Odds Ratio**	**LCI**	**UCI**	**Odds Ratio**	**LCI**	**UCI**
Gender	Male	(ref)	(ref)	(ref)	(ref)	(ref)	(ref)	(ref)	(ref)	(ref)	(ref)	(ref)	(ref)	(ref)	(ref)	(ref)
	Female	0.880	0.783	0.990	0.897	0.804	1.001	0.933	0.842	1.035	0.921	0.831	1.021	0.856	0.769	0.953
Ethnicity	White - British	(ref)	(ref)	(ref)	(ref)	(ref)	(ref)	(ref)	(ref)	(ref)	(ref)	(ref)	(ref)	(ref)	(ref)	(ref)
	Any other White background	0.937	0.610	1.437	0.813	0.559	1.184	0.947	0.674	1.329	1.088	0.781	1.516	1.188	0.873	1.616
	Asian or Asian British	0.546	0.195	1.528	0.885	0.466	1.684	0.783	0.351	1.745	1.006	0.490	2.069	0.764	0.377	1.549
	Mixed/Dual background	1.000	0.565	1.773	0.733	0.440	1.219	0.818	0.524	1.277	1.016	0.692	1.493	0.866	0.554	1.353
	Any other ethnic group	1.205	0.559	2.597	0.898	0.378	2.134	0.577	0.263	1.267	0.878	0.474	1.627	1.095	0.612	1.959
SES	IMD 2010	1.910	1.070	3.408	1.799	1.039	3.114	1.199	0.716	2.010	1.777	1.063	2.971	1.307	0.760	2.245
NCMP participation (non-participation >0.2)		0.961	0.811	1.140	1.090	0.931	1.276	1.074	0.888	1.299	1.129	0.948	1.344	1.297	0.983	1.711
Factor 4		0.985	0.929	1.044	1.035	0.979	1.094	0.978	0.929	1.030	0.973	0.924	1.025	1.028	0.972	1.086

**Reception two-level**		**2006/07**			**2007/08**			**2008/09**			**2009/10**			**2010/11**		
n		4002			4769			4889			4908			4520		
Log likelihood	**Null**	-1621.95			-1944.48			-2250.55			-2162.02			-2008.25		
	**Individual**	-1619.64			-1932.20			-2246.54			-2158.56			-2003.00		
	**Factor**	-1619.60			-1932.20			-2246.46			-2157.43			-2002.87		
School variance	**Null**	0.084	0.024	0.286	0.012	0.000	3.544	0.088	0.039	0.200	0.027	0.002	0.300	0.018	4.06E-04	0.777
	**Individual**	0.080	0.022	0.288	1.64E-08	0 .		0.087	0.038	0.197	0.025	0.002	0.338	0.016	2.79E-04	0.961
	**Factor**	0.079	0.021	0.290	1.27E-10	0 .		0.087	0.038	0.197	0.026	0.002	0.309	0.014	1.36E-04	1.513
**Individual**		**Odds Ratio**	**LCI**	**UCI**	**Odds Ratio**	**LCI**	**UCI**	**Odds Ratio**	**LCI**	**UCI**	**Odds Ratio**	**LCI**	**UCI**	**Odds Ratio**	**LCI**	**UCI**
Gender	Male	(ref)	(ref)	(ref)	(ref)	(ref)	(ref)	(ref)	(ref)	(ref)	(ref)	(ref)	(ref)	(ref)	(ref)	(ref)
	Female	0.914	0.763	1.095	0.830	0.704	0.977	0.941	0.810	1.094	0.875	0.751	1.020	0.841	0.718	0.986
Ethnicity	White - British	(ref)	(ref)	(ref)	(ref)	(ref)	(ref)	(ref)	(ref)	(ref)	(ref)	(ref)	(ref)	(ref)	(ref)	(ref)
	Any other White background	0.709	0.383	1.310	0.639	0.378	1.078	0.801	0.486	1.322	1.365	0.885	2.105	1.147	0.758	1.735
	Asian or Asian British	0.913	0.264	3.154	0.406	0.125	1.321	0.373	0.086	1.610	0.563	0.170	1.867	0.671	0.260	1.735
	Mixed/Dual background	1.011	0.421	2.427	0.322	0.117	0.884	0.817	0.457	1.462	0.854	0.491	1.485	0.808	0.436	1.496
	Any other ethnic group	1.262	0.469	3.397	0.597	0.138	2.575	1.021	0.414	2.517	0.995	0.412	2.399	0.668	0.234	1.905
SES	IMD 2010	1.336	0.535	3.337	2.754	1.257	6.038	1.534	0.706	3.332	1.407	0.659	3.006	1.493	0.672	3.319
NCMP participation (non-participation >0.2)		0.819	0.604	1.112	0.841	0.639	1.107	1.412	0.945	2.109	0.937	0.727	1.207	1.724	0.927	3.208
**Factor**		**Odds Ratio**	**LCI**	**UCI**	**Odds Ratio**	**LCI**	**UCI**	**Odds Ratio**	**LCI**	**UCI**	**Odds Ratio**	**LCI**	**UCI**	**Odds Ratio**	**LCI**	**UCI**
Gender	Male	(ref)	(ref)	(ref)	(ref)	(ref)	(ref)	(ref)	(ref)	(ref)	(ref)	(ref)	(ref)	(ref)	(ref)	(ref)
	Female	0.914	0.763	1.095	0.830	0.704	0.978	0.941	0.810	1.093	0.876	0.751	1.021	0.841	0.718	0.986
Ethnicity	White - British	(ref)	(ref)	(ref)	(ref)	(ref)	(ref)	(ref)	(ref)	(ref)	(ref)	(ref)	(ref)	(ref)	(ref)	(ref)
	Any other White background	0.707	0.383	1.307	0.639	0.378	1.079	0.802	0.486	1.323	1.368	0.887	2.110	1.143	0.756	1.730
	Asian or Asian British	0.906	0.262	3.134	0.406	0.125	1.320	0.371	0.086	1.602	0.577	0.174	1.914	0.664	0.257	1.718
	Mixed/Dual background	1.007	0.419	2.417	0.322	0.117	0.884	0.816	0.457	1.460	0.862	0.495	1.499	0.807	0.436	1.495
	Any other ethnic group	1.254	0.466	3.377	0.596	0.138	2.572	1.015	0.412	2.502	1.032	0.427	2.492	0.664	0.233	1.893
SES	IMD 2010	1.318	0.526	3.301	2.732	1.228	6.076	1.502	0.687	3.282	1.500	0.695	3.237	1.450	0.649	3.240
NCMP participation (non-participation >0.2)		0.822	0.605	1.116	0.843	0.639	1.112	1.419	0.949	2.122	0.952	0.738	1.228	1.750	0.939	3.262
Factor 4		1.015	0.920	1.120	1.005	0.922	1.094	1.017	0.934	1.107	0.939	0.865	1.019	1.021	0.940	1.110

**Year 6 two-level**		**2006/07**			**2007/08**			**2008/09**			**2009/10**			**2010/11**		
n		4745			5292			5260			5448			5084		
Log likelihood	**Null**	-2095.61			-2325.40			-2404.51			-2534.54			-2325.19		
	**Individual**	-2088.90			-2322.30			-2400.36			-2526.67			-2321.69		
	**Factor**	-2088.32			-2321.43			-2398.93			-2526.64			-2321.32		
School variance	**Null**	0.019	0.001	0.468	0.016	3.19E-04	0.763	0.043	0.009	0.200	0.026	0.003	0.216	5.17E-16	0 .	
	**Individual**	0.004	1.10E-08	1830.554	0.009	9.25E-06	7.967	0.040	0.008	0.207	0.020	0.001	0.288	7.71E-19	0 .	
	**Factor**	2.02E-04	#######	3.30E+112	0.008	3.97E-06	14.807	0.041	0.008	0.202	0.021	0.002	0.279	1.19E-13	0 .	
**Individual**		**Odds Ratio**	**LCI**	**UCI**	**Odds Ratio**	**LCI**	**UCI**	**Odds Ratio**	**LCI**	**UCI**	**Odds Ratio**	**LCI**	**UCI**	**Odds Ratio**	**LCI**	**UCI**
Gender	Male	(ref)	(ref)	(ref)	(ref)	(ref)	(ref)	(ref)	(ref)	(ref)	(ref)	(ref)	(ref)	(ref)	(ref)	(ref)
	Female	0.851	0.729	0.994	0.951	0.821	1.102	0.926	0.802	1.070	0.957	0.832	1.102	0.869	0.751	1.006
Ethnicity	White - British	(ref)	(ref)	(ref)	(ref)	(ref)	(ref)	(ref)	(ref)	(ref)	(ref)	(ref)	(ref)	(ref)	(ref)	(ref)
	Any other White background	1.418	0.764	2.632	1.154	0.668	1.994	1.099	0.685	1.763	0.842	0.499	1.423	1.269	0.797	2.021
	Asian or Asian British	0.241	0.032	1.791	1.604	0.721	3.570	1.232	0.458	3.317	1.633	0.638	4.182	0.897	0.308	2.613
	Mixed/Dual background	1.001	0.467	2.144	1.233	0.670	2.268	0.755	0.371	1.537	1.210	0.702	2.085	0.958	0.499	1.839
	Any other ethnic group	1.113	0.318	3.891	1.198	0.403	3.558	0.159	0.022	1.167	0.781	0.327	1.866	1.497	0.735	3.051
SES	IMD 2010	2.488	1.141	5.426	1.398	0.646	3.023	0.865	0.406	1.845	2.322	1.121	4.810	1.247	0.596	2.610
NCMP participation (non-participation >0.2)		1.047	0.845	1.296	1.199	0.986	1.459	0.977	0.768	1.242	1.487	1.143	1.933	1.172	0.858	1.602
**Factor**		**Odds Ratio**	**LCI**	**UCI**	**Odds Ratio**	**LCI**	**UCI**	**Odds Ratio**	**LCI**	**UCI**	**Odds Ratio**	**LCI**	**UCI**	**Odds Ratio**	**LCI**	**UCI**
Gender	Male	(ref)	(ref)	(ref)	(ref)	(ref)	(ref)	(ref)	(ref)	(ref)	(ref)	(ref)	(ref)	(ref)	(ref)	(ref)
	Female	0.850	0.728	0.993	0.950	0.820	1.101	0.925	0.800	1.069	0.957	0.832	1.101	0.869	0.751	1.007
Ethnicity	White - British	(ref)	(ref)	(ref)	(ref)	(ref)	(ref)	(ref)	(ref)	(ref)	(ref)	(ref)	(ref)	(ref)	(ref)	(ref)
	Any other White background	1.411	0.761	2.618	1.151	0.666	1.988	1.104	0.688	1.771	0.841	0.498	1.422	1.264	0.794	2.014
	Asian or Asian British	0.243	0.033	1.806	1.579	0.709	3.517	1.269	0.471	3.419	1.625	0.634	4.165	0.881	0.302	2.569
	Mixed/Dual background	1.004	0.469	2.150	1.229	0.668	2.261	0.759	0.373	1.545	1.207	0.700	2.080	0.954	0.497	1.833
	Any other ethnic group	1.126	0.322	3.935	1.184	0.399	3.518	0.162	0.022	1.194	0.781	0.327	1.866	1.490	0.731	3.036
SES	IMD 2010	2.654	1.207	5.833	1.326	0.613	2.865	0.931	0.433	2.004	2.298	1.104	4.784	1.197	0.570	2.515
NCMP participation (non-participation >0.2)		1.040	0.841	1.287	1.210	0.995	1.473	0.976	0.767	1.240	1.489	1.144	1.937	1.189	0.868	1.627
Factor 4		0.958	0.886	1.035	1.051	0.975	1.133	0.936	0.866	1.011	1.009	0.939	1.084	1.034	0.958	1.115

**Three-level**		**2006/07**			**2007/08**			**2008/09**			**2009/10**			**2010/11**		
n		8747			10061			10149			10356			9604		
Log likelihood	**Null**	-3721.38			-4272.82			-4655.48			-4698.53			-4334.14		
	**Individual**	-3716.23			-4266.83			-4652.40			-4693.85			-4326.84		
	**Factor**	-3716.13			-4266.14			-4651.95			-4693.40			-4326.41		
	**Interaction**	-3712.24			-4263.17			-4650.74			-4689.96			-4325.96		
School variance	**Null**	2.31E-11	0 .		0.006	2.30E-06	16.533	0.001	7.92E-22	1.86E+15	0.003	3.68E-10	21371.7	0.003		
	**Individual**	5.24E-18	0 .		0.001	2.70E-24	3.73E+17	9.97E-09	0 .		0.003	1.66E-09	5768.52	0.005	8.60E-06	2.605
	**Factor**	7.77E-13	0 .		0.001	1.24E-24	7.87E+17	3.12E-09	0 .		0.002	6.91E-12	742658	0.003	5.18E-08	142.427
	**Interaction**	4.97E-16	0 .		0.007	0.0000152	3.684	2.30E-04			0.006	5.09E-06	7.427	0.003	2.20E-08	290.693
Year group variar	**Null**	0.048	0.015	0.154	0.017	3.49E-04	0.813	0.066	0.023	0.190	0.026	0.002	0.304	9.93E-12		
	**Individual**	0.042	0.012	0.156	0.016	2.63E-04	0.943	0.067	0.033	0.138	0.026	0.002	0.305	7.28E-14	0 .	
	**Factor**	0.042	0.011	0.156	0.015	0.000	1.147	0.068	0.033	0.138	0.026	0.002	0.302	8.86E-13	0 .	
	**Interaction**	0.031	0.006	0.172	0.001	1.31E-45	3.11E+38	0.066			0.020	0.001	0.453	1.70E-12	0 .	
**Individual**		**Odds Ratio**	**LCI**	**UCI**	**Odds Ratio**	**LCI**	**UCI**	**Odds Ratio**	**LCI**	**UCI**	**Odds Ratio**	**LCI**	**UCI**	**Odds Ratio**	**LCI**	**UCI**
Gender	Male	(ref)	(ref)	(ref)	(ref)	(ref)	(ref)	(ref)	(ref)	(ref)	(ref)	(ref)	(ref)	(ref)	(ref)	(ref)
	Female	0.880	0.782	0.990	0.897	0.804	1.001	0.93	0.84	1.03	0.921	0.831	1.022	0.856	0.768	0.953
Ethnicity	White - British	(ref)	(ref)	(ref)	(ref)	(ref)	(ref)	(ref)	(ref)	(ref)	(ref)	(ref)	(ref)	(ref)	(ref)	(ref)
	Any other White background	0.934	0.607	1.438	0.817	0.560	1.191	0.93	0.66	1.32	1.096	0.785	1.529	1.193	0.876	1.623
	Asian or Asian British	0.540	0.192	1.518	0.894	0.470	1.703	0.76	0.34	1.71	0.998	0.484	2.056	0.773	0.381	1.567
	Mixed/Dual background	0.992	0.558	1.762	0.734	0.441	1.222	0.80	0.51	1.25	1.011	0.687	1.487	0.867	0.555	1.356
	Any other ethnic group	1.190	0.549	2.580	0.904	0.380	2.153	0.57	0.26	1.26	0.882	0.474	1.638	1.103	0.616	1.973
SES	IMD 2010	1.789	0.984	3.253	1.873	1.074	3.267	1.15	0.67	1.98	1.764	1.040	2.991	1.357	0.789	2.333
NCMP participation (non-participation >0.2)		0.959	0.802	1.147	1.079	0.919	1.266	1.07	0.87	1.32	1.132	0.943	1.360	1.285	0.972	1.698
**Factor**		**Odds Ratio**	**LCI**	**UCI**	**Odds Ratio**	**LCI**	**UCI**	**Odds Ratio**	**LCI**	**UCI**	**Odds Ratio**	**LCI**	**UCI**	**Odds Ratio**	**LCI**	**UCI**
Gender	Male	(ref)	(ref)	(ref)	(ref)	(ref)	(ref)	(ref)	(ref)	(ref)	(ref)	(ref)	(ref)	(ref)	(ref)	(ref)
	Female	0.880	0.782	0.989	0.897	0.804	1.001	0.933	0.841	1.035	0.922	0.831	1.022	0.856	0.769	0.953
Ethnicity	White - British	(ref)	(ref)	(ref)	(ref)	(ref)	(ref)	(ref)	(ref)	(ref)	(ref)	(ref)	(ref)	(ref)	(ref)	(ref)
	Any other White background	0.935	0.608	1.440	0.817	0.560	1.191	0.935	0.663	1.318	1.097	0.786	1.531	1.188	0.873	1.618
	Asian or Asian British	0.543	0.193	1.527	0.885	0.465	1.686	0.770	0.343	1.728	1.010	0.490	2.081	0.762	0.376	1.547
	Mixed/Dual background	0.994	0.559	1.767	0.733	0.440	1.221	0.801	0.511	1.255	1.016	0.691	1.495	0.865	0.553	1.353
	Any other ethnic group	1.197	0.552	2.597	0.895	0.376	2.131	0.575	0.260	1.270	0.887	0.477	1.649	1.096	0.612	1.961
SES	IMD 2010	1.820	0.996	3.325	1.789	1.023	3.128	1.187	0.686	2.055	1.817	1.067	3.093	1.309	0.759	2.256
NCMP participation (non-participation >0.2)		0.957	0.800	1.144	1.089	0.927	1.280	1.070	0.870	1.318	1.134	0.945	1.362	1.299	0.983	1.717
Factor 4		0.986	0.926	1.049	1.035	0.977	1.096	0.973	0.918	1.030	0.974	0.922	1.028	1.027	0.971	1.086
**Interaction**		**Odds Ratio**	**LCI**	**UCI**	**Odds Ratio**	**LCI**	**UCI**	**Odds Ratio**	**LCI**	**UCI**	**Odds Ratio**	**LCI**	**UCI**	**Odds Ratio**	**LCI**	**UCI**
Gender	Male	(ref)	(ref)	(ref)	(ref)	(ref)	(ref)	(ref)	(ref)	(ref)	(ref)	(ref)	(ref)	(ref)	(ref)	(ref)
	Female	0.879	0.781	0.988	0.898	0.805	1.002	0.932	0.840	1.034	0.922	0.831	1.022	0.856	0.769	0.954
Ethnicity	White - British	(ref)	(ref)	(ref)	(ref)	(ref)	(ref)	(ref)	(ref)	(ref)	(ref)	(ref)	(ref)	(ref)	(ref)	(ref)
	Any other White background	0.959	0.623	1.477	0.833	0.572	1.215	0.937	0.665	1.322	1.105	0.792	1.541	1.196	0.878	1.628
	Asian or Asian British	0.543	0.193	1.526	0.894	0.470	1.703	0.769	0.342	1.731	1.020	0.495	2.104	0.769	0.379	1.560
	Mixed/Dual background	0.992	0.559	1.762	0.739	0.444	1.230	0.800	0.510	1.255	1.025	0.697	1.509	0.870	0.556	1.361
	Any other ethnic group	1.218	0.562	2.640	0.904	0.380	2.150	0.573	0.259	1.270	0.899	0.483	1.671	1.095	0.612	1.960
SES	IMD 2010	1.885	1.035	3.431	1.805	1.032	3.155	1.180	0.682	2.041	1.830	1.074	3.118	1.318	0.765	2.271
NCMP participation (non-participation >0.2)		0.949	0.795	1.133	1.073	0.915	1.259	1.075	0.872	1.326	1.165	0.970	1.399	1.281	0.967	1.696
Factor 4		1.019	0.929	1.117	1.022	0.939	1.114	1.019	0.938	1.106	0.931	0.859	1.010	1.019	0.940	1.105
Year group	Reception	(ref)	(ref)	(ref)	(ref)	(ref)	(ref)	(ref)	(ref)	(ref)	(ref)	(ref)	(ref)	(ref)	(ref)	(ref)
	Year 6	1.411	0.950	2.096	1.068	0.745	1.531	1.294	0.906	1.846	0.884	0.631	1.239	1.004	0.706	1.427
Year group*factor	Reception	(ref)	(ref)	(ref)	(ref)	(ref)	(ref)	(ref)	(ref)	(ref)	(ref)	(ref)	(ref)	(ref)	(ref)	(ref)
	Year 6	0.944	0.835	1.068	1.024	0.916	1.145	0.914	0.816	1.024	1.085	0.974	1.209	1.015	0.910	1.133

Supporting file 3 to Williams, A.J., Wyatt, K.M., Williams, C.A., Logan, S., and Henley, W.E. Exploring the potential of a school impact on pupil weight status: exploratory factor analysis and repeat cross-sectional study of the National Child Measurement Programme.

S3 file - Detailed results and sensitivity analysis

Factor 4 - Prioritisation of physical activity

Red shading indicates significance (p<0.05), Green shading indicates modles which had to be estimated in R

Obese (UK90)

Single level		2006/07			2007/08			2008/09			2009/10			2010/11		
n		9910			11353			11696			11851			11016		
Log likelihood	**Null**	-3583.69			-4023.42			-4569.33			-4491.54			-4218.10		
	**Individual**	-3567.36			-4005.35			-4548.51			-4469.42			-4184.92		
	**Factor**	-3566.89			-4004.38			-4546.83			-4469.38			-4184.92		
**Individual**		**Odds Ratio**	**LCI**	**UCI**	**Odds Ratio**	**LCI**	**UCI**	**Odds Ratio**	**LCI**	**UCI**	**Odds Ratio**	**LCI**	**UCI**	**Odds Ratio**	**LCI**	**UCI**
Gender	Male	(ref)	(ref)	(ref)	(ref)	(ref)	(ref)	(ref)	(ref)	(ref)	(ref)	(ref)	(ref)	(ref)	(ref)	(ref)
	Female	0.807	0.714	0.913	0.765	0.680	0.860	0.780	0.700	0.869	0.839	0.752	0.935	0.711	0.635	0.797
Ethnicity	White - British	(ref)	(ref)	(ref)	(ref)	(ref)	(ref)	(ref)	(ref)	(ref)	(ref)	(ref)	(ref)	(ref)	(ref)	(ref)
	Any other White background	0.880	0.551	1.404	0.986	0.679	1.433	1.154	0.830	1.604	1.000	0.698	1.433	0.786	0.542	1.140
	Asian or Asian British	0.835	0.330	2.112	1.134	0.614	2.097	1.031	0.487	2.183	1.071	0.526	2.181	1.071	0.563	2.039
	Mixed/Dual background	1.962	1.246	3.090	0.960	0.586	1.573	1.049	0.685	1.607	0.942	0.622	1.426	0.949	0.604	1.490
	Any other ethnic group	1.179	0.530	2.625	1.630	0.791	3.359	1.355	0.757	2.426	1.285	0.737	2.240	0.618	0.284	1.344
SES	IMD 2010	2.893	1.611	5.196	2.992	1.704	5.256	3.052	1.847	5.043	3.977	2.371	6.669	4.059	2.394	6.880
NCMP participation (non-participation >0.2)		1.069	0.899	1.270	0.996	0.841	1.181	1.093	0.898	1.330	0.745	0.604	0.920	1.257	0.948	1.667
**Factor**		**Odds Ratio**	**LCI**	**UCI**	**Odds Ratio**	**LCI**	**UCI**	**Odds Ratio**	**LCI**	**UCI**	**Odds Ratio**	**LCI**	**UCI**	**Odds Ratio**	**LCI**	**UCI**
Gender	Male	(ref)	(ref)	(ref)	(ref)	(ref)	(ref)	(ref)	(ref)	(ref)	(ref)	(ref)	(ref)	(ref)	(ref)	(ref)
	Female	0.807	0.714	0.913	0.765	0.680	0.860	0.779	0.700	0.868	0.838	0.752	0.935	0.711	0.635	0.797
Ethnicity	White - British	(ref)	(ref)	(ref)	(ref)	(ref)	(ref)	(ref)	(ref)	(ref)	(ref)	(ref)	(ref)	(ref)	(ref)	(ref)
	Any other White background	0.883	0.553	1.409	0.984	0.677	1.430	1.154	0.830	1.604	1.000	0.698	1.432	0.786	0.542	1.140
	Asian or Asian British	0.847	0.335	2.143	1.119	0.605	2.069	1.011	0.477	2.142	1.066	0.523	2.173	1.071	0.562	2.040
	Mixed/Dual background	1.975	1.254	3.110	0.959	0.585	1.572	1.044	0.681	1.599	0.941	0.621	1.425	0.949	0.604	1.490
	Any other ethnic group	1.195	0.537	2.661	1.607	0.780	3.313	1.326	0.740	2.374	1.281	0.735	2.234	0.618	0.284	1.344
SES	IMD 2010	3.024	1.669	5.478	2.807	1.590	4.954	2.817	1.696	4.679	3.927	2.327	6.626	4.057	2.374	6.930
NCMP participation (non-participation >0.2)		1.063	0.894	1.263	1.009	0.851	1.197	1.097	0.901	1.335	0.745	0.603	0.920	1.258	0.947	1.670
Factor 4		0.970	0.913	1.031	1.043	0.983	1.107	1.052	0.996	1.111	1.008	0.954	1.065	1.000	0.944	1.060

Reception two-level		2006/07			2007/08			2008/09			2009/10			2010/11		
n		4352			5190			5421			5373			4970		
Log likelihood	**Null**	-1217.69			-1459.60			-1732.98			-1560.98			-1507.99		
	**Individual**	-1210.86			-1447.28			-1727.23			-1551.17			-1497.76		
	**Factor**	-1210.86			-1447.26			-1725.92			-1550.91			-1497.67		
School variance	**Null**	3.47E-20	0 .		0.091	0.023	0.364	0.145	0.071	0.298	0.305	0.175	0.530	0.106	0.032	0.351
	**Individual**	8.49E-13	0 .		0.049	0.004	0.568	0.136	0.064	0.288	0.235	0.123	0.450	0.105	0.032	0.338
	**Factor**	2.60E-17	0 .		0.048	0.004	0.592	0.133	0.062	0.286	0.231	0.120	0.447	0.103	0.032	0.336
**Individual**		**Odds Ratio**	**LCI**	**UCI**	**Odds Ratio**	**LCI**	**UCI**	**Odds Ratio**	**LCI**	**UCI**	**Odds Ratio**	**LCI**	**UCI**	**Odds Ratio**	**LCI**	**UCI**
Gender	Male	(ref)	(ref)	(ref)	(ref)	(ref)	(ref)	(ref)	(ref)	(ref)	(ref)	(ref)	(ref)	(ref)	(ref)	(ref)
	Female	0.824	0.661	1.027	0.821	0.671	1.005	0.797	0.664	0.956	0.764	0.628	0.930	0.703	0.577	0.858
Ethnicity	White - British	(ref)	(ref)	(ref)	(ref)	(ref)	(ref)	(ref)	(ref)	(ref)	(ref)	(ref)	(ref)	(ref)	(ref)	(ref)
	Any other White background	1.028	0.533	1.985	1.299	0.791	2.133	1.151	0.672	1.971	0.665	0.331	1.338	1.077	0.640	1.812
	Asian or Asian British	1.996	0.680	5.860	0.897	0.315	2.553	0.626	0.144	2.717	1.477	0.556	3.927	0.919	0.322	2.618
	Mixed/Dual background	1.872	0.834	4.203	0.443	0.139	1.415	1.082	0.569	2.056	1.064	0.560	2.020	1.135	0.581	2.217
	Any other ethnic group	0.405	0.055	2.989	0.495	0.066	3.733	1.091	0.378	3.150	0.758	0.227	2.533	1.222	0.426	3.502
SES	IMD 2010	3.599	1.308	9.900	7.312	2.861	18.687	2.478	1.010	6.082	4.185	1.559	11.238	3.945	1.511	10.298
NCMP participation (non-participation >0.2)		1.010	0.726	1.405	0.843	0.592	1.199	1.276	0.781	2.083	0.702	0.467	1.057	1.236	0.537	2.842
**Factor**		**Odds Ratio**	**LCI**	**UCI**	**Odds Ratio**	**LCI**	**UCI**	**Odds Ratio**	**LCI**	**UCI**	**Odds Ratio**	**LCI**	**UCI**	**Odds Ratio**	**LCI**	**UCI**
Gender	Male	(ref)	(ref)	(ref)	(ref)	(ref)	(ref)	(ref)	(ref)	(ref)	(ref)	(ref)	(ref)	(ref)	(ref)	(ref)
	Female	0.824	0.661	1.027	0.822	0.671	1.006	0.796	0.663	0.955	0.763	0.627	0.929	0.703	0.577	0.858
Ethnicity	White - British	(ref)	(ref)	(ref)	(ref)	(ref)	(ref)	(ref)	(ref)	(ref)	(ref)	(ref)	(ref)	(ref)	(ref)	(ref)
	Any other White background	1.028	0.533	1.986	1.299	0.791	2.132	1.156	0.675	1.978	0.664	0.330	1.334	1.079	0.641	1.816
	Asian or Asian British	1.996	0.679	5.872	0.894	0.314	2.545	0.611	0.141	2.650	1.457	0.548	3.875	0.929	0.326	2.652
	Mixed/Dual background	1.873	0.833	4.208	0.443	0.139	1.415	1.076	0.566	2.045	1.060	0.558	2.012	1.136	0.581	2.219
	Any other ethnic group	0.405	0.055	2.993	0.493	0.065	3.721	1.060	0.367	3.062	0.745	0.223	2.489	1.232	0.429	3.533
SES	IMD 2010	3.601	1.292	10.036	7.146	2.742	18.627	2.222	0.902	5.473	4.003	1.486	10.783	4.081	1.542	10.799
NCMP participation (non-participation >0.2)		1.010	0.725	1.406	0.848	0.594	1.211	1.305	0.798	2.136	0.697	0.463	1.049	1.214	0.526	2.800
Factor 4		1.000	0.896	1.116	1.013	0.907	1.130	1.093	0.981	1.217	1.045	0.926	1.180	0.977	0.876	1.089

Year 6 two-level		2006/07			2007/08			2008/09			2009/10			2010/11		
n		5558			6163			6275			6478			6046		
Log likelihood	**Null**	-2312.74			-2506.79			-2771.22			-2833.62			-2648.44		
	**Individual**	-2299.05			-2494.93			-2756.94			-2825.60			-2624.68		
	**Factor**	-2298.58			-2494.07			-2756.21			-2825.60			-2624.54		
School variance	**Null**	0.027	0.003	0.261	0.089	0.037	0.218	0.092	0.043	0.197	0.074	0.030	0.179	0.033	0.006	0.173
	**Individual**	0.014	2.27E-04	0.906	0.086	0.034	0.217	0.075	0.032	0.177	0.066	0.025	0.174	0.005	3.68E-07	71.441
	**Factor**	0.010	0.0000285	3.502	0.081	0.031	0.215	0.076	0.032	0.177	0.066	0.025	0.174	0.005	4.93E-07	56.453
**Individual**		**Odds Ratio**	**LCI**	**UCI**	**Odds Ratio**	**LCI**	**UCI**	**Odds Ratio**	**LCI**	**UCI**	**Odds Ratio**	**LCI**	**UCI**	**Odds Ratio**	**LCI**	**UCI**
Gender	Male	(ref)	(ref)	(ref)	(ref)	(ref)	(ref)	(ref)	(ref)	(ref)	(ref)	(ref)	(ref)	(ref)	(ref)	(ref)
	Female	0.794	0.683	0.923	0.737	0.636	0.854	0.778	0.679	0.893	0.886	0.774	1.014	0.717	0.623	0.826
Ethnicity	White - British	(ref)	(ref)	(ref)	(ref)	(ref)	(ref)	(ref)	(ref)	(ref)	(ref)	(ref)	(ref)	(ref)	(ref)	(ref)
	Any other White background	0.986	0.502	1.935	0.881	0.487	1.594	1.201	0.782	1.846	1.288	0.831	1.997	0.683	0.395	1.178
	Asian or Asian British	0.266	0.036	1.977	1.319	0.599	2.908	1.213	0.490	3.000	0.897	0.306	2.628	1.351	0.581	3.140
	Mixed/Dual background	2.028	1.160	3.545	1.359	0.768	2.406	1.106	0.614	1.992	1.001	0.570	1.756	0.944	0.507	1.759
	Any other ethnic group	2.111	0.826	5.395	2.222	0.970	5.088	1.588	0.769	3.282	1.571	0.816	3.024	0.367	0.113	1.188
SES	IMD 2010	2.999	1.428	6.296	1.766	0.804	3.879	3.418	1.709	6.835	3.201	1.569	6.532	4.870	2.500	9.487
NCMP participation (non-participation >0.2)		1.030	0.836	1.270	0.936	0.746	1.173	0.870	0.680	1.113	1.007	0.754	1.346	1.041	0.766	1.415
**Factor**		**Odds Ratio**	**LCI**	**UCI**	**Odds Ratio**	**LCI**	**UCI**	**Odds Ratio**	**LCI**	**UCI**	**Odds Ratio**	**LCI**	**UCI**	**Odds Ratio**	**LCI**	**UCI**
Gender	Male	(ref)	(ref)	(ref)	(ref)	(ref)	(ref)	(ref)	(ref)	(ref)	(ref)	(ref)	(ref)	(ref)	(ref)	(ref)
	Female	0.794	0.683	0.923	0.737	0.636	0.854	0.778	0.679	0.893	0.886	0.775	1.015	0.717	0.623	0.826
Ethnicity	White - British	(ref)	(ref)	(ref)	(ref)	(ref)	(ref)	(ref)	(ref)	(ref)	(ref)	(ref)	(ref)	(ref)	(ref)	(ref)
	Any other White background	0.984	0.501	1.933	0.878	0.486	1.588	1.197	0.779	1.840	1.289	0.831	1.999	0.681	0.394	1.175
	Asian or Asian British	0.268	0.036	1.994	1.301	0.590	2.868	1.195	0.483	2.958	0.898	0.306	2.635	1.337	0.574	3.111
	Mixed/Dual background	2.035	1.165	3.555	1.354	0.765	2.397	1.101	0.611	1.984	1.002	0.571	1.757	0.943	0.506	1.756
	Any other ethnic group	2.129	0.833	5.439	2.191	0.956	5.018	1.563	0.756	3.232	1.572	0.816	3.026	0.366	0.113	1.186
SES	IMD 2010	3.174	1.498	6.721	1.687	0.770	3.697	3.220	1.603	6.469	3.216	1.567	6.601	4.732	2.411	9.286
NCMP participation (non-participation >0.2)		1.025	0.833	1.262	0.946	0.755	1.185	0.869	0.679	1.112	1.006	0.753	1.346	1.048	0.770	1.427
Factor 4		0.963	0.892	1.039	1.056	0.973	1.146	1.049	0.970	1.134	0.996	0.924	1.073	1.020	0.948	1.097

Three-level		2006/07			2007/08			2008/09			2009/10			2010/11		
n		9910			11353			11696			11851			11016		
Log likelihood	**Null**	-3574.27			-4002.79			-4542.37			-4448.49			-4200.78		
	**Individual**	-3559.41			-3986.74			-4524.94			-4435.32			-4171.06		
	**Factor**	-3559.20			-3986.32			-4523.36			-4435.32			-4171.06		
	**Interaction**	-3512.36			-3946.54			-4483.47			-4382.78			-4124.71		
School variance	**Null**	2.61E-18	0 .		2.56E-14	0 .		1.74E-13	0 .		2.13E-17	0 .		1.23E-14	0 .	
	**Individual**	2.45E-21	0 .		6.01E-11	0 .		8.58E-11	0 .		7.45E-20	0 .		1.42E-12	0 .	
	**Factor**	2.64E-10	0 .		1.28E-11	0 .		9.33E-12	0 .		4.05E-19	0 .		1.43E-11	0 .	
	**Interaction**	0.023	0.003	0.174	0.059	0.018	0.191	0.041	0.010	0.173	0.043	0.010	0.193	0.032	0.009	0.112
Year group variai	**Null**	0.122	0.066	0.226	0.191	0.123	0.296	0.182	0.122	0.273	0.261	0.183	0.372	0.152	0.095	0.245
	**Individual**	0.114	0.059	0.219	0.181	0.114	0.286	0.167	0.109	0.255	0.233	0.159	0.341	0.132	0.079	0.222
	**Factor**	0.112	0.058	0.217	0.179	0.113	0.284	0.166	0.109	0.254	0.233	0.159	0.341	0.132	0.079	0.223
	**Interaction**	2.44E-13	0 .		0.021	0.001	0.837	0.057	0.016	0.199	0.075	0.025	0.228	6.32E-11	0 .	
**Individual**		**Odds Ratio**	**LCI**	**UCI**	**Odds Ratio**	**LCI**	**UCI**	**Odds Ratio**	**LCI**	**UCI**	**Odds Ratio**	**LCI**	**UCI**	**Odds Ratio**	**LCI**	**UCI**
Gender	Male	(ref)	(ref)	(ref)	(ref)	(ref)	(ref)	(ref)	(ref)	(ref)	(ref)	(ref)	(ref)	(ref)	(ref)	(ref)
	Female	0.800	0.706	0.906	0.758	0.673	0.854	0.783	0.702	0.874	0.839	0.751	0.938	0.707	0.630	0.793
Ethnicity	White - British	(ref)	(ref)	(ref)	(ref)	(ref)	(ref)	(ref)	(ref)	(ref)	(ref)	(ref)	(ref)	(ref)	(ref)	(ref)
	Any other White background	0.915	0.569	1.470	1.011	0.687	1.487	1.159	0.828	1.623	1.031	0.713	1.490	0.807	0.553	1.179
	Asian or Asian British	0.866	0.339	2.214	1.069	0.568	2.013	0.966	0.450	2.074	1.129	0.546	2.334	1.104	0.574	2.124
	Mixed/Dual background	1.991	1.254	3.159	0.983	0.595	1.623	1.066	0.691	1.645	1.014	0.664	1.548	0.965	0.610	1.524
	Any other ethnic group	1.242	0.550	2.806	1.543	0.735	3.241	1.392	0.767	2.526	1.313	0.741	2.327	0.621	0.283	1.361
SES	IMD 2010	2.669	1.413	5.042	2.756	1.471	5.167	2.895	1.646	5.094	3.216	1.762	5.871	3.856	2.156	6.895
NCMP participation (non-participation >0.2)		1.053	0.866	1.281	0.957	0.779	1.177	1.079	0.852	1.366	0.824	0.639	1.063	1.227	0.887	1.698
**Factor**		**Odds Ratio**	**LCI**	**UCI**	**Odds Ratio**	**LCI**	**UCI**	**Odds Ratio**	**LCI**	**UCI**	**Odds Ratio**	**LCI**	**UCI**	**Odds Ratio**	**LCI**	**UCI**
Gender	Male	(ref)	(ref)	(ref)	(ref)	(ref)	(ref)	(ref)	(ref)	(ref)	(ref)	(ref)	(ref)	(ref)	(ref)	(ref)
	Female	0.800	0.706	0.907	0.758	0.673	0.854	0.783	0.702	0.874	0.839	0.751	0.938	0.707	0.630	0.793
Ethnicity	White - British	(ref)	(ref)	(ref)	(ref)	(ref)	(ref)	(ref)	(ref)	(ref)	(ref)	(ref)	(ref)	(ref)	(ref)	(ref)
	Any other White background	0.916	0.570	1.473	1.009	0.686	1.485	1.158	0.827	1.622	1.031	0.713	1.490	0.807	0.553	1.178
	Asian or Asian British	0.874	0.342	2.233	1.060	0.563	1.996	0.950	0.442	2.040	1.128	0.545	2.333	1.103	0.573	2.123
	Mixed/Dual background	1.997	1.259	3.170	0.982	0.595	1.622	1.061	0.688	1.638	1.014	0.664	1.548	0.964	0.610	1.524
	Any other ethnic group	1.252	0.554	2.829	1.531	0.729	3.216	1.368	0.754	2.483	1.313	0.741	2.326	0.621	0.283	1.361
SES	IMD 2010	2.741	1.443	5.208	2.665	1.418	5.006	2.709	1.536	4.779	3.210	1.752	5.880	3.843	2.137	6.909
NCMP participation (non-participation >0.2)		1.049	0.863	1.276	0.967	0.786	1.189	1.082	0.854	1.371	0.824	0.639	1.063	1.229	0.887	1.702
Factor 4		0.977	0.910	1.048	1.034	0.962	1.111	1.063	0.993	1.138	1.002	0.933	1.077	1.003	0.937	1.073
**Interaction**		**Odds Ratio**	**LCI**	**UCI**	**Odds Ratio**	**LCI**	**UCI**	**Odds Ratio**	**LCI**	**UCI**	**Odds Ratio**	**LCI**	**UCI**	**Odds Ratio**	**LCI**	**UCI**
Gender	Male	(ref)	(ref)	(ref)	(ref)	(ref)	(ref)	(ref)	(ref)	(ref)	(ref)	(ref)	(ref)	(ref)	(ref)	(ref)
	Female	0.802	0.708	0.908	0.764	0.679	0.861	0.784	0.703	0.875	0.839	0.751	0.938	0.712	0.635	0.799
Ethnicity	White - British	(ref)	(ref)	(ref)	(ref)	(ref)	(ref)	(ref)	(ref)	(ref)	(ref)	(ref)	(ref)	(ref)	(ref)	(ref)
	Any other White background	1.011	0.630	1.621	1.120	0.764	1.642	1.181	0.844	1.652	1.049	0.727	1.514	0.856	0.588	1.246
	Asian or Asian British	0.900	0.354	2.291	1.116	0.594	2.095	0.969	0.452	2.077	1.187	0.575	2.453	1.176	0.613	2.257
	Mixed/Dual background	1.985	1.254	3.145	0.997	0.605	1.644	1.092	0.708	1.685	1.047	0.686	1.597	1.016	0.644	1.604
	Any other ethnic group	1.300	0.578	2.923	1.543	0.737	3.228	1.368	0.754	2.480	1.300	0.736	2.295	0.620	0.283	1.355
SES	IMD 2010	3.165	1.690	5.925	2.787	1.493	5.201	2.723	1.551	4.779	3.447	1.903	6.244	4.224	2.394	7.454
NCMP participation (non-participation >0.2)		1.026	0.857	1.228	0.918	0.760	1.110	0.923	0.736	1.158	0.853	0.673	1.081	1.069	0.795	1.438
Factor 4		1.003	0.898	1.120	1.028	0.921	1.148	1.082	0.977	1.200	1.049	0.939	1.172	0.974	0.880	1.077
Year group	Reception	(ref)	(ref)	(ref)	(ref)	(ref)	(ref)	(ref)	(ref)	(ref)	(ref)	(ref)	(ref)	(ref)	(ref)	(ref)
	Year 6	2.232	1.456	3.420	1.770	1.166	2.686	1.972	1.323	2.937	2.436	1.611	3.683	1.642	1.115	2.417
Year group*factor	Reception	(ref)	(ref)	(ref)	(ref)	(ref)	(ref)	(ref)	(ref)	(ref)	(ref)	(ref)	(ref)	(ref)	(ref)	(ref)
	Year 6	0.961	0.843	1.096	1.021	0.897	1.161	0.975	0.861	1.104	0.946	0.830	1.079	1.050	0.931	1.185

Supporting file 3 to Williams, A.J., Wyatt, K.M., Williams, C.A., Logan, S., and Henley, W.E. Exploring the potential of a school impact on pupil weight status: exploratory factor analysis and repeat cross-sectional study of the National Child Measurement Programme.

S3 file - Detailed results and sensitivity analysis

Factor 4 - Prioritisation of physical activity

Red shading indicates significance (p<0.05), Green shading indicates modles which had to be estimated in R

Overweight (IOTF)

**Single level**		**2006/07**			**2007/08**			**2008/09**			**2009/10**			**2010/11**		
n		9532			10955			11217			11345			10559		
Log likelihood	**Null**	-4160.76			-4786.96			-5421.59			-5292.83			-4916.86		
	**Individual**	-4143.36			-4767.80			-5401.96			-5271.96			-4908.28		
	**Factor**	-4143.34			-4765.17			-5401.94			-5270.46			-4907.27		
**Individual**		**Odds Ratio**	**LCI**	**UCI**	**Odds Ratio**	**LCI**	**UCI**	**Odds Ratio**	**LCI**	**UCI**	**Odds Ratio**	**LCI**	**UCI**	**Odds Ratio**	**LCI**	**UCI**
Gender	Male	(ref)	(ref)	(ref)	(ref)	(ref)	(ref)	(ref)	(ref)	(ref)	(ref)	(ref)	(ref)	(ref)	(ref)	(ref)
	Female	1.319	1.181	1.473	1.322	1.193	1.466	1.288	1.171	1.417	1.346	1.222	1.483	1.129	1.021	1.248
Ethnicity	White - British	(ref)	(ref)	(ref)	(ref)	(ref)	(ref)	(ref)	(ref)	(ref)	(ref)	(ref)	(ref)	(ref)	(ref)	(ref)
	Any other White background	1.047	0.705	1.554	0.807	0.563	1.157	1.115	0.830	1.499	1.071	0.781	1.467	1.089	0.808	1.467
	Asian or Asian British	0.738	0.312	1.744	0.779	0.411	1.475	0.910	0.458	1.809	0.964	0.486	1.912	0.761	0.388	1.490
	Mixed/Dual background	1.409	0.886	2.241	0.721	0.445	1.169	1.085	0.746	1.578	0.896	0.615	1.305	0.951	0.631	1.432
	Any other ethnic group	1.348	0.670	2.710	1.031	0.481	2.209	0.950	0.519	1.737	1.061	0.623	1.806	0.516	0.248	1.073
SES	IMD 2010	2.098	1.225	3.592	1.829	1.095	3.054	2.124	1.345	3.353	1.694	1.049	2.735	1.368	0.831	2.253
NCMP participation (non-participation >0.2)		1.071	0.917	1.250	1.077	0.930	1.247	1.099	0.923	1.308	0.963	0.810	1.144	1.358	1.058	1.743
**Factor**		**Odds Ratio**	**LCI**	**UCI**	**Odds Ratio**	**LCI**	**UCI**	**Odds Ratio**	**LCI**	**UCI**	**Odds Ratio**	**LCI**	**UCI**	**Odds Ratio**	**LCI**	**UCI**
Gender	Male	(ref)	(ref)	(ref)	(ref)	(ref)	(ref)	(ref)	(ref)	(ref)	(ref)	(ref)	(ref)	(ref)	(ref)	(ref)
	Female	1.319	1.181	1.473	1.323	1.194	1.467	1.288	1.171	1.417	1.348	1.223	1.485	1.129	1.021	1.248
Ethnicity	White - British	(ref)	(ref)	(ref)	(ref)	(ref)	(ref)	(ref)	(ref)	(ref)	(ref)	(ref)	(ref)	(ref)	(ref)	(ref)
	Any other White background	1.047	0.705	1.555	0.806	0.562	1.156	1.115	0.830	1.499	1.075	0.784	1.473	1.084	0.804	1.460
	Asian or Asian British	0.740	0.313	1.749	0.765	0.404	1.449	0.908	0.457	1.806	0.986	0.497	1.957	0.745	0.380	1.460
	Mixed/Dual background	1.411	0.887	2.244	0.720	0.444	1.168	1.085	0.746	1.577	0.901	0.619	1.313	0.949	0.630	1.430
	Any other ethnic group	1.352	0.672	2.720	1.010	0.471	2.166	0.948	0.518	1.734	1.076	0.632	1.831	0.511	0.246	1.064
SES	IMD 2010	2.115	1.229	3.639	1.678	1.002	2.809	2.108	1.327	3.347	1.788	1.101	2.903	1.301	0.787	2.149
NCMP participation (non-participation >0.2)		1.069	0.915	1.249	1.097	0.947	1.272	1.099	0.923	1.308	0.966	0.812	1.148	1.381	1.075	1.774
Factor 4		0.994	0.941	1.050	1.063	1.009	1.121	1.005	0.958	1.054	0.958	0.913	1.006	1.038	0.986	1.093

**Reception two-level**		**2006/07**			**2007/08**			**2008/09**			**2009/10**			**2010/11**		
n		4214			5017			5199			5165			4783		
Log likelihood	**Null**	-1716.68			-1998.43			-2399.35			-2231.13			-2109.18		
	**Individual**	-1704.54			-1975.03			-2381.45			-2215.84			-2098.70		
	**Factor**	-1704.32			-1974.97			-2381.45			-2214.79			-2098.48		
School variance	**Null**	0.045	0.007	0.277	0.097	0.038	0.250	0.127	0.071	0.229	0.037	0.005	0.276	0.057	0.015	0.209
	**Individual**	0.030	0.002	0.452	0.080	0.027	0.242	0.120	0.065	0.220	0.036	0.005	0.285	0.065	0.020	0.210
	**Factor**	0.027	0.001	0.514	0.080	0.027	0.242	0.120	0.065	0.220	0.037	0.005	0.276	0.063	0.019	0.211
**Individual**		**Odds Ratio**	**LCI**	**UCI**	**Odds Ratio**	**LCI**	**UCI**	**Odds Ratio**	**LCI**	**UCI**	**Odds Ratio**	**LCI**	**UCI**	**Odds Ratio**	**LCI**	**UCI**
Gender	Male	(ref)	(ref)	(ref)	(ref)	(ref)	(ref)	(ref)	(ref)	(ref)	(ref)	(ref)	(ref)	(ref)	(ref)	(ref)
	Female	1.510	1.267	1.800	1.521	1.290	1.793	1.467	1.267	1.699	1.471	1.262	1.713	1.355	1.159	1.585
Ethnicity	White - British	(ref)	(ref)	(ref)	(ref)	(ref)	(ref)	(ref)	(ref)	(ref)	(ref)	(ref)	(ref)	(ref)	(ref)	(ref)
	Any other White background	0.913	0.525	1.589	0.704	0.418	1.183	0.835	0.515	1.353	1.174	0.746	1.846	1.338	0.899	1.992
	Asian or Asian British	1.150	0.385	3.431	0.266	0.063	1.112	0.676	0.226	2.019	0.578	0.174	1.920	0.529	0.185	1.508
	Mixed/Dual background	1.236	0.572	2.674	0.334	0.121	0.922	1.189	0.722	1.959	0.877	0.513	1.502	1.270	0.751	2.149
	Any other ethnic group	1.004	0.343	2.937	0.563	0.129	2.454	1.251	0.534	2.928	0.929	0.385	2.246	0.486	0.148	1.602
SES	IMD 2010	2.056	0.862	4.905	3.447	1.515	7.841	1.738	0.810	3.728	1.793	0.845	3.802	1.169	0.519	2.632
NCMP participation (non-participation >0.2)		1.005	0.767	1.317	0.917	0.687	1.224	1.633	1.106	2.409	0.830	0.636	1.082	1.347	0.691	2.629
**Factor**		**Odds Ratio**	**LCI**	**UCI**	**Odds Ratio**	**LCI**	**UCI**	**Odds Ratio**	**LCI**	**UCI**	**Odds Ratio**	**LCI**	**UCI**	**Odds Ratio**	**LCI**	**UCI**
Gender	Male	(ref)	(ref)	(ref)	(ref)	(ref)	(ref)	(ref)	(ref)	(ref)	(ref)	(ref)	(ref)	(ref)	(ref)	(ref)
	Female	1.510	1.267	1.800	1.520	1.290	1.792	1.467	1.267	1.699	1.473	1.264	1.716	1.355	1.159	1.585
Ethnicity	White - British	(ref)	(ref)	(ref)	(ref)	(ref)	(ref)	(ref)	(ref)	(ref)	(ref)	(ref)	(ref)	(ref)	(ref)	(ref)
	Any other White background	0.908	0.522	1.580	0.703	0.418	1.182	0.835	0.515	1.353	1.178	0.749	1.853	1.334	0.896	1.985
	Asian or Asian British	1.131	0.379	3.375	0.267	0.064	1.118	0.676	0.226	2.021	0.593	0.178	1.971	0.522	0.183	1.488
	Mixed/Dual background	1.223	0.565	2.645	0.334	0.121	0.921	1.189	0.722	1.959	0.883	0.515	1.511	1.269	0.750	2.147
	Any other ethnic group	0.987	0.337	2.889	0.566	0.130	2.468	1.252	0.535	2.931	0.964	0.398	2.332	0.482	0.146	1.590
SES	IMD 2010	1.994	0.835	4.760	3.542	1.535	8.171	1.743	0.808	3.761	1.913	0.894	4.095	1.129	0.500	2.553
NCMP participation (non-participation >0.2)		1.012	0.772	1.326	0.909	0.679	1.218	1.631	1.104	2.409	0.843	0.646	1.100	1.377	0.704	2.693
Factor 4		1.031	0.942	1.130	0.983	0.895	1.079	0.997	0.914	1.088	0.941	0.866	1.022	1.030	0.944	1.123

**Year 6 two-level**		**2006/07**			**2007/08**			**2008/09**			**2009/10**			**2010/11**		
n		5318			5938			6018			6180			5776		
Log likelihood	**Null**	-2435.38			-2765.54			-3002.77			-3044.09			-2797.82		
	**Individual**	-2426.60			-2760.43			-2993.90			-3034.06			-2794.09		
	**Factor**	-2426.27			-2756.77			-2993.88			-3033.90			-2793.37		
School variance	**Null**	7.39E-16	0 .		0.075	0.029	0.196	0.051	0.017	0.155	0.049	0.016	0.153	0.038	0.009	0.160
	**Individual**	4.22E-16	0 .		0.071	0.026	0.194	0.050	0.016	0.152	0.046	0.014	0.153	0.031	0.005	0.172
	**Factor**	7.67E-16	0 .		0.054	0.015	0.190	0.050	0.016	0.152	0.045	0.013	0.153	0.030	0.005	0.173
**Individual**		**Odds Ratio**	**LCI**	**UCI**	**Odds Ratio**	**LCI**	**UCI**	**Odds Ratio**	**LCI**	**UCI**	**Odds Ratio**	**LCI**	**UCI**	**Odds Ratio**	**LCI**	**UCI**
Gender	Male	(ref)	(ref)	(ref)	(ref)	(ref)	(ref)	(ref)	(ref)	(ref)	(ref)	(ref)	(ref)	(ref)	(ref)	(ref)
	Female	1.202	1.042	1.387	1.219	1.065	1.395	1.185	1.043	1.346	1.274	1.122	1.447	0.996	0.872	1.137
Ethnicity	White - British	(ref)	(ref)	(ref)	(ref)	(ref)	(ref)	(ref)	(ref)	(ref)	(ref)	(ref)	(ref)	(ref)	(ref)	(ref)
	Any other White background	1.384	0.777	2.464	1.076	0.638	1.812	1.386	0.939	2.045	1.037	0.664	1.619	0.920	0.576	1.469
	Asian or Asian British	0.435	0.102	1.856	1.376	0.644	2.941	1.053	0.424	2.616	1.450	0.606	3.472	1.066	0.432	2.632
	Mixed/Dual background	1.533	0.854	2.752	1.109	0.625	1.969	0.942	0.522	1.698	0.964	0.564	1.648	0.691	0.352	1.358
	Any other ethnic group	1.905	0.736	4.933	1.369	0.542	3.456	0.765	0.316	1.852	1.176	0.596	2.322	0.544	0.213	1.388
SES	IMD 2010	2.182	1.074	4.435	1.025	0.485	2.163	2.423	1.264	4.645	1.758	0.888	3.480	1.538	0.775	3.052
NCMP participation (non-participation >0.2)		1.083	0.892	1.315	1.092	0.893	1.336	0.904	0.723	1.131	1.246	0.962	1.615	1.285	0.963	1.714
**Factor**		**Odds Ratio**	**LCI**	**UCI**	**Odds Ratio**	**LCI**	**UCI**	**Odds Ratio**	**LCI**	**UCI**	**Odds Ratio**	**LCI**	**UCI**	**Odds Ratio**	**LCI**	**UCI**
Gender	Male	(ref)	(ref)	(ref)	(ref)	(ref)	(ref)	(ref)	(ref)	(ref)	(ref)	(ref)	(ref)	(ref)	(ref)	(ref)
	Female	1.203	1.042	1.387	1.217	1.064	1.393	1.185	1.043	1.346	1.275	1.123	1.448	0.996	0.872	1.138
Ethnicity	White - British	(ref)	(ref)	(ref)	(ref)	(ref)	(ref)	(ref)	(ref)	(ref)	(ref)	(ref)	(ref)	(ref)	(ref)	(ref)
	Any other White background	1.381	0.776	2.459	1.067	0.634	1.797	1.385	0.939	2.044	1.039	0.665	1.622	0.915	0.573	1.461
	Asian or Asian British	0.438	0.103	1.870	1.344	0.630	2.868	1.051	0.423	2.610	1.465	0.612	3.509	1.043	0.422	2.577
	Mixed/Dual background	1.538	0.857	2.762	1.106	0.624	1.961	0.941	0.522	1.697	0.967	0.566	1.654	0.689	0.351	1.354
	Any other ethnic group	1.927	0.744	4.991	1.336	0.530	3.369	0.763	0.315	1.848	1.177	0.596	2.322	0.541	0.212	1.380
SES	IMD 2010	2.269	1.107	4.649	0.958	0.460	1.995	2.401	1.245	4.628	1.796	0.904	3.570	1.464	0.736	2.909
NCMP participation (non-participation >0.2)		1.078	0.888	1.309	1.116	0.916	1.360	0.904	0.723	1.131	1.241	0.958	1.608	1.304	0.977	1.740
Factor 4		0.971	0.904	1.043	1.110	1.030	1.197	1.007	0.939	1.081	0.980	0.915	1.050	1.045	0.972	1.124

**Three-level**		**2006/07**			**2007/08**			**2008/09**			**2009/10**			**2010/11**		
n		9532			10955			11217			11345			10559		
Log likelihood	**Null**	-4160.65			-4774.86			-5407.81			-5287.50			-4912.78		
	**Individual**	-4143.36			-4756.93			-5388.58			-5266.85			-4904.63		
	**Factor**	-4143.34			-4755.39			-5388.58			-5265.70			-4903.84		
	**Interaction**	-4134.55			-4742.40			-5384.19			-5251.66			-4898.16		
School variance	**Null**	8.23E-11	0 .		1.53E-07	0 .		2.04E-10	0 .		0.017	0.001	0.314	0.009	4.04E-05	1.870
	**Individual**	4.56E-17	0 .		7.04E-10	0 .		2.13E-15	0 .		0.014	4.15E-04	0.475	0.014	4.61E-04	0.399
	**Factor**	2.19E-15	0 .		5.61E-10	0 .		6.94E-13	0 .		0.012	2.32E-04	0.650	0.012	2.39E-04	0.574
	**Interaction**	1.11E-21	0 .		0.021	0.002	0.288	4.88E-11	0 .		0.034	0.009	0.132	0.021	0.002	0.182
Year group variai	**Null**	9.30E-03	1.07E-04	0.813	0.115	0.067	0.196	0.095	0.057	0.156	0.048	0.013	0.183	0.047	0.013	0.168
	**Individual**	2.04E-14	0 .		0.109	0.062	0.190	0.093	0.056	0.154	0.050	0.014	0.184	0.038	0.008	0.172
	**Factor**	3.93E-12	0 .		0.103	0.057	0.184	0.093	0.056	0.154	0.050	0.013	0.184	0.038	0.009	0.171
	**Interaction**	1.50E-21	0 .		0.049	0.011	0.212	0.088	0.053	0.149	0.010	3.83E-05	2.566	0.021	0.002	0.277
**Individual**		**Odds Ratio**	**LCI**	**UCI**	**Odds Ratio**	**LCI**	**UCI**	**Odds Ratio**	**LCI**	**UCI**	**Odds Ratio**	**LCI**	**UCI**	**Odds Ratio**	**LCI**	**UCI**
Gender	Male	(ref)	(ref)	(ref)	(ref)	(ref)	(ref)	(ref)	(ref)	(ref)	(ref)	(ref)	(ref)	(ref)	(ref)	(ref)
	Female	1.319	1.181	1.473	1.331	1.200	1.478	1.301	1.181	1.432	1.350	1.225	1.489	1.130	1.021	1.250
Ethnicity	White - British	(ref)	(ref)	(ref)	(ref)	(ref)	(ref)	(ref)	(ref)	(ref)	(ref)	(ref)	(ref)	(ref)	(ref)	(ref)
	Any other White background	1.047	0.705	1.554	0.834	0.577	1.205	1.107	0.819	1.496	1.098	0.799	1.511	1.105	0.817	1.494
	Asian or Asian British	0.738	0.312	1.744	0.769	0.403	1.469	0.874	0.435	1.757	0.965	0.483	1.925	0.743	0.377	1.463
	Mixed/Dual background	1.409	0.886	2.241	0.727	0.446	1.184	1.076	0.736	1.574	0.909	0.622	1.329	0.958	0.634	1.448
	Any other ethnic group	1.348	0.670	2.710	1.000	0.462	2.166	0.961	0.521	1.772	1.084	0.633	1.858	0.517	0.248	1.080
SES	IMD 2010	2.098	1.225	3.592	1.657	0.947	2.902	2.099	1.273	3.461	1.699	1.013	2.849	1.310	0.770	2.229
NCMP participation (non-participation >0.2)		1.071	0.917	1.250	1.067	0.900	1.266	1.096	0.899	1.338	0.991	0.819	1.198	1.370	1.045	1.797
**Factor**		**Odds Ratio**	**LCI**	**UCI**	**Odds Ratio**	**LCI**	**UCI**	**Odds Ratio**	**LCI**	**UCI**	**Odds Ratio**	**LCI**	**UCI**	**Odds Ratio**	**LCI**	**UCI**
Gender	Male	(ref)	(ref)	(ref)	(ref)	(ref)	(ref)	(ref)	(ref)	(ref)	(ref)	(ref)	(ref)	(ref)	(ref)	(ref)
	Female	1.319	1.181	1.473	1.332	1.200	1.478	1.301	1.181	1.432	1.352	1.226	1.490	1.130	1.021	1.250
Ethnicity	White - British	(ref)	(ref)	(ref)	(ref)	(ref)	(ref)	(ref)	(ref)	(ref)	(ref)	(ref)	(ref)	(ref)	(ref)	(ref)
	Any other White background	1.047	0.705	1.555	0.833	0.576	1.203	1.107	0.819	1.496	1.102	0.801	1.515	1.100	0.813	1.487
	Asian or Asian British	0.740	0.313	1.749	0.758	0.397	1.448	0.873	0.434	1.756	0.983	0.493	1.963	0.731	0.371	1.440
	Mixed/Dual background	1.411	0.887	2.244	0.726	0.446	1.183	1.076	0.736	1.574	0.914	0.625	1.336	0.957	0.633	1.445
	Any other ethnic group	1.352	0.672	2.720	0.987	0.456	2.138	0.960	0.520	1.771	1.096	0.639	1.878	0.513	0.246	1.072
SES	IMD 2010	2.115	1.229	3.639	1.571	0.898	2.749	2.096	1.266	3.469	1.774	1.054	2.984	1.261	0.740	2.147
NCMP participation (non-participation >0.2)		1.069	0.915	1.249	1.085	0.914	1.287	1.097	0.899	1.338	0.992	0.821	1.199	1.387	1.057	1.819
Factor 4		0.994	0.941	1.050	1.056	0.994	1.123	1.001	0.947	1.059	0.958	0.907	1.012	1.037	0.980	1.098
**Interaction**		**Odds Ratio**	**LCI**	**UCI**	**Odds Ratio**	**LCI**	**UCI**	**Odds Ratio**	**LCI**	**UCI**	**Odds Ratio**	**LCI**	**UCI**	**Odds Ratio**	**LCI**	**UCI**
Gender	Male	(ref)	(ref)	(ref)	(ref)	(ref)	(ref)	(ref)	(ref)	(ref)	(ref)	(ref)	(ref)	(ref)	(ref)	(ref)
	Female	1.318	1.180	1.472	1.334	1.202	1.480	1.302	1.182	1.433	1.353	1.228	1.492	1.132	1.023	1.252
Ethnicity	White - British	(ref)	(ref)	(ref)	(ref)	(ref)	(ref)	(ref)	(ref)	(ref)	(ref)	(ref)	(ref)	(ref)	(ref)	(ref)
	Any other White background	1.090	0.733	1.620	0.866	0.600	1.249	1.110	0.821	1.500	1.115	0.811	1.532	1.122	0.830	1.518
	Asian or Asian British	0.744	0.314	1.759	0.773	0.405	1.475	0.878	0.437	1.765	1.008	0.505	2.012	0.749	0.381	1.476
	Mixed/Dual background	1.404	0.883	2.234	0.732	0.450	1.192	1.090	0.745	1.593	0.934	0.639	1.364	0.975	0.645	1.474
	Any other ethnic group	1.381	0.686	2.782	1.000	0.462	2.164	0.960	0.520	1.771	1.094	0.639	1.873	0.512	0.245	1.069
SES	IMD 2010	2.194	1.274	3.778	1.634	0.934	2.856	2.102	1.271	3.476	1.791	1.064	3.016	1.280	0.752	2.179
NCMP participation (non-participation >0.2)		1.056	0.903	1.234	1.049	0.889	1.237	1.051	0.860	1.283	1.036	0.861	1.248	1.323	1.011	1.733
Factor 4		1.032	0.946	1.125	0.998	0.911	1.092	0.992	0.913	1.077	0.937	0.863	1.018	1.027	0.945	1.116
Year group	Reception	(ref)	(ref)	(ref)	(ref)	(ref)	(ref)	(ref)	(ref)	(ref)	(ref)	(ref)	(ref)	(ref)	(ref)	(ref)
	Year 6	1.522	1.059	2.186	0.991	0.687	1.431	1.125	0.795	1.591	1.178	0.858	1.618	1.139	0.808	1.606
Year group*factor	Reception	(ref)	(ref)	(ref)	(ref)	(ref)	(ref)	(ref)	(ref)	(ref)	(ref)	(ref)	(ref)	(ref)	(ref)	(ref)
	Year 6	0.941	0.842	1.052	1.107	0.987	1.242	1.019	0.911809	1.138677	1.040	0.939	1.151	1.019	0.915	1.134

Supporting file 3 to Williams, A.J., Wyatt, K.M., Williams, C.A., Logan, S., and Henley, W.E. Exploring the potential of a school impact on pupil weight status: exploratory factor analysis and repeat cross-sectional study of the National Child Measurement Programme.

S3 file - Detailed results and sensitivity analysis

Factor 4 - Prioritisation of physical activity

Red shading indicates significance (p<0.05), Green shading indicates modles which had to be estimated in R

Obese (IOTF)

**Single level**		**2006/07**			**2007/08**			**2008/09**			**2009/10**			**2010/11**		
n		9910			11353			11696			11851			11016		
Log likelihood	**Null**	-1605.40			-1724.55			-1999.60			-2090.78			-1901.75		
	**Individual**	-1595.21			-1703.72			-1989.84			-2072.60			-1869.13		
	**Factor**	-1592.91			-1703.58			-1986.27			-2069.97			-1868.17		
**Individual**		**Odds Ratio**	**LCI**	**UCI**	**Odds Ratio**	**LCI**	**UCI**	**Odds Ratio**	**LCI**	**UCI**	**Odds Ratio**	**LCI**	**UCI**	**Odds Ratio**	**LCI**	**UCI**
Gender	Male	(ref)	(ref)	(ref)	(ref)	(ref)	(ref)	(ref)	(ref)	(ref)	(ref)	(ref)	(ref)	(ref)	(ref)	(ref)
	Female	1.066	0.868	1.310	1.207	0.987	1.475	1.255	1.045	1.508	1.220	1.020	1.459	1.308	1.083	1.580
Ethnicity	White - British	(ref)	(ref)	(ref)	(ref)	(ref)	(ref)	(ref)	(ref)	(ref)	(ref)	(ref)	(ref)	(ref)	(ref)	(ref)
	Any other White background	0.977	0.455	2.094	1.994	1.236	3.219	1.097	0.624	1.929	1.293	0.761	2.195	0.720	0.368	1.411
	Asian or Asian British	1.522	0.470	4.930	1.761	0.759	4.086	0.796	0.193	3.275	1.410	0.507	3.918	2.012	0.911	4.442
	Mixed/Dual background	1.619	0.747	3.504	1.083	0.476	2.466	1.078	0.527	2.205	0.752	0.352	1.609	1.467	0.768	2.804
	Any other ethnic group	1.503	0.465	4.855	2.289	0.819	6.401	2.249	1.027	4.925	1.443	0.627	3.323	1.456	0.586	3.621
SES	IMD 2010	7.403	2.939	18.646	7.276	2.922	18.121	3.863	1.682	8.873	9.268	4.174	20.580	19.715	8.886	43.742
NCMP participation (non-participation >0.2)		0.877	0.644	1.195	0.601	0.421	0.858	0.930	0.651	1.328	0.823	0.587	1.154	0.678	0.369	1.245
**Factor**		**Odds Ratio**	**LCI**	**UCI**	**Odds Ratio**	**LCI**	**UCI**	**Odds Ratio**	**LCI**	**UCI**	**Odds Ratio**	**LCI**	**UCI**	**Odds Ratio**	**LCI**	**UCI**
Gender	Male	(ref)	(ref)	(ref)	(ref)	(ref)	(ref)	(ref)	(ref)	(ref)	(ref)	(ref)	(ref)	(ref)	(ref)	(ref)
	Female	1.066	0.868	1.310	1.207	0.987	1.475	1.254	1.044	1.507	1.216	1.017	1.454	1.308	1.083	1.579
Ethnicity	White - British	(ref)	(ref)	(ref)	(ref)	(ref)	(ref)	(ref)	(ref)	(ref)	(ref)	(ref)	(ref)	(ref)	(ref)	(ref)
	Any other White background	0.989	0.461	2.122	1.991	1.233	3.214	1.098	0.624	1.932	1.279	0.753	2.172	0.724	0.370	1.419
	Asian or Asian British	1.602	0.494	5.197	1.747	0.752	4.055	0.760	0.184	3.130	1.341	0.482	3.729	2.078	0.939	4.597
	Mixed/Dual background	1.660	0.766	3.596	1.082	0.475	2.465	1.067	0.522	2.183	0.742	0.347	1.587	1.474	0.771	2.818
	Any other ethnic group	1.584	0.490	5.125	2.266	0.810	6.340	2.140	0.977	4.690	1.395	0.605	3.213	1.480	0.595	3.681
SES	IMD 2010	8.957	3.463	23.169	6.914	2.732	17.502	3.132	1.358	7.225	7.756	3.468	17.345	22.482	9.877	51.172
NCMP participation (non-participation >0.2)		0.862	0.633	1.174	0.606	0.424	0.867	0.940	0.658	1.342	0.818	0.584	1.147	0.658	0.358	1.211
Factor 4		0.895	0.809	0.989	1.029	0.928	1.141	1.138	1.034	1.252	1.114	1.015	1.223	0.933	0.847	1.029

**Reception two-level**		**2006/07**			**2007/08**			**2008/09**			**2009/10**			**2010/11**		
n		4352			5190			5421			5373			4970		
Log likelihood	**Null**	-612.05			-756.86			-925.76			-854.75			-796.40		
	**Individual**	-605.01			-742.63			-914.05			-848.53			-786.91		
	**Factor**	-604.73			-742.18			-911.66			-847.51			-786.58		
School variance	**Null**	7.88E-12	0	.	0.205	0.056	0.750	0.117	0.024	0.577	0.458	0.255	0.823	0.110	0.010	1.184
	**Individual**	3.72E-16	0	.	0.151			0.126			0.384	0.202	0.732	0.117	0.015	0.929
	**Factor**	1.20E-13	0	.	0.133			0.136			0.353	0.179	0.696	0.113	0.014	0.931
**Individual**		**Odds Ratio**	**LCI**	**UCI**	**Odds Ratio**	**LCI**	**UCI**	**Odds Ratio**	**LCI**	**UCI**	**Odds Ratio**	**LCI**	**UCI**	**Odds Ratio**	**LCI**	**UCI**
Gender	Male	(ref)	(ref)	(ref)	(ref)	(ref)	(ref)	(ref)	(ref)	(ref)	(ref)	(ref)	(ref)	(ref)	(ref)	(ref)
	Female	1.443	1.023	2.034	1.369	1.000	1.876	1.675	1.264	2.221	1.139	0.857	1.515	1.236	0.920	1.660
Ethnicity	White - British	(ref)	(ref)	(ref)	(ref)	(ref)	(ref)	(ref)	(ref)	(ref)	(ref)	(ref)	(ref)	(ref)	(ref)	(ref)
	Any other White background	1.375	0.551	3.428	2.273	1.224	4.222	1.339	0.634	2.826	1.149	0.513	2.574	0.951	0.412	2.197
	Asian or Asian British	3.585	1.053	12.208	1.743	0.511	5.943	3.52E-06	0	.	3.548	1.191	10.567	2.297	0.795	6.635
	Mixed/Dual background	1.999	0.613	6.520	7.40E-07	0.000	.	0.683	0.209	2.232	0.647	0.201	2.085	0.820	0.255	2.631
	Any other ethnic group	1.063	0.143	7.903	6.68E-07	0.000	.	1.423	0.327	6.199	1.260	0.291	5.457	2.223	0.668	7.398
SES	IMD 2010	5.261	1.155	23.970	6.738	1.563	29.048	4.295	1.217	15.162	6.086	1.539	24.074	12.443	3.260	47.488
NCMP participation (non-participation >0.2)		0.861	0.499	1.484	0.558	0.289	1.077	0.726	0.304	1.736	0.782	0.437	1.398	0.822	0.192	3.519
**Factor**		**Odds Ratio**	**LCI**	**UCI**	**Odds Ratio**	**LCI**	**UCI**	**Odds Ratio**	**LCI**	**UCI**	**Odds Ratio**	**LCI**	**UCI**	**Odds Ratio**	**LCI**	**UCI**
Gender	Male	(ref)	(ref)	(ref)	(ref)	(ref)	(ref)	(ref)	(ref)	(ref)	(ref)	(ref)	(ref)	(ref)	(ref)	(ref)
	Female	1.443	1.023	2.034	1.373	1.003	1.879	1.672	1.260	2.218	1.134	0.853	1.508	1.235	0.919	1.659
Ethnicity	White - British	(ref)	(ref)	(ref)	(ref)	(ref)	(ref)	(ref)	(ref)	(ref)	(ref)	(ref)	(ref)	(ref)	(ref)	(ref)
	Any other White background	1.390	0.557	3.470	2.272	1.226	4.210	1.355	0.641	2.862	1.144	0.511	2.561	0.955	0.414	2.206
	Asian or Asian British	3.725	1.089	12.743	1.707	0.502	5.802	3.37E-06	0	.	3.380	1.135	10.070	2.366	0.817	6.854
	Mixed/Dual background	2.047	0.626	6.688	7.41E-07	0	.	0.679	0.207	2.223	0.642	0.199	2.069	0.823	0.257	2.642
	Any other ethnic group	1.108	0.149	8.266	6.47E-07	0	.	1.344	0.308	5.875	1.190	0.275	5.152	2.276	0.683	7.579
SES	IMD 2010	5.818	1.237	27.371	5.771	1.315	25.317	3.336	0.937	11.882	5.335	1.360	20.924	13.895	3.529	54.708
NCMP participation (non-participation >0.2)		0.851	0.493	1.469	0.580	0.300	1.120	0.750	0.311	1.808	0.760	0.425	1.360	0.787	0.184	3.371
Factor 4		0.938	0.792	1.110	1.087	0.912	1.296	1.186	1.013	1.389	1.139	0.953	1.361	0.937	0.800	1.097

**Year 6 two-level**		**2006/07**			**2007/08**			**2008/09**			**2009/10**			**2010/11**		
n		5558			6163			6275			6478			6046		
Log likelihood	**Null**	-987.29			-963.28			-1068.95			-1206.63			-1102.13		
	**Individual**	-979.22			-950.71			-1064.68			-1197.20			-1076.18		
	**Factor**	-977.15			-950.71			-1063.35			-1196.87			-1075.60		
School variance	**Null**	0.123	0.032	0.474	0.213	0.072	0.627	0.253	0.101	0.633	0.151	0.046	0.494	0.119	0.027	0.535
	**Individual**	0.057	0.004	0.758	0.185	0.056	0.616	0.233	0.088	0.618	0.112			0.007	4.94E-11	8.76E+05
	**Factor**	0.040			0.185	0.056	0.617	0.234	0.088	0.625	0.105			0.005	1.34E-13	1.85E+08
**Individual**		**Odds Ratio**	**LCI**	**UCI**	**Odds Ratio**	**LCI**	**UCI**	**Odds Ratio**	**LCI**	**UCI**	**Odds Ratio**	**LCI**	**UCI**	**Odds Ratio**	**LCI**	**UCI**
Gender	Male	(ref)	(ref)	(ref)	(ref)	(ref)	(ref)	(ref)	(ref)	(ref)	(ref)	(ref)	(ref)	(ref)	(ref)	(ref)
	Female	0.891	0.686	1.156	1.089	0.832	1.426	0.996	0.774	1.283	1.675	1.264	2.221	1.367	1.068	1.750
Ethnicity	White - British	(ref)	(ref)	(ref)	(ref)	(ref)	(ref)	(ref)	(ref)	(ref)	(ref)	(ref)	(ref)	(ref)	(ref)	(ref)
	Any other White background	0.684	0.166	2.820	1.636	0.700	3.821	0.877	0.353	2.178	1.339	0.634	2.826	0.501	0.157	1.593
	Asian or Asian British	0.000	0	.	1.715	0.504	5.838	1.807	0.419	7.796	3.52E-06	0	.	1.790	0.531	6.031
	Mixed/Dual background	1.426	0.511	3.974	2.136	0.909	5.020	1.591	0.630	4.020	0.683	0.209	2.232	2.321	1.049	5.133
	Any other ethnic group	2.142	0.494	9.287	4.246	1.426	12.646	3.255	1.235	8.577	1.423	0.327	6.199	0.957	0.229	3.990
SES	IMD 2010	8.900	2.628	30.136	7.916	2.112	29.672	2.819	0.789	10.075	4.295	1.217	15.162	31.501	11.229	88.371
NCMP participation (non-participation >0.2)		0.853	0.579	1.257	0.585	0.364	0.940	0.970	0.620	1.516	0.726	0.304	1.736	0.597	0.303	1.176
**Factor**		**Odds Ratio**	**LCI**	**UCI**	**Odds Ratio**	**LCI**	**UCI**	**Odds Ratio**	**LCI**	**UCI**	**Odds Ratio**	**LCI**	**UCI**	**Odds Ratio**	**LCI**	**UCI**
Gender	Male	(ref)	(ref)	(ref)	(ref)	(ref)	(ref)	(ref)	(ref)	(ref)	(ref)	(ref)	(ref)	(ref)	(ref)	(ref)
	Female	0.891	0.686	1.159	1.090	0.832	1.426	0.997	0.774	1.283	1.322	1.041	1.677	1.367	1.068	1.749
Ethnicity	White - British	(ref)	(ref)	(ref)	(ref)	(ref)	(ref)	(ref)	(ref)	(ref)	(ref)	(ref)	(ref)	(ref)	(ref)	(ref)
	Any other White background	0.681	0.164	2.836	1.637	0.701	3.824	0.872	0.351	2.165	1.345	0.642	2.815	0.505	0.159	1.606
	Asian or Asian British	4.21E-06	0	.	1.719	0.505	5.856	1.742	0.403	7.524	3.12E-06	0	.	1.854	0.550	6.257
	Mixed/Dual background	1.446	0.516	4.053	2.137	0.909	5.023	1.578	0.624	3.989	0.901	0.321	2.525	2.335	1.056	5.165
	Any other ethnic group	2.217	0.509	9.662	4.257	1.428	12.690	3.137	1.190	8.272	1.628	0.572	4.634	0.964	0.231	4.019
SES	IMD 2010	11.445	3.311	39.555	8.003	2.104	30.445	2.400	0.670	8.604	5.872	1.799	19.166	36.138	12.443	104.956
NCMP participation (non-participation >0.2)		0.838	0.570	1.234	0.585	0.364	0.939	0.970	0.619	1.520	1.131	0.697	1.835	0.582	0.295	1.148
Factor 4		0.872	0.764	0.995	0.992	0.857	1.148	1.130	0.974	1.312	1.053	0.927	1.196	0.931	0.819	1.059

**Three-level**		**2006/07**			**2007/08**			**2008/09**			**2009/10**			**2010/11**		
n		9910			11353			11696			11851			11016		
Log likelihood	**Null**	-1602.53			-1719.64			-1992.75			-2063.11			-1898.27		
	**Individual**	-1594.02			-1700.41			-1984.67			-2053.82			-1868.08		
	**Factor**	-1592.20			-1700.35			-1980.96			-2052.85			-1867.21		
	**Interaction**	-1587.25			-1699.13			-1980.80			-2049.83			-1864.86		
School variance	**Null**	0.122	0.034	0.435	0.108	0.023	0.508	0.164	0.064	0.419	0.124			0.140	0.047	0.418
	**Individual**	0.074	0.010	0.551	0.098	0.020	0.484	0.135	0.045	0.411	0.081	0.013	0.499	0.057	0.006	0.533
	**Factor**	0.052	0.003	0.824	0.095	0.018	0.494	0.139	0.047	0.413	0.076	0.011	0.517	0.052	0.005	0.589
	**Interaction**	0.049	0.005	0.482	0.102	0.023	0.461	0.139	0.046	0.414	0.085	0.016	0.448	0.054	0.009	0.310
Year group variai	**Null**	0.007	1.29E-11	3.64E+06	0.097	0.010	0.914	0.035	4.31E-04	2.823	0.206			0.002	3.05E-43	1.03E+37
	**Individual**	0.012	1.75E-07	883.061	0.063	0.003	1.488	0.041	9.27E-04	1.801	0.173	0.059	0.506	0.007	2.17E-13	2.01E+08
	**Factor**	0.016	2.25E-06	110.738	0.064	0.003	1.462	0.038	6.05E-04	2.357	0.166	0.055	0.504	0.009	1.38E-10	5.77E+05
	**Interaction**	5.72E-11	0	.	0.046	0.001	3.217	0.040	7.89E-04	2.029	0.149	0.047	0.472	7.95E-10	0	.
**Individual**		**Odds Ratio**	**LCI**	**UCI**	**Odds Ratio**	**LCI**	**UCI**	**Odds Ratio**	**LCI**	**UCI**	**Odds Ratio**	**LCI**	**UCI**	**Odds Ratio**	**LCI**	**UCI**
Gender	Male	(ref)	(ref)	(ref)	(ref)	(ref)	(ref)	(ref)	(ref)	(ref)	(ref)	(ref)	(ref)	(ref)	(ref)	(ref)
	Female	1.064	0.866	1.308	1.200	0.981	1.469	1.262	1.049	1.518	1.234	1.030	1.479	1.310	1.084	1.583
Ethnicity	White - British	(ref)	(ref)	(ref)	(ref)	(ref)	(ref)	(ref)	(ref)	(ref)	(ref)	(ref)	(ref)	(ref)	(ref)	(ref)
	Any other White background	0.979	0.455	2.108	2.011	1.229	3.290	1.120	0.633	1.982	1.255	0.730	2.156	0.730	0.372	1.432
	Asian or Asian British	1.550	0.473	5.075	1.689	0.713	4.002	0.796	0.191	3.313	1.560	0.554	4.393	2.056	0.926	4.566
	Mixed/Dual background	1.654	0.760	3.597	1.113	0.486	2.546	1.093	0.532	2.249	0.779	0.362	1.677	1.453	0.758	2.784
	Any other ethnic group	1.537	0.470	5.023	2.280	0.801	6.488	2.341	1.054	5.198	1.518	0.650	3.547	1.483	0.593	3.705
SES	IMD 2010	6.827	2.568	18.149	7.068	2.639	18.934	3.241	1.292	8.127	5.636	2.250	14.115	20.503	8.866	47.418
NCMP participation (non-participation >0.2)		0.885	0.642	1.219	0.574	0.394	0.837	0.932	0.637	1.364	0.902	0.621	1.310	0.679	0.366	1.262
**Factor**		**Odds Ratio**	**LCI**	**UCI**	**Odds Ratio**	**LCI**	**UCI**	**Odds Ratio**	**LCI**	**UCI**	**Odds Ratio**	**LCI**	**UCI**	**Odds Ratio**	**LCI**	**UCI**
Gender	Male	(ref)	(ref)	(ref)	(ref)	(ref)	(ref)	(ref)	(ref)	(ref)	(ref)	(ref)	(ref)	(ref)	(ref)	(ref)
	Female	1.065	0.866	1.309	1.200	0.981	1.469	1.261	1.048	1.517	1.231	1.027	1.476	1.310	1.084	1.583
Ethnicity	White - British	(ref)	(ref)	(ref)	(ref)	(ref)	(ref)	(ref)	(ref)	(ref)	(ref)	(ref)	(ref)	(ref)	(ref)	(ref)
	Any other White background	0.991	0.461	2.132	2.008	1.228	3.286	1.121	0.633	1.984	1.246	0.725	2.142	0.733	0.374	1.440
	Asian or Asian British	1.620	0.496	5.298	1.680	0.709	3.984	0.762	0.183	3.175	1.512	0.537	4.260	2.118	0.952	4.710
	Mixed/Dual background	1.682	0.773	3.657	1.112	0.486	2.544	1.085	0.528	2.233	0.772	0.359	1.663	1.461	0.762	2.801
	Any other ethnic group	1.606	0.492	5.239	2.266	0.796	6.452	2.243	1.009	4.984	1.487	0.636	3.475	1.504	0.602	3.758
SES	IMD 2010	8.175	3.019	22.141	6.858	2.531	18.578	2.665	1.062	6.687	5.168	2.065	12.933	23.169	9.799	54.782
NCMP participation (non-participation >0.2)		0.870	0.632	1.196	0.578	0.396	0.844	0.940	0.642	1.379	0.900	0.620	1.307	0.662	0.356	1.230
Factor 4		0.899	0.807	1.001	1.021	0.911	1.145	1.167	1.042	1.308	1.080	0.969	1.204	0.933	0.843	1.034
**Interaction**		**Odds Ratio**	**LCI**	**UCI**	**Odds Ratio**	**LCI**	**UCI**	**Odds Ratio**	**LCI**	**UCI**	**Odds Ratio**	**LCI**	**UCI**	**Odds Ratio**	**LCI**	**UCI**
Gender	Male	(ref)	(ref)	(ref)	(ref)	(ref)	(ref)	(ref)	(ref)	(ref)	(ref)	(ref)	(ref)	(ref)	(ref)	(ref)
	Female	1.064	0.866	1.308	1.204	0.984	1.473	1.261	1.048	1.517	1.232	1.028	1.477	1.313	1.086	1.586
Ethnicity	White - British	(ref)	(ref)	(ref)	(ref)	(ref)	(ref)	(ref)	(ref)	(ref)	(ref)	(ref)	(ref)	(ref)	(ref)	(ref)
	Any other White background	1.054	0.489	2.269	2.064	1.261	3.380	1.123	0.635	1.988	1.264	0.736	2.172	0.751	0.382	1.475
	Asian or Asian British	1.656	0.507	5.406	1.692	0.714	4.010	0.762	0.183	3.174	1.538	0.546	4.335	2.172	0.976	4.832
	Mixed/Dual background	1.667	0.767	3.625	1.117	0.488	2.557	1.087	0.528	2.238	0.789	0.366	1.700	1.500	0.782	2.876
	Any other ethnic group	1.645	0.505	5.364	2.255	0.792	6.416	2.243	1.009	4.987	1.472	0.630	3.437	1.504	0.602	3.759
SES	IMD 2010	8.750	3.246	23.587	6.794	2.508	18.403	2.659	1.059	6.673	5.185	2.071	12.985	23.475	9.947	55.398
NCMP participation (non-participation >0.2)		0.852	0.620	1.169	0.571	0.391	0.833	0.935	0.634	1.378	0.916	0.630	1.331	0.622	0.334	1.159
Factor 4		0.939	0.793	1.113	1.073	0.904	1.272	1.203	1.026	1.410	1.153	0.974	1.365	0.925	0.796	1.076
Year group	Reception	(ref)	(ref)	(ref)	(ref)	(ref)	(ref)	(ref)	(ref)	(ref)	(ref)	(ref)	(ref)	(ref)	(ref)	(ref)
	Year 6	1.724	0.886	3.354	1.489	0.742	2.985	1.211	0.626	2.341	1.728	0.884	3.378	1.174	0.635	2.170
Year group*factor	Reception	(ref)	(ref)	(ref)	(ref)	(ref)	(ref)	(ref)	(ref)	(ref)	(ref)	(ref)	(ref)	(ref)	(ref)	(ref)
	Year 6	0.933	0.758	1.149	0.923	0.747	1.141	0.947	0.776	1.157	0.904	0.734	1.112	1.017	0.840	1.232

Supporting file 3 to Williams, A.J., Wyatt, K.M., Williams, C.A., Logan, S., and Henley, W.E. Exploring the potential of a school impact on pupil weight status: exploratory factor analysis and repeat cross-sectional study of the National Child Measurement Programme.

S3 file - Detailed results and sensitivity analysis

Factor 4 - Prioritisation of physical activity

Red shading indicates significance (p<0.05), Green shading indicates modles which had to be estimated in R

BMI-SDS

**Single level**		**2006/07**			**2007/08**			**2008/09**			**2009/10**			**2010/11**		
n		9910			11353			11696			11851			11016		
Log likelihood	**Null**	-14914.99			-16983.74			-17154.20			-17558.83			-16084.62		
	**Individual**	-14907.46			-16971.41			-17128.16			-17540.88			-16059.60		
	**Factor**	-14909.39			-16974.22			-17131.33			-17544.36			-16063.21		
Residual varianc	**Null**	1.187	1.155	1.221	1.166	1.136	1.197	1.100	1.072	1.128	1.133	1.104	1.162	1.085	1.057	1.114
	**Individual**	1.183	1.151	1.217	1.162	1.132	1.192	1.093	1.065	1.121	1.128	1.099	1.157	1.079	1.050	1.107
	**Factor**	1.183	1.151	1.217	1.161	1.132	1.192	1.093	1.065	1.121	1.128	1.099	1.157	1.079	1.051	1.108
**Null**		**Mean difference**	**LCI**	**UCI**	**Mean difference**	**LCI**	**UCI**	**Mean difference**	**LCI**	**UCI**	**Mean difference**	**LCI**	**UCI**	**Mean difference**	**LCI**	**UCI**
Constant		0.340	0.319	0.362	0.327	0.307	0.347	0.463	0.444	0.482	0.434	0.415	0.453	0.457	0.438	0.477
**Individual**		**Mean difference**	**LCI**	**UCI**	**Mean difference**	**LCI**	**UCI**	**Mean difference**	**LCI**	**UCI**	**Mean difference**	**LCI**	**UCI**	**Mean difference**	**LCI**	**UCI**
Constant		0.289	0.236	0.342	0.322	0.273	0.370	0.434	0.388	0.480	0.395	0.348	0.441	0.440	0.393	0.486
Gender	Male	(ref)	(ref)	(ref)	(ref)	(ref)	(ref)	(ref)	(ref)	(ref)	(ref)	(ref)	(ref)	(ref)	(ref)	(ref)
	Female	-0.085	-0.127	-0.042	-0.123	-0.162	-0.083	-0.136	-0.173	-0.098	-0.112	-0.150	-0.073	-0.143	-0.182	-0.104
Ethnicity	White - British	(ref)	(ref)	(ref)	(ref)	(ref)	(ref)	(ref)	(ref)	(ref)	(ref)	(ref)	(ref)	(ref)	(ref)	(ref)
	Any other White background	0.040	-0.115	0.194	-0.035	-0.163	0.092	0.083	-0.038	0.205	0.009	-0.118	0.135	0.031	-0.089	0.150
	Asian or Asian British	-0.164	-0.466	0.139	-0.183	-0.409	0.042	-0.205	-0.475	0.064	-0.096	-0.361	0.169	-0.035	-0.268	0.198
	Mixed/Dual background	0.204	0.005	0.402	-0.032	-0.198	0.135	0.021	-0.132	0.174	-0.135	-0.279	0.009	-0.058	-0.213	0.097
	Any other ethnic group	0.034	-0.262	0.331	0.007	-0.287	0.300	0.037	-0.194	0.268	0.156	-0.058	0.371	-0.145	-0.366	0.077
SES	IMD 2010	0.462	0.248	0.676	0.343	0.141	0.545	0.464	0.278	0.650	0.476	0.283	0.668	0.440	0.247	0.633
NCMP participation (non-participation >0.2)		-0.007	-0.068	0.054	-0.007	-0.065	0.051	0.027	-0.044	0.098	0.005	-0.063	0.073	0.038	-0.068	0.144
**Factor**		**Mean difference**	**LCI**	**UCI**	**Mean difference**	**LCI**	**UCI**	**Mean difference**	**LCI**	**UCI**	**Mean difference**	**LCI**	**UCI**	**Mean difference**	**LCI**	**UCI**
Constant		0.346	0.265	0.426	0.284	0.209	0.358	0.406	0.336	0.475	0.413	0.342	0.483	0.430	0.357	0.502
Gender	Male	(ref)	(ref)	(ref)	(ref)	(ref)	(ref)	(ref)	(ref)	(ref)	(ref)	(ref)	(ref)	(ref)	(ref)	(ref)
	Female	-0.084	-0.127	-0.042	-0.123	-0.162	-0.083	-0.136	-0.174	-0.098	-0.112	-0.150	-0.073	-0.143	-0.182	-0.104
Ethnicity	White - British	(ref)	(ref)	(ref)	(ref)	(ref)	(ref)	(ref)	(ref)	(ref)	(ref)	(ref)	(ref)	(ref)	(ref)	(ref)
	Any other White background	0.042	-0.113	0.196	-0.036	-0.163	0.092	0.083	-0.038	0.205	0.009	-0.117	0.136	0.030	-0.089	0.149
	Asian or Asian British	-0.155	-0.457	0.148	-0.188	-0.413	0.037	-0.209	-0.479	0.061	-0.092	-0.358	0.173	-0.037	-0.270	0.197
	Mixed/Dual background	0.208	0.009	0.406	-0.032	-0.198	0.134	0.020	-0.133	0.173	-0.134	-0.278	0.010	-0.058	-0.213	0.097
	Any other ethnic group	0.043	-0.253	0.340	0.002	-0.292	0.296	0.033	-0.199	0.264	0.159	-0.056	0.373	-0.145	-0.367	0.076
SES	IMD 2010	0.486	0.271	0.702	0.325	0.122	0.529	0.450	0.262	0.638	0.484	0.290	0.678	0.435	0.240	0.630
NCMP participation (non-participation >0.2)		-0.011	-0.072	0.051	-0.003	-0.061	0.056	0.028	-0.043	0.099	0.005	-0.063	0.073	0.040	-0.067	0.146
Factor 4		-0.020	-0.041	0.001	0.013	-0.007	0.034	0.010	-0.009	0.029	-0.007	-0.026	0.013	0.004	-0.016	0.023

**Reception two-level**		**2006/07**			**2007/08**			**2008/09**			**2009/10**			**2010/11**		
n		4352			5190			5421			5373			4970		
Log likelihood	**Null**	-6239.98			-7379.80			-7429.67			-7484.45			-6734.36		
	**Individual**	-6245.04			-7371.92			-7428.49			-7486.78			-6735.96		
	**Factor**	-6247.99			-7374.83			-7431.44			-7489.49			-6739.04		
School variance	**Null**	0.041	0.026	0.065	0.042	0.028	0.063	0.025	0.016	0.039	0.040	0.028	0.059	0.014	0.006	0.030
	**Individual**	0.040	0.025	0.064	0.040	0.026	0.062	0.024	0.015	0.038	0.038	0.026	0.057	0.014	0.006	0.029
	**Factor**	0.041	0.026	0.065	0.041	0.027	0.062	0.024	0.015	0.038	0.039	0.026	0.057	0.014	0.007	0.029
Residual varianc	**Null**	1.000	0.958	1.045	0.977	0.939	1.016	0.888	0.855	0.923	0.922	0.887	0.958	0.867	0.833	0.903
	**Individual**	1.001	0.958	1.045	0.972	0.934	1.011	0.886	0.853	0.921	0.921	0.886	0.957	0.866	0.832	0.901
	**Factor**	1.001	0.958	1.045	0.972	0.934	1.011	0.886	0.853	0.921	0.921	0.886	0.957	0.866	0.832	0.901
**Null**		**Mean difference**	**LCI**	**UCI**	**Mean difference**	**LCI**	**UCI**	**Mean difference**	**LCI**	**UCI**	**Mean difference**	**LCI**	**UCI**	**Mean difference**	**LCI**	**UCI**
Constant		0.292	0.251	0.334	0.293	0.255	0.332	0.466	0.433	0.500	0.411	0.373	0.448	0.460	0.429	0.491
**Individual**		**Mean difference**	**LCI**	**UCI**	**Mean difference**	**LCI**	**UCI**	**Mean difference**	**LCI**	**UCI**	**Mean difference**	**LCI**	**UCI**	**Mean difference**	**LCI**	**UCI**
Constant		0.297	0.212	0.381	0.261	0.185	0.338	0.408	0.340	0.477	0.404	0.332	0.477	0.433	0.365	0.501
Gender	Male	(ref)	(ref)	(ref)	(ref)	(ref)	(ref)	(ref)	(ref)	(ref)	(ref)	(ref)	(ref)	(ref)	(ref)	(ref)
	Female	-0.041	-0.101	0.019	-0.087	-0.141	-0.032	-0.054	-0.104	-0.003	-0.063	-0.115	-0.011	-0.071	-0.124	-0.019
Ethnicity	White - British	(ref)	(ref)	(ref)	(ref)	(ref)	(ref)	(ref)	(ref)	(ref)	(ref)	(ref)	(ref)	(ref)	(ref)	(ref)
	Any other White background	-0.049	-0.234	0.137	-0.078	-0.232	0.077	-0.008	-0.166	0.150	-0.024	-0.187	0.140	0.057	-0.086	0.200
	Asian or Asian British	-0.060	-0.454	0.334	-0.550	-0.839	-0.261	-0.511	-0.872	-0.151	-0.166	-0.489	0.157	-0.025	-0.304	0.255
	Mixed/Dual background	0.157	-0.125	0.438	-0.130	-0.350	0.090	-0.029	-0.214	0.157	-0.227	-0.404	-0.050	-0.151	-0.339	0.036
	Any other ethnic group	-0.062	-0.437	0.312	-0.255	-0.674	0.164	-0.003	-0.313	0.306	-0.183	-0.483	0.118	-0.048	-0.344	0.248
SES	IMD 2010	0.174	-0.156	0.504	0.480	0.173	0.788	0.430	0.156	0.704	0.232	-0.057	0.520	0.313	0.037	0.589
NCMP participation (non-participation >0.2)		-0.113	-0.227	0.000	-0.071	-0.185	0.043	0.083	-0.074	0.241	-0.008	-0.121	0.104	0.140	-0.107	0.386
**Factor**		**Mean difference**	**LCI**	**UCI**	**Mean difference**	**LCI**	**UCI**	**Mean difference**	**LCI**	**UCI**	**Mean difference**	**LCI**	**UCI**	**Mean difference**	**LCI**	**UCI**
Constant		0.309	0.167	0.450	0.287	0.156	0.418	0.378	0.266	0.490	0.447	0.324	0.570	0.409	0.300	0.518
Gender	Male	(ref)	(ref)	(ref)	(ref)	(ref)	(ref)	(ref)	(ref)	(ref)	(ref)	(ref)	(ref)	(ref)	(ref)	(ref)
	Female	-0.041	-0.101	0.019	-0.087	-0.141	-0.032	-0.054	-0.104	-0.003	-0.063	-0.115	-0.011	-0.071	-0.124	-0.019
Ethnicity	White - British	(ref)	(ref)	(ref)	(ref)	(ref)	(ref)	(ref)	(ref)	(ref)	(ref)	(ref)	(ref)	(ref)	(ref)	(ref)
	Any other White background	-0.048	-0.234	0.137	-0.077	-0.232	0.078	-0.008	-0.166	0.150	-0.023	-0.186	0.141	0.056	-0.087	0.199
	Asian or Asian British	-0.059	-0.453	0.335	-0.548	-0.838	-0.259	-0.514	-0.875	-0.154	-0.161	-0.485	0.162	-0.029	-0.308	0.251
	Mixed/Dual background	0.157	-0.125	0.439	-0.130	-0.350	0.090	-0.029	-0.215	0.156	-0.226	-0.403	-0.049	-0.151	-0.339	0.036
	Any other ethnic group	-0.061	-0.436	0.314	-0.254	-0.673	0.165	-0.006	-0.316	0.303	-0.177	-0.478	0.124	-0.051	-0.347	0.245
SES	IMD 2010	0.176	-0.155	0.507	0.488	0.179	0.797	0.420	0.144	0.696	0.240	-0.049	0.530	0.303	0.025	0.582
NCMP participation (non-participation >0.2)		-0.114	-0.228	-0.001	-0.075	-0.190	0.041	0.086	-0.072	0.245	-0.006	-0.119	0.108	0.146	-0.101	0.394
Factor 4		-0.004	-0.044	0.036	-0.009	-0.047	0.029	0.011	-0.021	0.044	-0.016	-0.052	0.021	0.009	-0.022	0.039

**Year 6 two-level**		**2006/07**			**2007/08**			**2008/09**			**2009/10**			**2010/11**		
n		5558			6163			6275			6478			6046		
Log likelihood	**Null**	-8607.29			-9526.30			-9610.21			-9959.39			-9263.90		
	**Individual**	-8598.02			-9519.79			-9588.28			-9944.15			-9240.02		
	**Factor**	-8599.62			-9522.04			-9591.35			-9947.25			-9243.31		
School variance	**Null**	0.021	0.011	0.041	0.018	0.009	0.036	0.031	0.019	0.051	0.023	0.013	0.041	0.003	2.01E-04	0.045
	**Individual**	0.019	0.009	0.039	0.018	0.009	0.036	0.029	0.018	0.048	0.022	0.012	0.040	9.06E-04	1.73E-07	4.743
	**Factor**	0.018	0.008	0.038	0.017	0.009	0.035	0.029	0.018	0.048	0.023	0.013	0.040	1.08E-03	7.77E-07	1.509
Residual varianc	**Null**	1.278	1.230	1.327	1.272	1.227	1.319	1.228	1.185	1.273	1.248	1.205	1.293	1.250	1.206	1.296
	**Individual**	1.273	1.225	1.322	1.267	1.222	1.314	1.219	1.176	1.263	1.240	1.198	1.285	1.240	1.196	1.286
	**Factor**	1.273	1.226	1.322	1.268	1.223	1.314	1.219	1.176	1.263	1.240	1.198	1.285	1.240	1.196	1.286
**Null**		**Mean difference**	**LCI**	**UCI**	**Mean difference**	**LCI**	**UCI**	**Mean difference**	**LCI**	**UCI**	**Mean difference**	**LCI**	**UCI**	**Mean difference**	**LCI**	**UCI**
Constant		0.385	0.348	0.422	0.349	0.315	0.383	0.456	0.420	0.493	0.469	0.435	0.503	0.457	0.428	0.487
**Individual**		**Mean difference**	**LCI**	**UCI**	**Mean difference**	**LCI**	**UCI**	**Mean difference**	**LCI**	**UCI**	**Mean difference**	**LCI**	**UCI**	**Mean difference**	**LCI**	**UCI**
Constant		0.320	0.240	0.400	0.379	0.303	0.456	0.460	0.385	0.536	0.423	0.348	0.497	0.442	0.374	0.510
Gender	Male	(ref)	(ref)	(ref)	(ref)	(ref)	(ref)	(ref)	(ref)	(ref)	(ref)	(ref)	(ref)	(ref)	(ref)	(ref)
	Female	-0.123	-0.183	-0.064	-0.155	-0.212	-0.098	-0.202	-0.257	-0.147	-0.145	-0.199	-0.090	-0.202	-0.259	-0.146
Ethnicity	White - British	(ref)	(ref)	(ref)	(ref)	(ref)	(ref)	(ref)	(ref)	(ref)	(ref)	(ref)	(ref)	(ref)	(ref)	(ref)
	Any other White background	0.184	-0.080	0.448	0.067	-0.154	0.287	0.168	-0.015	0.352	0.060	-0.131	0.250	-0.013	-0.210	0.184
	Asian or Asian British	-0.295	-0.751	0.160	0.166	-0.179	0.511	0.011	-0.382	0.404	0.031	-0.393	0.455	-0.100	-0.482	0.281
	Mixed/Dual background	0.236	-0.037	0.509	0.068	-0.176	0.312	0.053	-0.192	0.299	0.020	-0.208	0.247	0.069	-0.180	0.318
	Any other ethnic group	0.154	-0.312	0.620	0.175	-0.231	0.581	0.056	-0.282	0.394	0.463	0.162	0.763	-0.221	-0.544	0.102
SES	IMD 2010	0.595	0.268	0.922	0.192	-0.125	0.510	0.451	0.148	0.754	0.523	0.214	0.831	0.574	0.291	0.857
NCMP participation (non-participation >0.2)		0.034	-0.062	0.130	0.010	-0.079	0.100	0.005	-0.102	0.111	0.104	-0.021	0.228	0.018	-0.109	0.144
**Factor**		**Mean difference**	**LCI**	**UCI**	**Mean difference**	**LCI**	**UCI**	**Mean difference**	**LCI**	**UCI**	**Mean difference**	**LCI**	**UCI**	**Mean difference**	**LCI**	**UCI**
Constant		0.408	0.281	0.536	0.315	0.195	0.435	0.447	0.324	0.569	0.439	0.322	0.556	0.445	0.338	0.551
Gender	Male	(ref)	(ref)	(ref)	(ref)	(ref)	(ref)	(ref)	(ref)	(ref)	(ref)	(ref)	(ref)	(ref)	(ref)	(ref)
	Female	-0.123	-0.183	-0.064	-0.155	-0.212	-0.099	-0.202	-0.257	-0.147	-0.145	-0.199	-0.090	-0.202	-0.259	-0.146
Ethnicity	White - British	(ref)	(ref)	(ref)	(ref)	(ref)	(ref)	(ref)	(ref)	(ref)	(ref)	(ref)	(ref)	(ref)	(ref)	(ref)
	Any other White background	0.183	-0.081	0.447	0.065	-0.155	0.286	0.168	-0.016	0.351	0.061	-0.130	0.251	-0.013	-0.209	0.184
	Asian or Asian British	-0.289	-0.744	0.167	0.160	-0.184	0.505	0.010	-0.383	0.403	0.034	-0.391	0.458	-0.100	-0.482	0.282
	Mixed/Dual background	0.239	-0.035	0.512	0.067	-0.177	0.311	0.053	-0.192	0.299	0.021	-0.207	0.248	0.069	-0.180	0.318
	Any other ethnic group	0.162	-0.304	0.628	0.170	-0.236	0.576	0.055	-0.284	0.393	0.463	0.162	0.764	-0.221	-0.544	0.102
SES	IMD 2010	0.623	0.296	0.951	0.176	-0.142	0.494	0.447	0.142	0.752	0.528	0.218	0.837	0.574	0.289	0.860
NCMP participation (non-participation >0.2)		0.030	-0.065	0.126	0.014	-0.075	0.103	0.005	-0.102	0.111	0.103	-0.022	0.227	0.017	-0.110	0.144
Factor 4		-0.031	-0.066	0.004	0.023	-0.010	0.056	0.005	-0.030	0.040	-0.006	-0.039	0.027	-0.001	-0.030	0.029

**Three-level**		**2006/07**			**2007/08**			**2008/09**			**2009/10**			**2010/11**		
n		9910			11353			11696			11851			11016		
Log likelihood	**Null**	-14878.53			-16951.41			-17108.34			-17502.07			-16081.32		
	**Individual**	-14874.00			-16939.27			-17086.60			-17489.98			-16057.03		
	**Factor**	-14876.54			-16942.49			-17089.85			-17493.03			-16060.58		
	**Interaction**	-14873.54			-16945.09			-17095.32			-17494.80			-16066.40		
School variance	**Null**	0.021	0.012	0.038	0.008	0.002	0.029	0.013	0.006	0.027	0.019	0.010	0.034	0.003	3.15E-04	0.028
	**Individual**	0.020	0.011	0.037	0.007	0.002	0.030	0.011	0.005	0.026	0.017	0.009	0.032	0.002	6.69E-05	0.054
	**Factor**	0.020	0.011	0.037	0.007	0.002	0.030	0.011	0.005	0.026	0.018	0.010	0.033	0.002	7.66E-05	0.050
	**Interaction**	0.022	0.013	0.038	0.008	0.002	0.029	0.011	0.004	0.026	0.018	0.010	0.033	0.002	4.89E-05	0.065
Year group variai	**Null**	0.009	0.003	0.034	0.021	0.011	0.038	0.016	0.008	0.030	0.014	0.007	0.030	0.004	5.01E-04	0.031
	**Individual**	0.009	0.002	0.034	0.021	0.012	0.039	0.016	0.008	0.031	0.014	0.007	0.030	0.004	5.71E-04	0.030
	**Factor**	0.009	0.002	0.034	0.021	0.012	0.039	0.016	0.008	0.031	0.014	0.007	0.030	0.004	5.77E-04	0.030
	**Interaction**	0.004	2.91E-04	0.060	0.020	0.010	0.037	0.016	0.008	0.031	0.013	0.006	0.028	0.005	7.20E-04	0.029
Residual varianc	**Null**	1.157	1.125	1.191	1.138	1.108	1.169	1.071	1.043	1.099	1.100	1.072	1.129	1.078	1.050	1.108
	**Individual**	1.155	1.122	1.188	1.134	1.104	1.164	1.066	1.038	1.094	1.097	1.068	1.125	1.073	1.044	1.102
	**Factor**	1.155	1.122	1.188	1.134	1.104	1.164	1.066	1.038	1.094	1.096	1.068	1.125	1.073	1.044	1.102
	**Interaction**	1.155	1.123	1.189	1.134	1.104	1.164	1.066	1.038	1.094	1.096	1.068	1.125	1.073	1.044	1.102
**Null**		**Mean difference**	**LCI**	**UCI**	**Mean difference**	**LCI**	**UCI**	**Mean difference**	**LCI**	**UCI**	**Mean difference**	**LCI**	**UCI**	**Mean difference**	**LCI**	**UCI**
Constant		0.345	0.314	0.376	0.322	0.295	0.349	0.461	0.434	0.488	0.444	0.415	0.472	0.459	0.437	0.480
**Individual**		**Mean difference**	**LCI**	**UCI**	**Mean difference**	**LCI**	**UCI**	**Mean difference**	**LCI**	**UCI**	**Mean difference**	**LCI**	**UCI**	**Mean difference**	**LCI**	**UCI**
Constant		0.318	0.257	0.379	0.324	0.268	0.379	0.439	0.387	0.492	0.421	0.366	0.475	0.442	0.393	0.491
Gender	Male	(ref)	(ref)	(ref)	(ref)	(ref)	(ref)	(ref)	(ref)	(ref)	(ref)	(ref)	(ref)	(ref)	(ref)	(ref)
	Female	-0.089	-0.132	-0.047	-0.124	-0.163	-0.084	-0.133	-0.171	-0.095	-0.109	-0.148	-0.071	-0.144	-0.183	-0.105
Ethnicity	White - British	(ref)	(ref)	(ref)	(ref)	(ref)	(ref)	(ref)	(ref)	(ref)	(ref)	(ref)	(ref)	(ref)	(ref)	(ref)
	Any other White background	0.028	-0.127	0.183	-0.030	-0.160	0.099	0.081	-0.041	0.203	0.027	-0.100	0.153	0.029	-0.091	0.149
	Asian or Asian British	-0.178	-0.481	0.125	-0.215	-0.440	0.011	-0.222	-0.491	0.047	-0.079	-0.343	0.186	-0.041	-0.274	0.192
	Mixed/Dual background	0.199	0.001	0.396	-0.032	-0.198	0.133	0.011	-0.141	0.163	-0.114	-0.257	0.029	-0.057	-0.212	0.098
	Any other ethnic group	0.030	-0.268	0.328	-0.017	-0.310	0.276	0.023	-0.208	0.255	0.197	-0.018	0.411	-0.141	-0.363	0.081
SES	IMD 2010	0.371	0.130	0.612	0.324	0.098	0.549	0.432	0.222	0.643	0.375	0.156	0.594	0.436	0.234	0.638
NCMP participation (non-participation >0.2)		-0.023	-0.096	0.050	-0.017	-0.087	0.054	0.011	-0.073	0.096	0.027	-0.056	0.109	0.042	-0.068	0.153
**Factor**		**Mean difference**	**LCI**	**UCI**	**Mean difference**	**LCI**	**UCI**	**Mean difference**	**LCI**	**UCI**	**Mean difference**	**LCI**	**UCI**	**Mean difference**	**LCI**	**UCI**
Constant		0.370	0.267	0.473	0.302	0.210	0.393	0.419	0.331	0.507	0.450	0.358	0.542	0.433	0.356	0.511
Gender	Male	(ref)	(ref)	(ref)	(ref)	(ref)	(ref)	(ref)	(ref)	(ref)	(ref)	(ref)	(ref)	(ref)	(ref)	(ref)
	Female	-0.089	-0.132	-0.047	-0.124	-0.163	-0.084	-0.133	-0.171	-0.095	-0.109	-0.147	-0.071	-0.144	-0.183	-0.105
Ethnicity	White - British	(ref)	(ref)	(ref)	(ref)	(ref)	(ref)	(ref)	(ref)	(ref)	(ref)	(ref)	(ref)	(ref)	(ref)	(ref)
	Any other White background	0.029	-0.126	0.185	-0.031	-0.160	0.099	0.081	-0.041	0.203	0.027	-0.099	0.154	0.029	-0.091	0.148
	Asian or Asian British	-0.174	-0.477	0.129	-0.216	-0.442	0.009	-0.224	-0.493	0.045	-0.075	-0.340	0.189	-0.043	-0.276	0.191
	Mixed/Dual background	0.201	0.003	0.398	-0.032	-0.198	0.133	0.011	-0.141	0.163	-0.113	-0.256	0.030	-0.057	-0.212	0.097
	Any other ethnic group	0.035	-0.264	0.333	-0.018	-0.311	0.275	0.021	-0.210	0.253	0.199	-0.016	0.413	-0.142	-0.364	0.080
SES	IMD 2010	0.383	0.141	0.625	0.318	0.092	0.544	0.427	0.215	0.638	0.382	0.162	0.601	0.432	0.229	0.635
NCMP participation (non-participation >0.2)		-0.025	-0.098	0.048	-0.015	-0.086	0.056	0.012	-0.073	0.097	0.027	-0.056	0.109	0.044	-0.067	0.155
Factor 4		-0.018	-0.048	0.011	0.008	-0.018	0.034	0.008	-0.018	0.033	-0.011	-0.038	0.016	0.003	-0.018	0.025
**Interaction**		**Mean difference**	**LCI**	**UCI**	**Mean difference**	**LCI**	**UCI**	**Mean difference**	**LCI**	**UCI**	**Mean difference**	**LCI**	**UCI**	**Mean difference**	**LCI**	**UCI**
Constant		0.262	0.132	0.392	0.313	0.191	0.435	0.419	0.304	0.534	0.424	0.304	0.543	0.426	0.321	0.531
Gender	Male	(ref)	(ref)	(ref)	(ref)	(ref)	(ref)	(ref)	(ref)	(ref)	(ref)	(ref)	(ref)	(ref)	(ref)	(ref)
	Female	-0.090	-0.132	-0.047	-0.123	-0.163	-0.084	-0.133	-0.171	-0.095	-0.109	-0.147	-0.071	-0.144	-0.183	-0.105
Ethnicity	White - British	(ref)	(ref)	(ref)	(ref)	(ref)	(ref)	(ref)	(ref)	(ref)	(ref)	(ref)	(ref)	(ref)	(ref)	(ref)
	Any other White background	0.044	-0.111	0.199	-0.024	-0.154	0.106	0.081	-0.041	0.203	0.030	-0.096	0.156	0.028	-0.091	0.148
	Asian or Asian British	-0.171	-0.474	0.132	-0.213	-0.438	0.013	-0.224	-0.493	0.045	-0.070	-0.335	0.194	-0.043	-0.277	0.190
	Mixed/Dual background	0.198	0.000	0.395	-0.031	-0.197	0.134	0.010	-0.142	0.162	-0.109	-0.252	0.034	-0.058	-0.213	0.097
	Any other ethnic group	0.041	-0.258	0.339	-0.018	-0.311	0.275	0.022	-0.210	0.253	0.198	-0.016	0.412	-0.142	-0.364	0.080
SES	IMD 2010	0.399	0.158	0.641	0.325	0.098	0.551	0.426	0.215	0.638	0.383	0.163	0.602	0.431	0.228	0.635
NCMP participation (non-participation >0.2)		-0.027	-0.099	0.044	-0.023	-0.093	0.048	0.017	-0.069	0.103	0.036	-0.046	0.118	0.044	-0.068	0.156
Factor 4		-0.001	-0.039	0.037	-0.006	-0.043	0.031	0.010	-0.025	0.045	-0.014	-0.051	0.023	0.006	-0.025	0.037
Year group	Reception	(ref)	(ref)	(ref)	(ref)	(ref)	(ref)	(ref)	(ref)	(ref)	(ref)	(ref)	(ref)	(ref)	(ref)	(ref)
	Year 6	0.187	0.043	0.332	-0.022	-0.170	0.126	-0.001	-0.140	0.138	0.043	-0.093	0.180	0.014	-0.118	0.145
Year group*factor	Reception	(ref)	(ref)	(ref)	(ref)	(ref)	(ref)	(ref)	(ref)	(ref)	(ref)	(ref)	(ref)	(ref)	(ref)	(ref)
	Year 6	-0.030	-0.075	0.015	0.026	-0.021	0.073	-0.005	-0.049	0.040	0.007	-0.037	0.051	-0.006	-0.047	0.036
