# Supplementary material for: Exploring the Potential of a School Impact on Pupil Weight Status: Exploratory Factor Analysis and Repeat Cross-Sectional Study of the National Child Measurement Programme
Source: PLoS One. 2015 Dec 23;10(12):e0145128. doi: 10.1371/journal.pone.0145128 (PMC4699206; doi:10.1371/journal.pone.0145128)
Supplement: S4 File — (PDF) [file pone.0145128.s004.pdf]

Supporting file 4 to Williams, A.J., Wyatt, K.M., Williams, C.A., Logan, S., and Henley, W.E. Exploring the potential of a school impact on pupil weight status: exploratory factor analysis and repeat cross-sectional study of the National Child Measurement Programme.

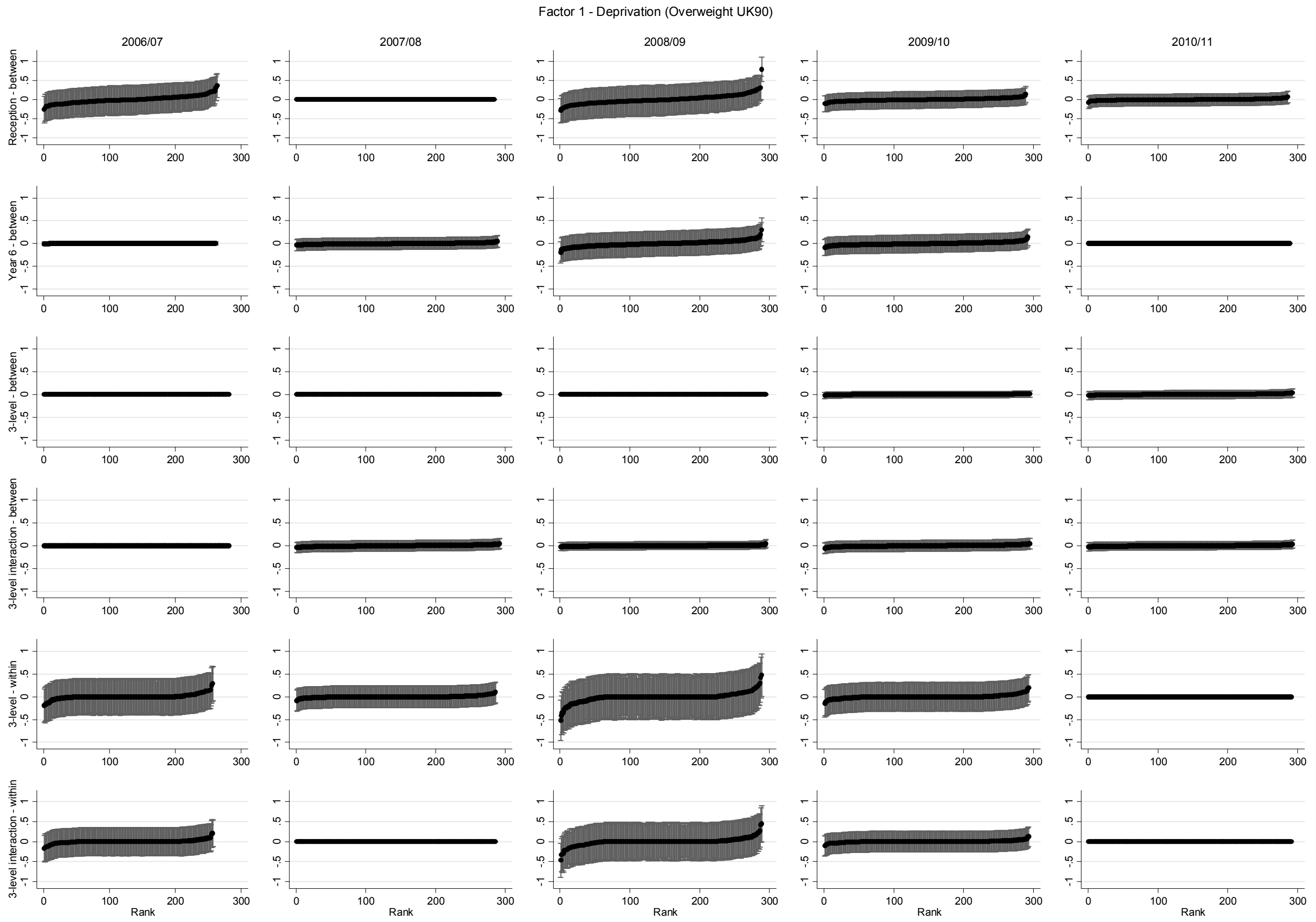

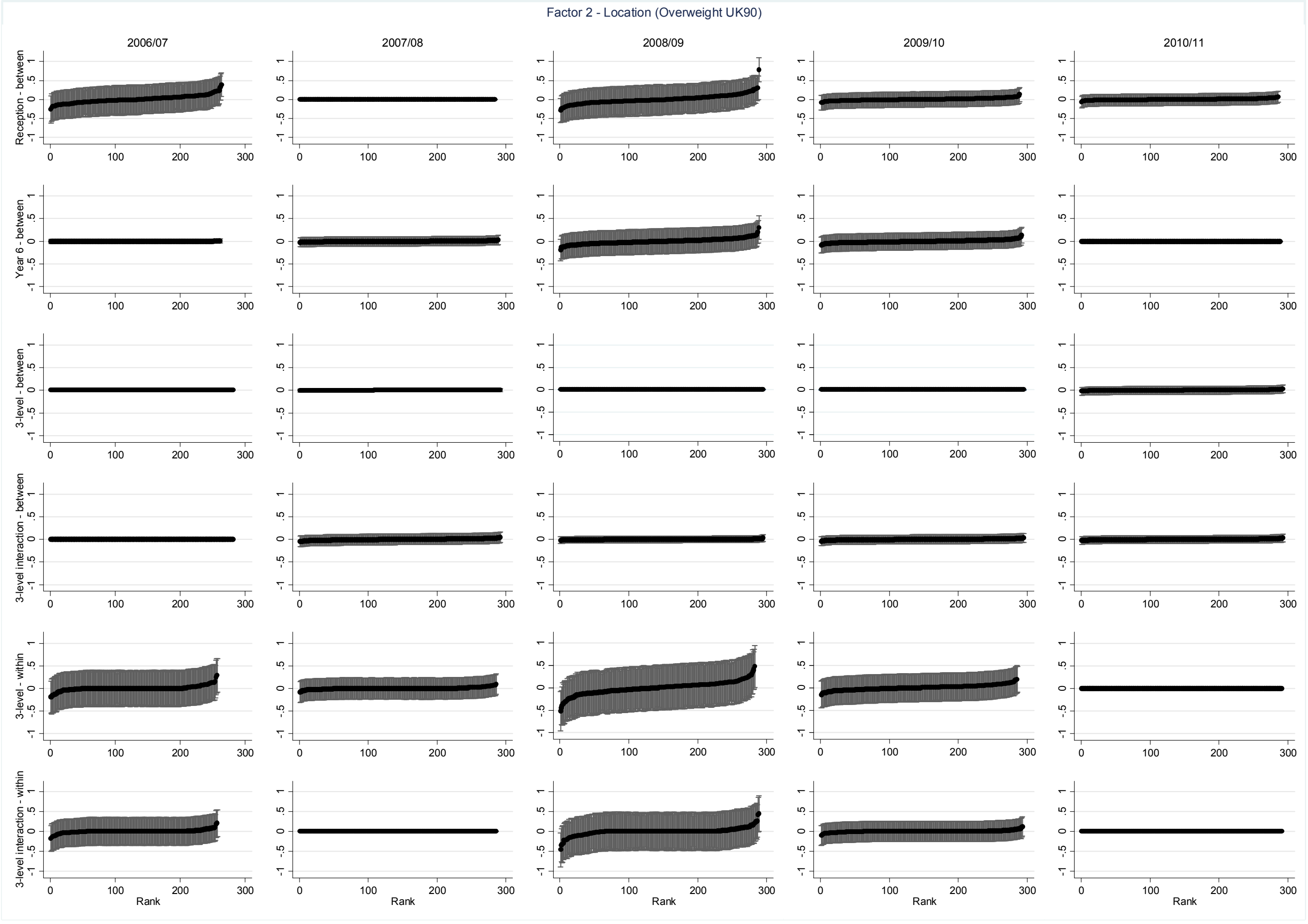

S4 file – Caterpillar plots

Supporting file 4 to Williams, A.J., Wyatt, K.M., Williams, C.A., Logan, S., and Henley, W.E. Exploring the potential of a school impact on pupil weight status: exploratory factor analysis and repeat cross-sectional study of the National Child Measurement Programme.

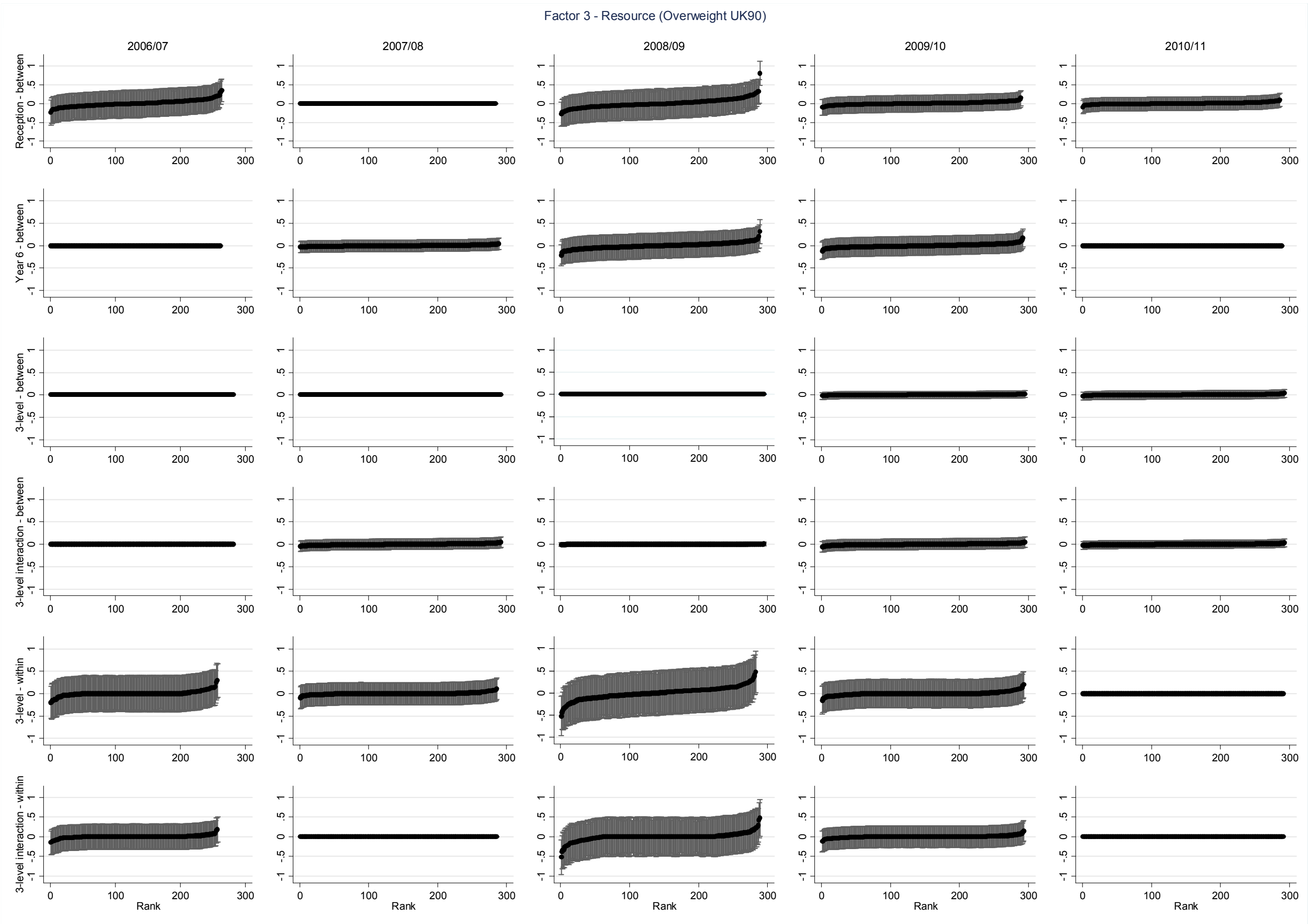

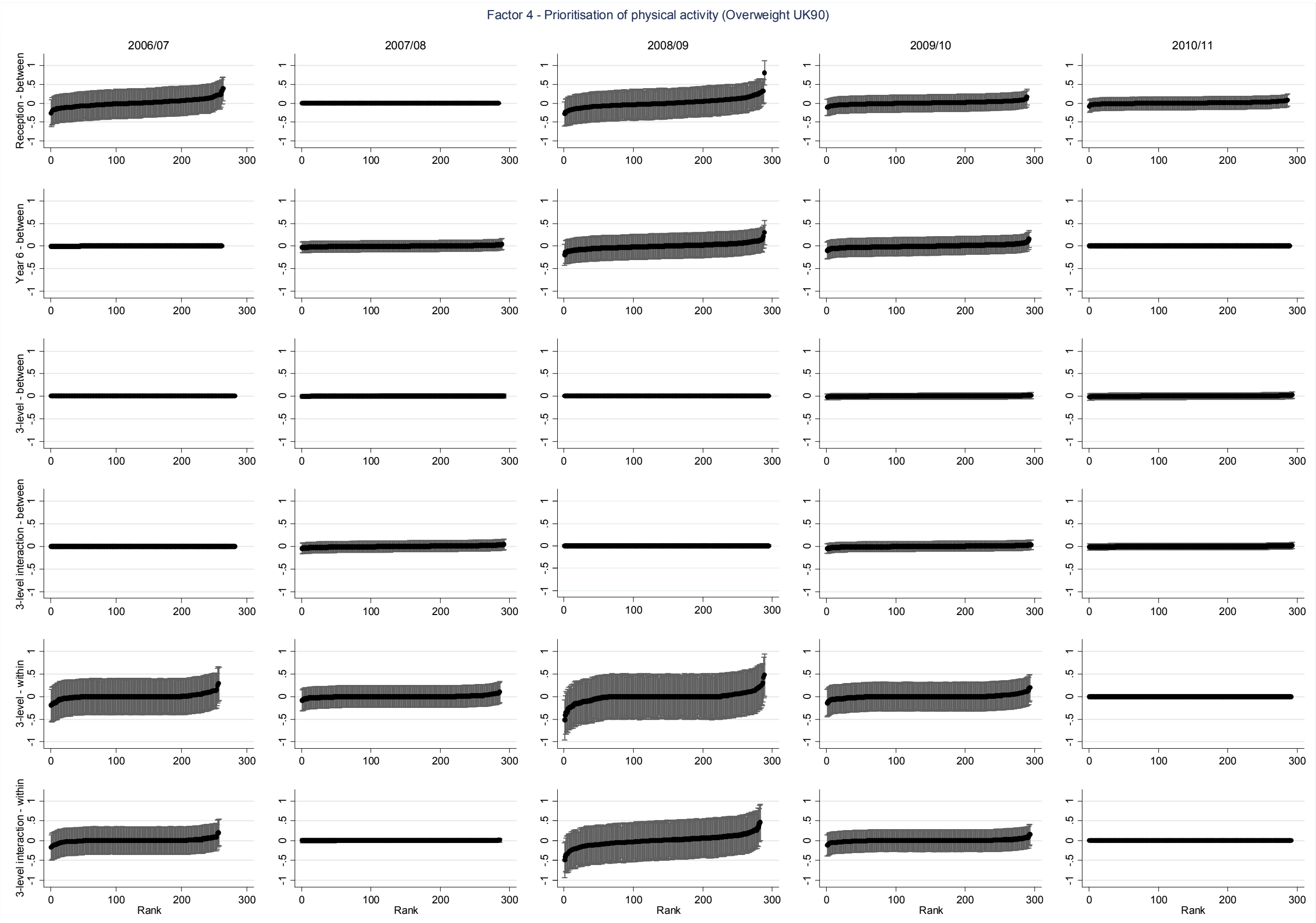

S4 file – Caterpillar plots

Supporting file 4 to Williams, A.J., Wyatt, K.M., Williams, C.A., Logan, S., and Henley, W.E. Exploring the potential of a school impact on pupil weight status: exploratory factor analysis and repeat cross-sectional study of the National Child Measurement Programme.

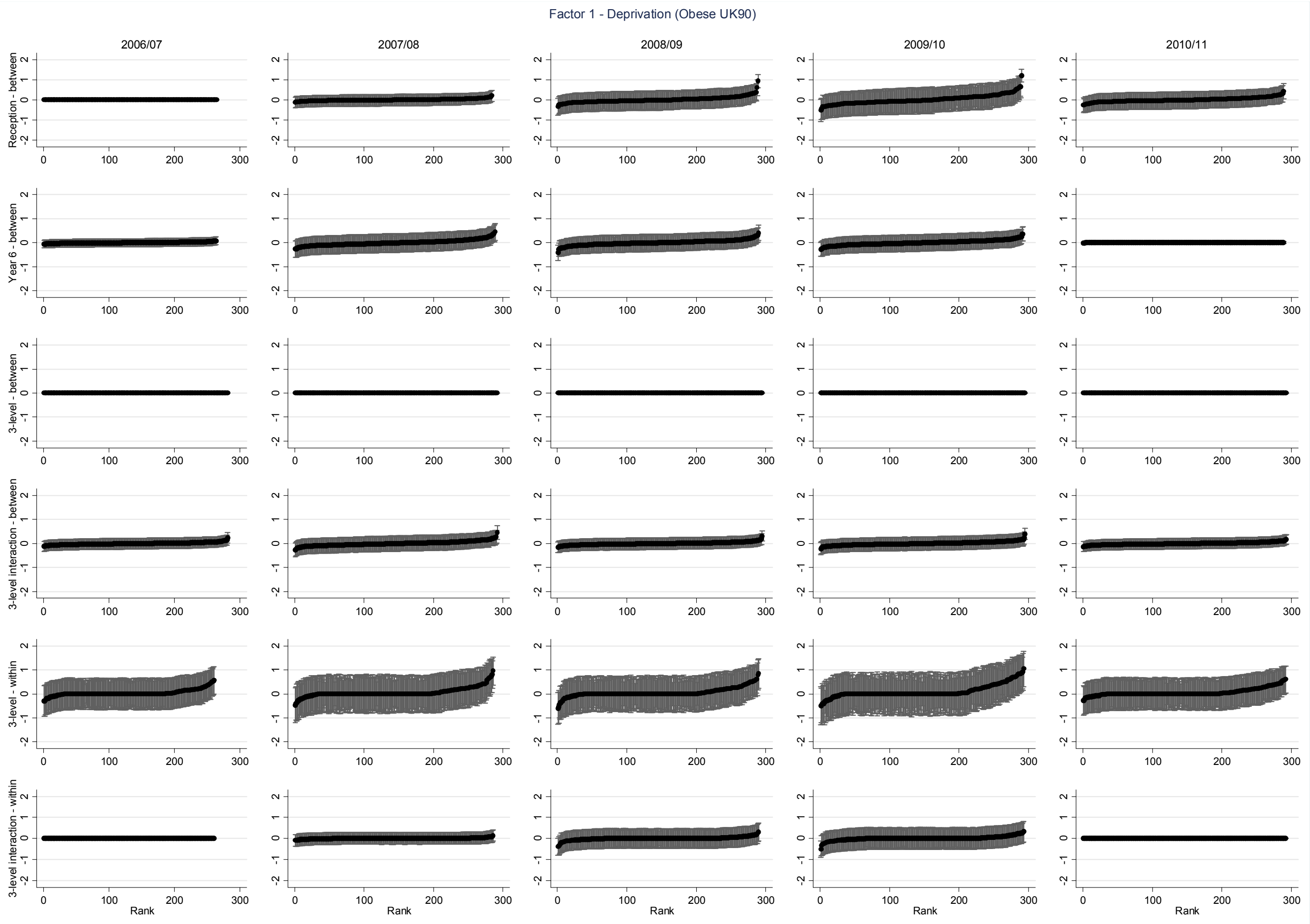

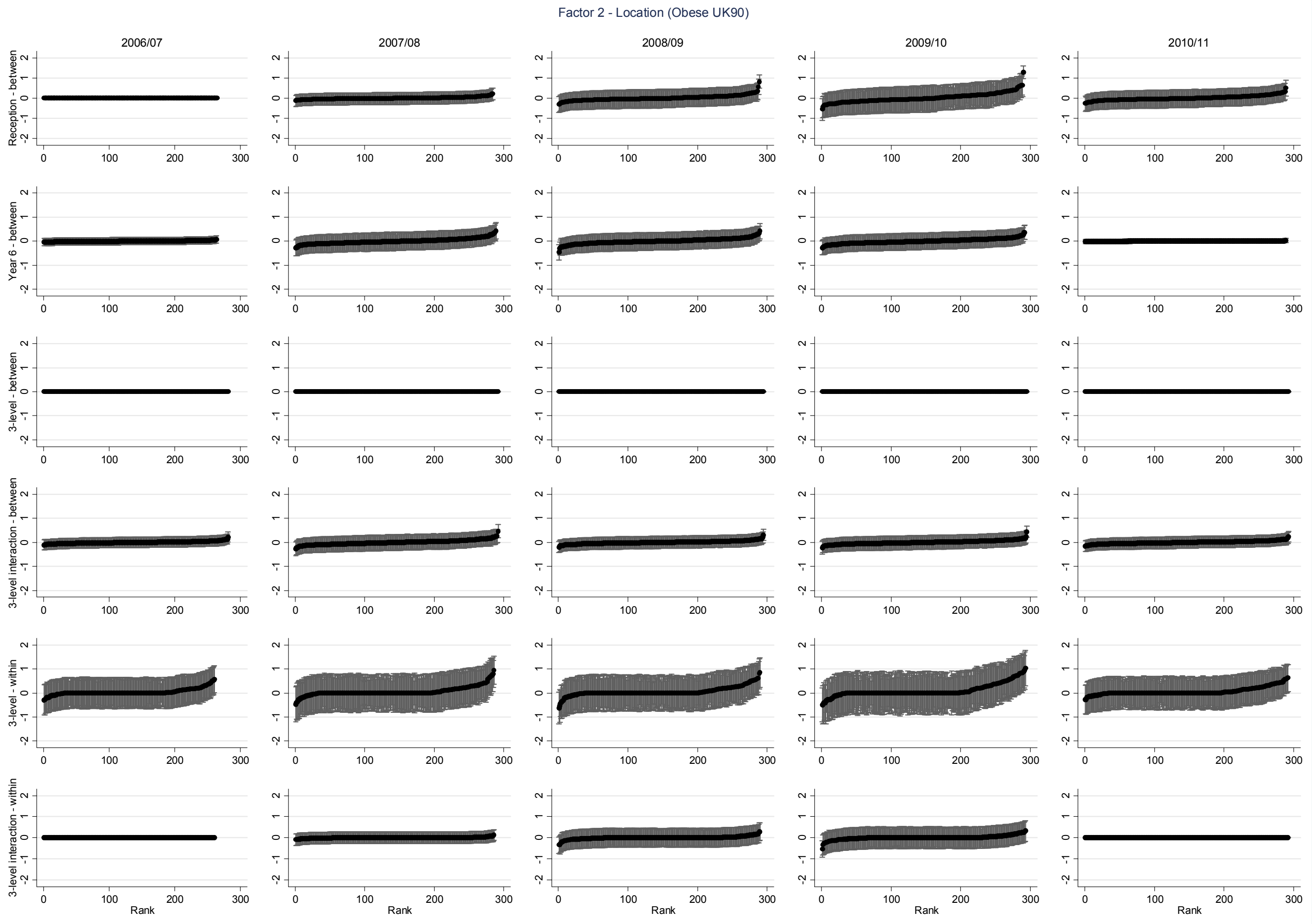

S4 file – Caterpillar plots

Supporting file 4 to Williams, A.J., Wyatt, K.M., Williams, C.A., Logan, S., and Henley, W.E. Exploring the potential of a school impact on pupil weight status: exploratory factor analysis and repeat cross-sectional study of the National Child Measurement Programme.

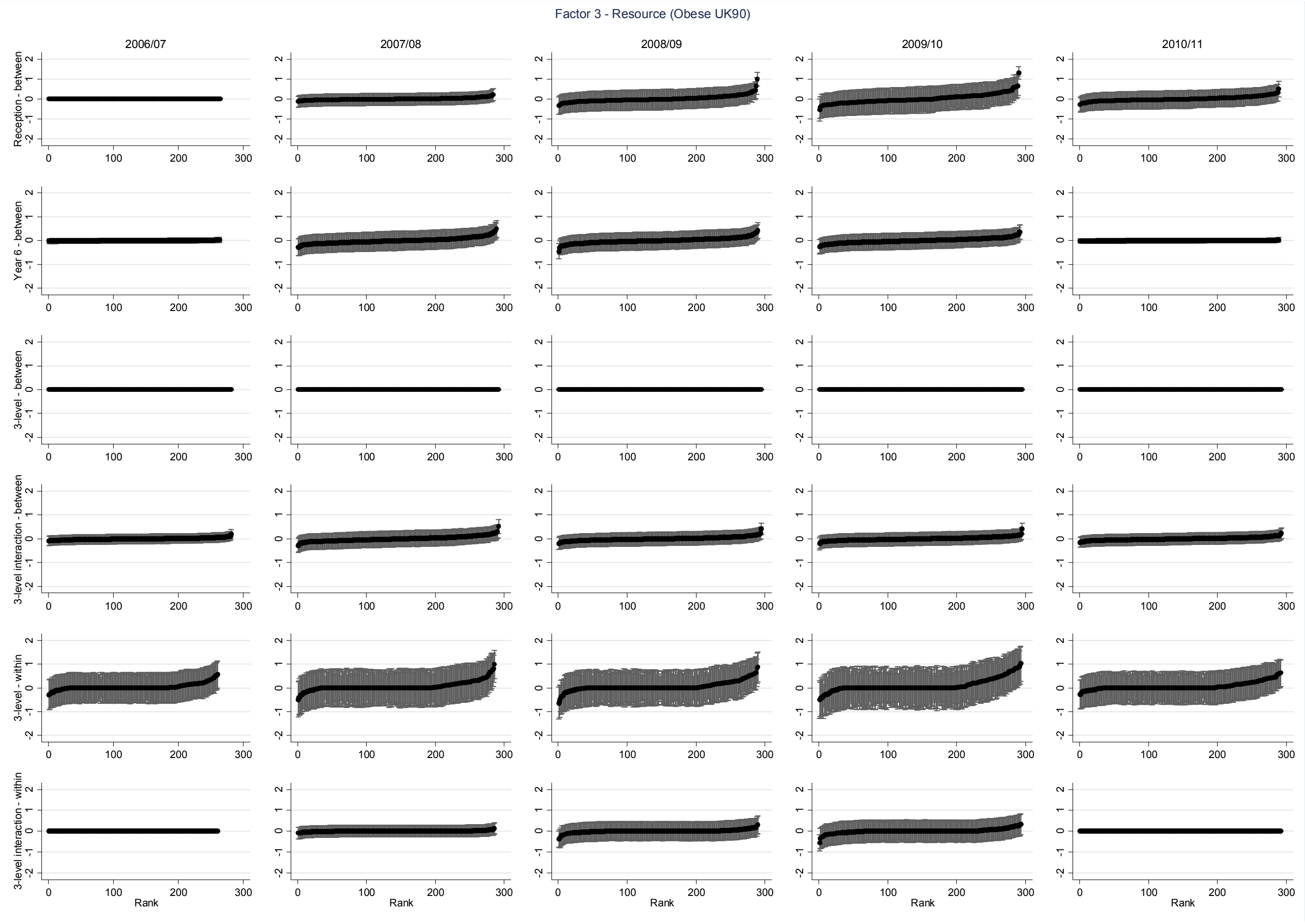

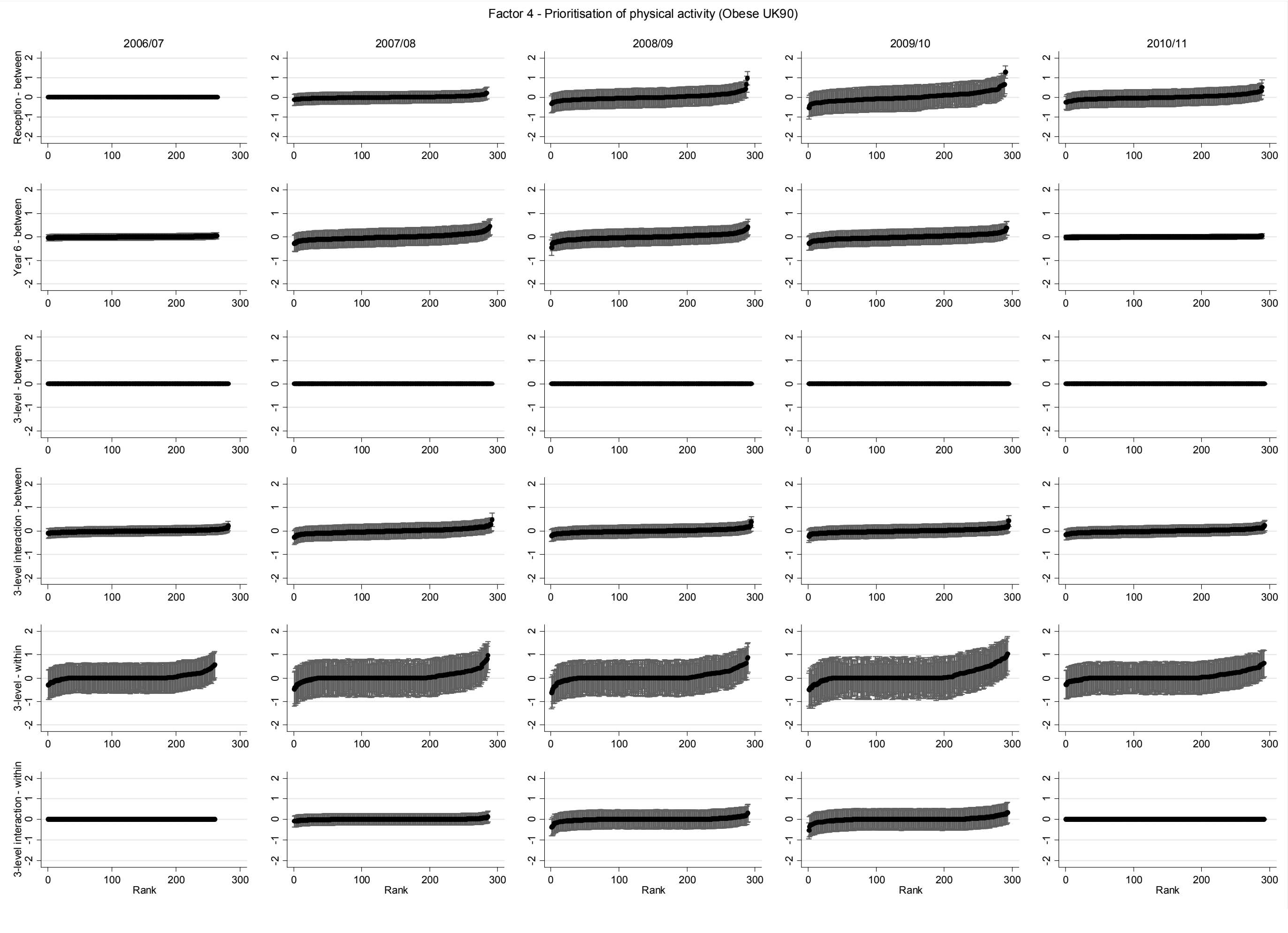

S4 file – Caterpillar plots

Types of caterpillar plot

<table>
<tr>
<td>Type 1 (≋) – Large variation, normal distribution
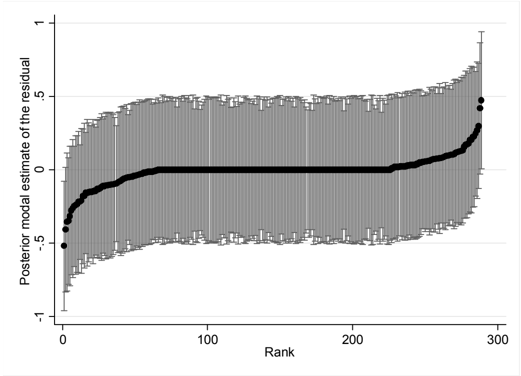

Example – Outcome; Overweight (UK90), Factor; Factor 4 – Prioritisation of physical activity, Year; 2008/09, Model; Three-level, Residuals; Difference in year group</td>
<td>Type 2 (≈) – Medium variation, normal distribution
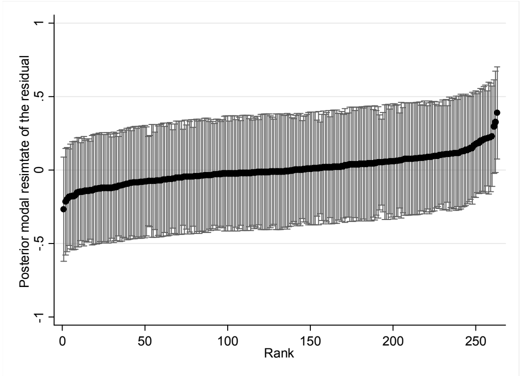

Example – Outcome; Overweight (UK90), Factor; Factor 2 – Location, Year; 2006/07, Model; Reception two-level, Residuals; School</td>
<td>Type 3 (~) – Small variation, normal distribution
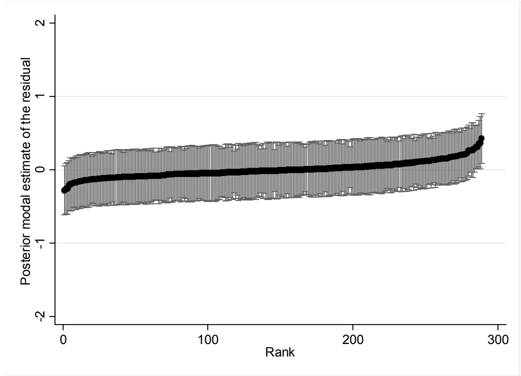

Example – Outcome; Obese (UK90), Factor; Factor 2 – Location, Year; 2007/08, Model; Year 6 two-level, Residuals; School</td>
</tr>
<tr>
<td>Type 4 (–) – 'Flat line' no variation, uniform distribution
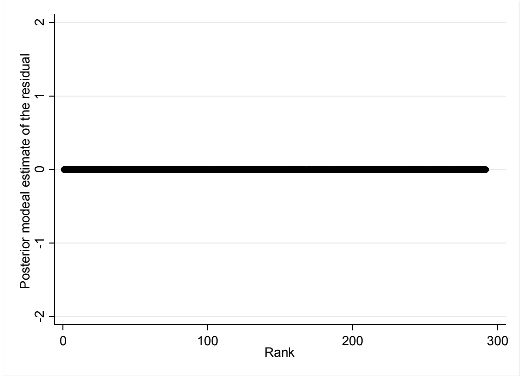

Example – Outcome; Obese (UK90), Factor; Factor 1 – Deprivation, Year; 2007/08, Model; Three-level, Residuals; School</td>
<td>Positive skew variant (+)
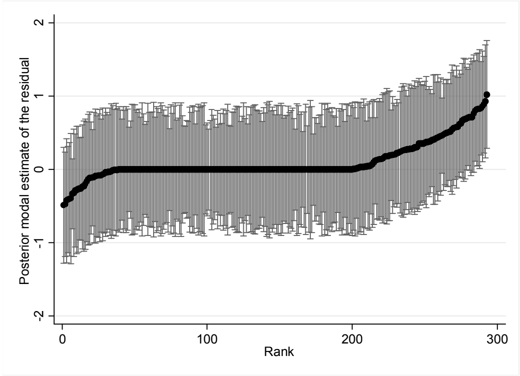

Example – Outcome; Obese (UK90), Factor; Factor 3 – Resource, Year; 2009/10, Model; Three-level, Residuals, Difference in year group</td>
<td>'Outlier' variant (°)
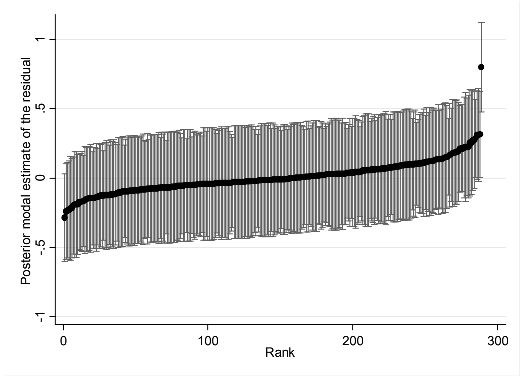

Example – Outcome; Overweight (UK90), Factor; Factor 4 – Prioritisation of physical activity, Year; 2008/09, Model; Reception two-level, Residuals, School</td>
</tr>
</table>

Confidence interval; posterior modal estimate of the residual (PME) ± 1.4 x standard error of the PME [43]
